# Palladium/Xu-Phos-catalyzed asymmetric carboamination towards isoxazolidines and pyrrolidines†

**DOI:** 10.1039/d1sc01337h

**Published:** 2021-05-05

**Authors:** Yuzhuo Wang, Lei Wang, Mingjie Chen, Youshao Tu, Yu Liu, Junliang Zhang

**Affiliations:** College of Chemistry and Life Science, Jilin Province Key Laboratory of Carbon Fiber Development and Application, Changchun University of Technology Changchun 130012 China yuliu@ccut.edu.cn; Department of Chemistry, Fudan University 2005 Songhu Road Shanghai 200438 China junliangzhang@fudan.edu.cn; Shanghai Key Laboratory of Green Chemistry and Chemical Processes, Department of Chemistry, East China Normal University 3663 N. Zhongshan Road Shanghai 200062 China

## Abstract

An efficient palladium-catalyzed enantioselective carboamination reaction of *N*-Boc-*O*-homoallyl-hydroxylamines and *N*-Boc-pent-4-enylamines with aryl or alkenyl bromides was developed, delivering various substituted isoxazolidines and pyrrolidines in good yields with up to 97% ee. The reaction features mild conditions, general substrate scope and scalability. The obtained products can be transformed into chiral 1,3-aminoalcohol derivatives without erosion of chirality. The newly identified Xu-Phos ligand bearing an *ortho*-O^i^Pr group is responsible for the good yield and high enantioselectivity.

## Abstract 2

The new chiral ligand (*S*,*Rs*)-**Xu4** with *ortho*-O^i^Pr showed good performance in the asymmetric carboamination reaction of *N*-Boc-*O*-homoallyl-hydroxylamines and *N*-Boc-pent-4-enylamines with aryl or alkenyl bromides.

## Graphical abstract

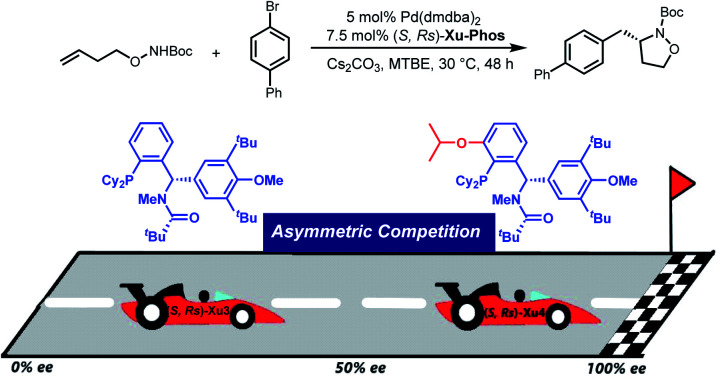

## Introduction

**Fig. 1:**
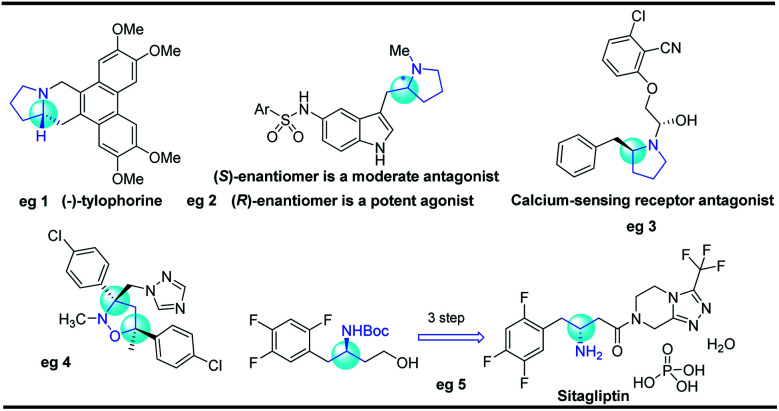
Biologically active molecules with pyrrolidine and isoxazolidine motifs.

Five-membered N-heterocyclic skeletons are commonly found in biological and pharmaceutical molecules (Fig. 1).^1^ For example, pyrrolidine-based compounds can act as receptor antagonists, and their diverse activities are determined by the different configurations (eg 2 and 3).^2^ Isoxazolidine is a versatile precursor for the synthesis of 1,3-amino alcohols, taking sitagliptin as a typical case (eg 5).^3^ Therefore, it is highly desirable to develop synthetic methods towards isoxazolidine and pyrrolidine compounds, especially for efficient construction of enantioenriched molecules.

**Scheme 1:**
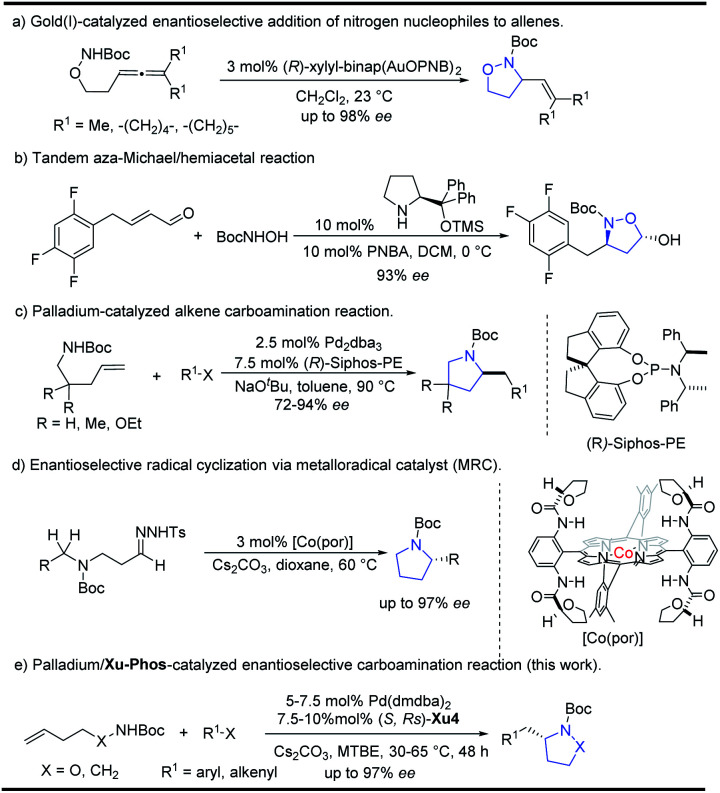
Enantioselective synthesis of isoxazolidines and pyrrolidines.

Isoxazolidines could be furnished by 1,3-dipolar cycloaddition reactions or cyclization of unsaturated hydroxylamines proceeding through electrophilic or free radical pathways, Michael addition reactions *etc.*^4^ Transition metal catalyzed cyclization strategies have also been showcased in recent years using palladium or gold as common catalysts.^5^ In 2010, Toste^6*a*^ reported the Au(i)-catalyzed enantioselective synthesis of isoxazolidines from allenic hydroxylamines, which could be also extended for the preparation of pyrazolidines and tetrahydroxazines with high enantioselectivity (Scheme 1a). Recently, Gao and co-workers reported a tandem aza-Michael/hemiacetal reaction between (*E*)-4-phenylbut-2-enal and *N*-Boc-hydroxyl-amine for the synthesis of 2-hydroxyl-isoxazolidines (Scheme 1b).^3^ The Wolfe group reported an elegant palladium-catalyzed carboamination of alkenes, which provides facile access to enantioenriched pyrrolidines (Scheme 1c).^7^ Recently, Zhang and co-workers^8^ also implemented an enantioselective radical cyclization approach by metalloradical C–H alkylation reactions (Scheme 1d).

Despite the above advances, the development of an efficient methodology for N-heterocyclic skeletons with high enantioselectivity is of great importance and still challenging, especially for the introduction of an alkenyl group. Based on our interests in the asymmetric synthesis of heterocyclic compounds,^9^ herein we wish to report our efforts in the development of palladium-catalyzed intermolecular carboamination of unsaturated hydroxylamines with aryl or alkenyl halides, in which the newly identified (*S*,*Rs*)-**Xu4** bearing an *ortho*-O^i^Pr group ligand showed a unique effect, leading to substituted isoxazolidines in relatively high yield and selectivity. Moreover, pyrrolidines could also be synthesized efficiently starting from the corresponding carbamates.

## Results and discussion

**Fig. 2:**
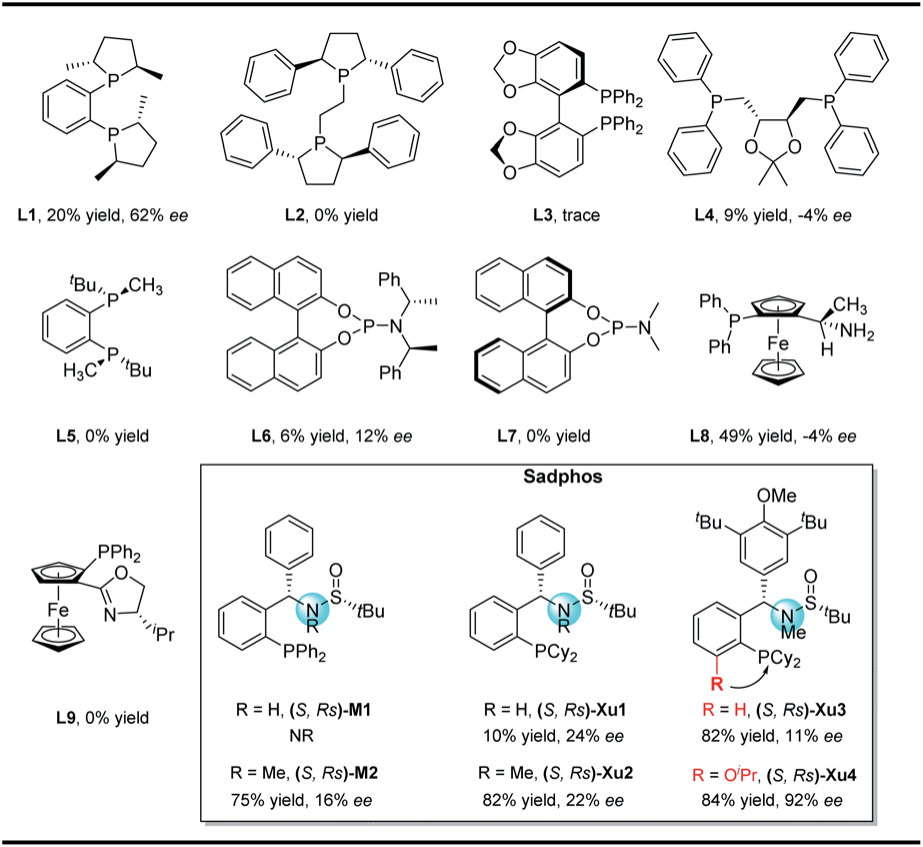
Representative chiral ligands tested in this work.

In our initial study, *N*-Boc-*O*-homoallyl-hydroxylamine **1a** and 4-bromobiphenyl were selected as the model substrates. A series of commercially available chiral ligands were investigated at first (Fig. 2). Although **L1** afforded the desired product **3a** with moderate enantioselectivity, other bisphosphine ligands **L2–L5** suppressed the reaction, with no or only a small amount of **3a** detected. The desired product could be obtained in low yield with poor enantioselectivity when using chiral phosphoramidite ligand **L6**, but **L7** is not effective at all. Josiphos **L8** delivered the product **3a** with moderate yield but as a close to racemic mixture. The phoxphos derivative **L9** is not effective for this reaction.

Inspired by the success of our developed Sadphos ligands in asymmetric catalysis, we turned our attention to evaluate their performance in the present carboamination reaction. Of note, the Sadphos kits are commercially available from Strem Inc. now. As an initial trial, Ming-Phos^9*h*,10^ (*S*,*Rs*)-**M1** with free NH was inactive, leaving the starting materials untouched. Surprisingly, when the amine moiety was protected by a methyl group ((*S*,*Rs*)-**M2**), **3a** was obtained in 75% yield, albeit the enantioselectivity was unsatisfactory, indicating that the NH moiety of Ming-Phos might inhibit carboamination. The dicyclohexyl phosphine ligand Xu-Phos^11^ showed a consistent trend, and (*S*,*Rs*)-**Xu2** bearing a *N*-methyl group resulted in higher yield and enantioselectivity. Inspired by these notable results, we investigated the modification of Xu-Phos ligand. (*S*,*Rs*)-**Xu3** bearing a 3,5-di-*tert*-butyl-4-methoxybenzyl group did not provide a better result. Amazingly, the introduction of O^i^Pr at the *ortho*-position of the phosphine moiety dramatically improved the enantioselectivity, delivering **3a** in 84% yield and 92% ee.^13^ These results unambiguously prove the subtleness and unique efficacy of the Xu-Phos ligands. We attribute this ortho-effect to the repulsion of the iso-propyl group with the cyclohexyl group on the P-atom, which would push the cyclohexyl group close to the catalytic center and affect the enantioselectivity.

Other factors were also systematically examined consequently. Among the palladium sources tested (Table 1), PdCl_2_, Pd(OAc)_2_, Pd(MeCN)_2_Cl_2_ and [Pd(allyl)Cl]_2_ were obviously less active, generating **3a** in lower yields (Table 1, entries 1–4). Pd_2_(dba)_3_ performed similarly to Pd(dmdba)_2_, with slightly reduced yield (Table 1, entry 5). Other bases including NaO^*t*^Bu, KO^*t*^Bu and K_2_CO_3_ led to lower yields (Table 1, entries 7–9). Various solvents were also screened, and MTBE was demonstrated to be the best choice (entries 10–14). To our delight, the enantioselectivity was further improved by lowering the temperature, and the yield could also be promoted with prolonged reaction time (Table 1, entries 15 and 16). Finally, using Pd(dmdba)_2_/(*S*,*Rs*)-**Xu4** as the catalyst and Cs_2_CO_3_ as the base, the reaction proceeds smoothly in MTBE at 30 °C to afford the desired product in 88% yield and 95% ee (Table 1, entry 16).

**Table 1:** Optimization of the reaction conditions^a^

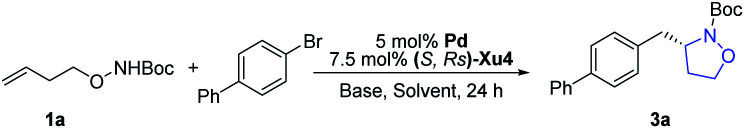					
Entry	Pd sources	Base	Solvent	Yield^b^ (%)	ee^c^ (%)
1	PdCl_2_	Cs_2_CO_3_	MTBE	55	85
2	Pd(OAc)_2_	Cs_2_CO_3_	MTBE	64	80
3	Pd(MeCN)_2_Cl_2_	Cs_2_CO_3_	MTBE	24	91
4^f^	[Pd(allyl)Cl]_2_	Cs_2_CO_3_	MTBE	70	92
5^f^	Pd_2_(dba)_3_	Cs_2_CO_3_	MTBE	82	92
6	Pd(dmdba)_2_	Cs_2_CO_3_	MTBE	84	92
7	Pd(dmdba)_2_	NaO^*t*^Bu	MTBE	49	90
8	Pd(dmdba)_2_	KO^*t*^Bu	MTBE	38	91
9	Pd(dmdba)_2_	K_2_CO_3_	MTBE	40	91
10	Pd(dmdba)_2_	Cs_2_CO_3_	Toluene	59	89
11	Pd(dmdba)_2_	Cs_2_CO_3_	THF	78	90
12	Pd(dmdba)_2_	Cs_2_CO_3_	DCM	79	88
13	Pd(dmdba)_2_	Cs_2_CO_3_	MeCN	63	86
14	Pd(dmdba)_2_	Cs_2_CO_3_	DMF	79	89
15^d^	Pd(dmdba)_2_	Cs_2_CO_3_	MTBE	82	93
16^e^	Pd(dmdba)_2_	Cs_2_CO_3_	MTBE	88	95

a Reaction conditions: **1a** (0.2 mmol), 4-bromobiphenyl (0.4 mmol), Cs_2_CO_3_ (2 equiv.), 5 mol% Pd, and 7.5 mol% ligand in 2.0 mL MTBE at 75 °C under Ar for 24 h.

b Isolated yield.

c ee was determined by HPLC analysis.

d 50 °C, 24 h.

e 30 °C, 48 h.

f 2.5 mol% Pd.

### Enantioselective synthesis of substituted isoxazolidines

**Scheme 2:**
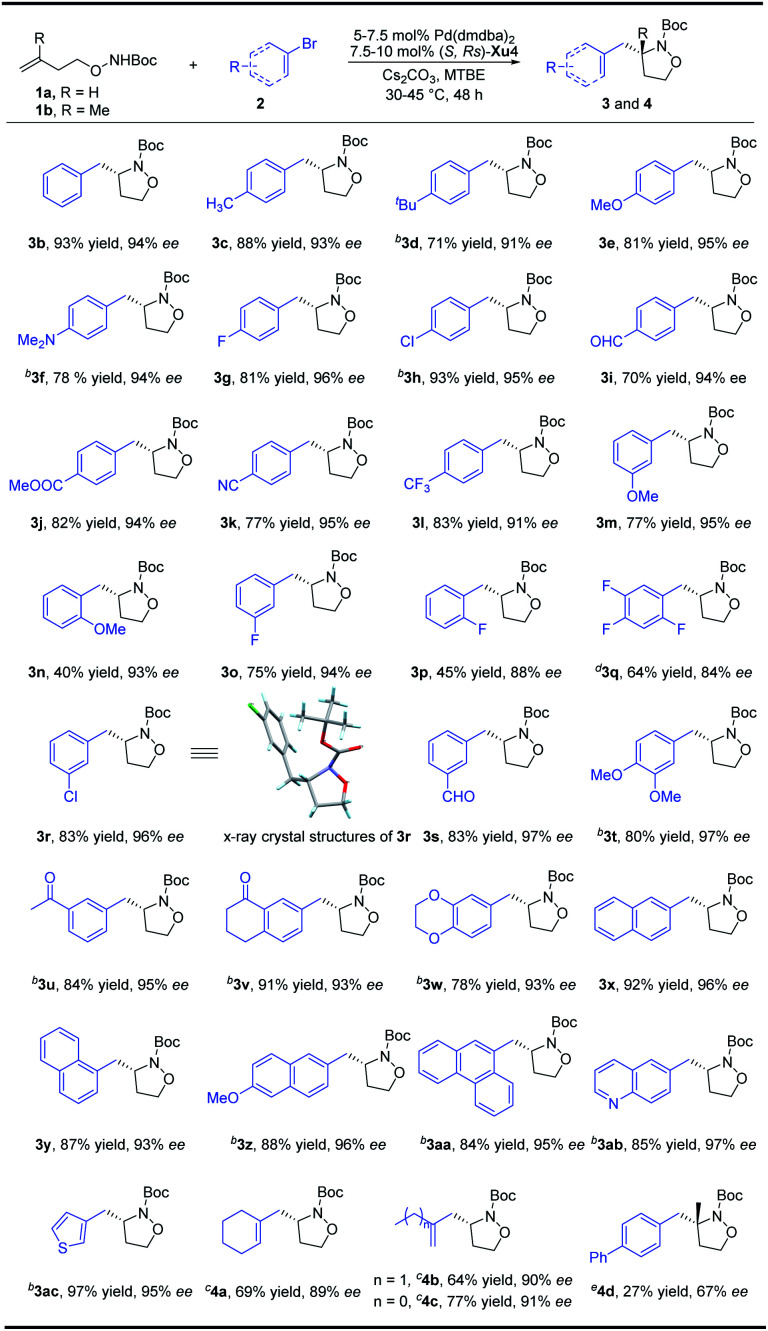
Synthesis of substituted isoxazolidines. ^a^Unless otherwise noted, all reactions were carried out with **1a** (0.2 mmol), aryl bromides (0.4 mmol), Cs_2_CO_3_ (2 equiv.), 5 mol% Pd(dmdba)_2_, and 7.5 mol% ligand in 2.0 mL MTBE at 30 °C under Ar for 48 h. ^b^**1a** (0.4 mmol), aryl bromides (0.8 mmol), 4.0 mL MTBE. ^c^**1a** (0.4 mmol), alkenyl bromides (0.8 mmol), Cs_2_CO_3_ (2 equiv.), 7.5 mol% Pd(dmdba)_2_, and 10 mol% ligand in 4 mL MTBE at 45 °C under Ar for 48 h. ^d^**1a** (0.4 mmol), aryl bromides (0.8 mmol), 4.0 mL MTBE, 75 °C, 40 h. ^e^**1b** (0.4 mmol), 65 °C.

With the optimized reaction conditions in hand, a variety of aryl bromides were reacted with *N*-Boc-*O*-homoallyl-hydroxylamine **1a** to verify the generality of the reaction system (Scheme 2). Both electron-donating and electron-withdrawing groups at the *para*-position of the aryl bromides are compatible, providing isoxazolidines **3a–3h** with 91–96% ee. When 4-biphenylyl trifluoromethanesulfonate is used instead of *p*-bromobiphenyl at 75 °C, **3a** can be prepared with 81% yield and 91% ee. In addition to halogens, electron-withdrawing groups at the *para*-position of the benzene ring such as an aldehyde group, ester group, cyano group, and trifluoromethyl group are all tolerated, delivering products **3i–3l** in good yields with 91–95% ee. Aryl bromides bearing different substituents such as OMe, F, Cl, CHO, or COCH_3_ at the *meta*-position could also efficiently give the desired products **3m**, **3o**, **3r**, **3s** and **3u** with up to 97% ee. The absolute configuration of **3r** was confirmed by X-ray crystallography analysis.^13^ Moreover, when a methoxy or fluorine substituent was located at the *ortho*-position of the phenyl bromide, the products **3n** and **3p** were obtained with 93% and 88% ee, respectively in moderate yields. Disubstituted phenyl bromides could also be transformed smoothly, and the corresponding products **3t**, **3v** and **3w** were furnished in 78–91% yields with up to 97% ee. The trisubstituted compound **3q** on the phenyl ring can also be obtained in 64% yield and 84% ee. Naphthyl and phenanthryl isoxazolidines were furnished in notably high yields and enantioselectivity (**3x–3aa**). Moreover, heteroaromatic rings including quinolinyl and thienyl could also be well tolerated, affording **3ab** and **3ac** with no inferior effects. The introduction of an alkenyl group to the molecule could increase the diversity of the compounds due to the alkenyl group having abundant functional group transformations. To our delight, the alkenyl group could also be transferred into the final products from the corresponding alkenyl bromides by slight adjustment of the catalyst loading. Both cyclic and linear precursors performed gratifyingly, delivering the desired products **4a–4c** in good yields with up to 91% ee. A preliminary and promising result showed that the present method is also promising for the synthesis of isoxazolidine **4d** with an aza-quaternary carbon stereocenter, albeit the efficiency and enantioselectivity are not satisfactory yet and further modification of the chiral ligand is necessary.

### Enantioselective synthesis of aryl substituted pyrrolidines

**Fig. 3:**
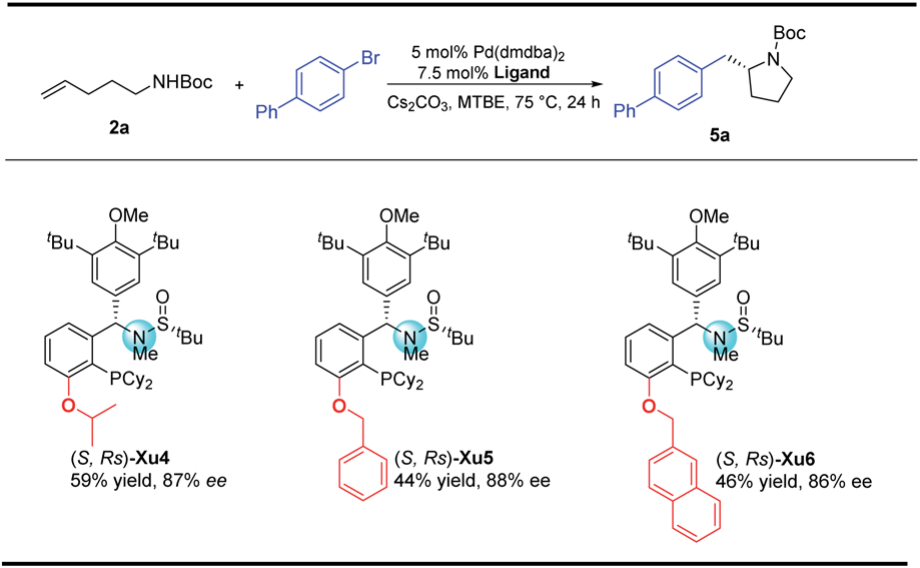
Screening ligands in the synthesis of pyrrolidines.

**Scheme 3:**
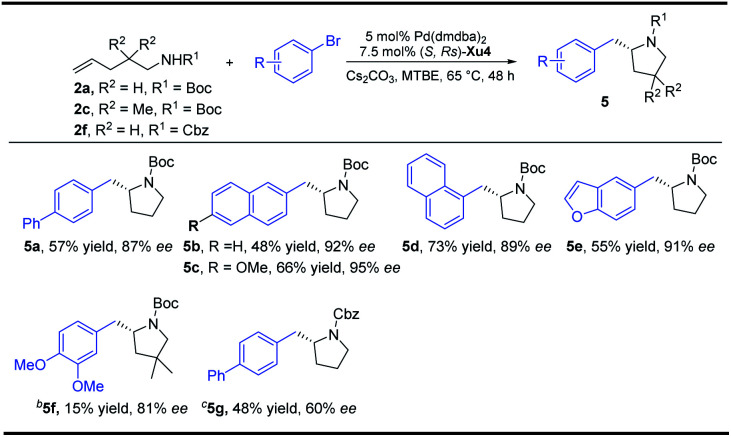
Synthesis of substituted pyrrolidines. ^a^Unless otherwise noted, all reactions were carried out with **2a** (0.4 mmol), aryl bromides (0.8 mmol), Cs_2_CO_3_ (2 equiv.), 5 mol% Pd(dmdba)_2_ and 7.5 mol% ligand in 4.0 mL MTBE at 65 °C under Ar for 48 h. ^b^**2c** (0.2 mmol), 20 h. ^c^**2f** (0.2 mmol), 24 h.

With regard to the importance of pyrrolidine derivatives, we next turned to investigate this catalyst system in the asymmetric carboamination reaction of C-linked alkenyl carbamates (Fig. 3). The desired product **5a** was obtained with similar enantioselectivity with the use of (*S*,*R*s)-**Xu4**, (*S*,*R*s)-**Xu5** and (*S*,*R*s)-**Xu6** with different *ortho*-substituents, among which (*S*,*R*s)-**Xu4** gives the highest yield. Various substituted pyrrolidines **5a–5d** were delivered in moderate to good yields with high enantioselectivity (Scheme 3). The benzofuranyl group (**5e**) could also be introduced into the final product easily. We next investigated the substituent effect on the alkyl chain and the corresponding product **5f** was produced in low yield (15%) and relatively lower enantioselectivity (81% ee). The amide moiety also affects the reaction significantly, for instance, the Cbz-derived **5g** was delivered in only 48% yield with 60% ee. The tosylated substrate produced the corresponding *N*-arylation product rather than the carboamination product, indicating that these two reaction pathways are competitive.

**Scheme 4:**
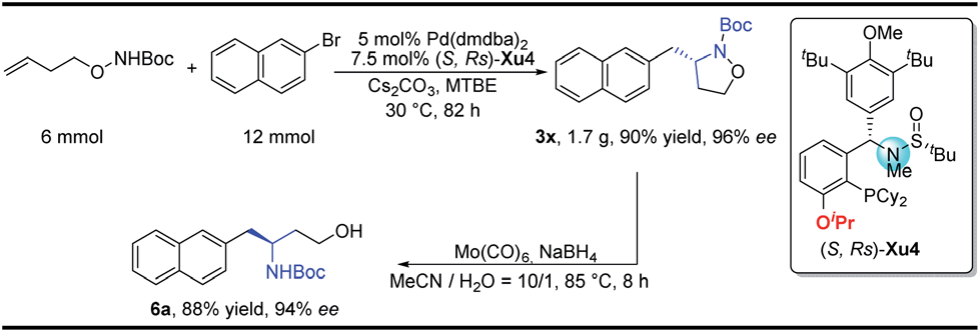
Gram-scale synthesis and synthetic applications.

To demonstrate the practicability of our protocol, a 6 mmol scale reaction was carried out under standard conditions, delivering 1.7 g of naphthyl isoxazolidine **3x** in 90% yield with 96% ee. Further treatment with NaBH_4_ furnished chiral aminoalcohol **9a** in 88% yield with 94% ee (Scheme 4).^12^

**Scheme 5:**
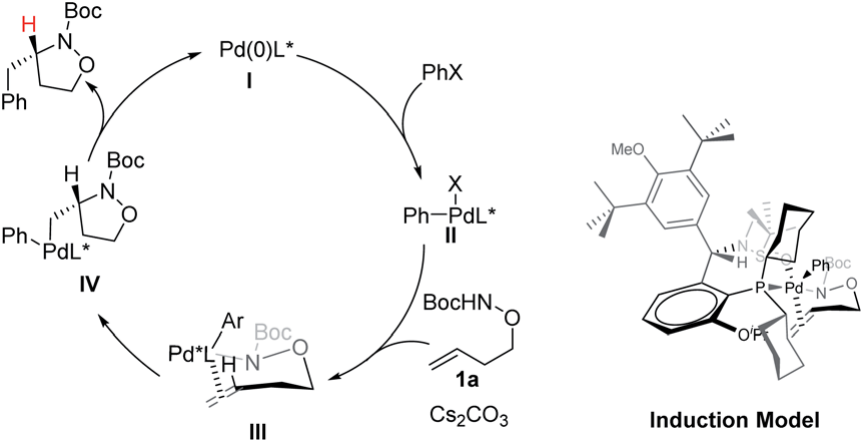
Catalytic cycle and chirality induction model.

A catalytic cycle and asymmetric induction model were proposed in Scheme 5. The oxidative addition of aryl bromides to the (*S*,*R*s)-**Xu4**/Pd(0) complex would generate Pd(ii) species **II**. In the presence of a base, species **II** would form a Pd–N bond to deliver the intermediate **III**, which undergoes insertion or aminopalladation of the alkene to produce the intermediate **IV**. The final product was obtained *via* reductive elimination, and the catalytic species were also regenerated. Xu-Phos and Pd coordinate through P on the ligand and O on the sulfinamide to form the corresponding catalytic center. The cyclohexyl group is in the sensitive area of metal active species. The introduction of O^i^Pr as a side arm group may push the cyclohexyl group closer to the catalytic center and produce a key dynamic steric hindrance effect.

## Conclusions

In summary, we have successfully developed a palladium-catalyzed asymmetric carboamination reaction of *N*-Boc-*O*-homoallyl-hydroxylamine and *N*-Boc-pent-4-enylamine with either aryl or alkenyl bromides under mild reaction conditions, furnishing various substituted isoxazolidines and pyrrolidines in moderate to high yields with high enantioselectivity. The newly identified ligand of (*S*,*Rs*)-**Xu4** with O^i^Pr at the *ortho*-position, which is easily prepared from commercially available starting materials, is responsible for the general substrate scope, good yield and high enantioselectivity. The application of this chiral ligand in other transition metal asymmetric reactions is ongoing in our lab.

## Author contributions

Y. Wang, L. Wang, M. Chen and Tu did the experiments and collected the data. Y. Liu and J. Zhang directed the research. Y. Wang, Y. Liu and J. Zhang wrote the manuscript.

## Conflicts of interest

There are no conflicts to declare.

## Supplementary Material

SC-012-D1SC01337H-s001

SC-012-D1SC01337H-s002
