# Supplementary material for: Palladium/Xu-Phos-catalyzed asymmetric carboamination towards isoxazolidines and pyrrolidines
Source: Chem Sci. 2021 May 5;12(23):8241–5. doi: 10.1039/d1sc01337h (PMC8208297; doi:10.1039/d1sc01337h)
Supplement: SC-012-D1SC01337H-s001 [file SC-012-D1SC01337H-s001.pdf]

Electronic Supplementary Material (ESI) for Chemical Science.
This journal is © The Royal Society of Chemistry 2021

Supporting Information

Palladium/Xu-Phos-Catalyzed Asymmetric Carboamination towards Isoxazolidines and Pyrrolidines

Yuzhuo Wang, Lei Wang, Mingjie Chen, Youshao Tu, Yu Liu* and Junliang Zhang*

Table of Contents

1. General Information....................S3

2. General Procedure....................S3

3. Screening the Known Ligands....................S6

4. Optimization of the Reaction Conditions....................S7

5. General Procedure for Preparation of Substrates **1a** and **2a**....................S8

6. Synthesis of (*S*, *Rs*)-**Xu4**....................S11

7. Gram-scale Synthesis of **3x** and Synthetic Applications....................S15

8. Supplementary experiment....................S16

9. Enantioselective Synthesis of Aryl Substituted Isoxazolidines....................S21

10. Enantioselective Synthesis of Alkenyl Substituted Isoxazolidines....................S43

11. Enantioselective Synthesis of Aryl Substituted Pyrrolidines....................S46

12. ^{1}H, ^{19}F, ^{31}P, ^{13}C NMR S51

13. Crystal Data Compounds of **3r** and (*S*, *Rs*)-**Xu4**....................S118

14. References....................S119

1. General Information.

Unless otherwise noted, all reactions were carried out under a argon atmosphere; materials obtained from commercial suppliers were used directly without further purification. The [±]D was measured on an Autopol III S2, Serial #32008. Manufactured by Rudolph Research Analytical, Hackettstown, NJ, USA. ^{1}H NMR spectra, ^{13}C NMR spectra, and ^{31}P NMR spectra were recorded on a Bruker 300 MHz, 400 MHz and 500 MHz spectrometer in $CDCl_3$. NMR experiments are reported in δ units, parts per million (ppm), and were referenced to $CDCl_3$ (δ 7.26 or 77.0 ppm) as the internal standard. The data is being reported as (s = singlet, d = doublet, dd = doublet of doublet, t = triplet, m = multiplet or unresolved, br = broad signal, coupling constant(s) in Hz, integration).Tetrahydrofuran (THF), toluene, hexane and ether were dried with sodium benzophenone and distilled before use; Reactions were monitored by thin layer chromatography (TLC) using silicycle pre-coated silica gel plates. Flash column chromatography was performed on silica gel 60 (particle size 300-400 mesh ASTM, purchased from Yantai, China) and eluted with petroleum ether/ethyl acetate. The absolute configuration of products is inferred from the single crystal data of **3r**

2. General Procedure.

2.1. General procedure for synthesis of chiral isoxazolines.

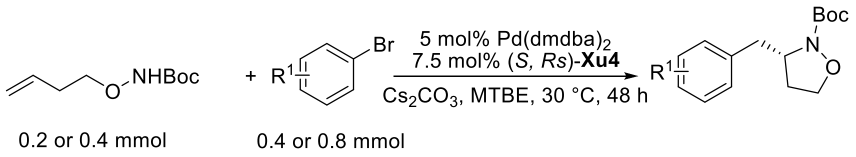

A schlenk tube (25 mL) equipped with a magnetic stir bar was charged with appropriate Phos (0.015 or 0.03 mmol), Pd(dmdba)$_2$ (0.01 or 0.02 mmol) and 2 or 4 mL *tert*-butyl methyl ether. After the catalyst/ligand solution was stirred for 30-60 min at 25 ℃. *N*-Boc-*O*-homoallyl-hydroxylamine (0.2 or 0.4 mmol), aryl bromines (0.4 or 0.8 mmol), Cs_2CO_3 (0.4 or 0.8 mmol) were successively added. Under argon atmosphere, the reaction mixture was kept stirring at 30 ℃ for 48 h. After completion of the reaction (monitored by TLC), the mixture was concentrated in vacuum and the residue was purified by flash column chromatography on silica gel with petroleum ether - ethyl acetate as eluent to give the desired product.

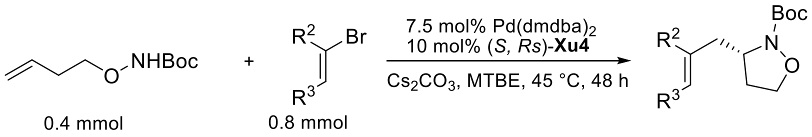

A schlenk tube (25 mL) equipped with a magnetic stir bar was charged with appropriate Phos (0.04 mmol), Pd(dmdba)$_2$ (0.03 mmol) and 4 mL *tert*-butyl methyl ether. After the catalyst/ligand solution was stirred for 30-60 min at 25 ℃. *N*-Boc-*O*-homoallyl-hydroxylamine (0.4 mmol), aryl bromines (0.8 mmol), Cs_2CO_3 (0.8 mmol) were successively added. Under argon atmosphere, the reaction mixture was kept stirring at 45 ℃. for 48 h. After completion of the reaction (monitored by TLC), the mixture was concentrated in vacuum and the residue was purified by flash column chromatography on silica gel with petroleum ether - ethyl acetate as eluent to give the desired product.

2.2. General procedure for synthesis of chiral pyrrolidines.

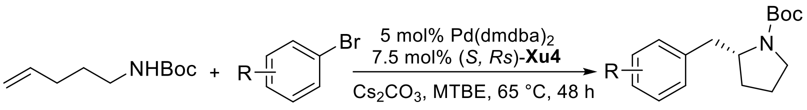

A schlenk tube (25 mL) equipped with a magnetic stir bar was charged with appropriate Phos (0.03 mmol), Pd(dmdba)$_2$ (0.02 mmol) and 4 mL *tert*-butyl methyl ether. After the catalyst/ligand solution was stirred for 30-60 min at 25 ℃. *N*-(boc)-pent-4-enylamine (0.4 mmol), aryl bromines (0.8 mmol), Cs_2CO_3 (0.8 mmol) were successively added. Under argon atmosphere, the reaction mixture was kept stirring at 65 ℃. for 48 h. After completion of the reaction (monitored by TLC), the mixture was concentrated in vacuum and the residue was purified by flash column chromatography on silica gel with petroleum ether - ethyl acetate as eluent to give the desired product.

2.3. General procedure for synthesis of racemic isoxazolidines.[1]

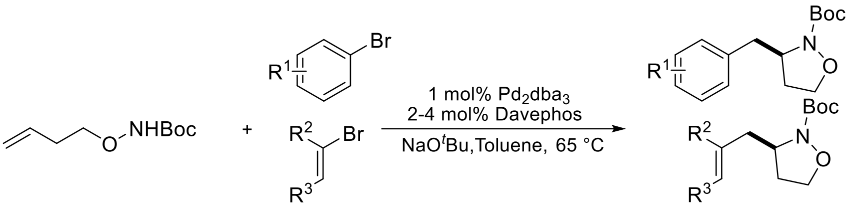

To a solution of Pd_2dba_3 (1.83 mg, 0.002 mmol) in toluene (2.0 mL) was added Davephos (1.57-3.14 mg, 0.004-0.008 mmol). After the catalyst/ligand solution was stirred for 30-60 min at 25 ℃. *N*-Boc-*O*-homoallyl-hydroxylamine (0.2 mmol), aryl bromines (0.4 mmol), NaO^{t}Bu (38.44 mg, 0.4 mmol) were successively added. Under argon atmosphere, the reaction mixture was stirred overnight at 65 °C. After completion of the reaction (monitored by TLC), the mixture was concentrated in vacuum and the residue was purified by flash column chromatography on silica gel with petroleum ether - ethyl acetate as eluent to give the desired product.

2.4. General procedure for synthesis of racemic pyrrolidines.[2]

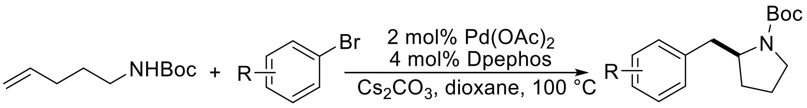

To a solution of $Pd(OAc)_2$ (0.9 mg, 0.004 mmol) in dioxane (2.0 mL) was added Dpephos (4.3 mg, 0.008 mmol).After the catalyst/ligand solution was stirred for 30-60 min at 25 ℃. *N*-(boc)-pent-4-enylamine (0.2 mmol), aryl bromines (0.4 mmol),Cs_2CO_3 (130 mg, 0.4 mmol),were successively added. Under argon atmosphere, the reaction mixture was stirred overnight at 100 °C. After completion of the reaction (monitored by TLC), the mixture was concentrated in vacuum and the residue was purified by flash column chromatography on silica gel with petroleum ether-ethyl acetate as eluent to give the desired product.

3. Screening the Known Ligands.

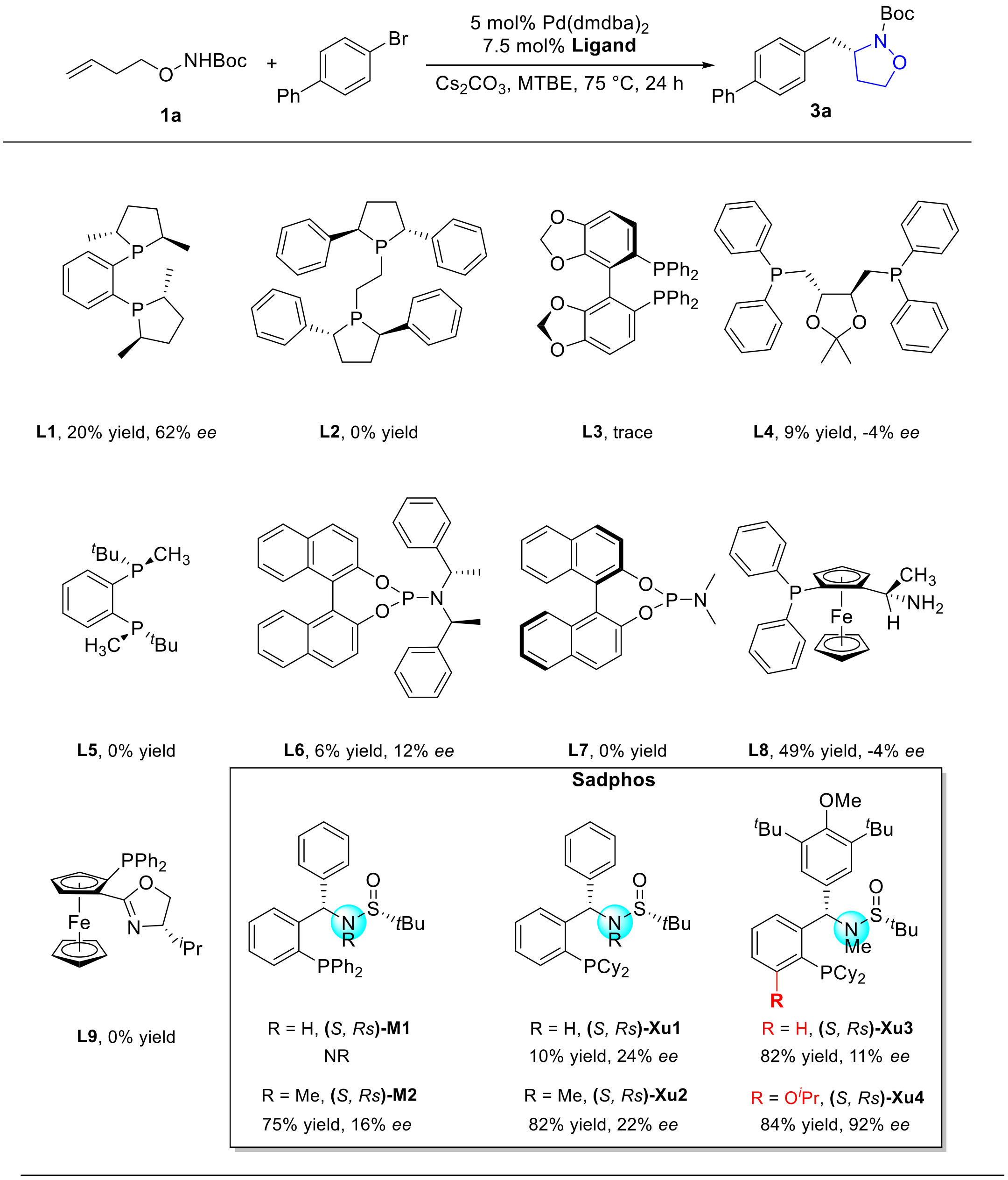

A Schlenk tube (25 mL) equipped with a magnetic stir bar was charged with appropriate Phos (0.015 mmol), Pd(dmdba)$_2$ (8.15 mg, 0.01 mmol) and 2.0 mL *tert*-butyl methyl ether. After the catalyst/ligand solution was stirred for 30-60 min at 25 ℃. *N*-Boc-*O*-homoallyl-hydroxylamine (0.2 mmol), aryl bromines (0.4 mmol), Cs_2CO_3 (130 mg, 0.4 mmol) were successively added. Under argon atmosphere, the reaction mixture was kept stirring at 75 ℃. for 24 h. After completion of the reaction (monitored by TLC), the mixture was concentrated in vacuum and the residue was purified by flash column chromatography on silica gel with petroleum ether-ethyl acetate as eluent to give the desired product.

4. Optimization of the Reaction Conditions.

Table 1. Screening of Palladium Salts for Reaction[a]

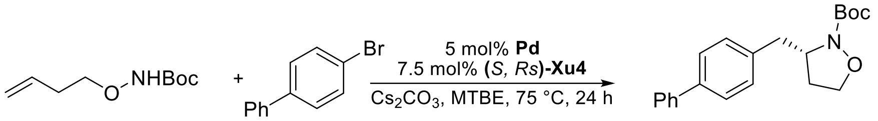

1a **3a**

Entry	Pd	Yield[b](%)	*ee*[c](%)
1	$PdCl_2$	55	85
2	$Pd(OAc)_2$	64	80
3	$Pd(MeCN)_2Cl_2$	24	91
4[d]	$[Pd(allyl)Cl]_2$	70	92
5[d]	$Pd_2(dba)_3$	82	92
6	$Pd(dmdba)_2$	84	92

[a] Reaction conditions: **1a** (0.2 mmol), 4-bromobiphenyl (0.4 mmol), Cs_2CO_3 (2 equiv), 5 mol% **Pd**, and 7.5 mol% ligand in 2.0 mL MTBE, 75 °C under Ar for 24 h. [b] Isolated yield. [c] ***Ee*** was determined by HPLC analysis. [d] 2.5 mol% Pd.

Table 2. Screening of Bases for Reaction[a]

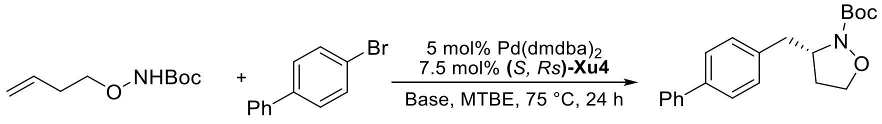

1a **3a**

Entry	Base	Yield[b](%)	*ee*[c](%)
1	NaO^{t}Bu	49	90
2	KO^{t}Bu	38	91
3	Na_2CO_3	-	-
4	K_2CO_3	40	91
5	Et_3N	-	-
6	NaOH	61	92
7	KOH	73	92
8	Cs_2CO_3	84	92

[a] Reaction conditions: **1a** (0.2 mmol), 4-bromobiphenyl (0.4 mmol), Base (2 equiv), 5 mol% $Pd(dmdba)_2$, and 7.5 mol% ligand in 2.0 mL MTBE, 75 °C under Ar for 24 h. [b] Isolated yield. [c] ***ee*** was determined by HPLC analysis.

Table 3. Screening of Solvents for Reaction[a]

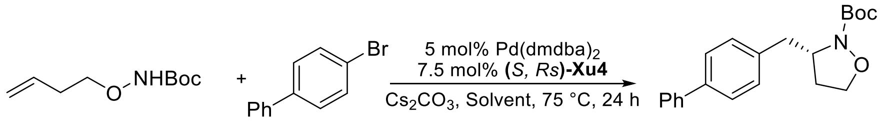

1a **3a**

Entry	Solvent	Yield[b](%)	*ee*[c](%)
1	Tol	59	89
2	THF	78	90
3	Mesitylene	67	91
4	DCM	79	88
5	MTBE	84	92
6	MeCN	63	86
7	EA	80	90
8	DMF	79	89

[a] Reaction conditions: **1a** (0.2 mmol), 4-bromobiphenyl (0.4 mmol), Cs_2CO_3 (2 equiv), 5 mol% $Pd(dmdba)_2$, and 7.5 mol% ligand in 2.0 mL Solvent, 75 ℃ under Ar for 24 h. [b] Isolated yield. [c]***ee*** was determined by HPLC analysis.

Table 4. Screening of reaction temperature[a]

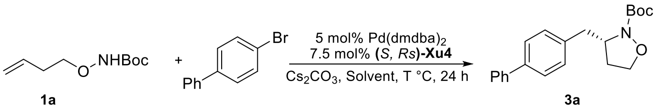

Entry	T(℃)	Yield[b](%)	*ee*[c](%)
1	70	88	92
2	65	87	92.5
3	60	86	93
4	50	89	93
5	40	88	93.5
6	30	91	94

[a] Reaction conditions: **1a** (0.2 mmol), 4-bromobiphenyl (0.4 mmol), Cs_2CO_3 (2 equiv), 5 mol% $Pd(dmdba)_2$, and 7.5 mol% ligand in 2.0 mL MTBE 2mL, T ℃ under Ar for 24 h. [b] Isolated yield. [c]***Ee*** was determined by HPLC analysis.

5. General Procedure for Preparation of Substrates 1a and 2a.[3,4]

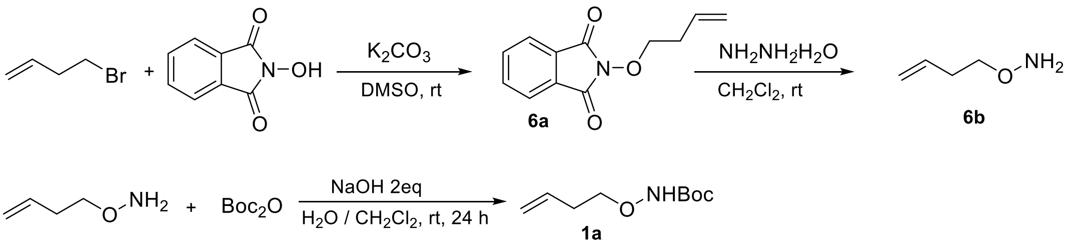

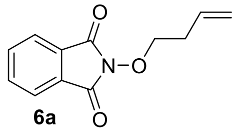

6a

To a solution of K_2CO_3 (10.36 g, 75 mmol) and *N*-hydroxyphthalimide (8.15 g, 50 mmol) in DMSO (150 mL) was added 4-bromobut-1-ene (6.75 g, 50 mmol) slowly. Then this mixture was stirred at room temperature for 24 hours. The solution was diluted with water (150 mL), and extracted with ethyl acetate (100 mL × 3). The combined organic layers were dried over Na_2SO_4, filtered, concentrated and then was purified by flash column chromatography on silica gel (petroleum ether : ethyl acetate = 10:1) to afford the product **6a** as a colorless liquid (6.6 g, 61 % yield). ^{1}H NMR (400 MHz, $CDCl_3$) δ 7.86-7.78 (m, 2H), 7.74-7.72 (m, 2H), 5.95-5.81 (m, 1H), 5.18 (dd, *J* = 17.2 Hz, *J* = 1.2Hz, 1H), 5.10 (dd, *J* = 10.3 Hz, *J* = 1 Hz, 1H), 4.24 (t, *J* = 6.9 Hz, 2H), 2.55 (q, *J* = 6.8 Hz, 2H); ^{13}C NMR (101 MHz, $CDCl_3$) δ 163.50, 134.40, 133.10, 128.91, 123.43, 117.43, 77.23, 32.47.

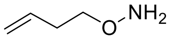

6b

To a solution of the previously synthesized 2-(but-3-en-1-yloxy)isoindoline-1,3-dione **6a** (30 mmol) in DCM (300 ml) were added hydrazine monohydrate (180 mmol). After stirring overnight, the mixture was filtered through celite and concentrated in vacuum to give **6b** as a crude product, which was used for the next step without further purification.

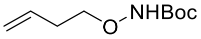

1a

The *O*-(but-3-en-1-yl)hydroxylamine **6b** was dissolved in water/dichloromethane (150 mL, 1:1). Sodium hydroxide (60 mmol, 2eq) was added to the reaction mixture followed by di-tert-butyldicarbonate (30 mmol, 1eq). The resulting reaction mixture was stirred at room temperature for 24 hours. The organic layer was separated, and the aqueous layer was extracted with dichloromethane (3×100 mL). The combined organic layers were dried over Na_2SO_4, filtered, concentrated and then was purified by flash column chromatography on silica gel (petroleum ether : ethyl acetate = 10:1) to afford the product **1a** as a colorless liquid (3.2 g, 67 % yield). ^{1}H NMR (400 MHz, $CDCl_3$) δ 7.37 (d, *J* = 6.0 Hz, 1H), 5.84-5.70 (m, 1H), 5.07 (d, *J* = 17.2 Hz, 1H), 5.00 (d, *J* = 10.2 Hz, 1H), 3.85 (t, *J* = 6.7 Hz, 2H), 2.34 (q, *J* = 6.4 Hz, 2H), 1.43 (s, 9H); ^{13}C NMR (101 MHz, $CDCl_3$) δ 156.84, 134.30, 116.64, 81.41, 75.49, 32.39, 28.08.

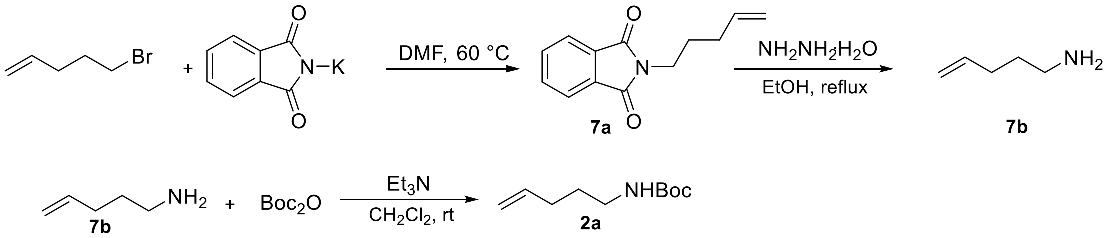

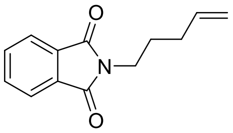

7a

A solution of 5-bromopent-1-ene (14.9 g, 100 mmol) and potassium phthalimide (18.5 g, 100 mmol) in DMF (100 mL) was heated at 60 ℃ overnight. The solution was diluted with water (150 mL), and extracted with diethyl ether (100 mL × 3). The combined organic layers were dried over Na_2SO_4, filtered, concentrated and then was purified by flash column chromatography on silica gel (petroleum ether : ethyl acetate = 10:1) to afford the product **7a** as a colorless liquid (20.3 g, 94 % yield). ^{1}H NMR (400 MHz, $CDCl_3$) δ 7.85-7.78 (m, 2H), 7.72-7.64 (m, 2H), 5.85-5.72 (m, 1H), 5.02 (dd, *J* = 17.1 Hz, *J* = 1.3 Hz, 1H), 4.95 (d, *J* = 10.2 Hz, 1H), 3.67 (t, *J* = 7.3 Hz, 2H), 2.09 (q, *J* = 6.8 Hz, 2H), 1.81-1.72 (m, 2H); ^{13}C NMR (101 MHz, $CDCl_3$) δ 168.28, 137.23, 133.76, 132.12, 123.07, 115.20, 37.49, 30.90, 27.57.

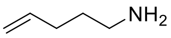

7b

A solution of 2-(pent-4-en-1-yl)isoindoline-1,3-dione **7a** (20.3 g, 94.3 mmol) and hydrazine monohydrate (50 %)(9.4 g, 94.3 mmol) was refluxed in ethanol (350 mL) for 24 h. Concentrated HCl (35 mL) was added dropwise at 0 ℃ and the mixture was refluxed for another 2 h. The mixture was filtered and the filtrate was concentrated in vacuo. The residue was diluted by water (150 mL) and washed by ethyl acetate (40 mL × 2). The aqueous layer was separated and basified with 3 M NaOH to pH 10 at 0 ℃. The solution was extracted with ethyl ether (100 mL × 3). The organic layers were combined, dried over anhydrous Na_2SO_4, filtered and concentrated in vacuo (below 20 ℃) to give **7b** as a yellow oil, which was used for the next step without further purification.

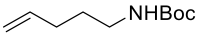

2a

To a stirring solution of di-t-butyldicarbonate (22.2 g, 103 mmol) in CH_2Cl_2 (150 mL) was added amine **7b** (94 mmol) and triethylamine (20.8 g, 206 mmol) in CH_2Cl_2 (150 mL) and the reaction was stirred overnight at room temperature. The reaction was quenched with 0.1 M aqueous citric acid, the mixture was stirred, and the layers were separated. The aqueous layer was extracted with CH_2Cl_2 and the organic layers were combined. The organic layer was washed with saturated aqueous $NaHCO_3$, dried over $MgSO_4$, filtered and concentrated. The crude material was purified by chromatography (petroleum ether : ethyl acetate = 10:1) to afford **2a** as a colorless oil (6.3 g, 36 %). ^{1}H NMR (400 MHz, $CDCl_3$) δ 5.82-5.68 (m, 1H), 4.98 (d, *J* = 17.1 Hz, 1H), 4.92 (d, *J* = 10.2 Hz, 1H), 4.64 (brs, 1H), 3.08 (d, *J* = 6.1 Hz, 2H), 2.03 (q, *J* = 7.1 Hz, 2H), 1.57-1.49 (m, 2H), 1.39 (s, 9H); ^{13}C NMR (101 MHz, $CDCl_3$) δ 155.88, 137.74, 114.92, 78.86, 39.99, 30.86, 29.15, 28.31.

6. Synthesis of (*S*, *Rs*)-Xu4.[5]

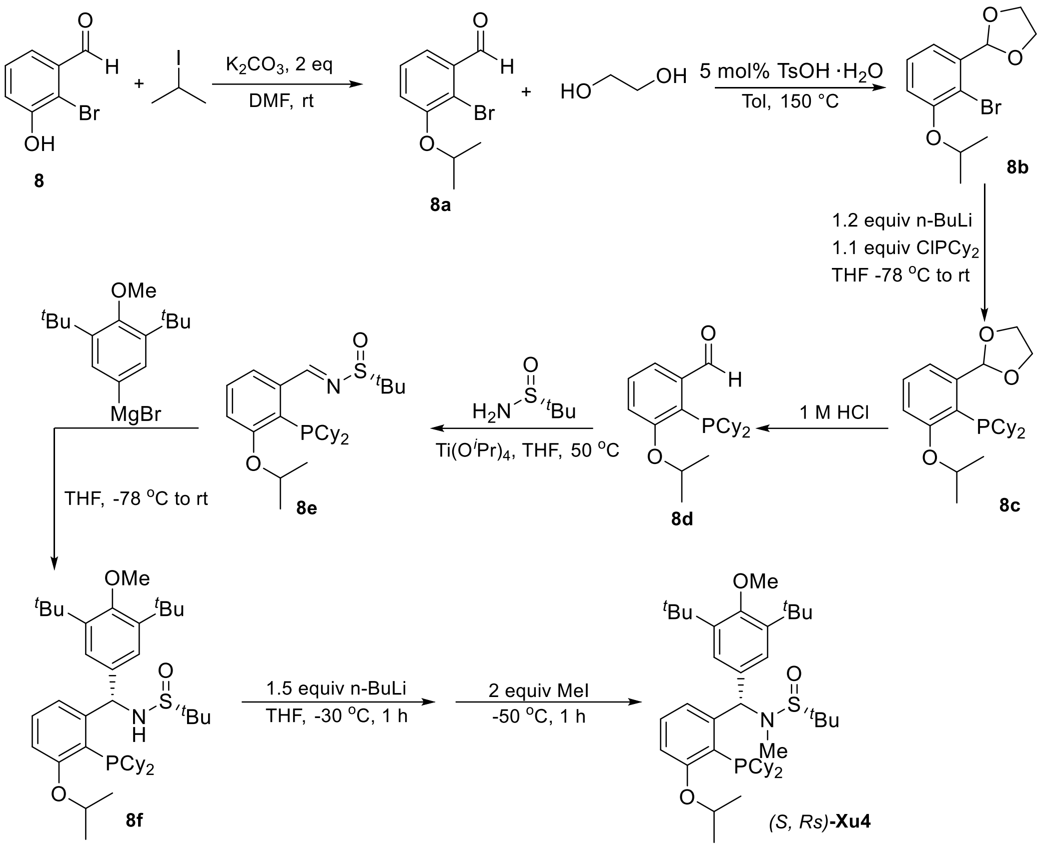

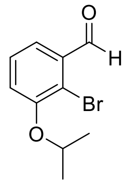

8a

To a solution of 2-bromo-3-hydroxybenzaldehyde **8** (20.1 g, 100 mmol) in 250 mL DMF, was added 2-iodopropane (18.45 g, 150 mmol) and K_2CO_3 (20.73 g, 150 mmol). The resulting solution was stirred at room temperature overnight. The reaction mixture was quenched by the addition of H_2O and diluted with EtOAc. The organic layer was separated, and the aqueous layer was extracted twice with EtOAc. The combined organic layers were dried over Na_2SO_4, filtered, concentrated. The crude product was then was purified by flash column chromatography on silica gel (petroleum ether : ethyl acetate = 30:1) to afford the product **8a** as a colorless liquid. (19.4 g, 80% yield). ^{1}H NMR (500 MHz, $CDCl_3$) δ 10.43 (s, 1H), 7.54-7.47 (m, 1H), 7.34 (t, *J* = 7.9 Hz, 1H), 7.15-7.13 (m, 1H), 4.64-4.56 (m, 1H), 1.42 (dd, *J* = 6.1Hz, *J* = 0.6 Hz, 6H); ^{13}C NMR (126 MHz, $CDCl_3$) δ 192.41, 154.96, 134.98, 128.04, 121.49, 120.48, 118.92, 72.69, 21.91.

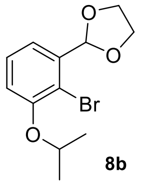

8b

To a solution of 2-bromo-3-isopropoxybenzaldehyde **8a** (90 mmol) in 250 mL toluene, was added ethylene glycol (180 mmol, 2.0 equiv) and *p*-toluenesulfonic acid (5 mol%). The resulting solution was stirred 18 hours at 150 ℃. Upon reaction completion, the reaction mixture was quenched by the addition of H_2O and diluted with EtOAc. The organic layer was separated, and the aqueous layer was extracted twice with EtOAc. The combined organic layers were dried over Na_2SO_4 were dried over Na_2SO_4 and the solvents were removed in vacuo. The residue was purified by silica gel chromatography using petroleum ether/EtOAc as the eluent to afford the desired product **8b** as a white solid. Mp:68.0-68.5℃. ^{1}H NMR (500 MHz, $CDCl_3$) δ 7.25 (t, *J* = 7.9 Hz, 1H), 7.20 (dd, *J* = 7.7 Hz, J = 1.6 Hz, 1H), 6.92 (dd, *J* = 8.0 Hz, *J* = 1.5 Hz, 1H), 6.16 (s, 1H), 4.60-4.50 (m, 1H), 4.18-4.02 (m, 4H), 1.37 (dd, *J* = 6.1 Hz, *J* = 0.4 Hz, 6H); ^{13}C NMR (126 MHz, $CDCl_3$) δ 154.60, 138.35, 127.74, 119.54, 116.28, 114.72, 102.76, 72.34, 65.39, 21.99.

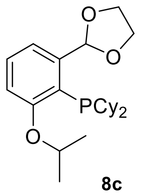

8c

To a solution of 2-(2-bromo-3-isopropoxyphenyl)-1,3-dioxolane **8b** (13.6 g, 47 mmol) in 250 mL dry THF, was added n-BuLi (56 mmol, 2.4 M in hexane) dropwise under argon at -78 ℃, The resulting solution at this temperature during 1 hour, and dicyclohexyl- chlorophosphine (51.7 mmol, 1.1 equiv) was added dropwise. The reaction mixture was warmed to room temperature overnight. The reaction mixture was quenched by the addition of NH_4Cl (aq.) and diluted with EtOAc. The organic layer was separated, and the aqueous layer was extracted twice with EtOAc. The combined organic layers were dried over Na_2SO_4, filtered, concentrated. The crude product was then purified by flash column chromatography on silica gel (petroleum ether : ethyl acetate = 30:1) to afford the product **8c** as a white solid (14 g, 74% yield). Mp: 41.0-42.1°C. ESI-MS calculated for $C_{24}H_{38}O_3P$: m/z (%): 405.2553 (M+ H^+), found: 405.2558. ^{1}H NMR (400 MHz, $CDCl_3$) δ 7.32 (t, *J* = 7.9 Hz, 1H), 7.27-7.21 (m, 1H), 6.83 (d, *J* = 7.9 Hz, 1H), 6.76 (d, *J* = 8.1 Hz, 1H), 4.68-4.59 (m, 1H), 4.17 -3.99 (m, 4H), 2.51-2.40 (m, 2H), 2.00-1.85 (br, 2H), 1.85-1.70 (br, 2H), 1.60 (d, *J* = 7.3 Hz, 4H), 1.38 (d, *J* = 6.0 Hz, 6H), 1.34-1.23 (m, 6H), 1.21 -1.07 (m, 4H), 1.02-0.91 (m, 2H); ^{13}C NMR (101 MHz, $CDCl_3$) δ 159.46 (d, *J* = 3.9 Hz), 146.63 (d, *J* = 21.6 Hz), 130.33, 123.74 (d, *J* = 30.5 Hz), 117.62 (d, *J* = 6.8 Hz), 110.78, 101.63 (d, *J* = 39.5 Hz), 68.38, 65.36, 34.72 (d, *J* = 9.9 Hz), 32.86 (d, *J* = 24.2 Hz), 30.56 (d, *J* = 9.1 Hz), 27.31 (d, *J* = 8.6 Hz), 27.16 (d, *J* = 14.3 Hz), 26.34, 26.33, 21.79; ^{31}P NMR (162 MHz, $CDCl_3$) δ -10.91.

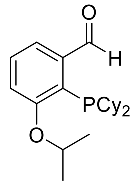

8d

To a solution of (2-(1,3-dioxolan-2-yl)-6-isopropoxyphenyl)dicyclohexylphos-phane **8c** (32 mmol) in 100 mL THF, was added 60 mLHCl (1.0 M) under argon at 60 ℃. The resulting solution was stirred 5 hours. The reaction mixture was quenched by the addition of $NaHCO_3$ (aq.) and diluted with EtOAc. The organic layer was separated, and the aqueous layer was extracted twice with EtOAc. The combined organic layers were dried over Na_2SO_4, filtered, concentrated and then was purified by flash column chromatography on silica gel (petroleum ether : ethyl acetate = 20:1) to afford the product **8d** (5.9 g, 51% yield) as a yellow solid. Mp: 92.0-93.2 °C. ^{1}H NMR (400 MHz, $CDCl_3$) δ 11.25 (d, *J* = 9.0 Hz, 1H), 7.47 (dd, *J* = 7.5 Hz, *J* = 2.7 Hz, 1H), 7.39 (t, *J* = 7.9 Hz, 1H), 6.99 (d, *J* = 8.1 Hz, 1H), 4.73-4.63 (m, 1H), 2.52-2.45 (m, 2H), 1.93 (s, 2H), 1.78 (s, 2H), 1.61 (s, 4H), 1.41 (d, *J* = 6.0 Hz, 6H), 1.34-1.23 (m, 6H), 1.21-1.09 (m, 4H), 1.00 -0.88 (m, 2H); ^{13}C NMR (101 MHz, $CDCl_3$) δ 194.71 (d, *J* = 45.3 Hz), 160.19 (d, *J* = 3.4 Hz), 145.42 (d, *J* = 17.4 Hz), 130.50 (d, *J* = 0.9 Hz), 127.73 (d, *J* = 35.7 Hz), 118.99 (d, *J* = 6.8 Hz), 115.03, 69.07, 34.47 (d, *J* = 10.5 Hz), 32.50 (d, *J* = 23.4 Hz), 30.67 (d, *J* = 8.6 Hz), 27.15 (d, *J* = 8.5 Hz), 26.96 (d, *J* = 14.3 Hz), 26.29 (d, *J* = 0.8 Hz), 21.80; ^{31}P NMR (162 MHz, $CDCl_3$) δ -16.76.

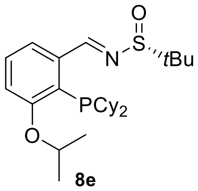

8e

To a solution of 2-(dicyclohexylphosphanyl)-3-isopropoxybenzaldehyde **8d** (5.8 g, 16 mmol) in 50 mL THF, was added (*R*)-2-methylpropane-2-sulfinamide (24 mmol, 1.5 equiv) and titanium tetraisopropanolate (48 mmol, 3 equiv) under argon at 60 ℃. The resulting solution was stirred 8 hours. The reaction mixture was quenched by the addition of H_2O (aq.) and diluted with EtOAc. The solution was filtered and the residue was washed twice with EtOAc. The combined organic layers were dried over Na_2SO_4, filtered, concentrated and then was purified by flash column chromatography on silica gel (petroleum ether : ethyl acetate = 10:1) to afford the product **8e** (4.5 g, 60 yield) as a yellow solid. Mp: 101.0-103.5 °C. ESI-MS calculated for $C_{26}H_{43}NO_2PS$: m/z (%): 464.2747 (M+H$^+$), found: 464.2746. $[a]_D^{22}$ = -157.2 (*c* = 1.0, $CHCl_3$).^{1}H NMR (400 MHz, $CDCl_3$) δ 9.86 (d, *J* = 6.9 Hz, 1H), 7.55 (d, *J* = 5.2 Hz, 1H), 7.35 (t, *J* = 7.7 Hz, 1H), 6.89 (d, *J* = 7.9 Hz, 1H), 4.71-4.61 (m, 1H), 2.55 - 2.33 (brs, 2H), 1.93-1.83 (brs, 2H), 1.81-1.70 (brs, 2H), 1.67-1.51 (brs, 4H), 1.44-1.36 (brs, 6H), 1.36-1.20(brs, 15H), 1.19-1.04 (brs, 4H), 1.02-0.87 (brs, 2H); ^{13}C NMR (101 MHz, $CDCl_3$) δ 164.90 (d, *J* = 41.1 Hz), 160.08 (d, *J* = 3.3 Hz), 143.76 (d, *J* = 21.7 Hz), 130.22, 126.97 (d, *J* = 33.9 Hz), 119.51 (d, *J* = 6.4 Hz), 113.12,

68.77, 57.60, 34.55 (d, *J* = 11.5 Hz), 32.25 (dd, *J* = 23.5, *J* = 3.3 Hz), 30.44 (dd, *J* = 13.1, *J* = 8.8 Hz), 27.12 (dd, *J* = 8.3, *J* = 3.6 Hz), 26.91 (dd, *J* = 14.2, *J* = 6.2 Hz), 22.64, 21.70 (d, *J* = 4.3 Hz). ^{31}P NMR (162 MHz, $CDCl_3$) δ -12.15.

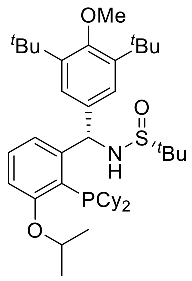

8f

To a solution of (*R*,*E*)-*N*-(2-(dicyclohexylphosphanyl)-3-isopropoxybenzylidene)-2-methylpropane -2-sulfinamide **8e** (3 g, 6 mmol) in 20 mL dry THF, was added (3,5-di-*tert*-butyl-4-methoxyphenyl)magnesium bromide (18 mmol, 3 equiv) dropwise under argon at -78 ℃. The reaction mixture was warmed to room temperature overnight and was quenched by the addition of NH_4Cl (aq.) and diluted with EtOAc. The organic layer was separated, and the aqueous layer was extracted twice with EtOAc. The combined organic layers were dried over Na_2SO_4, filtered, concentrated. The crude product was purified by flash column chromatography on silica gel (petroleum ether : ethyl acetate = 5:1) to afford the product **8f** as a white solid (3.7 g, 84% yield). Mp: 92.0-93.1 °C. ESI-MS calculated for $C_{41}H_{67}NO_3PS$: m/z (%): 684.4574 (M+H$^+$), found: 684.4580. $[a]_D^{16}$ = -81.0 (*c* = 1.0, $CHCl_3$). ^{1}H NMR (400 MHz, $CDCl_3$) δ 7.34 (t, *J* = 7.9 Hz, 1H), 7.28-7.18 (m, 3H), 6.91 (s, 1H), 6.69 (d, *J* = 8.1 Hz, 1H), 4.69-4.55 (m, 1H), 3.83 (s, 1H), 3.65 (s, 3H), 2.48-2.25 (m, 2H), 1.90-1.73 (m, 3H), 1.70-1.60 (m, 3H), 1.50-1.41 (m, 2H), 1.39-1.29 (m, 25H), 1.26-1.18 (m, 12H), 1.16-1.03 (m, 3H), 1.03-0.76 (m, 5H); ^{13}C NMR (101 MHz, $CDCl_3$) δ 159.96 (d, *J* = 3.2 Hz), 158.45, 151.66 (d, *J* = 23.6 Hz), 142.94, 136.96, 129.67, 127.32, 123.14 (d, *J* = 29.9 Hz), 119.89 (d, *J* = 5.5 Hz), 108.84, 68.27, 64.01, 55.71, 35.75, 35.63 (d, *J* = 10.9 Hz), 34.84 (d, *J* = 11.5 Hz), 33.38 (d, *J* = 27.3 Hz), 32.62 (d, *J* = 24.1 Hz), 32.04, 30.31 (d, *J* = 9.7 Hz), 29.82 (d, *J* = 7.2 Hz), 27.40 (dd, *J* = 8.0, *J* = 5.7 Hz), 27.114 (dd, *J* = 4.2 Hz, *J* = 4.2 Hz), 26.86, 26.24 (d, *J* = 9.6 Hz), 22.77, 21.83 (d, *J* = 5.0 Hz); ^{31}P NMR (162 MHz, $CDCl_3$) δ -10.96.

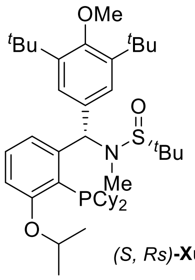

(S, Rs)-**Xu4**

To an oven-dried 100 mL round bottom flask equipped with a stir bar was added the (*R*)-N-((*S*)-(3,5-di-*tert*-butyl-4-methoxyphenyl)(2-(dicyclohexylphosphanyl)-3-isopropoxyphenyl)methyl)-2-methylpropane-2-sulfinamide (3.8 mmol, 1.0 equiv) and 20 mL dry THF under N_2 atmosphere. The

reaction mixture was stirred at -30 ℃ and n-BuLi (5.7 mmol, 1.5 equiv) was then added dropwise to the reaction. After 1.5 h continued stirring, followed by addition of MeI (7.6 mmol, 2.0 equiv) at -50 ℃. After completion, the reaction mixture was quenched by the addition of saturated NH_4Cl solution and diluted with ethyl acetate. The combined organic layers were dried over Na_2SO_4, filtered, concentrated. The crude product was purified by flash column chromatography on silica gel (petroleum ether : ethyl acetate = 5:1) to afford the product (*S*, *Rs*)-**Xu4** as a white solid (2.44 g, 92% yield). Mp: 148.7-149.4 ℃.ESI-MS calculated for $C_{42}H_{69}NO_3PS$: m/z (%): 698.4730 (M+H$^+$), found: 698.4734. $[a]_D^{20}$ = 39.9 (*c* = 1.0, $CHCl_3$). ^{1}H NMR (400 MHz, $CDCl_3$) δ 7.46-7.41 (m, 1H), 7.37 (t, *J* = 7.9 Hz, 1H), 7.00 (s, 2H), 6.93 (d, *J* = 11.6 Hz, 1H), 6.68 (d, *J* = 7.9 Hz, 1H), 4.65-4.55 (m, 1H), 3.60 (s, 3H), 2.61 (s, 3H), 2.51-2.41 (m, 1H), 2.19-2.08 (m, 1H), 1.89-1.71 (m, 3H), 1.67-1.55 (m, 3H), 1.49-1.40 (m, 2H), 1.37-1.29 (m, 24H), 1.28-1.19 (m, 5H), 1.18-1.07 (m, 3H), 1.00 (s, 9H), 0.95-0.62 (m, 4H); ^{13}C NMR (101 MHz, $CDCl_3$) δ 160.12 (d, *J* = 3.6 Hz), 158.42, 150.17 (d, *J* = 22.7 Hz), 142.13, 133.76, 130.47, 130.06, 122.75 (d, *J* = 28.6 Hz), 118.98 (d, *J* = 5.4 Hz), 109.02, 71.26 (d, *J* = 41.1 Hz), 68.14, 64.16, 58.35, 35.55, 35.53 (d, *J* = 10.5), 34.51 (d, *J* = 11.8 Hz), 33.65 (d, *J* = 28.2 Hz), 32.33 (d, *J* = 23.3 Hz), 32.02, 30.69 (d, *J* = 11.2 Hz), 30.57, 29.28 (d, *J* = 6.4 Hz), 27.33 (dd, *J* = 8.0Hz, *J* = 6.0 Hz), 27.12, 26.97 (d, *J* = 3.9 Hz), 26.29 (d, *J* = 29.5 Hz), 23.98 (s), 21.81 (d, *J* = 19.6 Hz); ^{31}P NMR (162 MHz, $CDCl_3$) δ -10.85.

7. Gram-scale synthesis of 3x and synthetic Applications.[6,7]

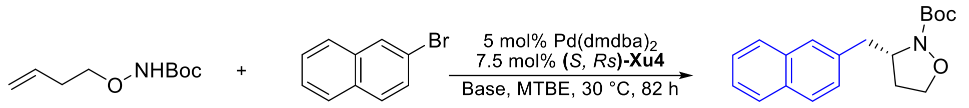

6 mmol 12 mmol **3x**, 1.7 g, 90% yield, 95% ee

A Schlenk tube charged with (*S*, *Rs*)-**Xu4** (314 mg, 0.45 mmol, 7.5 mol%), $Pd(dmdba)_2$ (244 mg, 0.3 mmol, 5 mol%) and 50 mL *tert*-butyl methyl ether. After the catalyst/ligand solution was stirred for 30-60 min at 25 ℃. Then, *N*-Boc-*O*-homoallyl-hydroxylamine (6 mmol), 2-bromonaphthalene (12 mmol) and Cs_2CO_3 (12 mmol) were successively added. Under argon atmosphere, the reaction mixture was kept stirring at 65 ℃ for 82 h. After completion of the reaction (monitored by TLC), the mixture was concentrated in vacuum and the residue was purified by flash column chromatography on silica gel with petroleum ether-ethyl acetate as eluent to give the desired product **3x** as a white solid (1.7 g, 90% yield, 96% ee).

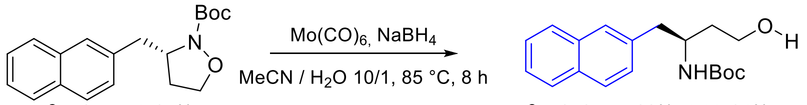

3x, 1 mmol, 95% ee **9a**, 278 mg, 88% yield, 94% ee

To a solution of *tert*-butyl (*S*)-3-(naphthalen-2-ylmethyl)isoxazolidine-2-carboxylate **3x** (313.4 mg,1 mmol) in 5.5 mL MeCN / H_2O (10:1), was added $Mo(CO)_6$ (88 mg, 0.33 mmol) and $NaBH_4$ (60.5 mg. 1.6 mmol). Under argon atmosphere, the reaction mixture was kept stirring at 85 ℃ for 8 h. After completion of the reaction (monitored by TLC), the mixture was concentrated in vacuum and the residue was purified by flash column chromatography on silica gel with petroleum ether-ethyl acetate as eluent to give the desired product **9a** as a white solid (278 mg, 88% yield, 94% *ee*). Mp: 90.7-91.9 °C. ESI-MS calculated for $C_{19}H_{25}NNaO_3$: m/z (%): 338.1727 (M+Na$^+$), found: 338.1723. Enantiomeric excess was determined by HPLC with a chiralpak ADH column (hexanes: 2-propanol = 85:15, 0.5 mL/min, 250 nm); minor enantiomer tr = 14.180 min, major enantiomer tr = 16.684 min. $[a]_D^{16}$ = 25.9 (*c* = 1.0, $CHCl_3$). ^1H NMR (400 MHz, $CDCl_3$) δ 7.77 (t, *J* = 9.1 Hz, 3H), 7.60 (s, 1H), 7.48 -7.38 (m, 2H), 7.31 (d, *J* = 8.3 Hz, 1H), 4.72-4.54 (br, 1H), 4.16 (brs, 1H), 3.62 (brs, 2H), 3.45-3.20 (br, 1H), 3.00-2.84 (br, 2H), 1.96-1.72 (br, 1H), 1.38 (s, 9H); ^{13}C NMR (101 MHz, $CDCl_3$) δ 156.65, 135.33, 133.43, 132.19, 128.00, 127.65, 127.56, 127.52, 127.42, 125.95, 125.39, 79.65, 58.88, 48.17, 41.52, 37.68, 28.22.

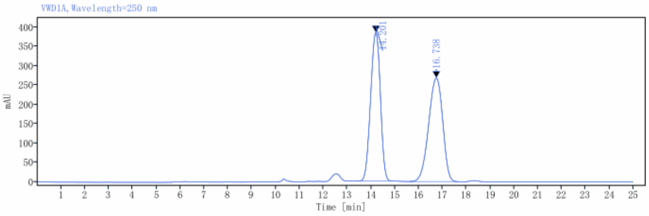

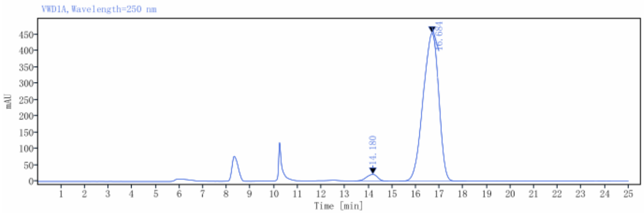

VWD1A, Wavelength=250 nm

Ret. Time [min]	Area	Height	Height%	Area%
14.201	10903.34	383.64	58.92	49.88
16.738	10954.35	267.45	41.08	50.12
Total.	21857.69	651.10	100.00	100.00

VWD1A, Wavelength=250 nm

Ret. Time [min]	Area	Height	Height%	Area%
14.180	649.77	20.08	4.24	3.03
16.684	20801.60	453.39	95.76	96.97
Total.	21451.37	473.47	100.00	100.00

8. Supplementary experiment

8.1 Attempts with other ligands

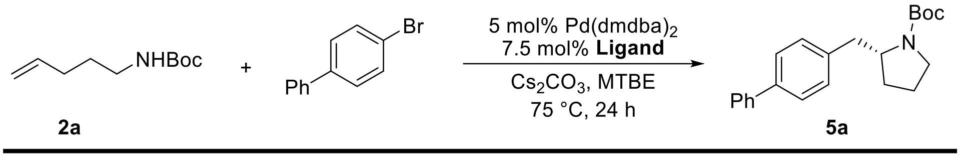

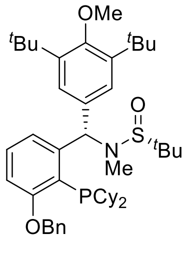

(*S*, *Rs*)-**Xu5**
44% yield, 88% *ee*

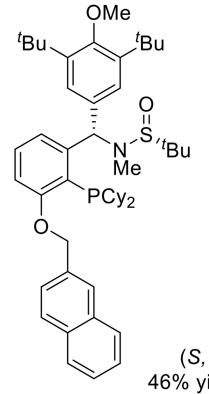

(*S*, *Rs*)-**Xu6**
46% yield, 86% *ee*

[a] Reaction conditions: **2a** (0.2 mmol), 4-bromobiphenyl (0.4 mmol), Cs_2CO_3 (2 equiv.), 5 mol% **Pd**, and 7.5 mol% ligand in 2.0 mL MTBE, 75 ℃ under **Ar** for 24 h. [b] Isolated yield. [c] Ee was determined by HPLC analysis. [d] **2a** (0.1 mmol), 4-bromobiphenyl (0.2 mmol), Cs_2CO_3 (2 equiv.)

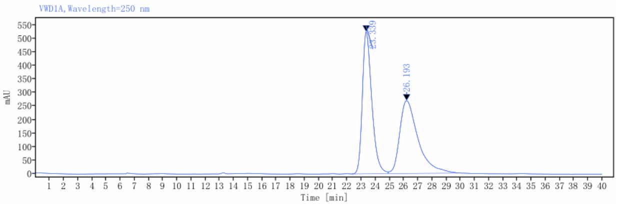

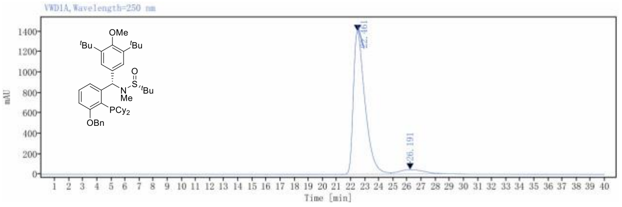

VWD1A, Wavelength=250 nm

Ret. Time [min]	Area	Height	Height%	Area%
23.339	24370.52	526.25	66.10	50.09
26.193	24287.02	269.93	33.90	49.91
Total.	48657.54	796.18	100.00	100.00

VWD1A, Wavelength=250 nm

Ret. Time [min]	Area	Height	Height%	Area%
22.461	80291.55	1402.45	97.04	93.79
26.191	5312.51	42.75	2.96	6.21
Total.	85604.05	1445.21	100.00	100.00

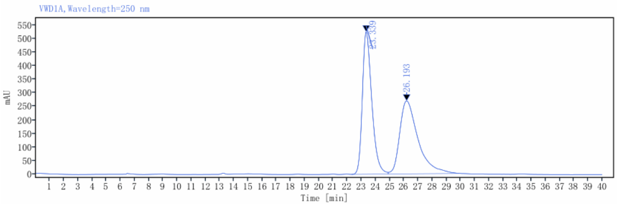

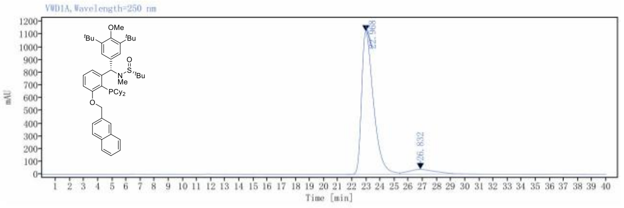

VWD1A, Wavelength=250 nm

Ret. Time [min]	Area	Height	Height%	Area%
23.339	24370.52	526.25	66.10	50.09
26.193	24287.02	269.93	33.90	49.91
Total.	48657.54	796.18	100.00	100.00

VWD1A, Wavelength=250 nm

Ret. Time [min]	Area	Height	Height%	Area%
22.968	65441.46	1120.81	96.81	92.96
26.832	4959.06	36.89	3.19	7.04
Total.	70400.52	1157.69	100.00	100.00

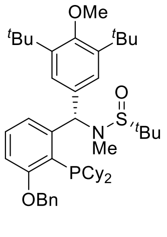

(*S*, *Rs*)-**Xu5**

^{1}H NMR (400 MHz, $CDCl_3$) δ 7.54-7.48 (m, 1H), 7.42-7.32 (m, 6H), 7.00 (s, 2H), 6.92 (d, *J* = 11.5 Hz,

1H), 6.81 (d, J = 8.0 Hz, 1H), 5.07-4.97 (m, 2H), 3.60 (s, 3H), 2.62 (s, 3H), 2.38-2.26 (m, 1H), 2.04-1.94 (m, 1H), 1.72-1.53 (m, 6H), 1.50 (d, J = 12.7 Hz, 1H), 1.32 (s, 22H), 1.23-1.11 (m, 4H), 1.00 (s, 9H), 0.90-0.66 (m, 5H); ^{13}C NMR (101 MHz, $CDCl_3$) δ 161.63 (d, J = 3.2 Hz), 158.48, 150.03 (d, J = 22.2 Hz), 142.21, 136.86, 133.67, 130.48, 130.32, 128.45, 128.04, 119.99 (d, J = 4.9 Hz), 109.46, 77.32, 77.00, 76.68, 71.29 (d, J = 40.1 Hz), 70.37, 64.24, 58.42, 35.60, 35.04 (d, J = 10.6 Hz), 33.90 (d, J = 11.6 Hz), 33.46 (d, J = 27.6 Hz), 32.26, 32.13, 32.06, 30.80 (d, J = 11.7 Hz), 30.66, 29.41 (d, J = 6.1 Hz), 27.29 (d, J = 2.8 Hz), 27.20 (d, J = 4.6 Hz), 27.20 (d, J = 4.6 Hz), 26.84, 26.44, 26.17, 24.01.
^{31}P NMR (162 MHz, $CDCl_3$) δ -10.55.

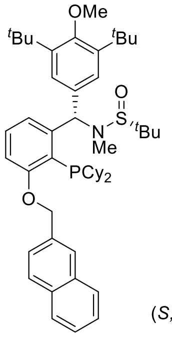

(*S*, *Rs*)-**Xu6**

^{1}H NMR (400 MHz, $CDCl_3$) δ 7.91-7.77 (m, 2H), 7.56-7.47 (m, 1H), 7.41 (t, J = 7.9 Hz, 1H), 7.01 (s, 2H), 6.94 (d, J = 11.5 Hz, 1H), 6.85 (d, J = 8.1 Hz, 1H), 5.24-5.15 (m, 1H), 3.60 (s, 1H), 2.62 (s, 1H), 2.45-2.35 (m, 1H), 2.15-2.00 (m, 1H), 1.68-1.55(m, 5H), 1.51-1.40 (m, 2H), 1.33 (s, 20H), 1.27-1.11 (m, 5H), 1.00 (s, 9H), 0.97-0.82 (m, 4H), 0.81-0.72 (m, 2H); ^{13}C NMR (101 MHz, $CDCl_3$) δ 161.67 (d, J = 3.7 Hz), 158.50, 150.09 (d, J = 22.4 Hz), 142.23, 134.44, 133.68, 133.31, 133.05, 130.52, 130.38, 128.25, 127.78 (d, J = 4.7 Hz), 126.63, 126.29, 126.08, 125.63, 123.21 (d, J = 29.6 Hz), 120.07 (d, J = 5.1 Hz), 109.63, 71.34 (d, J = 40.4 Hz), 70.42, 64.25, 58.43, 35.61, 35.14 (d, J = 10.6 Hz), 33.98 (d, J = 11.7 Hz), 33.47 (d, J = 27.5 Hz), 32.28, 32.08, 30.85 (d, J = 11.4 Hz), 30.68, 29.45 (d, J = 6.7 Hz), 27.31 (dd, J = 8.1, 4.1 Hz), 26.95 (d, J = 4.1 Hz), 26.81, 26.43, 26.15, 24.02; ^{31}P NMR (162 MHz, $CDCl_3$) δ -10.36.

8.2 Synthesis of six-membered ring products

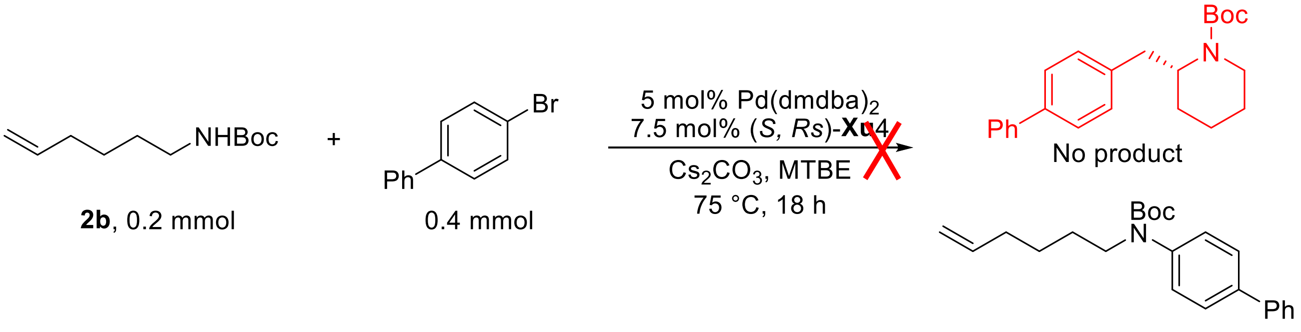

2b, 0.2 mmol

0.4 mmol

B1, 23% yield

We tried to use the substrate *tert*-butyl hex-5-en-1-ylcarbamate to synthesize a six-membered ring product under standard conditions. Unfortunately, under this condition, the raw material remained, and we did not find the corresponding six-membered ring product.

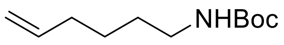

2b

2b(colorless liquid)^{1}H NMR (400 MHz, $CDCl_3$) δ 5.76 (ddt, *J* = 16.9, *J* = 10.1, *J* = 6.7 Hz, 1H), 5.02-4.95 (m, 1H), 4.93 (d, *J* = 10.2 Hz, 1H), 4.55 (s, 1H), 3.09 (d, *J* = 6.0 Hz, 2H), 2.04 (q, *J* = 6.8 Hz, 2H), 1.51-1.33 (m, 13H). ^{13}C NMR (101 MHz, $CDCl_3$) δ 155.94, 138.44, 114.60, 78.94, 77.32, 77.00, 76.68, 40.41, 33.28, 29.48, 28.38, 26.00.

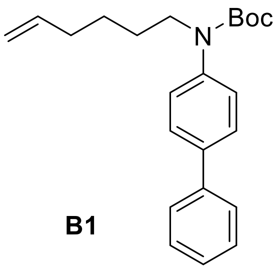

B1

B1, (colorless liquid) ^{1}H NMR (400 MHz, $CDCl_3$) δ 7.56 (dd, *J* = 12.3, *J* = 8.1 Hz, 4H), 7.43 (t, *J* = 7.6 Hz, 2H), 7.33 (t, *J* = 7.3 Hz, 1H), 7.25 (d, *J* = 8.1 Hz, 2H), 5.84-5.72 (m, 1H), 4.96 (dd, *J* = 19.7, *J* =13.8 Hz, 2H), 3.66 (t, *J* = 7.3 Hz, 2H), 2.06 (q, *J* = 6.9 Hz, 2H), 1.64-1.56 (m, 1H), 1.49-1.36 (m, 11H). ^{13}C NMR (101 MHz, $CDCl_3$) δ 154.70, 141.83, 140.55, 138.67, 138.54, 128.72, 127.31, 127.17, 126.97, 114.55, 80.08, 77.32, 77.00, 76.68, 49.80, 33.32, 28.35, 27.99, 26.00.

8.3 Synthesis of Alkyl Chains with Substituted and Quaternary Carbon Center Products

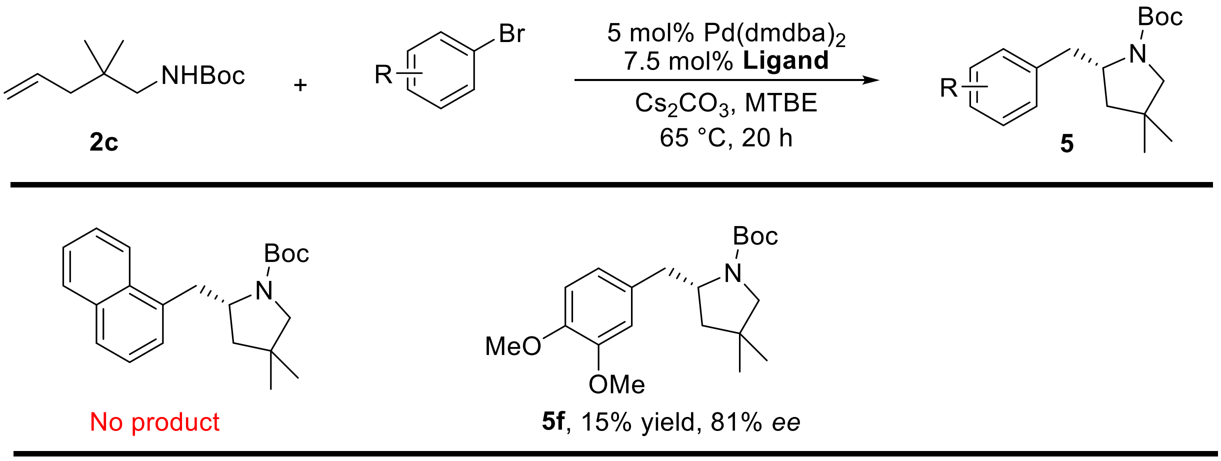

[a] Reaction conditions: **2a** (0.2 mmol), 4-bromobiphenyl (0.4 mmol), Cs_2CO_3 (2 equiv.), 5 mol% **Pd**, and 7.5 mol% ligand in 2.0 mL MTBE, 65 ℃ under **Ar** for 20 h.
[b] Isolated yield. [c] Ee was determined by HPLC analysis.

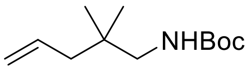

2c

2c(colorless liquid) ^{1}H NMR (400 MHz, $CDCl_3$) δ 5.82 (td, *J* = 17.4, *J* = 7.5 Hz, 1H), 5.07-4.99 (m, 2H), 4.59 (s, 1H), 2.95 (d, *J* = 6.4 Hz, 2H), 1.96 (d, *J* = 7.4 Hz, 2H), 1.44 (s, 9H), 0.87 (s, 6H). ^{13}C NMR (101 MHz, $CDCl_3$) δ 156.16, 134.81, 117.34, 78.93, 77.32, 77.00, 76.68, 50.28, 44.31, 34.71, 28.37, 24.68.

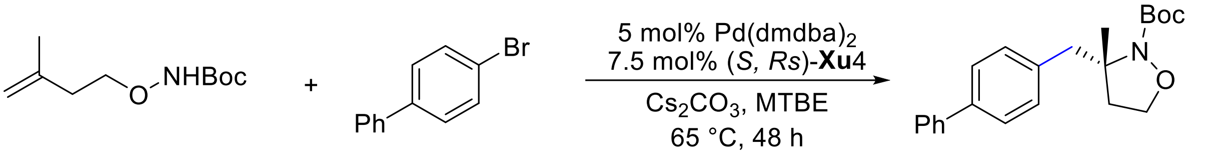

1b **4d**, 27% yield, 67% *ee*

8.4 Reactions of other protection groups

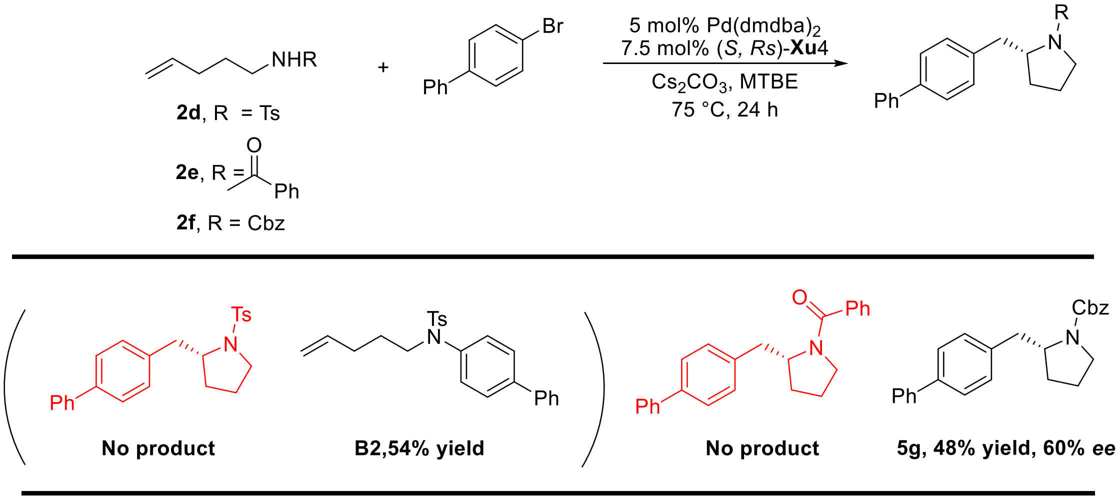

[a] Reaction conditions: **2a** (0.2 mmol), 4-bromobiphenyl (0.4 mmol), Cs_2CO_3 (2 equiv.), 5 mol% **Pd**, and 7.5 mol% ligand in 2.0 mL MTBE, 65 ℃ under **Ar** for 20 h. [b] Isolated yield. [c] Ee was determined by HPLC analysis.

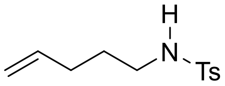

2d

2d (colorless liquid)^{1}H NMR (400 MHz, $CDCl_3$) δ 7.75 (d, *J* = 8.2 Hz, 2H), 7.29 (d, *J* = 8.1 Hz, 2H), 5.76-5.58 (m, 1H), 5.02-4.82 (m, 3H), 2.92 (q, *J* = 6.8 Hz, 2H), 2.41 (s, 3H), 2.02 (q, *J* = 7.0 Hz, 2H), 1.55 (p, *J* = 7.2 Hz, 2H). ^{13}C NMR (101 MHz, $CDCl_3$) δ 143.25, 137.20, 137.02, 129.62, 127.03, 115.42,

77.32, 77.00, 76.68, 42.56, 30.57, 28.63, 21.42.

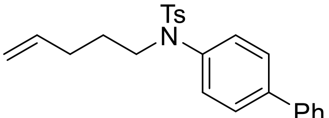

B2

B2, ^{1}H NMR (400 MHz, $CDCl_3$) δ 7.75 (d, *J* = 8.2 Hz, 2H), 7.29 (d, *J* = 8.1 Hz, 2H), 5.76-5.58 (m, 1H), 5.02-4.82 (m, 3H), 2.92 (q, *J* = 6.8 Hz, 2H), 2.41 (s, 3H), 2.02 (q, *J* = 7.0 Hz, 2H), 1.55 (p, *J* = 7.2 Hz, 2H). ^{13}C NMR (101 MHz, $CDCl_3$) δ 143.25, 137.20, 137.02, 129.62, 127.03, 115.42, 77.32, 77.00, 76.68, 42.56, 30.57, 28.63, 21.42.

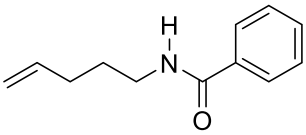

2e

2e (colorless liquid)^{1}H NMR (400 MHz, $CDCl_3$) δ 7.75 (d, *J* = 7.5 Hz, 2H), 7.45 (t, *J* = 7.3 Hz, 1H), 7.37 (t, *J* = 7.5 Hz, 2H), 6.58 (s, 1H), 5.88-5.74 (m, 1H), 5.03 (dd, *J* = 17.2, *J* = 1.0 Hz, 1H), 4.97 (d, *J* = 10.2 Hz, 1H), 3.43 (q, *J* = 6.7 Hz, 2H), 2.12 (q, *J* = 7.1 Hz, 2H), 1.74-1.64 (m, *J* = 7.3 Hz, 2H). ^{13}C NMR (101 MHz, $CDCl_3$) δ 167.51, 137.75, 134.72, 131.18, 128.38, 126.81, 115.12, 77.32, 77.00, 76.68, 39.56, 31.13, 28.67.

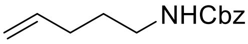

2f

2f (colorless liquid)^{1}H NMR (400 MHz, $CDCl_3$) δ 7.37-7.26 (m, 5H), 5.78 (dq, *J* = 10.0, *J* = 6.6 Hz, 1H), 5.15-4.90 (m, 5H), 3.18 (dd, *J* = 13.1, *J* = 6.5 Hz, 2H), 2.06 (dd, *J* = 13.6, *J* = 6.6 Hz, 2H), 1.64-1.50 (m, 2H). ^{13}C NMR (101 MHz, $CDCl_3$) δ 156.30, 137.58, 136.58, 128.35, 127.91, 115.04, 77.32, 77.00, 76.68, 66.42, 40.43, 30.75, 28.95.

9. Enantioselective synthesis of aryl-substituted isoxazolidines.

9.1. *tert*-Butyl (*S*)-3-([1,1'-biphenyl]-4-ylmethyl)isoxazolidine-2-carboxylate(3a)

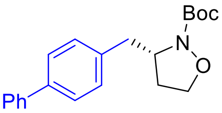

3a

Flash column chromatography on a silicagel (petroleum ether: ethyl acetate = 8: 1) give the product as a white solid (60 mg, 89% yield) with 95% *ee*. Mp: 65.1-66.7 ℃. ESI-MS calculated for $C_{21}H_{25}NNaO_3$: m/z (%): 362.1727 ($M+Na^+$), found: 362.1720. Enantiomeric excess was determined by HPLC with a chiralpak ODH column (hexanes: 2-propanol = 90:10, 0.8 mL/min, 254 nm); major enantiomer tr = 9.973 min, minor enantiomer tr = 11.547 min. $[a]_D^{28}$ = 24.1 (*c* = 1.0, $CHCl_3$). ^{1}H NMR (400 MHz, $CDCl_3$) δ 7.52-7.47 (m, 2H), 7.46-7.42 (m, 2H), 7.37-7.31 (m, 2H), 7.27-7.24 (m, 1H), 7.24-7.20 (m,, 2H), 4.36-4.27 (m, 1H), 3.99 (td, *J* = 8.0 Hz, *J* = 3.7 Hz, 1H), 3.68 (q, 8.4 Hz, 1H), 3.03 (dd, *J* = 13.5, *J* = 6.6 Hz, 1H), 2.67 (dd, *J* = 13.5, *J* = 7.7 Hz, 1H), 2.28-2.17 (m, 1H), 2.02-1.90 (m, 1H), 1.35 (s, 9H); ^{13}C NMR (101 MHz, $CDCl_3$) δ 157.11, 140.91, 139.36, 137.30, 129.74, 128.68, 127.07, 126.93, 68.95, 60.30, 41.22, 33.98, 28.10.

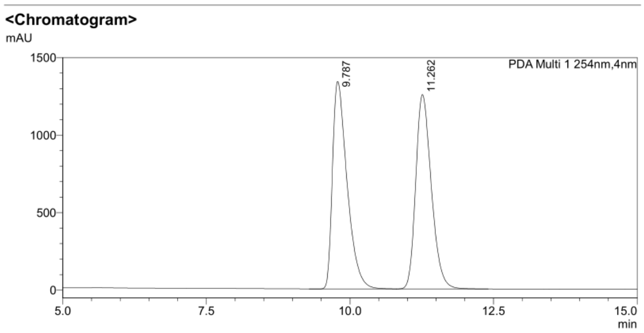

<Peak Table>
PDA Ch1 254nm

Peak#	Ret. Time	Height	Height%	Area	Area%
1	9.787	1338230	51.603	23729570	50.041
2	11.262	1255075	48.397	23691058	49.959
Total		2593305	100.000	47420628	100.000

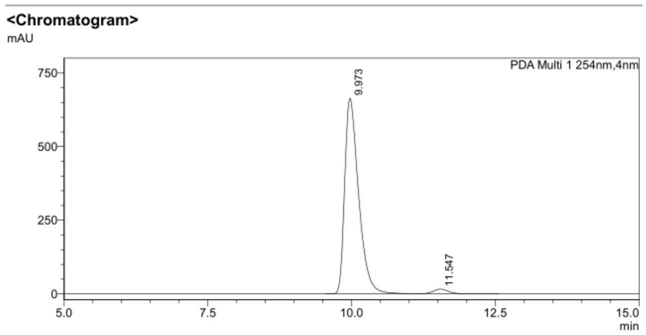

<Peak Table>
PDA Ch1 254nm

Peak#	Ret. Time	Height	Height%	Area	Area%
1	9.973	664123	97.651	11201835	97.319
2	11.547	15975	2.349	308628	2.681
Total		680098	100.000	11510463	100.000

4-Biphenylyl trifluoromethanesulfonate is used instead of p-bromobiphenyl, **3a** can be prepared with 81% yield and 91% *ee*. Enantiomeric excess was determined by HPLC with a chiralpak ODH column (hexanes: 2-propanol = 90:10, 0.8 mL/min, 250 nm); major enantiomer tr = 10.436 min, minor enantiomer tr = 11.859 min.

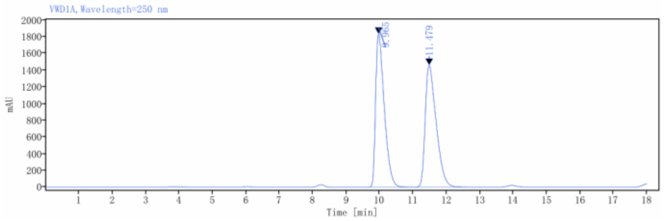

VWD1A, Wavelength=250 nm

Ret. Time [min]	Area	Height	Height%	Area%
9.965	33003.52	1830.01	55.68	49.93
11.479	33097.43	1456.40	44.32	50.07
Total.	66100.95	3286.41	100.00	100.00

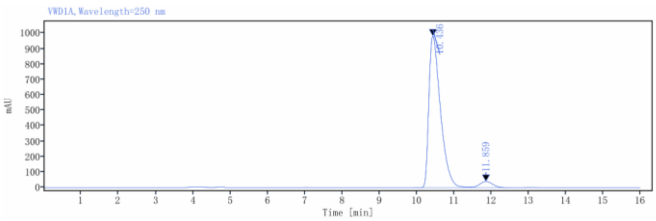

VWD1A, Wavelength=250 nm

Ret. Time [min]	Area	Height	Height%	Area%
10.436	21118.71	981.58	96.11	95.69
11.859	952.20	39.71	3.89	4.31
Total.	22070.90	1021.29	100.00	100.00

9.2. *tert*-Butyl (*S*)-3-benzylisoxazolidine-2-carboxylate(3b).

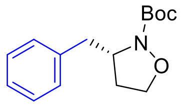

3b

Flash column chromatography on a silicagel (petroleum ether: ethyl acetate = 10: 1) give the product as a white solid (49 mg, 93% yield) with 94% *ee*. Mp: 40.0-41.0 °C. ESI-MS calculated for $C_{15}H_{21}NNaO_3$: m/z (%): 286.1414 ($M+Na^+$), found: 286.1414. Enantiomeric excess was determined by HPLC with a chiralpak ODH column (hexanes: 2-propanol = 90:10, 0.8 mL/min, 210 nm); minor enantiomer tr = 8.311 min, major enantiomer tr = 9.200 min. $[a]_D^{28}$ = 47.9 (*c* = 1.0, $CHCl_3$). ^1H NMR (400 MHz, $CDCl_3$) δ 7.32-7.27 (m, 2H), 7.25-7.19 (m, 3H), 4.41-4.32 (m, 1H), 4.05 (td, *J* = 8.0, *J* = 3.8 Hz, 1H), 3.75 (q, *J* = 8.4 Hz, 1H), 3.08 (dd, *J* = 13.5 Hz, *J* = 6.4 Hz, 1H), 2.70 (dd, *J* = 13.5 Hz, *J* = 8.0 Hz, 1H), 2.32-2.22 (m, 1H), 2.06-1.95 (m, 1H), 1.43 (s, 9H); ^{13}C NMR (101 MHz, $CDCl_3$) δ 157.10, 138.17, 129.32, 128.35, 126.40, 81.63, 68.91, 60.33, 41.57, 33.89, 28.10.

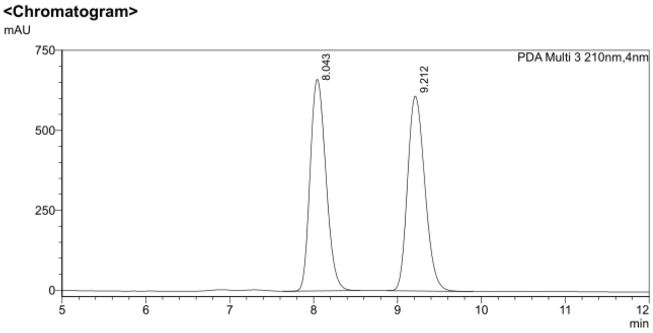

<Peak Table>

PDA Ch3 210nm

Peak#	Ret. Time	Height	Height%	Area	Area%
1	8.043	662609	52.098	8654854	49.711
2	9.212	609244	47.902	8755640	50.289
Total		1271854	100.000	17410494	100.000

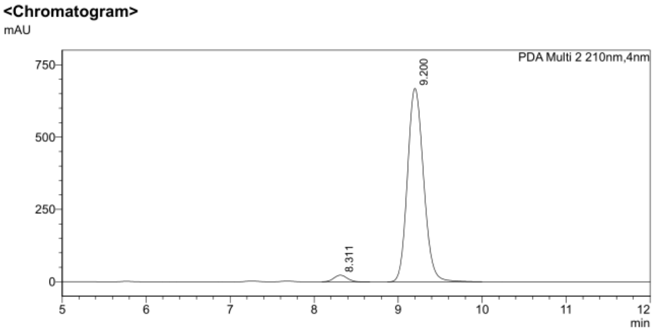

<Peak Table>

PDA Ch2 210nm

Peak#	Ret. Time	Height	Height%	Area	Area%
1	8.311	23103	3.341	257583	2.804
2	9.200	668451	96.659	8927546	97.196
Total		691554	100.000	9185129	100.000

9.3. *tert*-Butyl (*S*)-3-(4-methylbenzyl)isoxazolidine-2-carboxylate(3c).

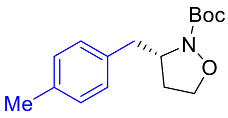

3c

Flash column chromatography on a silicagel (petroleum ether: ethyl acetate = 8: 1) give the product as a colorless liquid (49 mg, 88% yield) with 93% *ee*. ESI-MS calculated for $C_{16}H_{23}NNaO_3$: m/z (%): 300.1570 ($M+Na^+$), found: 300.1566. Enantiomeric excess was determined by HPLC with a chiralpak IC column (hexanes: 2-propanol = 90:10, 0.8 mL/min, 210 nm); major enantiomer tr = 18.113 min, minor enantiomer tr = 20.806 min. $[a]_D^{13}$ = 45.1 (*c* = 1.0, $CHCl_3$). ^1H NMR (400 MHz, $CDCl_3$) δ 7.15-7.06 (m, 4H), 4.38-2.27 (m, 1H), 4.04 (td, *J* = 8.0 Hz, *J* = 3.8 Hz, 1H), 3.74 (q, *J* = 8.3 Hz, 1H), 3.06 (dd, *J* = 13.5 Hz, *J* = 6.0 Hz, 1H), 2.65 (dd, *J* = 13.5 Hz, *J* = 8.3 Hz, 1H), 2.32 (s, 3H), 2.29-2.19

(m, 1H), 2.05-1.94 (m, 1H), 1.45 (s, 9H); ^{13}C NMR (101 MHz, $CDCl_3$) δ 157.09, 135.87, 134.99, 129.15, 129.03, 81.59, 68.89, 60.35, 41.06, 33.76, 28.10, 20.95.

<Chromatogram>

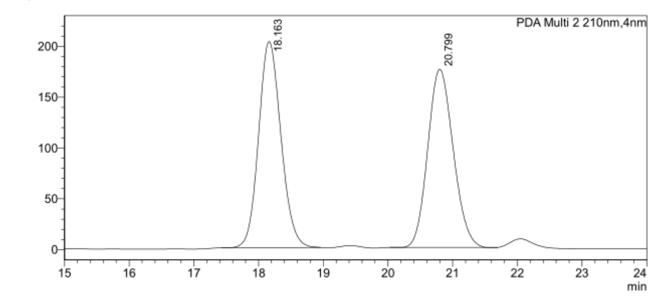

<Peak Table>

PDA Ch2 210nm

Peak#	Ret. Time	Height	Height%	Area	Area%
1	18.163	202739	53.629	4875381	50.382
2	20.799	175300	46.371	4801424	49.618
Total		378039	100.000	9676805	100.000

<Chromatogram>

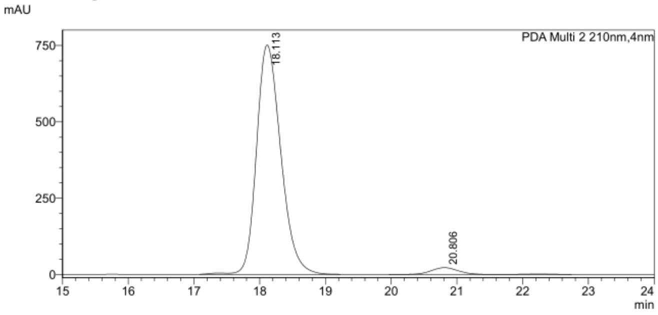

<Peak Table>

PDA Ch2 210nm

Peak#	Ret. Time	Height	Height%	Area	Area%
1	18.113	750736	97.132	19319340	96.671
2	20.806	22164	2.868	665304	3.329
Total		772900	100.000	19984644	100.000

9.4. *tert*-Butyl (*S*)-3-(4-(*tert*-butyl)benzyl)isoxazolidine-2-carboxylate(3d).

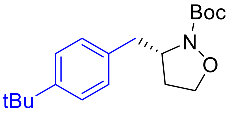

3d

Flash column chromatography on a silicagel (petroleum ether: ethyl acetate = 10: 1) give the product as a colorless liquid (91 mg, 71% yield) with 91% *ee*. ESI-MS calculated for $C_{19}H_{29}NNaO_3$: m/z (%): 342.2040 (M+Na$^+$), found: 342.2046. Enantiomeric excess was determined by HPLC with a chiralpak ADH column (hexanes: 2-propanol = 90:10, 0.8 mL/min, 210 nm); major enantiomer tr = 5.764 min, minor enantiomer tr = 6.840 min. $[a]_D^{29}$ = 30.6 (*c* = 1.0, $CHCl_3$). ^{1}H NMR (400 MHz, $CDCl_3$) δ 7.31 (d, *J* = 8.2 Hz, 2H), 7.16 (d, *J* = 8.1 Hz, 2H), 4.39-4.30 (m, 1H), 4.05 (td, *J* = 8.0 Hz, *J* = 3.8 Hz, 1H), 3.75 (q, *J* = 8.1 Hz, 1H), 3.04 (dd, *J* = 13.5 Hz, *J* = 6.4 Hz, 1H), 2.67 (dd, *J* = 13.5 Hz, *J* = 7.9 Hz, 1H), 2.33-2.23 (m, 1H), 2.07-1.97 (m, 1H), 1.41 (s, 9H), 1.30 (s, 9H); ^{13}C NMR (101 MHz, $CDCl_3$) δ 156.95, 149.10, 135.08, 128.93, 125.18, 81.48, 68.88, 60.32, 41.05, 34.28, 33.94, 31.29, 28.03.

<Chromatogram>

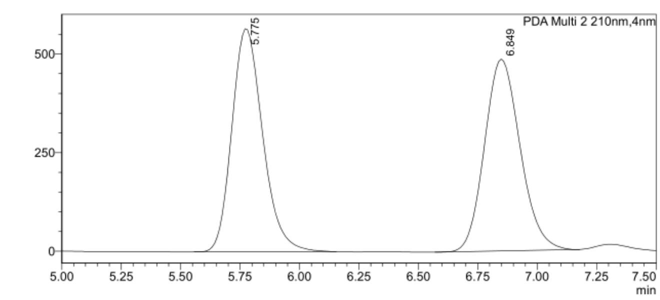

<Peak Table>

PDA Ch2 210nm

Peak#	Ret. Time	Height	Height%	Area	Area%
1	5.775	565503	53.771	5111300	50.199
2	6.849	486181	46.229	5070674	49.801
Total		1051684	100.000	10181974	100.000

<Chromatogram>

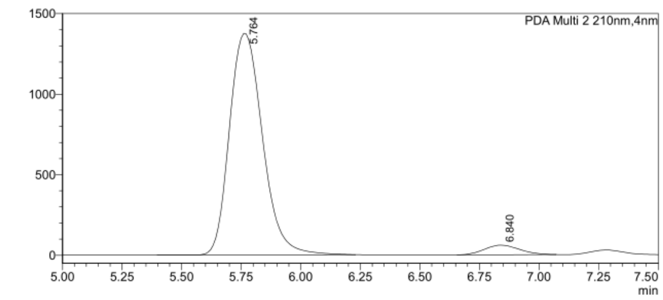

<Peak Table>

PDA Ch2 210nm

Peak#	Ret. Time	Height	Height%	Area	Area%
1	5.764	1375777	95.827	13267560	95.545
2	6.840	59919	4.173	618697	4.455
Total		1435695	100.000	13886257	100.000

9.5. ***tert*-Butyl (*S*)-3-(4-methoxybenzyl)isoxazolidine-2-carboxylate(3e).**

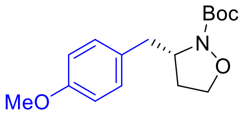

3e

Flash column chromatography on a silicagel (petroleum ether: ethyl acetate = 8: 1) give the product as a colorless liquid (48 mg, 81% yield) with 95% *ee*. ESI-MS calculated for $C_{16}H_{23}NNaO_4$: m/z (%): 316.1519 (M+Na$^+$), found: 316.1516.Enantiomeric excess was determined by HPLC with a chiralpak ADH column (hexanes: 2-propanol = 90:10, 0.8 mL/min, 210 nm); major enantiomer tr = 8.070 min, minor enantiomer tr = 8.910 min. $[a]_D^{26}$ = 39.7 (*c* = 1.0, $CHCl_3$). ^{1}H NMR (400 MHz, $CDCl_3$) δ 7.15-7.10 (m, 2H), 6.84-6.80 (m, 2H), 4.35-4.25 (m, 1H), 4.02 (td, *J* = 8.0 Hz, *J* = 3.8 Hz, 1H), 3.77 (s, 3H), 3.73 (q, *J* = 8.4 Hz, 1H), 3.01 (dd, *J* = 13.6 Hz, *J* = 6.2 Hz, 1H), 2.63 (dd, *J* = 13.6 Hz, *J* = 8.0 Hz, 1H), 2.29-2.19 (m, 1H), 2.04-1.93 (m, 1H), 1.44 (s, 9H); ^{13}C NMR (101 MHz, $CDCl_3$) δ 158.23, 157.11, 130.23, 113.78, 81.55, 68.86, 60.42, 55.16, 40.57, 33.75, 28.10.

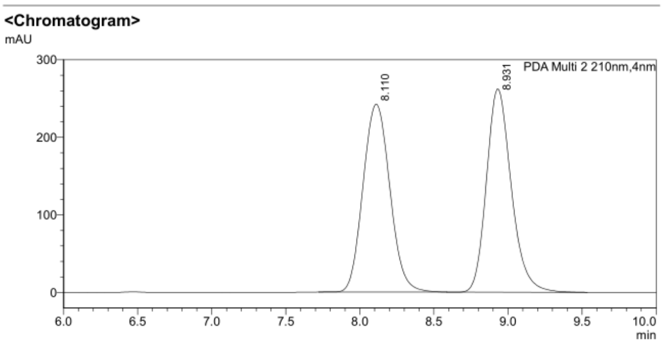

<Peak Table>
PDA Ch2 210nm

Peak#	Ret. Time	Height	Height%	Area	Area%
1	8.110	242364	48.026	3064309	49.967
2	8.931	262292	51.974	3068359	50.033
Total		504656	100.000	6132667	100.000

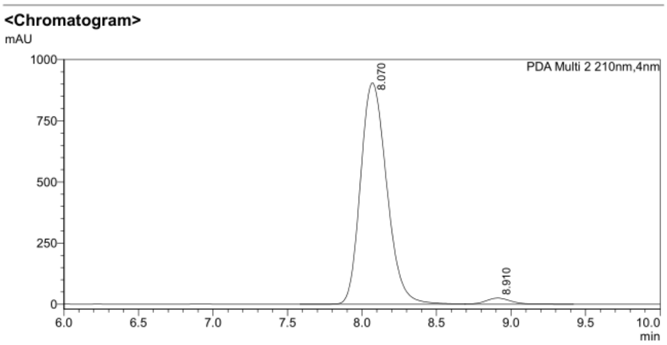

<Peak Table>
PDA Ch2 210nm

Peak#	Ret. Time	Height	Height%	Area	Area%
1	8.070	904566	97.262	11112540	97.325
2	8.910	25462	2.738	305476	2.675
Total		930028	100.000	11418016	100.000

9.6. ***tert*-Butyl (*S*)-3-(4-(dimethylamino)benzyl)isoxazolidine-2-carboxylate(3f).**

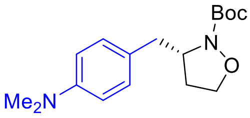

3f

Flash column chromatography on a silicagel (petroleum ether: ethyl acetate = 10: 1) give the product as a yellow liquid (97 mg, 79% yield) with 94% *ee*. ESI-MS calculated for $C_{17}H_{26}N_2NaO_3$: m/z (%): 329.1836 (M+Na$^+$), found: 329.1830. Enantiomeric excess was determined by HPLC with a chiralpak ADH column (hexanes: 2-propanol = 90:10, 0.8 mL/min, 254 nm); major enantiomer tr = 7.518 min, minor enantiomer tr = 9.083 min. $[a]_D^{28}$ = 40.3 (*c* = 1.0, $CHCl_3$). ^{1}H NMR (400 MHz, $CDCl_3$) δ 7.12-7.06 (m, 2H), 6.71-6.66 (m, 2H), 4.34-4.24 (m, 1H), 4.03 (td, *J* = 7.9 Hz, *J* = 4.0 Hz, 1H), 3.74

(q, J = 8.3 Hz, 1H), 3.03 (dd, J = 13.5 Hz, J = 5.6 Hz, 1H), 2.91 (s, 6H), 2.59 (dd, J = 13.6 Hz, J = 8.7 Hz, 1H), 2.27-2.16 (m, 1H), 2.06-1.94 (m, 1H), 1.47 (s, 9H); ^{13}C NMR (101 MHz, $CDCl_3$) δ 157.02, 149.30, 129.82, 125.96, 112.76, 81.43, 68.83, 60.50, 40.66, 40.39, 33.60, 28.09.

<Chromatogram>

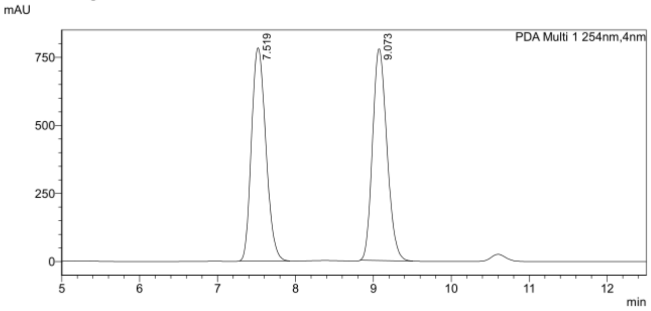

<Peak Table>

PDA Ch1 254nm

Peak#	Ret. Time	Height	Height%	Area	Area%
1	7.519	782781	50.191	10014149	50.077
2	9.073	776814	49.809	9983187	49.923
Total		1559594	100.000	19997336	100.000

<Chromatogram>

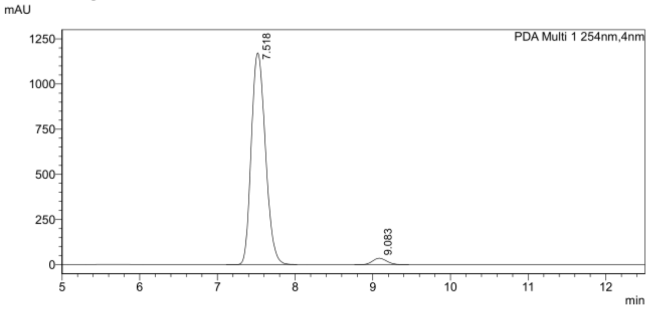

<Peak Table>

PDA Ch1 254nm

Peak#	Ret. Time	Height	Height%	Area	Area%
1	7.518	1172043	97.039	14848734	96.956
2	9.083	35758	2.961	466208	3.044
Total		1207801	100.000	15314942	100.000

9.7. *tert*-Butyl (*S*)-3-(4-fluorobenzyl)isoxazolidine-2-carboxylate(3g).

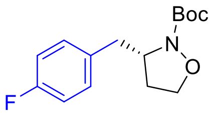

3g

Flash column chromatography on a silicagel (petroleum ether: ethyl acetate = 8: 1) give the product as a colorless liquid (45 mg, 81% yield) with 96% *ee*. ESI-MS calculated for $C_{15}H_{20}FNNaO_3$: m/z (%): 304.1319 (M+Na^+), found: 304.1316. Enantiomeric excess was determined by HPLC with a chiralpak ODH column (hexanes: 2-propanol = 90:10, 0.8 mL/min, 210 nm); major enantiomer tr = 7.331 min, minor enantiomer tr = 8.363 min. $[a]_D^{15}$ = 38.1 (c = 1.0, $CHCl_3$). ^{1}H NMR (400 MHz, $CDCl_3$) δ 7.21-7.14 (m, 2H), 7.00-6.91 (m, 2H), 4.36-4.28 (m, 1H), 4.03 (td, J = 8.1 Hz, J = 3.6 Hz, 1H), 3.73 (q, J = 8.0 Hz, 1H), 2.98 (dd, J = 13.7 Hz, J = 6.9 Hz, 1H), 2.68 (dd, J = 13.7 Hz, J = 7.2 Hz, 1H), 2.34-2.22 (m, 1H), 2.04-1.89 (m, 1H), 1.41 (s, 9H); ^{13}C NMR (101 MHz, $CDCl_3$) δ 161.65 (d, J = 244.3 Hz), 157.17, 133.91 (d, J = 3.3 Hz), 130.73 (d, J = 7.9 Hz), 115.06 (d, J = 21.2 Hz), 81.69, 68.92, 60.31, 40.69, 33.89, 28.06; ^{19}F NMR (376 MHz, $CDCl_3$) δ -116.83.

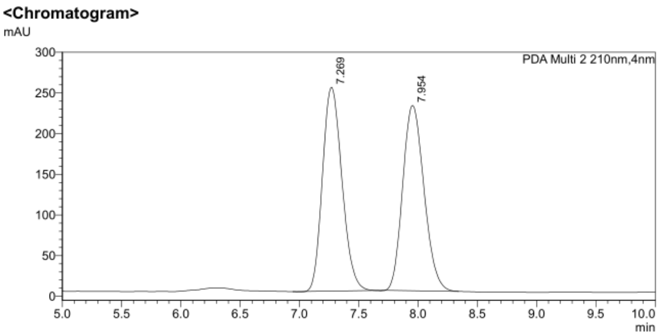

<Peak Table>

PDA Ch2 210nm

Peak#	Ret. Time	Height	Height%	Area	Area%
1	7.269	250477	52.363	2871620	50.117
2	7.954	227869	47.637	2858253	49.883
Total		478346	100.000	5729873	100.000

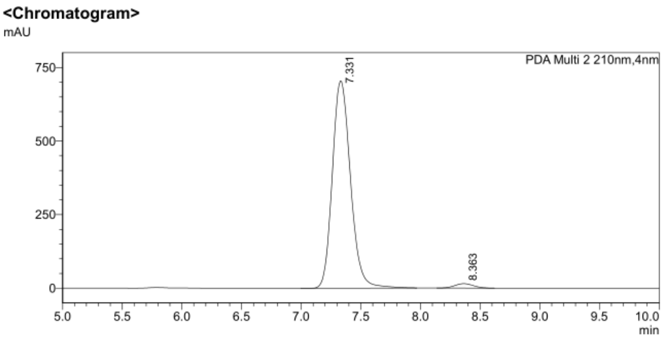

<Peak Table>

PDA Ch2 210nm

Peak#	Ret. Time	Height	Height%	Area	Area%
1	7.331	704841	97.882	7465729	97.811
2	8.363	15252	2.118	167114	2.189
Total		720093	100.000	7632843	100.000

9.8. *tert*-Butyl (*S*)-3-(4-chlorobenzyl)isoxazolidine-2-carboxylate(3h).

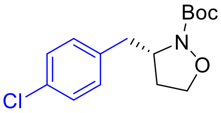

3h

Flash column chromatography on a silicagel (petroleum ether: ethyl acetate = 10: 1) give the product as a white solid (111 mg, 93% yield) with 95% *ee*. Mp: 38.9-40.0 ℃.ESI-MS calculated for $C_{15}H_{20}ClNNaO_3$: m/z (%): 320.1024 ($M+Na^+$), found: 320.1019. Enantiomeric excess was determined by HPLC with a chiralpak ADH column (hexanes: 2-propanol = 90:10, 0.8 mL/min, 210 nm); major enantiomer tr = 6.622 min, minor enantiomer tr = 7.762 min. $[a]_D^{18}$ = 37.6 (*c* = 1.0, $CHCl_3$). ^{1}H NMR (400 MHz, $CDCl_3$) δ 7.23 (d, *J* = 7.8 Hz, 2H), 7.14 (d, *J* = 8.2 Hz, 2H), 4.35-4.26 (m, 1H), 4.02 (td, *J* = 8.1 Hz, *J* = 3.5 Hz, 1H), 3.70 (q, J = 8.4 Hz, 1H), 2.96 (dd, *J* = 13.6 Hz, *J* = 7.0 Hz, 1H), 2.67 (dd, *J* = 13.6 Hz, *J* = 7.1 Hz, 1H), 2.33-2.22 (m, 1H), 2.00-1.89 (m, 1H), 1.40 (s, 9H); ^{13}C NMR (101 MHz, $CDCl_3$) δ 157.11, 136.63, 132.16, 130.63, 128.34, 81.70, 68.90, 60.08, 40.80, 33.88, 27.99.

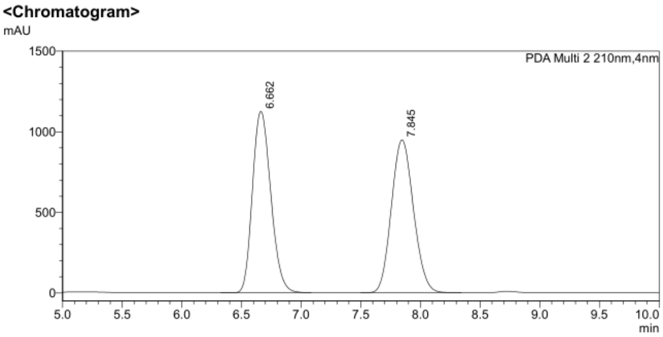

<Peak Table>

PDA Ch2 210nm

Peak#	Ret. Time	Height	Height%	Area	Area%
1	6.662	1124317	54.261	12063713	49.668
2	7.845	947731	45.739	12224810	50.332
Total		2072048	100.000	24288523	100.000

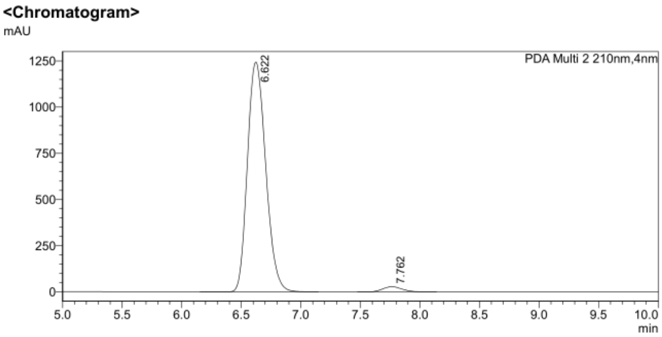

<Peak Table>

PDA Ch2 210nm

Peak#	Ret. Time	Height	Height%	Area	Area%
1	6.622	1244743	97.800	13294803	97.623
2	7.762	28006	2.200	323679	2.377
Total		1272749	100.000	13618482	100.000

9.9. ***tert*-Butyl (*S*)-3-(4-formylbenzyl)isoxazolidine-2-carboxylate(3i).**

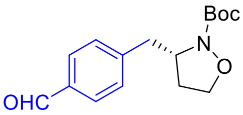

3i

Flash column chromatography on a silicagel (petroleum ether: ethyl acetate = 8: 1) give the product as a white solid (41 mg, 70% yield) with 94% *ee*. Mp: 54.7-55.2 °C. ESI-MS calculated for $C_{16}H_{21}NNaO_4$: m/z (%): 314.1363 ($M+Na^+$), found: 314.1368. Enantiomeric excess was determined by HPLC with a chiralpak ODH column (hexanes: 2-propanol = 80:20, 0.8 mL/min, 254 nm); major enantiomer tr = 9.532 min, minor enantiomer tr = 10.645 min. $[a]_{D30}$ = 25.7 (*c* = 1.0, $CHCl_3$). ^{1}H NMR (400 MHz, $CDCl_3$) δ 9.96 (s, 1H), 7.79 (d, *J* = 8.1 Hz, 2H), 7.39 (d, *J* = 8.1 Hz, 2H), 4.44-4.34 (m, 1H), 4.05 (td, *J* = 8.1 Hz, *J* = 3.5 Hz, 1H), 3.72 (q, 8.4 Hz, 1H), 3.05 (dd, *J* = 13.6 Hz, *J* = 7.4 Hz, 1H), 2.80 (dd, *J* = 13.6 Hz, *J* = 6.7 Hz, 1H), 2.39-2.27 (m, 1H), 2.03-1.92 (m, 1H), 1.37 (s, 9H); ^{13}C NMR (101 MHz, $CDCl_3$) δ 191.87, 157.14, 145.51, 134.86, 130.02, 129.76, 81.85, 68.94, 59.87, 41.67, 34.08, 27.99.

<Chromatogram>

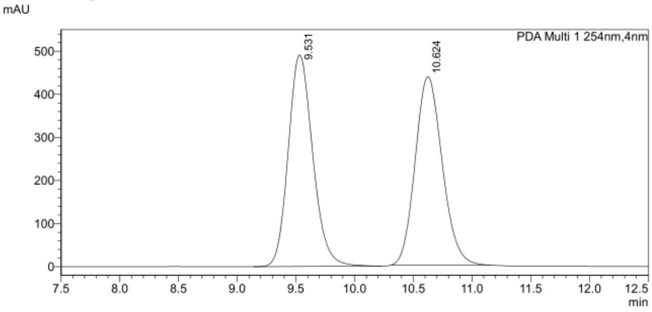

<Peak Table>

PDA Ch1 254nm

Peak#	Ret. Time	Height	Height%	Area	Area%
1	9.531	490938	52.867	7142052	50.453
2	10.624	437696	47.133	7013902	49.547
Total		928634	100.000	14155953	100.000

<Chromatogram>

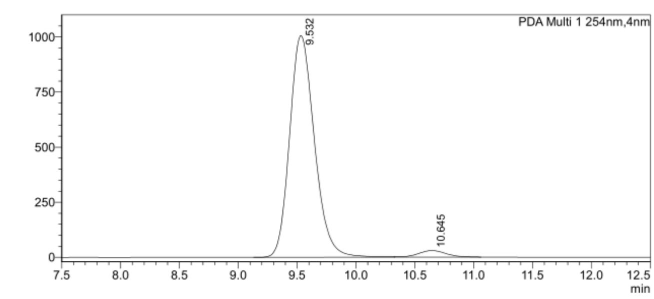

<Peak Table>

PDA Ch1 254nm

Peak#	Ret. Time	Height	Height%	Area	Area%
1	9.532	1006255	97.101	14755786	96.764
2	10.645	30044	2.899	493430	3.236
Total		1036299	100.000	15249217	100.000

9.10. ***tert*-Butyl (*S*)-3-(4-(methoxycarbonyl)benzyl)isoxazolidine-2-carboxylate(3j).**

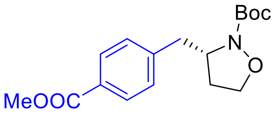

3j

Flash column chromatography on a silicagel (petroleum ether: ethyl acetate = 10: 1) give the product as a white solid (53 mg, 82% yield) with 94% *ee*. Mp: 54.9-56.9 °C.ESI-MS calculated for $C_{17}H_{23}NNaO_5$: m/z (%): 344.1468 ($M+Na^+$), found: 344.1466. Enantiomeric excess was determined by HPLC with a chiralpak ODH column (hexanes: 2-propanol = 90:10, 0.8 mL/min, 254 nm); major enantiomer tr = 11.738 min, minor enantiomer tr = 12.932 min. $[a]_D^{24}$ = 26.9 (*c* = 1.0,

CHCl3). ^{1}H NMR (400 MHz, $CDCl_3$) δ 7.97 (d, *J* = 8.2 Hz, 2H), 7.31 (d, *J* = 8.2 Hz, 2H), 4.44-4.36 (m, 1H), 4.07 (td, *J* = 8.1 Hz, *J* = 3.5 Hz, 1H), 3.90 (s, 3H), 3.74 (q, *J* = 8.3 Hz, 1H), 3.08 (dd, *J* = 13.5 Hz, *J* = 7.1 Hz, 1H), 2.78 (dd, *J* = 13.5 Hz, *J* = 7.1 Hz, 1H), 2.37-2.27 (m, 1H), 2.05-1.95 (m, 1H), 1.41 (s, 9H); ^{13}C NMR (101 MHz, $CDCl_3$) δ 166.90, 157.09, 143.57, 129.57, 129.32, 128.33, 81.75, 68.88, 59.92, 51.89, 41.48, 33.97, 28.00.

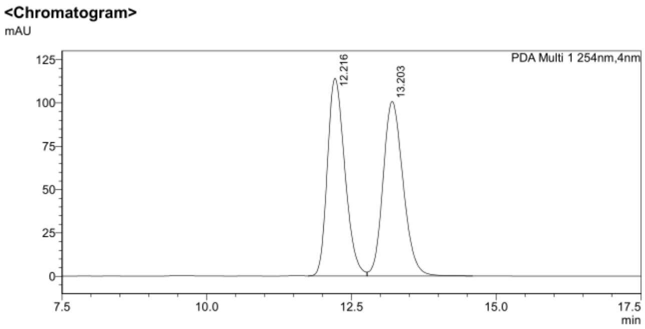

<Peak Table>
PDA Ch1 254nm

Peak#	Ret. Time	Height	Height%	Area	Area%
1	12.216	114044	53.101	2396967	49.650
2	13.203	100724	46.899	2430805	50.350
Total		214768	100.000	4827772	100.000

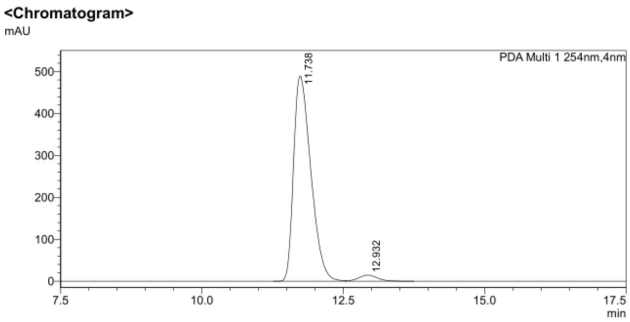

<Peak Table>
PDA Ch1 254nm

Peak#	Ret. Time	Height	Height%	Area	Area%
1	11.738	489573	97.133	10404805	96.856
2	12.932	14450	2.867	337781	3.144
Total		504023	100.000	10742586	100.000

9.11. *tert*-Butyl (*S*)-3-(4-cyanobenzyl)isoxazolidine-2-carboxylate(3k).

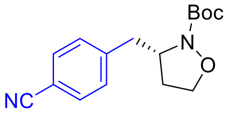

3k

Flash column chromatography on a silicagel (petroleum ether: ethyl acetate = 8: 1) give the product as a white solid (44 mg, 77% yield) with 95% *ee*. Mp: 54.0-56.0 °C..ESI-MS calculated for $C_{16}H_{20}N_2NaO_3$: m/z (%): 311.1366 ($M+Na^+$), found: 311.1365. Enantiomeric excess was determined by HPLC with a chiralpak ODH column (hexanes: 2-propanol = 80:20, 0.8 mL/min, 230 nm); major enantiomer tr = 9.295 min, minor enantiomer tr = 10.698 min. $[a]_D^{30}$ = 23.8 (*c* = 1.0, $CHCl_3$). ^{1}H NMR (400 MHz, $CDCl_3$) δ 7.59 (d, *J* = 8.2 Hz, 2H), 7.36 (d, *J* = 8.2 Hz, 2H), 4.46-4.33 (m, 1H), 4.08 (td, *J* = 8.2 Hz, *J* = 3.4 Hz, 1H), 3.74 (q, *J* = 8.2 Hz, 1H), 3.02 (dd, *J* = 13.7 Hz, *J* = 7.9 Hz, 1H), 2.82 (dd, *J* = 13.7 Hz, *J* = 6.2 Hz, 1H), 2.43-2.33 (m, 1H), 2.04-1.95 (m, 1H), 1.39 (s, 9H); ^{13}C NMR (101 MHz, $CDCl_3$) δ 157.17, 143.88, 131.98, 130.15, 118.82, 110.26, 81.92, 68.94, 59.77, 41.55, 34.10, 27.95.

<Chromatogram>

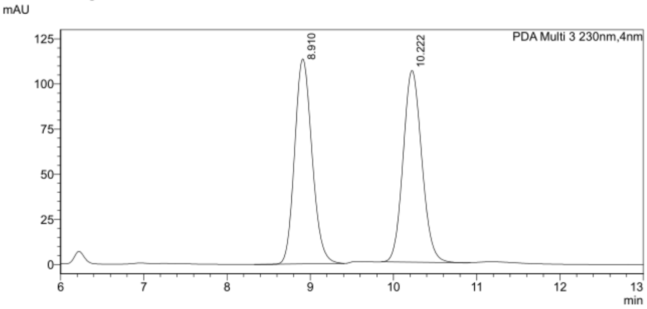

<Peak Table>

PDA Ch3 230nm

Peak#	Ret. Time	Height	Height%	Area	Area%
1	8.910	113422	51.664	1643742	49.825
2	10.222	106117	48.336	1655285	50.175
Total		219539	100.000	3299027	100.000

<Chromatogram>

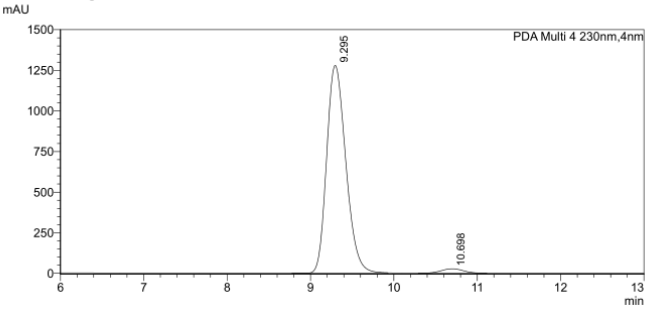

<Peak Table>

PDA Ch4 230nm

Peak#	Ret. Time	Height	Height%	Area	Area%
1	9.295	1278319	97.922	20393337	97.599
2	10.698	27123	2.078	501631	2.401
Total		1305442	100.000	20894968	100.000

9.12. *tert*-Butyl (*S*)-3-(4-(trifluoromethyl)benzyl)isoxazolidine-2-carboxylate(3l).

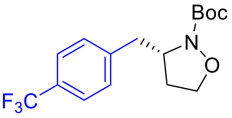

3l

Flash column chromatography on a silicagel (petroleum ether: ethyl acetate = 10: 1) give the product as a white solid (55 mg, 83% yield) with 91% *ee*. Mp: 55.0-56.7 °C..ESI-MS calculated for $C_{16}H_{20}F_3NNaO_3$: m/z (%): 354.1287 (M+Na$^+$), found: 354.1282. Enantiomeric excess was determined by HPLC with a chiralpak ADH column (hexanes: 2-propanol = 90:10, 0.8 mL/min, 210 nm); major enantiomer tr = 6.131 min, minor enantiomer tr = 6.950 min. $[a]_D^{30}$ = 31.9 (*c* = 1.0, $CHCl_3$). ^{1}H NMR (400 MHz, $CDCl_3$) δ 7.53 (d, *J* = 8.1 Hz, 2H), 7.34 (d, *J* = 8.0 Hz, 2H), 4.41 -4.32 (m, 1H), 4.06 (td, *J* = 8.1, *J* = 3.4 Hz, 1H), 3.73 (q, *J* = 8.2 Hz, 1H), 3.04 (dd, *J* = 13.6 Hz, *J* = 7.5 Hz, 1H), 2.78 (dd, *J* = 13.6 Hz, *J* = 6.7 Hz, 1H), 2.38-2.28 (m, 1H), 2.03-1.93 (m, 1H), 1.37 (s, 9H); ^{13}C NMR (101 MHz, $CDCl_3$) δ 157.15, 142.41 (d, *J* = 1.0 Hz), 129.69, 128.77 (q, *J* = 32.4 Hz), 125.18 (q, *J* = 3.8 Hz), 124.237 (q, J = 273.00 Hz), 81.85, 68.96, 60.03, 41.38, 34.10, 27.98; ^{19}F NMR (376 MHz, $CDCl_3$) δ -62.43.

<Chromatogram>

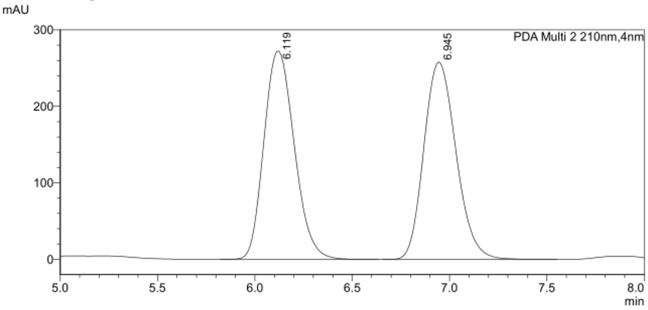

<Peak Table>

PDA Ch2 210nm

Peak#	Ret. Time	Height	Height%	Area	Area%
1	6.119	272695	51.339	2964444	49.892
2	6.945	258474	48.661	2977312	50.108
Total		531169	100.000	5941757	100.000

<Chromatogram>

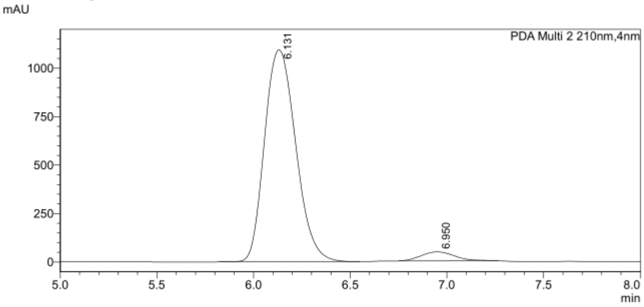

<Peak Table>

PDA Ch2 210nm

Peak#	Ret. Time	Height	Height%	Area	Area%
1	6.131	1094882	95.895	12322286	95.570
2	6.950	46868	4.105	571149	4.430
Total		1141750	100.000	12893435	100.000

9.13. ***tert*-Butyl (*S*)-3-(3-methoxybenzyl)isoxazolidine-2-carboxylate(3m).**

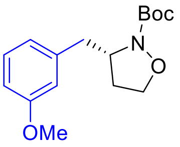

3m

Flash column chromatography on a silicagel (petroleum ether: ethyl acetate = 8:1) give the product as a colorless liquid (45 mg, 77% yield) with 95% *ee*. 55.0-56.7 ℃.ESI-MS calculated for $C_{16}H_{23}NNaO_4$: m/z (%): 316.1519 ($M+Na^+$), found: 316.1516. Enantiomeric excess was determined by HPLC with a chiralpak ODH column (hexanes: 2-propanol = 90:10, 0.8 mL/min, 210 nm); minor enantiomer tr = 9.932 min, major enantiomer tr = 12.657 min. $[a]_D^{27}$ = 39.9 (*c* = 1.0, $CHCl_3$). ^{1}H NMR (400 MHz, $CDCl_3$) δ 7.23-7.17 (m, 1H), 6.81 (d, *J* = 7.6 Hz, 1H), 6.79-6.73 (m, 2H), 4.40-4.32 (m, 1H), 4.05 (td, *J* = 8.0 Hz, *J* = 3.8 Hz, 1H), 3.78 (s, 3H), 3.74 (q, *J* = 8.3 Hz, 1H), 3.07 (dd, *J* = 13.5 Hz, *J* = 6.3 Hz, 1H), 2.66 (dd, *J* = 13.5 Hz, *J* = 8.1 Hz, 1H), 2.32-2.21 (m, 1H), 2.06-1.95 (m, 1H), 1.44 (s, 9H); ^{13}C NMR (101 MHz, $CDCl_3$) δ 159.64, 157.08, 139.74, 129.32, 121.66, 114.98, 111.84, 81.63, 68.89, 60.23, 55.10, 41.59, 33.89, 28.11.

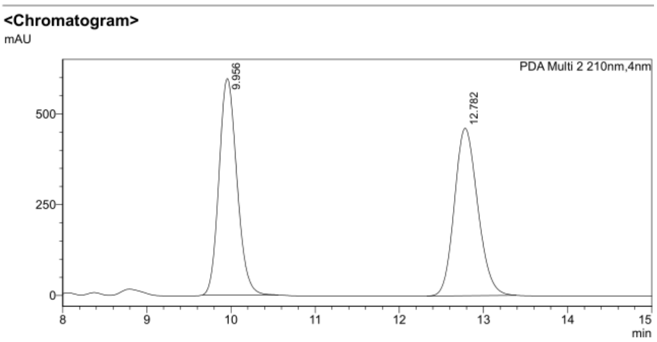

<Peak Table>
PDA Ch2 210nm

Peak#	Ret. Time	Height	Height%	Area	Area%
1	9.956	596079	56.355	8820983	49.731
2	12.782	461647	43.645	8916373	50.269
Total		1057725	100.000	17737356	100.000

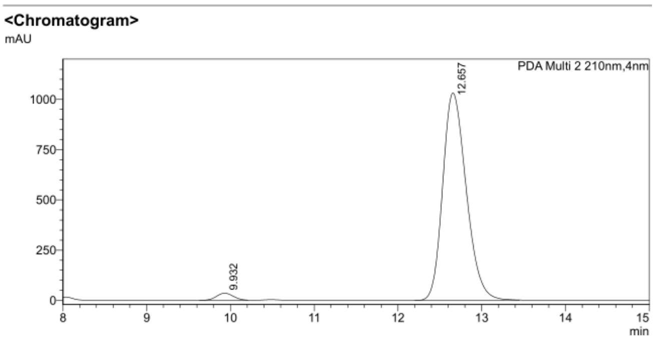

<Peak Table>
PDA Ch2 210nm

Peak#	Ret. Time	Height	Height%	Area	Area%
1	9.932	35153	3.296	491530	2.379
2	12.657	1031306	96.704	20170566	97.621
Total		1066458	100.000	20662095	100.000

9.14. ***tert*-Butyl (*S*)-3-(2-methoxybenzyl)isoxazolidine-2-carboxylate(3n).**

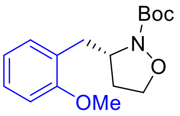

3n

Flash column chromatography on a silicagel (petroleum ether: ethyl acetate = 8:1) give the product as a colorless liquid (24 mg, 40% yield) with 93% *ee*. ESI-MS calculated for $C_{16}H_{23}NNaO_4$: m/z (%): 316.1519 ($M+Na^+$), found: 316.1522. Enantiomeric excess was determined by HPLC with a chiralpak ADH column (hexanes: 2-propanol = 90:10, 0.8 mL/min, 210 nm); minor enantiomer tr = 6.190 min, major enantiomer tr = 8.721 min. $[a]_D^{24}$ = 15.0 (*c* = 1.0, $CHCl_3$). ^{1}H NMR (400 MHz, $CDCl_3$) δ 7.21 (td,

J = 8.0 Hz, J = 1.7 Hz, 1H), 7.16 (dd, J = 7.4 Hz, J = 1.5 Hz, 1H), 6.90-6.82 (m, 2H), 4.52-4.43 (m, 1H), 4.07 (td, J = 8.0 Hz, J = 4.0 Hz, 1H), 3.83 (s, 3H), 3.75 (q, J = 8.1 Hz, 1H), 2.98 (dd, J = 13.2 Hz, J = 6.8 Hz, 1H), 2.79 (dd, J = 13.2 Hz, J = 7.8 Hz, 1H), 2.26-2.17 (m, 1H), 2.07-1.96 (m, 1H), 1.40 (s, 9H); ^{13}C NMR (101 MHz, $CDCl_3$) δ 157.66, 157.09, 131.31, 127.71, 126.73, 120.37, 110.18, 81.30, 68.89, 58.85, 55.19, 36.11, 33.71, 28.09.

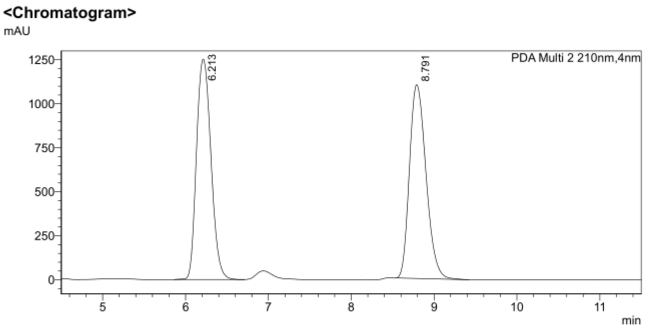

<Peak Table>

PDA Ch2 210nm

Peak#	Ret. Time	Height	Height%	Area	Area%
1	6.213	1252852	53.194	15271878	49.974
2	8.791	1102401	46.806	15287801	50.026
Total		2355253	100.000	30559679	100.000

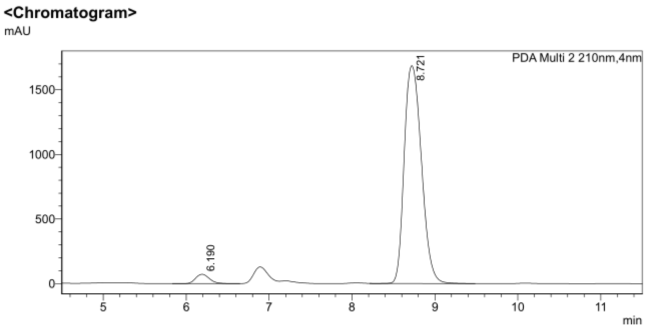

<Peak Table>

PDA Ch2 210nm

Peak#	Ret. Time	Height	Height%	Area	Area%
1	6.190	71920	4.091	824732	3.264
2	8.721	1685903	95.909	24442739	96.736
Total		1757822	100.000	25267472	100.000

9.15. *tert*-Butyl (*S*)-3-(3-fluorobenzyl)isoxazolidine-2-carboxylate(3o).

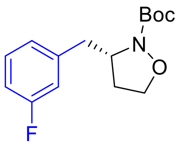

3o

Flash column chromatography on a silicagel (petroleum ether: ethyl acetate = 8:1) give the product as a white solid (42 mg, 75% yield) with 94% *ee*. Mp: 61.3-62.4 ℃.ESI-MS calculated for $C_{15}H_{20}FNNaO_3$: m/z (%): 304.1319 (M+Na$^+$), found: 304.1320. Enantiomeric excess was determined by HPLC with a chiralpak IC column (hexanes: 2-propanol = 90:10, 0.8 mL/min, 210 nm); major enantiomer tr = 15.908 min, minor enantiomer tr = 17.300 min. $[a]_D^{16}$ = 39.6 (*c* = 1.0, $CHCl_3$). ^{1}H NMR (400 MHz, $CDCl_3$) δ 7.28-7.22 (m, 1H), 7.01 (d, J = 7.6 Hz, 1H), 6.98-6.89 (m, 2H), 4.41-4.32 (m, 1H), 4.07 (td, J = 8.0 Hz, J = 3.5 Hz, 1H), 3.75 (q, J = 8.3 Hz, 1H), 3.03 (dd, J = 13.6 Hz, J = 7.0 Hz, 1H), 2.71 (dd, J = 13.6Hz, J = 7.2 Hz, 1H), 2.36-2.27 (m, 1H), 2.04-1.95 (m, 1H), 1.42 (s, 9H); ^{13}C NMR (101 MHz, $CDCl_3$) δ 162.75 (d, J = 245.4 Hz), 157.11, 140.72 (d, J = 7.3 Hz), 129.72 (d, J = 8.3 Hz), 125.00 (d, J = 2.8 Hz), 116.13 (d, J = 21.0 Hz), 113.29 (d, J = 21.0 Hz), 81.79, 68.95, 60.08, 41.28 (d, J = 1.5 Hz), 34.00, 28.04; ^{19}F NMR (376 MHz, $CDCl_3$) δ -113.71.

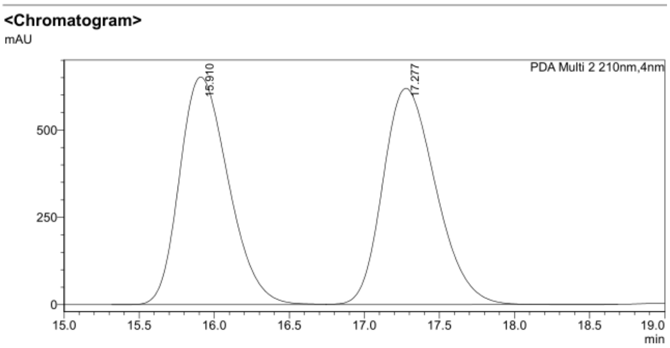

<Peak Table>
PDA Ch2 210nm

Peak#	Ret. Time	Height	Height%	Area	Area%
1	15.910	651575	51.282	14741407	49.245
2	17.277	619007	48.718	15193675	50.755
Total		1270582	100.000	29935082	100.000

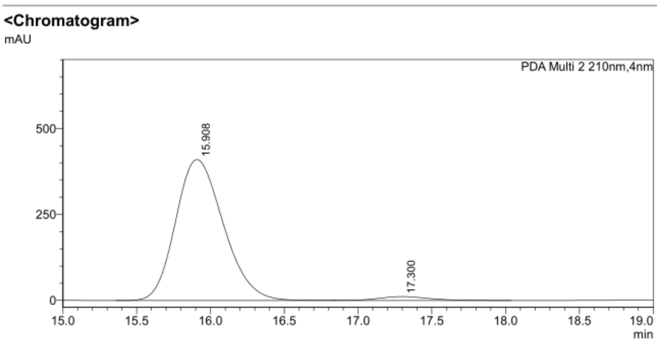

<Peak Table>
PDA Ch2 210nm

Peak#	Ret. Time	Height	Height%	Area	Area%
1	15.908	410154	97.300	9272130	97.118
2	17.300	11383	2.700	275107	2.882
Total		421537	100.000	9547237	100.000

9.16. *tert*-Butyl (*S*)-3-(2-fluorobenzyl)isoxazolidine-2-carboxylate(3p).

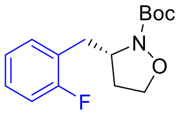

3p

Flash column chromatography on a silicagel (petroleum ether: ethyl acetate = 8:1) give the product as a colorless liquid (26 mg, 45% yield) with 88% *ee*. ESI-MS calculated for $C_{15}H_{20}FNNaO_3$: m/z (%): 304.1319 ($M+Na^+$), found: 304.1317. Enantiomeric excess was determined by HPLC with a chiralpak ODH column (hexanes: 2-propanol = 90:10, 0.8 mL/min, 210 nm); major enantiomer tr = 7.007 min, minor enantiomer tr = 8.369 min. $[a]_D^{18}$ = 30.4 (c = 0.8, $CHCl_3$). ^{1}H NMR (400 MHz, $CDCl_3$) δ 7.28-7.18 (m, 2H), 7.09-6.99 (m, 2H), 4.47-4.38 (m, 1H), 4.08 (td, J = 8.0 Hz, J = 3.6 Hz, 1H), 3.76 (q, J = 8.2 Hz, 1H), 2.98 (dd, J = 13.4 Hz, J = 7.1 Hz, 1H), 2.86 (dd, J = 13.5 Hz, J = 7.2 Hz, 1H), 2.39-2.26 (m, 1H), 2.09-1.96 (m, 1H), 1.40 (s, 9H); ^{13}C NMR (101 MHz, $CDCl_3$) δ 161.35 (d, J = 245.0 Hz), 157.04, 131.93 (d, J = 4.9 Hz), 128.24 (d, J = 8.1 Hz), 125.24 (d, J = 15.8 Hz), 123.96 (d, J = 3.5 Hz), 115.14 (d, J = 22.2 Hz), 81.67, 68.96, 59.20, 34.97, 34.04, 28.06. ^{19}F NMR (376 MHz, $CDCl_3$) δ -118.16.

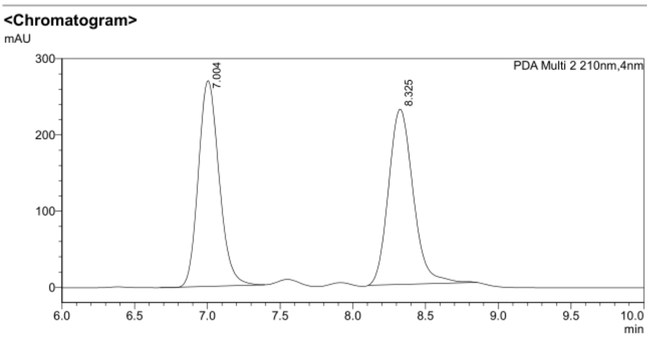

<Peak Table>
PDA Ch2 210nm

Peak#	Ret. Time	Height	Height%	Area	Area%
1	7.004	270113	54.012	2680918	49.608
2	8.325	229983	45.988	2723259	50.392
Total		500097	100.000	5404177	100.000

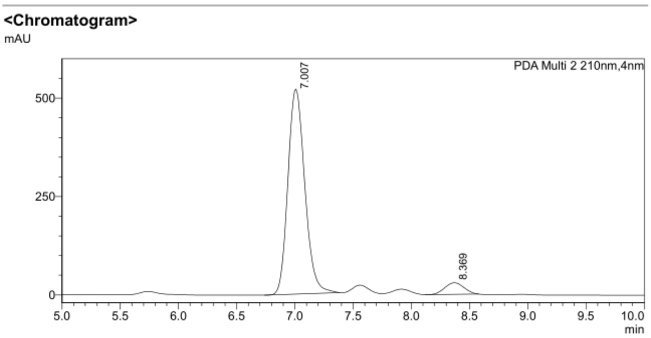

<Peak Table>
PDA Ch2 210nm

Peak#	Ret. Time	Height	Height%	Area	Area%
1	7.007	521185	94.648	5256977	93.927
2	8.369	29469	5.352	339877	6.073
Total		550654	100.000	5596853	100.000

9.17 *tert*-Butyl (*S*)-3-(2,4,5-trifluorobenzyl)isoxazolidine-2-carboxylate(3q)

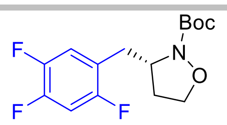

3q

Flash column chromatography on a silicagel (petroleum ether: ethyl acetate = 8:1) give the product as a white solid (81 mg, 64% yield) with 84% ee. Mp: 90.0-92.0 °C. ESI-MS calculated for $C_{15}H_{18}F_3NNaO_3$: m/z (%): 340.1131 (M+Na$^+$), found: 3240.1132. Enantiomeric excess was determined by HPLC with a chiralpak ODH column (hexanes: 2-propanol = 95:5, 0.5mL/min, 210 nm); major enantiomer tr = 13.908 min, minor enantiomer tr = 21.302 min. ^{1}H NMR (400 MHz, $CDCl_3$) δ 7.13-7.03 (m, 1H), 6.91-6.81 (m, 1H), 4.41-4.31 (m, 1H), 4.06 (td, *J* = 8.0, *J* = 2.9 Hz, 1H), 3.71 (q, 8.2 Hz, 1H), 2.80 (d, *J* = 6.9 Hz, 2H), 2.44-2.28 (m, 1H), 2.04-1.90 (m, 1H), 1.38 (s, 9H). ^{13}C NMR (101 MHz, $CDCl_3$) δ 157.28, 157.16, 154.88, 149.98, 147.62, 145.16, 121.74, 121.61, 121.56, 119.31, 119.18, 105.27, 105.07, 104.99, 104.78, 81.89, 68.93, 58.94, 35.52, 34.09, 31.99, 28.12, 27.95, 23.93. ^{19}F NMR (376 MHz, $CDCl_3$) δ -119.69 (dd, *J* = 15.8, 3.4 Hz), -136.23 (d, *J* = 22.2 Hz), -143.47 (dd, *J* = 22.2, 15.5 Hz).

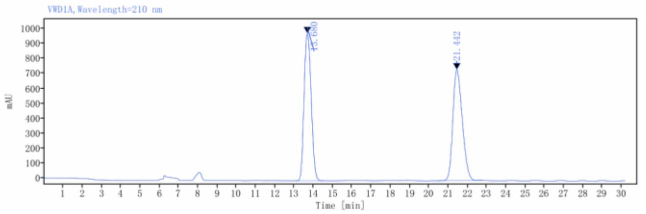

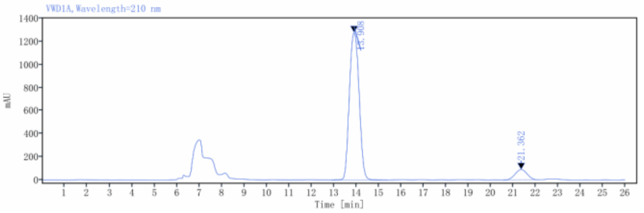

VWD1A, Wavelength=210 nm

Ret. Time [min]	Area	Height	Height%	Area%
13.680	25153.54	984.81	57.07	49.97
21.442	25180.27	740.94	42.93	50.03
Total.	50333.81	1725.75	100.00	100.00

VWD1A, Wavelength=210 nm

Ret. Time [min]	Area	Height	Height%	Area%
13.908	38582.28	1276.30	93.46	92.02
21.362	3345.00	89.28	6.54	7.98
Total.	41927.28	1365.58	100.00	100.00

9.18. *tert*-Butyl (*S*)-3-(3-chlorobenzyl)isoxazolidine-2-carboxylate(3r).

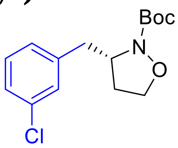

3r

Flash column chromatography on a silicagel (petroleum ether: ethyl acetate = 8:1) give the product as a white solid (50 mg, 83% yield) with 96% *ee*. Mp: 81.0-82.7 °C. ESI-MS calculated for $C_{15}H_{20}ClNNaO_3$: m/z (%): 320.1024 (M+Na$^+$), found: 320.1016. Enantiomeric excess was determined by HPLC with a chiralpak ODH column (hexanes: 2-propanol = 90:10, 0.8 mL/min, 210 nm); major enantiomer tr = 7.887 min, minor enantiomer tr = 8.608 min. $[a]_D^{29}$ = 33.0 (*c* = 1.0, $CHCl_3$). ^{1}H NMR (400 MHz, $CDCl_3$) δ 7.24-7.17 (m, 3H), 7.13-7.06 (m, 1H), 4.38-4.28 (m, 1H), 4.06 (td, *J* = 8.1Hz, *J* = 3.5 Hz, 1H), 3.73 (q, *J* = 8.3 Hz, 1H), 2.98 (dd, *J* = 13.6 Hz, *J* = 7.2 Hz, 1H), 2.68 (dd, *J* = 13.6 Hz, *J* = 7.1 Hz, 1H), 2.36-2.26 (m, 1H), 2.03-1.92 (m, 1H), 1.40 (s, 9H); ^{13}C NMR (101 MHz, $CDCl_3$) δ 157.09, 140.29, 134.03, 129.56, 129.37, 127.54, 126.58, 81.81, 68.96, 60.09, 41.24, 34.06, 28.03.

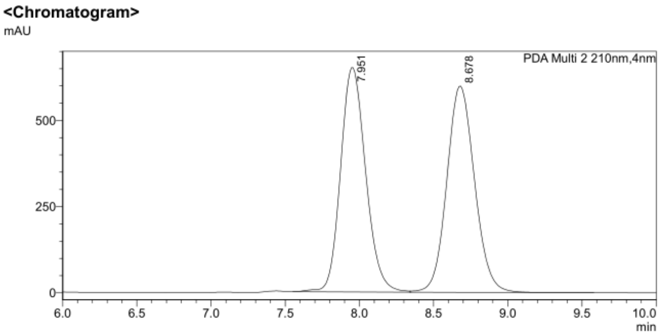

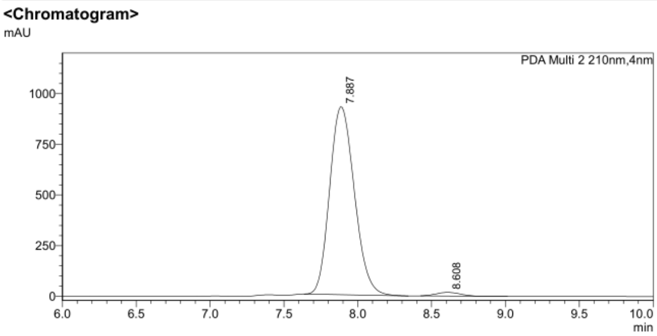

<Peak Table>
PDA Ch2 210nm

Peak#	Ret. Time	Height	Height%	Area	Area%
1	7.951	650532	52.120	7673208	49.670
2	8.678	597609	47.880	7775100	50.330
Total		1248141	100.000	15448308	100.000

<Peak Table>
PDA Ch2 210nm

Peak#	Ret. Time	Height	Height%	Area	Area%
1	7.887	926941	98.105	10805804	98.176
2	8.608	17906	1.895	200731	1.824
Total		944847	100.000	11006535	100.000

9.19. *tert*-Butyl (*S*)-3-(3-formylbenzyl)isoxazolidine-2-carboxylate(3s).

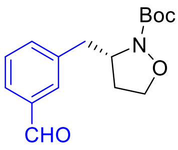

3s

Flash column chromatography on a silicagel (petroleum ether: ethyl acetate = 6:1) give the product as a colorless liquid (48 mg, 83% yield) with 97% *ee*. ESI-MS calculated for $C_{16}H_{21}NNaO_4$: m/z (%): 314.1363 (M+Na$^+$), found: 314.1365. Enantiomeric excess was determined by HPLC with a chiralpak ODH column (hexanes: 2-propanol = 80:20, 0.8 mL/min, 254 nm); major enantiomer tr = 9.378 min, minor enantiomer tr = 11.535 min. $[a]_D^{26}$ = 21.4 (*c* = 1.0, $CHCl_3$). ^{1}H NMR (400 MHz, $CDCl_3$) δ 9.97 (s, 1H), 7.74-7.70 (m, 2H), 7.52-7.47 (m, 1H), 7.46-7.41 (m, 1H), 4.42-4.33 (m, 1H), 4.04 (td, *J* = 8.1 Hz, *J* = 3.5 Hz, 1H), 3.72 (q, *J* = 8.3 Hz, 1H), 3.04 (dd, *J* = 13.7 Hz, *J* = 7.5 Hz, 1H), 2.80 (dd, *J* = 13.7 Hz, *J* = 6.6 Hz, 1H), 2.37-2.28 (m, 1H), 2.04-1.93 (m, 1H), 1.35 (s, 9H); ^{13}C NMR (101 MHz, $CDCl_3$) δ 192.25, 157.14, 139.39, 136.47, 135.59, 130.37, 128.96, 127.97, 81.78, 68.94, 60.05, 41.15, 34.03, 27.96.

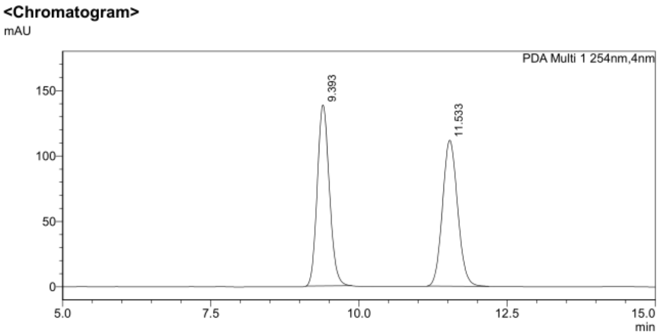

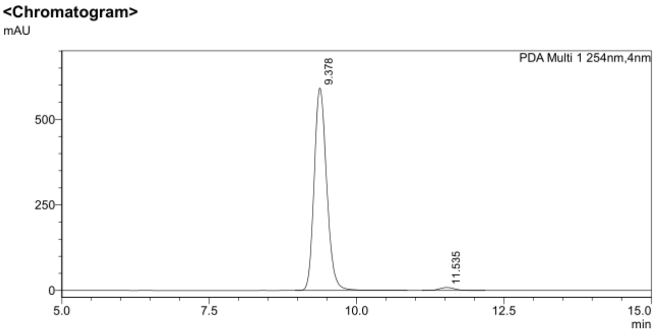

<Peak Table>
PDA Ch1 254nm

Peak#	Ret. Time	Height	Height%	Area	Area%
1	9.393	138452	55.365	1973876	49.832
2	11.533	111620	44.635	1987204	50.168
Total		250073	100.000	3961081	100.000

<Peak Table>
PDA Ch1 254nm

Peak#	Ret. Time	Height	Height%	Area	Area%
1	9.378	591915	98.560	8759321	98.272
2	11.535	8646	1.440	154000	1.728
Total		600560	100.000	8913321	100.000

9.20. ***tert*-Butyl (*S*)-3-(3,4-dimethoxybenzyl)isoxazolidine-2-carboxylate(3t).**

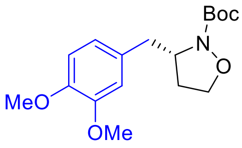

3t

Flash column chromatography on a silicagel (petroleum ether: ethyl acetate = 6:1) give the product as a colorless liquid (103 mg, 80% yield) with 97% *ee*. ESI-MS calculated for $C_{17}H_{25}NNaO_5$: m/z (%): 346.1625 (M+Na$^+$), found: 346.1629. Enantiomeric excess was determined by HPLC with a chiralpak ADH column (hexanes: 2-propanol = 90:10, 0.8 mL/min, 210 nm); minor enantiomer tr = 9.363 min, major enantiomer tr = 10.478 min. $[a]_D^{28}$ = 35.9 (*c* = 1.0, $CHCl_3$). ^{1}H NMR (400 MHz, $CDCl_3$) δ 6.78-6.69 (m, 3H), 4.35-4.25 (m, 1H), 4.01 (td, *J* = 8.0 Hz, *J* = 3.7 Hz, 1H), 3.82 (s, 3H), 3.81 (s, 3H), 3.70 (q, 8.2 Hz, 1H), 2.97 (dd, *J* = 13.6 Hz, *J* = 6.5 Hz, 1H), 2.59 (dd, *J* = 13.6 Hz, *J* = 7.8 Hz, 1H), 2.28-2.19 (m, 1H), 2.01-1.91 (m, 1H), 1.40 (s, 9H); ^{13}C NMR (101 MHz, $CDCl_3$) δ 157.05, 148.65, 147.51, 130.71, 121.13, 112.35, 111.05, 81.45, 68.83, 60.25, 55.72, 55.65, 40.99, 33.73, 27.97.

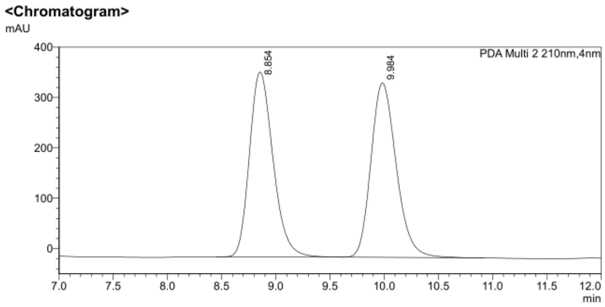

<Peak Table>
PDA Ch2 210nm

Peak#	Ret. Time	Height	Height%	Area	Area%
1	8.854	367414	51.463	5570981	49.801
2	9.984	346526	48.537	5615524	50.199
Total		713941	100.000	11186506	100.000

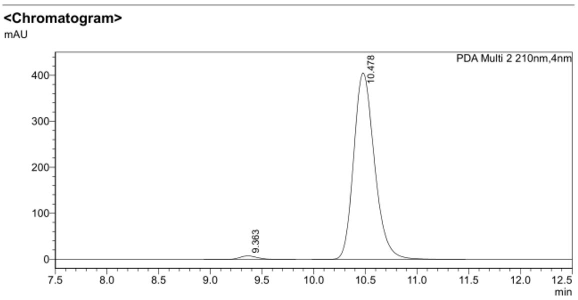

<Peak Table>
PDA Ch2 210nm

Peak#	Ret. Time	Height	Height%	Area	Area%
1	9.363	7754	1.875	95420	1.650
2	10.478	405729	98.125	5686814	98.350
Total		413483	100.000	5782234	100.000

9.21. ***tert*-Butyl (*S*)-3-(3-acetylbenzyl)isoxazolidine-2-carboxylate(3u).**

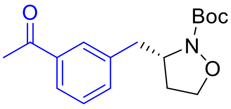

3u

Flash column chromatography on a silicagel (petroleum ether: ethyl acetate = 6:1) give the product as a colorless liquid (102 mg, 84% yield) with 96% *ee*. ESI-MS calculated for $C_{17}H_{23}NNaO_4$: m/z (%): 328.1519 (M+Na$^+$), found: 328.1527. Enantiomeric excess was determined by HPLC with a chiralpak ADH column (hexanes: 2-propanol = 90:10, 0.8 mL/min, 254 nm); minor enantiomer tr = 10.045 min, major enantiomer tr = 11.522 min. $[a]_D^{25}$ = 25.6 (*c* = 1.0, $CHCl_3$). ^{1}H NMR (400 MHz, $CDCl_3$) δ 7.79-7.74 (m, 2H), 7.44-7.38 (m, 1H), 7.37-7.30 (m, 1H), 4.40-4.31 (m, 1H), 4.02 (td, *J* = 8.1 Hz, *J* = 3.5 Hz,

1H), 3.69 (q, J = 8.3 Hz, 1H), 3.02 (dd, J = 13.6 Hz, J = 7.3 Hz, 1H), 2.75 (dd, J = 13.6 Hz, J = 6.9 Hz, 1H), 2.54 (s, 3H), 2.34-2.23 (m, 1H), 2.03-1.89 (m, 1H), 1.34 (s, 9H); ^{13}C NMR (101 MHz, $CDCl_3$) δ 198.00, 157.06, 138.73, 137.07, 134.09, 128.93, 128.46, 126.43, 81.62, 68.87, 60.02, 41.23, 33.92, 27.89, 26.52.

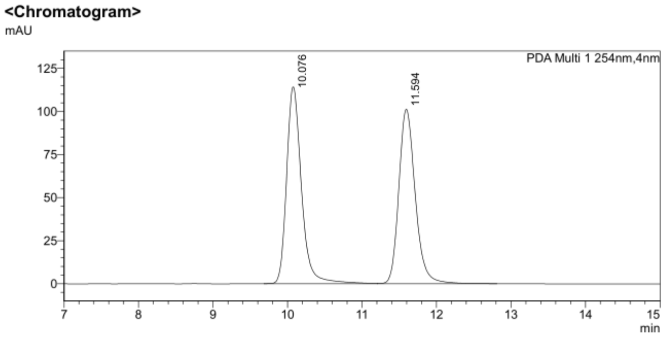

<Peak Table>
PDA Ch1 254nm

Peak#	Ret. Time	Height	Height%	Area	Area%
1	10.076	114305	53.008	1591163	50.620
2	11.594	101333	46.992	1552167	49.380
Total		215638	100.000	3143330	100.000

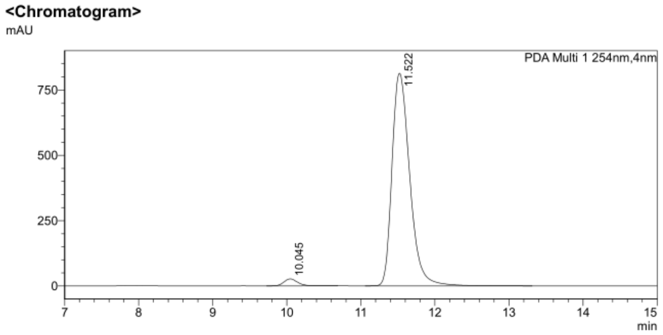

<Peak Table>
PDA Ch1 254nm

Peak#	Ret. Time	Height	Height%	Area	Area%
1	10.045	26834	3.189	353658	2.545
2	11.522	814661	96.811	13541614	97.455
Total		841495	100.000	13895272	100.000

9.22. *tert*-Butyl (*S*)-3-((8-oxo-5,6,7,8-tetrahydronaphthalen-2-yl)methyl) isoxazolidine-2-carboxylate(3v).

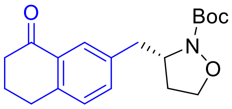

3v

Flash column chromatography on a silicagel (petroleum ether: ethyl acetate = 6:1) give the product as a white solid (120 mg, 91% yield) with 93% *ee*. Mp: 68.4-69.6 °C. ESI-MS calculated for $C_{19}H_{25}NNaO_4$: m/z (%): 354.1676 (M+Na$^+$), found: 354.1682. Enantiomeric excess was determined by HPLC with a chiralpak ADH column (hexanes: 2-propanol = 90:10, 0.8 mL/min, 254 nm); major enantiomer tr = 15.919 min, minor enantiomer tr = 17.456 min. $[a]_D^{15}$ = 11.6 (c = 1.0, $CHCl_3$). ^{1}H NMR (300 MHz, $CDCl_3$) δ 7.86 (s, 1H), 7.35 (d, J = 8.0 Hz, 1H), 7.17 (d, J = 7.9 Hz, 1H), 4.41-4.29 (m, 1H), 4.03 (td, J = 7.9 Hz, J = 3.6 Hz, 1H), 3.72 (q, J = 8.1 Hz, 1H), 3.03 (dd, J = 13.5 Hz, J = 6.5 Hz, 1H), 2.92 (t, J = 6.0 Hz, 2H), 2.73 (dd, J = 13.6 Hz, J = 7.6 Hz, 1H), 2.62 (t, J = 8.4 Hz, 2H), 2.34-2.20 (m, 1H), 2.16-2.05 (m, 2H), 2.04-1.92 (m, 1H), 1.40 (s, 9H); ^{13}C NMR (101 MHz, $CDCl_3$) δ 198.15, 156.94, 142.52, 136.51, 134.49, 132.26, 128.74, 127.34, 81.50, 68.74, 59.95, 40.76, 38.93, 33.70, 29.11, 27.88, 23.09.

<Chromatogram>

mAU

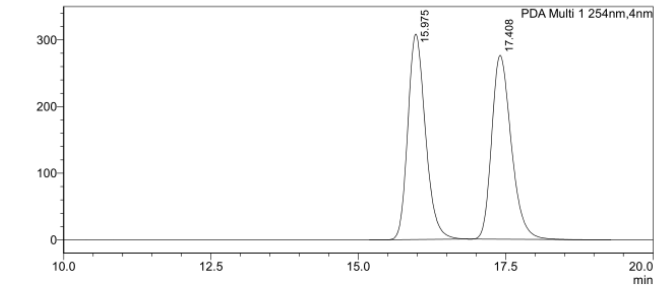

<Peak Table>

PDA Ch1 254nm

Peak#	Ret. Time	Height	Height%	Area	Area%
1	15.975	308465	52.754	6427053	50.097
2	17.408	276254	47.246	6402140	49.903
Total		584719	100.000	12829193	100.000

<Chromatogram>

mAU

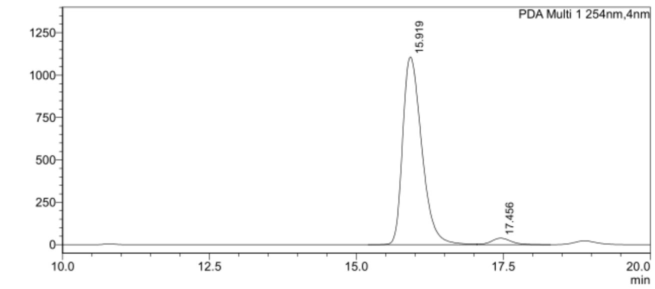

<Peak Table>

PDA Ch1 254nm

Peak#	Ret. Time	Height	Height%	Area	Area%
1	15.919	1106782	96.663	25041393	96.570
2	17.456	38207	3.337	889424	3.430
Total		1144989	100.000	25930817	100.000

9.23. *tert*-Butyl (*S*)-3-((2,3-dihydrobenzo[b][1,4]dioxin-6-yl)methyl) isoxazolidine-2-carboxylate(3w).

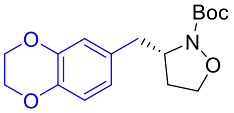

3w

Flash column chromatography on a silicagel (petroleum ether: ethyl acetate = 6:1) give the product as a colorless liquid (100 mg, 78% yield) with 93% *ee*. ESI-MS calculated for $C_{17}H_{23}NNaO_5$: m/z (%): 344.1468 (M+Na$^+$), found: 344.1466. Enantiomeric excess was determined by HPLC with a chiralpak ADH column (hexanes: 2-propanol = 90:10, 0.8 mL/min, 230 nm); minor enantiomer tr = 12.123 min, major enantiomer tr = 13.292 min. $[a]_D^{26}$ = 38.4 (*c* = 1.0, $CHCl_3$). ^{1}H NMR (400 MHz, $CDCl_3$) δ 6.76 (d, *J* = 8.2 Hz, 1H), 6.71 (d, *J* = 1.8 Hz, 1H), 6.66 (dd, *J* = 8.2 Hz, *J* = 1.7 Hz, 1H), 4.32-4.23 (m, 1H), 4.21 (s, 4H), 4.02 (td, *J* = 8.0 Hz, *J* = 3.8 Hz, 1H), 3.72 (q, *J* = 8.0 Hz, 1H), 2.96 (dd, *J* = 13.6 Hz, *J* = 6.1 Hz, 1H), 2.55 (dd, *J* = 13.6 Hz, *J* = 8.2 Hz, 1H), 2.29-2.18 (m, 1H), 2.03-.92 (m, 1H), 1.44 (s, 9H); ^{13}C NMR (101 MHz, $CDCl_3$) δ 157.00, 143.20, 142.08, 131.31, 122.14, 117.86, 116.97, 81.54, 68.84, 64.24, 64.20, 60.31, 40.69, 33.73, 28.06.

<Chromatogram>

mAU

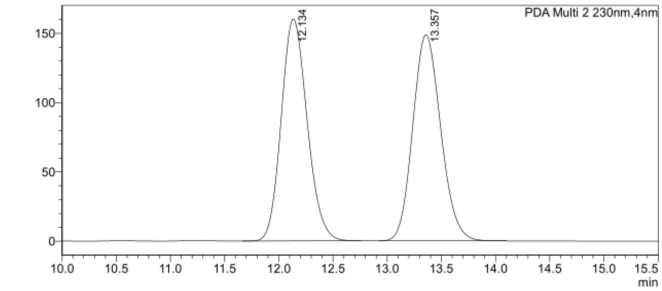

<Peak Table>

PDA Ch2 230nm

Peak#	Ret. Time	Height	Height%	Area	Area%
1	12.134	160025	51.835	2673510	49.986
2	13.357	148693	48.165	2674980	50.014
Total		308718	100.000	5348490	100.000

<Chromatogram>

mAU

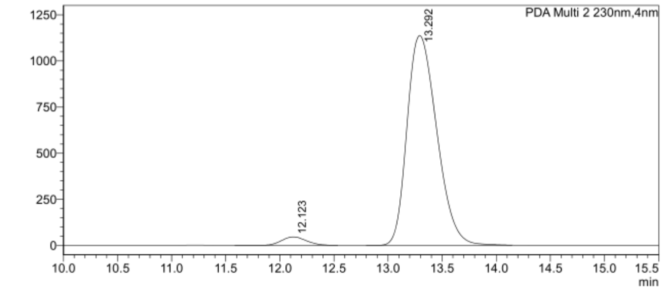

<Peak Table>

PDA Ch2 230nm

Peak#	Ret. Time	Height	Height%	Area	Area%
1	12.123	46570	3.933	755787	3.361
2	13.292	1137512	96.067	21732040	96.639
Total		1184083	100.000	22487827	100.000

9.24. *tert*-Butyl (*S*)-3-(naphthalen-2-ylmethyl)isoxazolidine-2-carboxylate(3x).

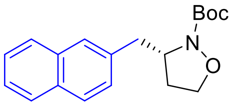

3x

Flash column chromatography on a silicagel (petroleum ether: ethyl acetate = 8:1) give the product as a colorless liquid (58 mg, 92% yield) with 96% *ee*. ESI-MS calculated for $C_{17}H_{23}NNaO_5$: m/z (%): 336.1570 ($M+Na^+$), found: 336.1566. Enantiomeric excess was determined by HPLC with a chiralpak ODH column (hexanes: 2-propanol = 90:10, 0.8 mL/min, 254 nm); major enantiomer tr = 12.120 min, minor enantiomer tr = 12.996 min. $[a]_D^{28}$ = 24.7 (*c* = 1.0, $CHCl_3$). ^1H NMR (400 MHz, $CDCl_3$) δ 7.84-7.76 (m, 3H), 7.68 (brs, 1H), 7.49-7.41 (m, 2H), 7.39 (dd, *J* = 8.4 Hz, *J* = 1.6 Hz, 1H), 4.52-4.43 (m, 1H), 4.08 (td, *J* = 8.0 Hz, *J* = 3.7 Hz, 1H), 3.76 (q, *J* = 8.4 Hz, 1H), 3.26 (dd, *J* = 13.5 Hz, *J* = 6.3 Hz, 1H), 2.87 (dd, *J* = 13.5 Hz, *J* = 8.0 Hz, 1H), 2.32-2.22 (m, 1H), 2.11-2.00 (m, 1H), 1.41 (s, 9H); ^{13}C NMR (101 MHz, $CDCl_3$) δ 157.14, 135.68, 133.46, 132.20, 127.94, 127.73, 127.68, 127.53, 127.45, 125.91, 125.37, 81.67, 68.96, 60.25, 41.70, 33.91, 28.05.

<Chromatogram>

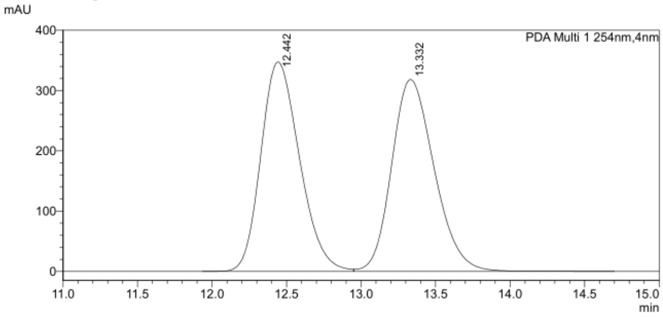

<Peak Table>

PDA Ch1 254nm

Peak#	Ret. Time	Height	Height%	Area	Area%
1	12.442	347397	52.167	6284740	49.830
2	13.332	318534	47.833	6327593	50.170
Total		665931	100.000	12612333	100.000

<Chromatogram>

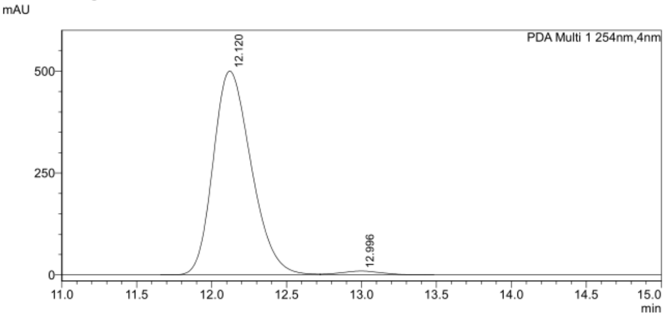

<Peak Table>

PDA Ch1 254nm

Peak#	Ret. Time	Height	Height%	Area	Area%
1	12.120	500794	98.125	8868348	97.943
2	12.996	9570	1.875	186224	2.057
Total		510364	100.000	9054572	100.000

9.25. *tert*-Butyl (*S*)-3-(naphthalen-1-ylmethyl)isoxazolidine-2-carboxylate(3y).

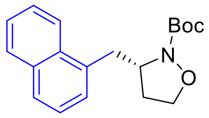

3y

Flash column chromatography on a silicagel (petroleum ether: ethyl acetate = 8:1) give the product as a white solid (54 mg, 87% yield) with 93% *ee*. Mp: 99.0-100.0 °C. ESI-MS calculated for $C_{17}H_{23}NNaO_5$: m/z (%): 336.1570 ($M+Na^+$), found: 336.1576. Enantiomeric excess was determined by HPLC with a chiralpak ODH column (hexanes: 2-propanol = 90:10, 0.8 mL/min, 210 nm); major enantiomer tr = 9.715 min, minor enantiomer tr = 11.033 min. $[a]_D^{29}$ = 30.6 (*c* = 1.0, $CHCl_3$). $[a]_D^{16}$ = -7.0 (*c* = 1.0, $CHCl_3$). ^1H NMR (400 MHz, $CDCl_3$) δ 8.19 (d, *J* = 8.4 Hz, 1H), 7.88-

7.84 (m, 1H), 7.76 (d, *J* = 8.1 Hz, 1H), 7.57-7.46 (m, 2H), 7.43-7.34 (m, 2H), 4.62 -4.53 (m, 1H), 4.14 (td, *J* = 7.9 Hz, *J* = 3.7 Hz, 1H), 3.77 (q, *J* = 8.4 Hz, 1H), 3.66 (dd, *J* = 13.6 Hz, *J* = 6.0 Hz, 1H), 3.06 (dd, *J* = 13.6 Hz, *J* = 8.7 Hz, 1H), 2.25-2.06 (m, 2H), 1.38 (s, 9H); ^{13}C NMR (101 MHz, $CDCl_3$) δ 156.78, 134.23, 133.81, 132.04, 128.69, 127.50, 127.33, 125.97, 125.53, 125.38, 123.80, 81.64, 69.02, 59.25, 39.17, 34.16, 28.00.

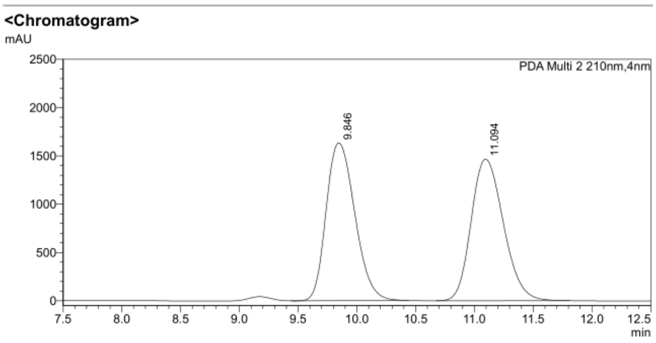

<Peak Table>
PDA Ch2 210nm

Peak#	Ret. Time	Area	Height	Height%	Area%
1	9.846	27821427	1635552	52.717	49.781
2	11.094	28066426	1466945	47.283	50.219
Total		55887852	3102497	100.000	100.000

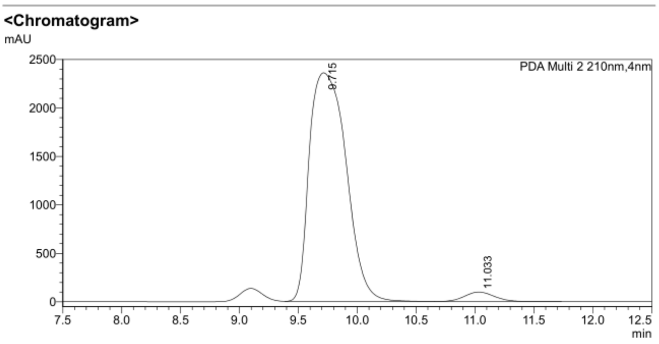

<Peak Table>
PDA Ch2 210nm

Peak#	Ret. Time	Height	Height%	Area	Area%
1	9.715	2360308	95.964	51296205	96.638
2	11.033	99275	4.036	1784750	3.362
Total		2459583	100.000	53080955	100.000

9.26. *tert*-Butyl (*S*)-3-((6-methoxynaphthalen-2-yl)methyl) isoxazolidine-2-carboxylate(3z).

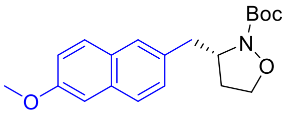

3z

Flash column chromatography on a silicagel (petroleum ether: ethyl acetate = 8:1) give the product as a white solid (121 mg, 88% yield) with 96% *ee*. Mp: 67.9-68.9 °C. ESI-MS calculated for $C_{20}H_{25}NNaO_4$: m/z (%): 366.1676 (M+Na$^+$), found: 366.1673. Enantiomeric excess was determined by HPLC with a chiralpak ADH column (hexanes: 2-propanol = 90:10, 0.8 mL/min, 254 nm); minor enantiomer tr = 8.645 min, major enantiomer tr = 9.668 min. $[a]_D^{25}$ = 22.1 (*c* = 1.0, $CHCl_3$). ^{1}H NMR (400 MHz, $CDCl_3$) δ 7.70-7.65 (m, 2H), 7.59 (s, 1H), 7.34 (dd, *J* = 8.4 Hz, *J* = 1.7 Hz, 1H), 7.16-7.09 (m, 2H), 4.52-4.37 (m, 1H), 4.06 (td, *J* = 8.0 Hz, *J* = 3.7 Hz, 1H), 3.89 (s, 3H), 3.75 (q, *J* = 8.3 Hz, 1H), 3.21 (dd, *J* = 13.5 Hz, *J* = 6.3 Hz, 1H), 2.81 (dd, *J* = 13.5 Hz, *J* = 8.0 Hz, 1H), 2.30-2.20 (m, 1H), 2.09-1.99 (m, 1H), 1.41 (s, 9H); ^{13}C NMR (101 MHz, $CDCl_3$) δ 157.21, 157.07, 133.24, 133.18, 128.86, 128.12, 127.49, 126.73, 118.64, 105.43, 81.54, 68.89, 60.27, 55.10, 41.43, 33.80, 27.98.

<Chromatogram>

mAU

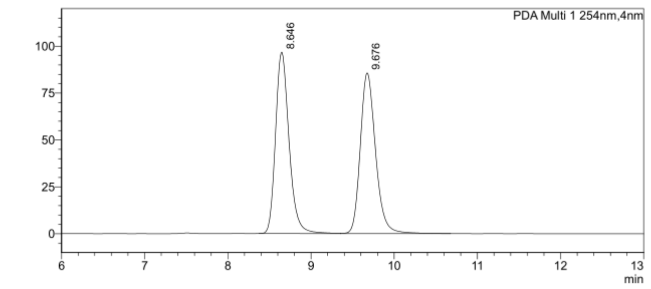

<Peak Table>

PDA Ch1 254nm

Peak#	Ret. Time	Height	Height%	Area	Area%
1	8.646	96630	52.980	1091543	49.942
2	9.676	85761	47.020	1094072	50.058
Total		182391	100.000	2185614	100.000

<Chromatogram>

mAU

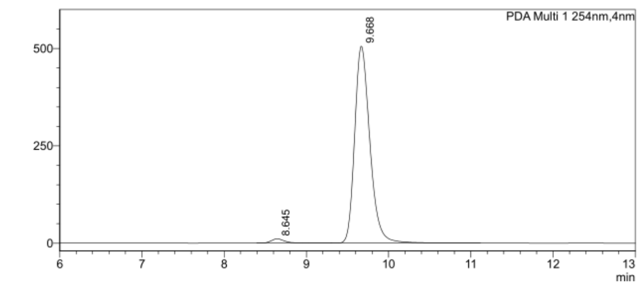

<Peak Table>

PDA Ch1 254nm

Peak#	Ret. Time	Height	Height%	Area	Area%
1	8.645	10994	2.127	133799	2.008
2	9.668	505953	97.873	6530863	97.992
Total		516946	100.000	6664662	100.000

9.27. ***tert*-Butyl (*S*)-3-(phenanthren-9-ylmethyl)isoxazolidine-2-carboxylate(3aa).**

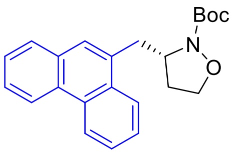

3aa

Flash column chromatography on a silicagel (petroleum ether: ethyl acetate = 7:1) give the product as a white solid (107 mg, 84% yield) with 95% *ee*. Mp: 102.3-104.5 ℃. ESI-MS calculated for $C_{23}H_{25}NNaO_3$: m/z (%): 386.1727 (M+Na$^+$), found: 386.1728. Enantiomeric excess was determined by HPLC with a chiralpak ADH column (hexanes: 2-propanol = 90:10, 0.8 mL/min, 254 nm); minor enantiomer tr = 8.031 min, major enantiomer tr = 10.880 min. $[a]_D^{16}$ = -17.9 (*c* = 1.0, $CHCl_3$). ^{1}H NMR (400 MHz, $CDCl_3$) δ 8.77-8.71 (m, 1H), 8.66 (d, *J* = 7.9 Hz, 1H), 8.30-8.24 (m, 1H), 7.84 (dd, *J* = 7.7 Hz, *J* = 1.5 Hz, 1H), 7.71-7.65 (m, 2H), 7.65-7.56 (m, 3H), 4.71-4.60 (m, 1H), 4.17 (td, *J* = 7.7 Hz, *J* = 4.1 Hz, 1H), 3.82-3.67 (m, 2H), 3.08 (dd, *J* = 13.7 Hz, *J* = 8.9 Hz, 1H), 2.25-2.09 (m, 2H), 1.40 (s, 9H); ^{13}C NMR (101 MHz, $CDCl_3$) δ 156.78, 132.36, 131.50, 130.99, 130.60, 129.81, 128.07, 127.93, 126.62, 126.57, 126.24, 124.36, 123.14, 122.31, 81.66, 69.03, 58.77, 39.65, 34.15, 27.96.

<Chromatogram>

mAU

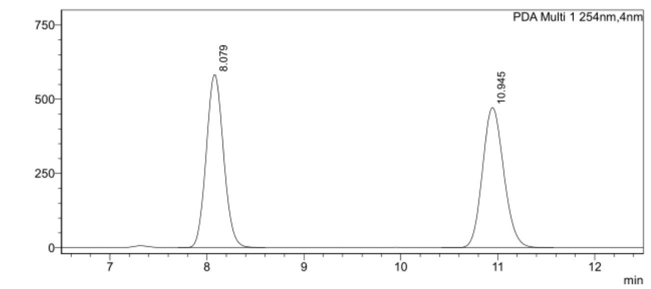

<Peak Table>

PDA Ch1 254nm

Peak#	Ret. Time	Height	Height%	Area	Area%
1	8.079	582510	55.221	7246951	50.086
2	10.945	472351	44.779	7222205	49.914
Total		1054861	100.000	14469155	100.000

<Chromatogram>

mAU

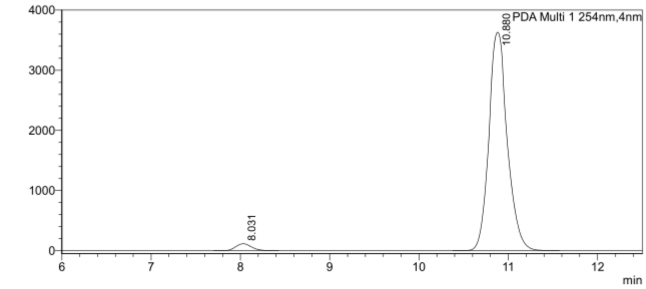

<Peak Table>

PDA Ch1 254nm

Peak#	Ret. Time	Height	Height%	Area	Area%
1	8.031	115806	3.087	1437738	2.690
2	10.880	3635902	96.913	52003336	97.310
Total		3751707	100.000	53441074	100.000

9.28. ***tert*-Butyl (*S*)-3-(quinolin-6-ylmethyl)isoxazolidine-2-carboxylate(3ab).**

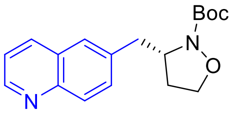

3ab

Flash column chromatography on a silicagel (petroleum ether: ethyl acetate = 2:1) give the product as a colorless liquid (123 mg, 85% yield) with 97% *ee*. ESI-MS calculated for $C_{18}H_{23}N_2O_3$: m/z (%): 315.1703 (M+H$^+$), found: 3315.1705. Enantiomeric excess was determined by HPLC with a chiralpak ADH column (hexanes: 2-propanol = 80:20, 0.8 mL/min, 254 nm); minor enantiomer tr = 8.940 min, major enantiomer tr = 9.821 min. $[a]_D^{17}$ = 25.7 (*c* = 1.0, $CHCl_3$). ^{1}H NMR (400 MHz, $CDCl_3$) δ 8.83-8.78 (m, 1H), 8.03 (d, *J* = 8.2 Hz, 1H), 7.99 (d, *J* = 8.6 Hz, 1H), 7.62-7.52 (m, 2H), 7.31 (dd, *J* = 8.3 Hz, *J* = 4.2 Hz, 1H), 4.47-4.37 (m, 1H), 4.03 (td, *J* = 8.1 Hz, *J* = 3.5 Hz, 1H), 3.70 (q, *J* = 8.2 Hz, 1H), 3.15 (dd, *J* = 13.6 Hz, *J* = 7.1 Hz, 1H), 2.85 (dd, *J* = 13.6 Hz, *J* = 7.1 Hz, 1H), 2.35-2.19 (m, 1H), 2.05-1.94 (m, 1H), 1.30 (s, 9H); ^{13}C NMR (101 MHz, $CDCl_3$) δ 157.07, 149.82, 147.15, 136.60, 135.50, 131.21, 129.23, 128.06, 127.49, 121.00, 81.64, 68.89, 60.06, 41.41, 33.96, 27.89.

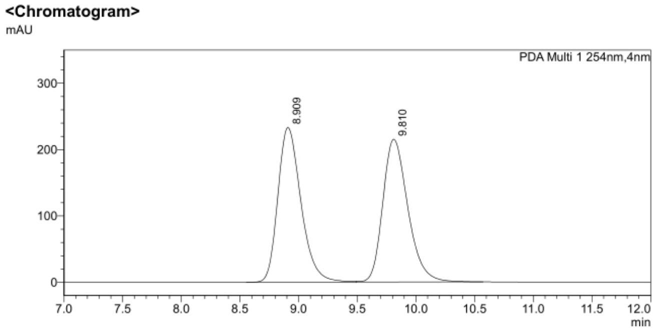

<Peak Table>
PDA Ch1 254nm

Peak#	Ret. Time	Height	Height%	Area	Area%
1	8.909	233423	51.996	3195338	49.971
2	9.810	215501	48.004	3199072	50.029
Total		448924	100.000	6394410	100.000

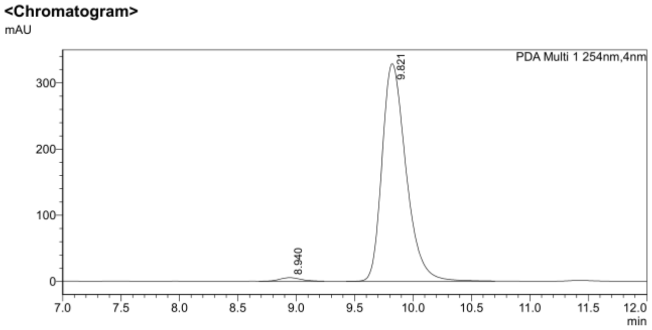

<Peak Table>
PDA Ch1 254nm

Peak#	Ret. Time	Height	Height%	Area	Area%
1	8.940	5341	1.596	65633	1.378
2	9.821	329184	98.404	4695841	98.622
Total		334525	100.000	4761474	100.000

9.29. ***tert*-Butyl (*S*)-3-(thiophen-3-ylmethyl)isoxazolidine-2-carboxylate(3ac).**

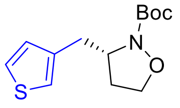

3ac

Flash column chromatography on a silicagel (petroleum ether: ethyl acetate = 10:1) give the product as a colorless liquid (104 mg, 97% yield) with 95% *ee*. ESI-MS calculated for $C_{13}H_{19}NNaO_3S$: m/z (%): 292.0978 (M+Na$^+$), found: 292.0973. Enantiomeric excess was determined by HPLC with a chiralpak ADH column (hexanes: 2-propanol = 90:10, 0.8 mL/min, 210 nm); major enantiomer tr = 6.918 min, minor enantiomer tr = 8.844 min. $[a]_D^{22}$ = 68.4 (*c* = 1.0, $CHCl_3$).

^{1}H NMR (300 MHz, $CDCl_3$) δ 7.26 (dd, *J* = 4.9 Hz, *J* = 3.0 Hz, 1H), 7.05 (d, *J* = 2.0 Hz, 1H), 6.99 (dd, *J* = 4.9 Hz, *J* = 1.0 Hz, 1H), 4.44-4.33 (m, 1H), 4.04 (td, *J* = 8.1 Hz, *J* = 3.8 Hz, 1H), 3.74 (q, *J* = 8.3 Hz, 1H), 3.06 (dd, *J* = 14.2 Hz, *J* = 6.4 Hz, 1H), 2.78 (dd, *J* = 14.2 Hz, *J* = 7.7 Hz, 1H), 2.39-2.24 (m, 1H), 2.09-1.91 (m, 1H), 1.46 (s, 9H); ^{13}C NMR (101 MHz, $CDCl_3$) δ 157.13, 138.25, 128.45, 125.25, 121.78, 81.58, 68.81, 59.51, 35.87, 33.89, 28.02.

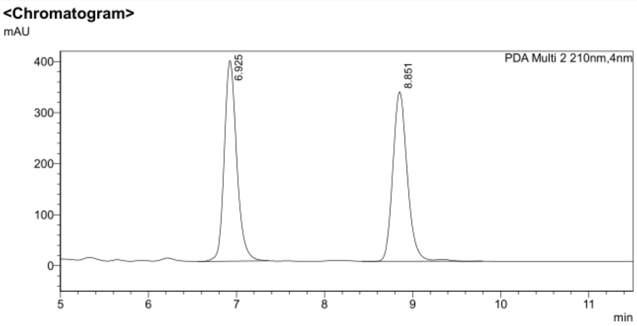

<Peak Table>

PDA Ch2 210nm

Peak#	Ret. Time	Height	Height%	Area	Area%
1	6.925	393353	54.210	3848459	50.340
2	8.851	332260	45.790	3796534	49.660
Total		725613	100.000	7644993	100.000

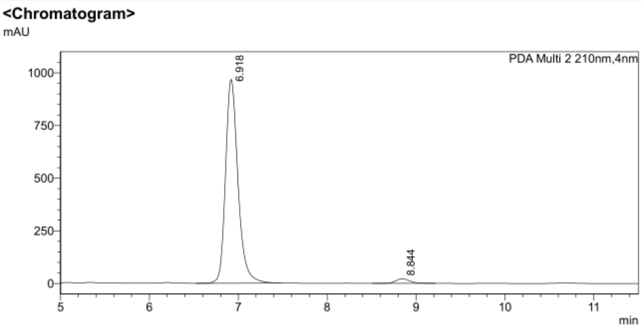

<Peak Table>

PDA Ch2 210nm

Peak#	Ret. Time	Height	Height%	Area	Area%
1	6.918	968337	97.767	9413440	97.423
2	8.844	22119	2.233	249025	2.577
Total		990456	100.000	9662466	100.000

10. Enantioselective synthesis of alkenyl substituted isoxazolidines.

10.1. *tert*-Butyl (*S*)-3-(cyclohex-1-en-1-ylmethyl)isoxazolidine-2-carboxylate(4a).

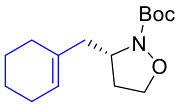

4a

Flash column chromatography on a silicagel (petroleum ether: ethyl acetate = 15:1) give the product as a colorless liquid (74 mg, 69% yield) with 89% *ee*. ESI-MS calculated for $C_{15}H_{25}NNaO_3$: m/z (%): 290.1727 ($M+Na^+$), found: 290.1723. Enantiomeric excess was determined by HPLC with a chiralpak ADH column (hexanes: 2-propanol = 95:5, 0.6 mL/min, 210 nm); minor enantiomer tr = 7.914 min, major enantiomer tr = 8.719 min. $[a]_D^{16}$ = 72.9 (*c* = 1.0, $CHCl_3$) ^{1}H NMR (400 MHz, $CDCl_3$) δ 5.45 (br, 1H), 4.29 – 4.20 (m, 1H), 4.01 (td, *J* = 8.0 Hz, *J* = 4.0 Hz, 1H), 3.72 (q, *J* = 8.1 Hz, 1H), 2.34 (dd, *J* = 13.5 Hz, *J* = 6.2 Hz, 1H), 2.30-2.20 (m, 1H), 2.04-1.86 (m, 6H), 1.62- 1.55 (m, 2H), 1.54-1.49 (m, 2H), 1.46 (s, 9H); ^{13}C NMR (101 MHz, $CDCl_3$) δ 157.36, 134.25, 124.03, 81.45, 68.76, 57.55, 43.94, 33.57, 28.25, 28.14, 25.18, 22.80, 22.26.

<Chromatogram>

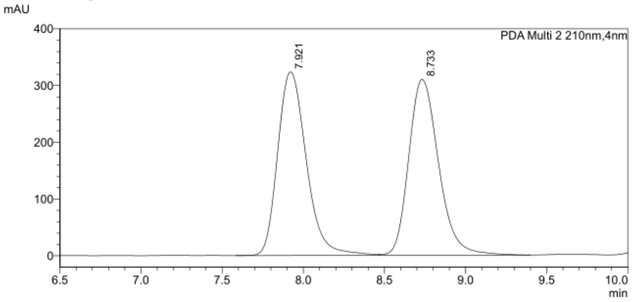

<Peak Table>

PDA Ch2 210nm

Peak#	Ret. Time	Height	Height%	Area	Area%
1	7.921	323754	51.076	3968639	50.033
2	8.733	310119	48.924	3963454	49.967
Total		633873	100.000	7932093	100.000

<Chromatogram>

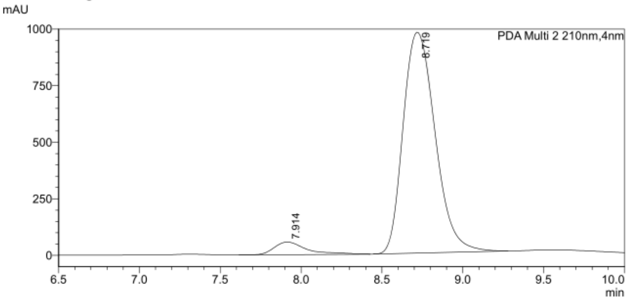

<Peak Table>

PDA Ch2 210nm

Peak#	Ret. Time	Height	Height%	Area	Area%
1	7.914	55951	5.420	788003	5.533
2	8.719	976325	94.580	13453579	94.467
Total		1032276	100.000	14241582	100.000

10.2 *tert*-Butyl (*S*)-3-(2-methylenebutyl)isoxazolidine-2-carboxylate(4b).

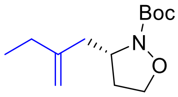

4b

Flash column chromatography on a silicagel (petroleum ether: ethyl acetate = 15:1) give the product as a colorless liquid (62 mg, 64% yield) with 90% *ee*. ESI-MS calculated for $C_{13}H_{23}NNaO_3$: m/z (%): 264.1570 ($M+Na^+$), found: 264.1565. Enantiomeric excess was determined by HPLC with a chiralpak ADH column (hexanes: 2-propanol = 95:5, 0.6 mL/min, 210 nm); major enantiomer tr = 7.768 min, minor enantiomer tr = 8.424 min. $[a]_D^{27}$ = 78.0 (*c* = 1.0, $CHCl_3$). ^1H NMR (400 MHz, $CDCl_3$) δ 4.81-4.73 (m, 2H), 4.33-4.24 (m, 1H), 4.02 (td, *J* = 8.0 Hz, *J* = 3.9 Hz, 1H), 3.72 (q, *J* = 8.1 Hz, 1H), 2.48 (dd, *J* = 14.1 Hz, *J* = 6.1 Hz, 1H), 2.33-2.23 (m, 1H), 2.10 (dd, *J* = 11.8 Hz, *J* = 6.2 Hz, 1H), 2.07-2.01 (m, 2H), 1.99-1.89 (m, 1H), 1.46 (s, 9H), 1.02 (t, *J* = 7.4 Hz, 3H); ^{13}C NMR (101 MHz, $CDCl_3$) δ 157.37, 147.61, 110.60, 81.58, 68.77, 57.60, 42.04, 33.73, 28.59, 28.13, 12.16.

<Chromatogram>

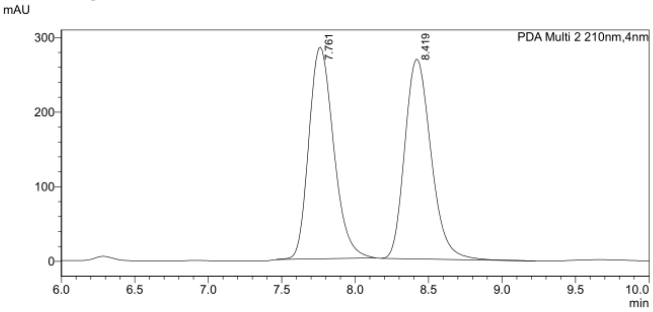

<Peak Table>

PDA Ch2 210nm

Peak#	Ret. Time	Height	Height%	Area	Area%
1	7.761	283589	51.435	3336949	50.006
2	8.419	267763	48.565	3336094	49.994
Total		551353	100.000	6673043	100.000

<Chromatogram>

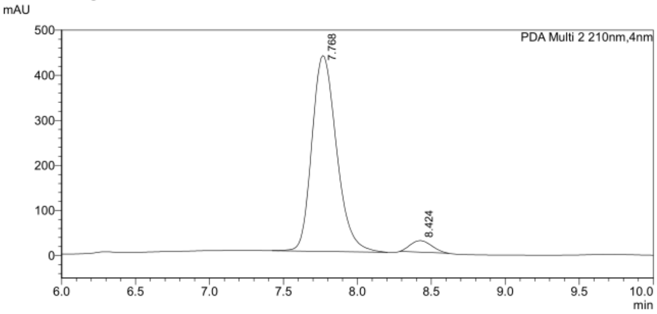

<Peak Table>

PDA Ch2 210nm

Peak#	Ret. Time	Height	Height%	Area	Area%
1	7.768	435031	94.447	5039062	95.131
2	8.424	25576	5.553	257897	4.869
Total		460607	100.000	5296960	100.000

10.3. *tert*-Butyl (*S*)-3-(2-methylallyl)isoxazolidine-2-carboxylate(4c).

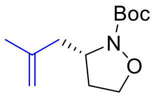

4c

Flash column chromatography on a silicagel (petroleum ether: ethyl acetate = 15:1) give the product as a colorless liquid (70 mg, 77% yield) with 91% *ee*. ESI-MS calculated for $C_{12}H_{21}NNaO_3$: m/z (%): 250.1414 ($M+Na^+$), found: 250.1410. Enantiomeric excess was determined by HPLC with a chiralpak IC column (hexanes: 2-propanol = 97:3, 0.8 mL/min, 210 nm); major enantiomer tr = 50.350 min, minor enantiomer tr = 53.314 min. $[a]_D^{29}$ = 88.3 (*c* = 1.0, $CHCl_3$). ^1H NMR (400 MHz, $CDCl_3$) δ 4.75 (d, *J* = 18.4 Hz, 2H), 4.34-4.24 (m, 1H), 4.03 (td, *J* = 8.1 Hz, *J* = 3.9 Hz, 1H), 3.72 (q, *J* = 8.1 Hz, 1H), 2.43 (dd, *J* = 13.9 Hz, *J* = 6.3 Hz, 1H), 2.34-2.24 (m, 1H), 2.09 (dd, *J* = 13.9 Hz, *J* = 8.2 Hz, 1H), 1.99-1.88 (m, 1H), 1.74 (s, 3H), 1.46 (s, 9H); ^{13}C NMR (101 MHz, $CDCl_3$) δ 157.42, 142.09, 112.85, 81.60, 68.76, 57.44, 43.63, 33.67, 28.11, 22.32.

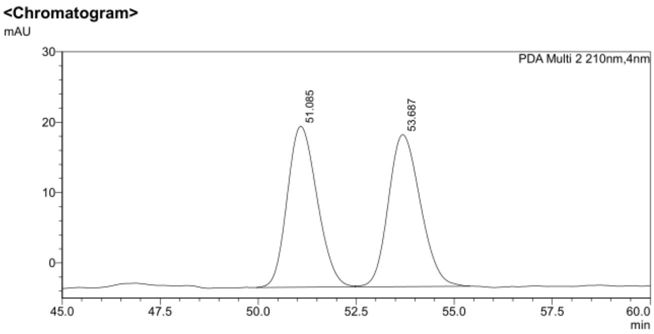

<Peak Table>
PDA Ch2 210nm

Peak#	Ret. Time	Height	Height%	Area	Area%
1	51.085	22868	51.445	1214464	49.710
2	53.687	21584	48.555	1228631	50.290
Total		44452	100.000	2443095	100.000

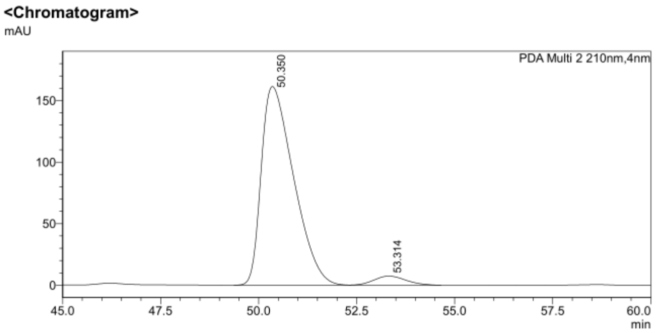

<Peak Table>
PDA Ch2 210nm

Peak#	Ret. Time	Height	Height%	Area	Area%
1	50.350	161375	95.647	9272841	95.695
2	53.314	7345	4.353	417116	4.305
Total		168719	100.000	9689957	100.000

10.4 tert-butyl (S)-3-([1,1'-biphenyl]-4-ylmethyl)-3-methylisoxazolidine-2-carboxylate(4d)

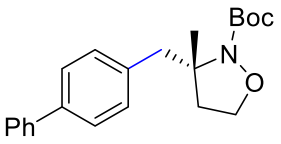

4d

Flash column chromatography on a silicagel (petroleum ether: ethyl acetate = 8: 1) give the product as a white solid (39 mg, 27% yield) with 67% *ee*. Enantiomeric excess was determined by HPLC with a chiralpak ODH column (hexanes: 2-propanol = 90:10, 0.8 mL/min, 250 nm); minor enantiomer tr = 8.787 min, major enantiomer tr = 9.565 min. ^1H NMR (400 MHz, $CDCl_3$) δ 7.58 (d, *J* = 7.4 Hz, 2H), 7.52 (d, *J* = 7.9 Hz, 2H), 7.43 (t, *J* = 7.5 Hz, 2H), 7.33 (t, *J* = 7.3 Hz, 1H), 7.28 (d, *J* = 7.9 Hz, 2H), 3.86

(q, J = 7.3 Hz, 1H), 3.56 (q, J = 7.2 Hz, 1H), 3.30 (d, J = 13.4 Hz, 1H), 3.03 (d, J = 13.4 Hz, 1H), 2.55-2.42 (m, J = 12.7, 6.5 Hz, 1H), 2.06-1.98 (m, 1H), 1.57 (s, 9H), 1.56 (s, 3H); ^{13}C NMR (101 MHz, $CDCl_3$) δ 152.83, 140.81, 139.49, 136.40, 130.95, 128.72, 127.15, 126.96, 126.85, 81.27, 77.32, 77.00, 76.68, 65.93, 64.43, 43.56, 41.21, 28.53, 25.24.

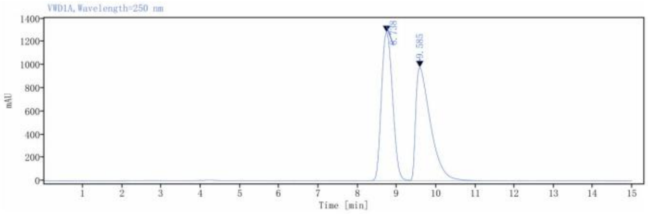

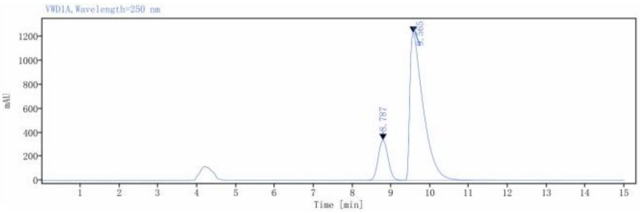

VWD1A, Wavelength=250 nm

Ret. Time [min]	Area	Height	Height%	Area%
8.738	25208.23	1280.24	56.72	49.75
9.585	25462.11	976.69	43.28	50.25
Total.	50670.34	2256.93	100.00	100.00

VWD1A, Wavelength=250 nm

Ret. Time [min]	Area	Height	Height%	Area%
8.787	5893.68	333.00	21.31	16.72
9.565	29359.76	1229.45	78.69	83.28
Total.	35253.44	1562.44	100.00	100.00

11. Enantioselective synthesis of aryl substituted pyrrolidines.

11.1. *tert*-Butyl (*R*)-2-([1,1'-biphenyl]-4-ylmethyl)pyrrolidine-1-carboxylate(5a).

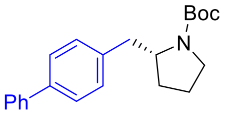

5a

Flash column chromatography on a silicagel (petroleum ether: ethyl acetate = 15:1) give the product as a white solid (77 mg, 57% yield) with 87% *ee*. Mp: 80.0-81.3 °C.ESI-MS calculated for $C_{22}H_{27}NNaO_2$: m/z (%): 360.1934 (M+Na$^+$), found: 360.1934. Enantiomeric excess was determined by HPLC with a chiralpak OJH column (hexanes: 2-propanol = 98:2, 0.5 mL/min, 250 nm); major enantiomer tr = 22.740 min, minor enantiomer tr = 26.205 min. $[a]_D^{16}$ = -4.6 (c = 1.0, $CHCl_3$).^{1}H NMR (400 MHz, $CDCl_3$) δ 7.54 (dd, J = 24.3 Hz, J = 7.3 Hz, 4H), 7.41 (t, J = 6.4 Hz, 2H), 7.35-7.15 (m, 3H), 4.17-3.90 (br, 1H), 3.50-3.25 (br, 2H), 3.25-2.95 (br, 1H), 2.70-2.50 (br, 1H), 1.85 -1.70 (br, 4H), 1.51 (s, 9H); ^{13}C NMR (101 MHz, $CDCl_3$) δ 154.49, 140.93, 139.08, 138.27, 129.77, 128.65, 127.01, 126.90, 79.18 & 79.02, 58.72, 46.75 & 46.26, 40.23 & 39.21, 29.76 & 28.97, 28.56, 23.38 & 22.63.

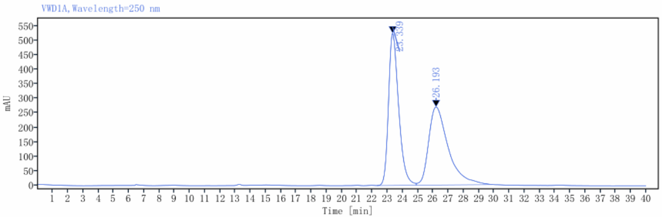

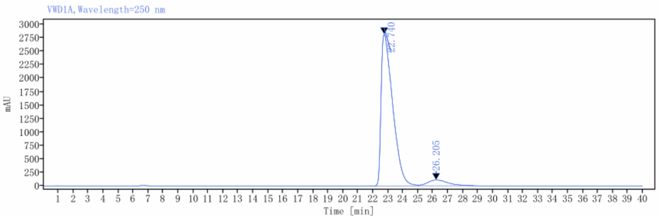

VWD1A, Wavelength=250 nm

Ret. Time [min]	Area	Height	Height%	Area%
23.339	24370.52	526.25	66.10	50.09
26.193	24287.02	269.93	33.90	49.91
Total.	48657.54	796.18	100.00	100.00

VWD1A, Wavelength=250 nm

Ret. Time [min]	Area	Height	Height%	Area%
22.740	150952.20	2811.31	96.37	93.74
26.205	10078.55	106.02	3.63	6.26
Total.	161030.75	2917.33	100.00	100.00

11.2. *tert*-Butyl (*R*)-2-(naphthalen-2-ylmethyl)pyrrolidine-1-carboxylate(5b).

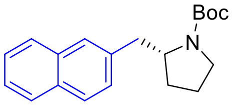

5b

Flash column chromatography on a silicagel (petroleum ether: ethyl acetate = 15:1) give the product as a white solid (60 mg, 48% yield) with 92% *ee*. Mp: 62.3-64.0 °C.ESI-MS calculated for $C_{20}H_{25}NNaO_2$: m/z (%): 334.1777 (M+Na$^+$), found: 334.1771. Enantiomeric excess was determined by HPLC with a chiralpak OJH column (hexanes: 2-propanol = 98:2, 0.5 mL/min, 250 nm); minor enantiomer tr = 18.755 min, major enantiomer tr = 22.040 min. $[a]_D^{25}$ = -8.7 (*c* = 1.0, $CHCl_3$). ^{1}H NMR (400 MHz, $CDCl_3$) δ 7.85-7.74 (m, 3H), 7.63 (d, *J* = 18.3 Hz, 1H), 7.50-7.30 (m, 3H), 4.25-4.00 (br, 1H), 3.50-3.15 (br, 3H), 2.80-2.63 (m, 1H), 1.84-1.71 (brs, 4H), 1.54 (s, 9H); ^{13}C NMR (101 MHz, $CDCl_3$) δ 154.54, 136.79 & 136.77, 133.56 & 133.53, 132.15 & 132.11, 128.19, 127.93, 127.74, 127.59 &127.56, 127.44 & 127.40, 125.90, 125.25, 79.23 & 79.05, 58.72, 46.79 & 46.32, 40.77 & 39.71, 29.72 & 28.94, 28.62 & 28.59, 23.45 & 22.69.

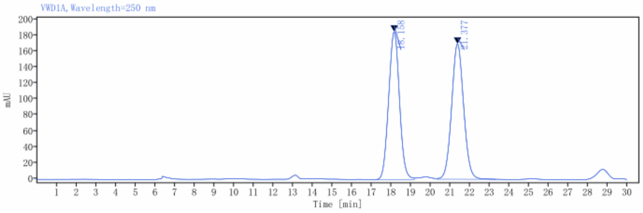

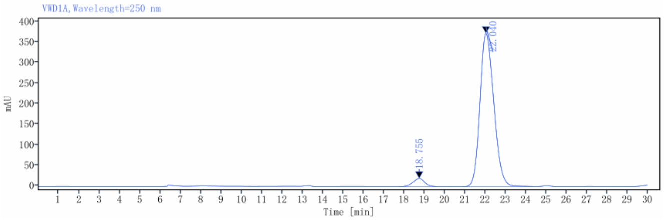

VWD1A, Wavelength=250 nm

Ret. Time [min]	Area	Height	Height%	Area%
18.158	7091.08	185.42	52.22	49.58
21.377	7212.21	169.65	47.78	50.42
Total.	14303.30	355.06	100.00	100.00

VWD1A, Wavelength=250 nm

Ret. Time [min]	Area	Height	Height%	Area%
18.755	779.18	19.63	5.01	4.24
22.040	17595.77	372.00	94.99	95.76
Total.	18374.95	391.63	100.00	100.00

11.3. ***tert*-Butyl (*R*)-2-((6-methoxynaphthalen-2-yl)methyl) pyrrolidine-1-carboxylate(5c).**

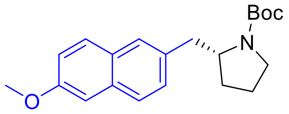

5c

Flash column chromatography on a silicagel (petroleum ether: ethyl acetate = 20:1) give the product as a white solid (90 mg, 66% yield) with 95% *ee*. Mp: 62.9-64.8 °C. ESI-MS calculated for $C_{21}H_{27}NNaO_3$: m/z (%): 364.1883 ($M+Na^+$), found: 364.1888. Enantiomeric excess was determined by HPLC with a chiralpak OJH column (hexanes: 2-propanol = 98:2, 0.5 mL/min, 250 nm); major enantiomer tr = 33.557 min, minor enantiomer tr = 44.692 min. $[a]_D^{18}$ = -8.2 (*c* = 1.0, $CHCl_3$). ^1H NMR (400 MHz, $CDCl_3$) δ 7.66 (d, *J* = 8.6 Hz, 2H), 7.60-7.48 (br, 1H), 7.38-7.22 (br, 1H), 7.11 (d, *J* = 11.6 Hz, 1H), 4.15-3.97 (br, 1H), 3.88 (s, 3H), 3.47-3.10 (br, 3H), 2.75-2.56(br, 1H), 1.92-1.67 (br, 4H), 1.52 (s, 9H); ^{13}C NMR (101 MHz, $CDCl_3$) δ 157.22, 154.48, 134.33, 133.09, 128.98, 128.83, 128.59 & 128.24, 127.51, 126.69, 118.63, 105.59, 79.09 & 78.92, 58.71, 55.15, 46.71 & 46.26, 40.48 & 39.46, 29.60 & 28.89, 28.54, 23.34 & 22.61.

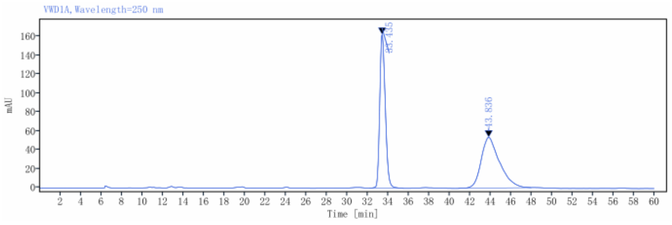

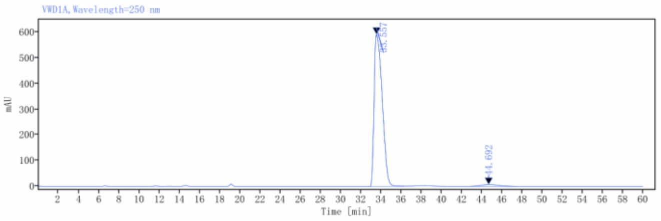

VWD1A, Wavelength=250 nm

Ret. Time [min]	Area	Height	Height%	Area%
33.435	6112.62	162.35	75.19	47.78
43.836	6680.66	53.57	24.81	52.22
Total.	12793.28	215.92	100.00	100.00

VWD1A, Wavelength=250 nm

Ret. Time [min]	Area	Height	Height%	Area%
33.557	32848.13	590.86	98.86	97.49
44.692	845.14	6.79	1.14	2.51
Total.	33693.27	597.65	100.00	100.00

11.4. ***tert*-Butyl (*R*)-2-(naphthalen-1-ylmethyl)pyrrolidine-1-carboxylate(5d).**

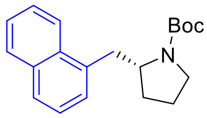

5d

Flash column chromatography on a silicagel (petroleum ether: ethyl acetate = 15:1) give the product as a white solid (91 mg, 73% yield) with 89% *ee*. Mp: 39.6-40.0 °C.ESI-MS calculated for $C_{20}H_{25}NNaO_2$: m/z (%): 334.1777 ($M+Na^+$), found: 334.1771. Enantiomeric excess was determined by HPLC with a chiralpak OJH column (hexanes: 2-propanol = 98:2, 0.5 mL/min, 250 nm); minor enantiomer tr = 11.607 min, major enantiomer tr = 12.857 min. $[a]_D^{15}$ = -107.8

(c = 1.0, $CHCl_3$). ^{1}H NMR (400 MHz, $CDCl_3$) δ 8.38 (d, J = 79.1 Hz, 1H), 7.86-7.65 (m, 2H), 7.59-7.18 (m, 4H), 4.32-4.20 (br, 1H), 4.00-3.60 (m, 1H), 3.51-3.23 (m, 2H), 2.77 (t, J = 11.1 Hz, 1H), 2.00-1.59 (m, 4H), 1.53 (s, 9H); ^{13}C NMR (101 MHz, $CDCl_3$) δ 154.58, 135.60 & 135.32, 133.79, 132.43 & 132.28, 128.60 & 128.30, 127.69 & 127.58, 126.99, 126.10 & 125.48, 125.40 & 125.17, 124.83 & 124.28, 79.59 & 78.91, 57.58 & 57.24, 46.71 & 46.30, 37.82 & 36.90, 29.61 & 28.79, 28.60, 23.33 & 22.40.

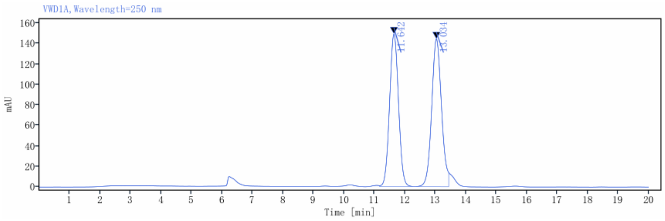

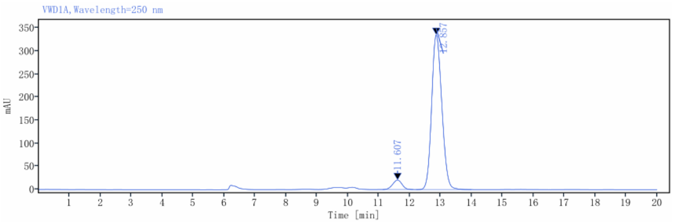

VWD1A, Wavelength=250 nm

Ret. Time [min]	Area	Height	Height%	Area%
11.642	3026.38	148.83	50.80	48.48
13.034	3216.21	144.15	49.20	51.52
Total.	6242.59	292.98	100.00	100.00

VWD1A, Wavelength=250 nm

Ret. Time [min]	Area	Height	Height%	Area%
11.607	431.84	20.52	5.76	5.33
12.857	7675.69	335.47	94.24	94.67
Total.	8107.53	355.99	100.00	100.00

11.5. *tert*-Butyl (*R*)-2-(benzofuran-5-ylmethyl)pyrrolidine-1-carboxylate(5e).

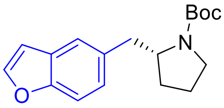

5e

Flash column chromatography on a silicagel (petroleum ether: ethyl acetate = 20:1) give the product as a colorless liquid (66 mg, 55% yield) with 91% *ee*. ESI-MS calculated for $C_{18}H_{23}NNaO_3$: m/z (%): 324.1570 ($M+Na^+$), found: 324.1568. Enantiomeric excess was determined by HPLC with a chiralpak OJH column (hexanes: 2-propanol = 98:2, 0.5 mL/min, 250 nm); minor enantiomer tr = 13.126 min, major enantiomer tr = 14.368 min. $[a]_D^{26}$ = -9.3 (c = 1.0, $CHCl_3$). ^{1}H NMR (400 MHz, $CDCl_3$) δ 7.58 (s, 1H), 7.46-7.32 (m, 2H), 7.24-7.04 (m, 1H), 6.70 (s, 1H), 4.16-3.90 (br, 1H), 3.45-3.05 (br, 3H), 2.70-2.55 (m, 1H), 1.80-1.68 (br, 4H), 1.51 (s, 9H); ^{13}C NMR (101 MHz, $CDCl_3$) δ 154.50, 153.75, 145.04, 133.58, 127.49, 125.87 & 125.65, 121.48, 111.00, 106.30, 79.12 & 78.93, 59.07, 46.75 & 46.27, 40.35 & 39.41, 29.55 & 28.74, 28.55, 23.37 & 22.60.

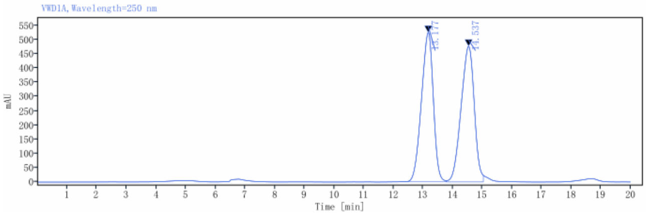

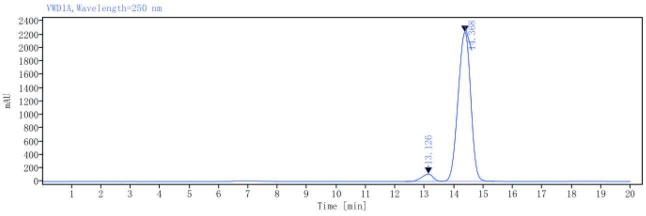

VWD1A, Wavelength=250 nm

Ret. Time [min]	Area	Height	Height%	Area%
13.177	14603.43	524.22	52.40	49.82
14.537	14707.26	476.25	47.60	50.18
Total.	29310.69	1000.47	100.00	100.00

VWD1A, Wavelength=250 nm

Ret. Time [min]	Area	Height	Height%	Area%
13.126	3073.57	110.62	4.72	4.40
14.368	66707.51	2230.79	95.28	95.60
Total.	69781.08	2341.41	100.00	100.00

11.6 *tert*-butyl (S)-2-(3,4-dimethoxybenzyl)-4,4-dimethylpyrrolidine-1-carboxylate(5f)

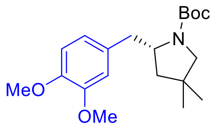

5f

Flash column chromatography on a silicagel (petroleum ether: ethyl acetate = 5: 1) give the product as a colorless liquid (10 mg, 15% yield) with 81% *ee*. Enantiomeric excess was determined by HPLC with a chiralpak ODH column (hexanes: 2-propanol = 95:5, 0.5 mL/min, 230 nm); minor enantiomer tr = 11.374 min, major enantiomer tr = 12.629 min. ^{1}H NMR (400 MHz, $CDCl_3$) δ 6.82-6.62 (m, 3H), 4.08-3.91 (m, 1H), 3.86 (d, *J* = 2.6 Hz, 6H), 3.47-3.10 (m, 2H), 2.82 (d, *J* = 10.7 Hz, 1H), 2.59 (dd, *J* = 12.7, 9.3 Hz, 1H), 1.69-1.39 (m, 11H), 1.02 (s, 1H), 0.93 (s, 1H); ^{13}C NMR (101 MHz, $CDCl_3$) δ 155.00 & 154.94, 148.73, 147.42, 131.31, 121.63 & 121.34, 112.76 & 112.63, 111.13 & 110.93, 79.17 & 78.98, 77.32, 77.00, 76.68, 59.89 & 58.95, 58.52 & 58.26, 55.81, 45.55 & 44.61, 40.89 & 39.48, 36.99 & 36.81, 28.57, 26.34, 26.07.

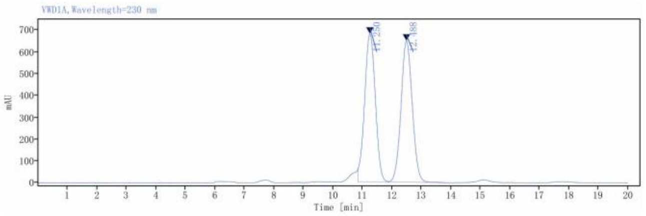

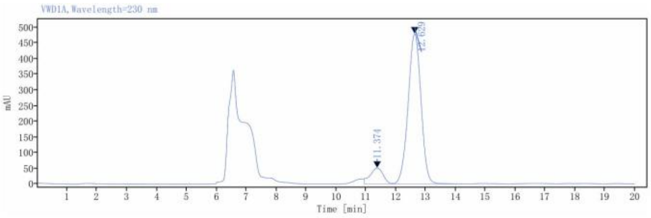

VWD1A, Wavelength=230 nm

Ret. Time [min]	Area	Height	Height%	Area%
11.250	17165.32	678.40	51.14	50.00
12.488	17165.04	648.03	48.86	50.00
Total.	34330.37	1326.43	100.00	100.00

VWD1A, Wavelength=230 nm

Ret. Time [min]	Area	Height	Height%	Area%
11.374	1549.76	50.75	9.57	9.74
12.629	14358.88	479.28	90.43	90.26
Total.	15908.63	530.03	100.00	100.00

11.7 benzyl (R)-2-([1,1'-biphenyl]-4-ylmethyl)pyrrolidine-1-carboxylate(5g)

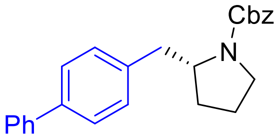

5g

Flash column chromatography on a silicagel (petroleum ether: ethyl acetate = 10: 1) give the product as a colorless liquid (36 mg, 48% yield) with 60% *ee*. Enantiomeric excess was determined by HPLC with a chiralpak OJH column (hexanes: 2-propanol = 90:10, 0.8 mL/min, 250 nm); major enantiomer tr = 42.687 min, minor enantiomer tr = 51.524 min. ^{1}H NMR (400 MHz, $CDCl_3$) δ 7.62-7.25 (m, 13H), 7.14 (d, *J* = 7.0 Hz, 1H), 5.19 (s, 2H), 4.20-4.03 (m, 1H), 3.42 (d, *J* = 16.9 Hz, 2H), 3.15 (dd, *J* = 72.6, *J* = 12.6 Hz, 1H), 2.63 (dt, *J* = 17.3, *J* = 13.0 Hz, 1H), 1.78 (s, 4H). ^{13}C NMR (101 MHz, $CDCl_3$) δ 154.87

(d, *J* = 7.4 Hz), 140.88, 139.13, 138.03, 136.88, 129.94, 129.74, 128.69, 128.45, 128.04, 127.80, 127.01, 126.93, 77.32, 77.00, 76.68, 66.71 (d, *J* = 45.4 Hz), 59.00 (d, *J* = 47.8 Hz), 46.70 (d, *J* = 22.7 Hz), 39.65 (d, *J* = 121.0 Hz), 29.39 (d, *J* = 89.3 Hz), 23.07 (d, *J* = 80.6 Hz).

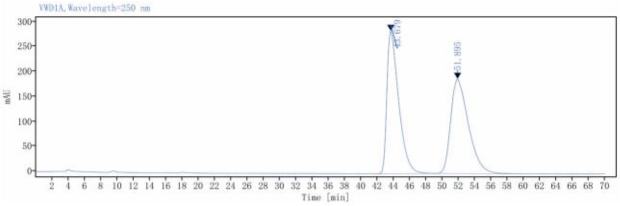

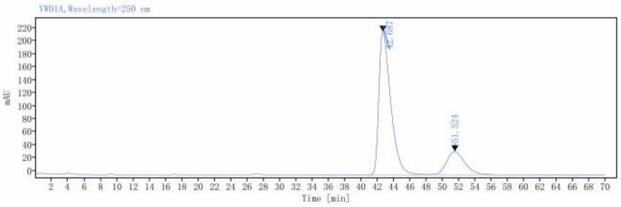

VWD1A, Wavelength=250 nm

Ret. Time [min]	Area	Height	Height%	Area%
43.679	28750.31	285.85	60.15	50.02
51.895	28721.69	189.38	39.85	49.98
Total.	57472.00	475.24	100.00	100.00

VWD1A, Wavelength=250 nm

Ret. Time [min]	Area	Height	Height%	Area%
42.687	22184.40	219.95	86.16	80.15
51.524	5493.11	35.33	13.84	19.85
Total.	27677.51	255.29	100.00	100.00

12. ^{1}H, ^{19}F, ^{31}P, ^{13}C NMR .

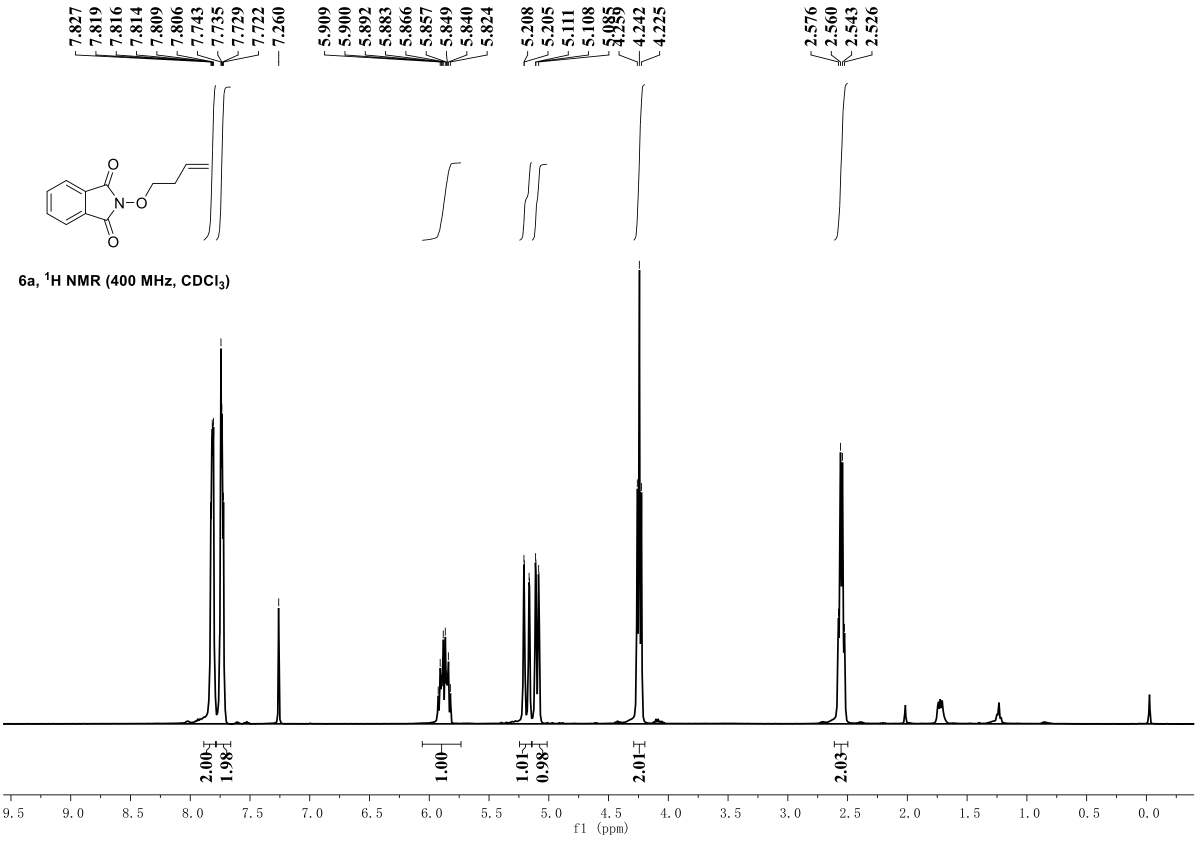

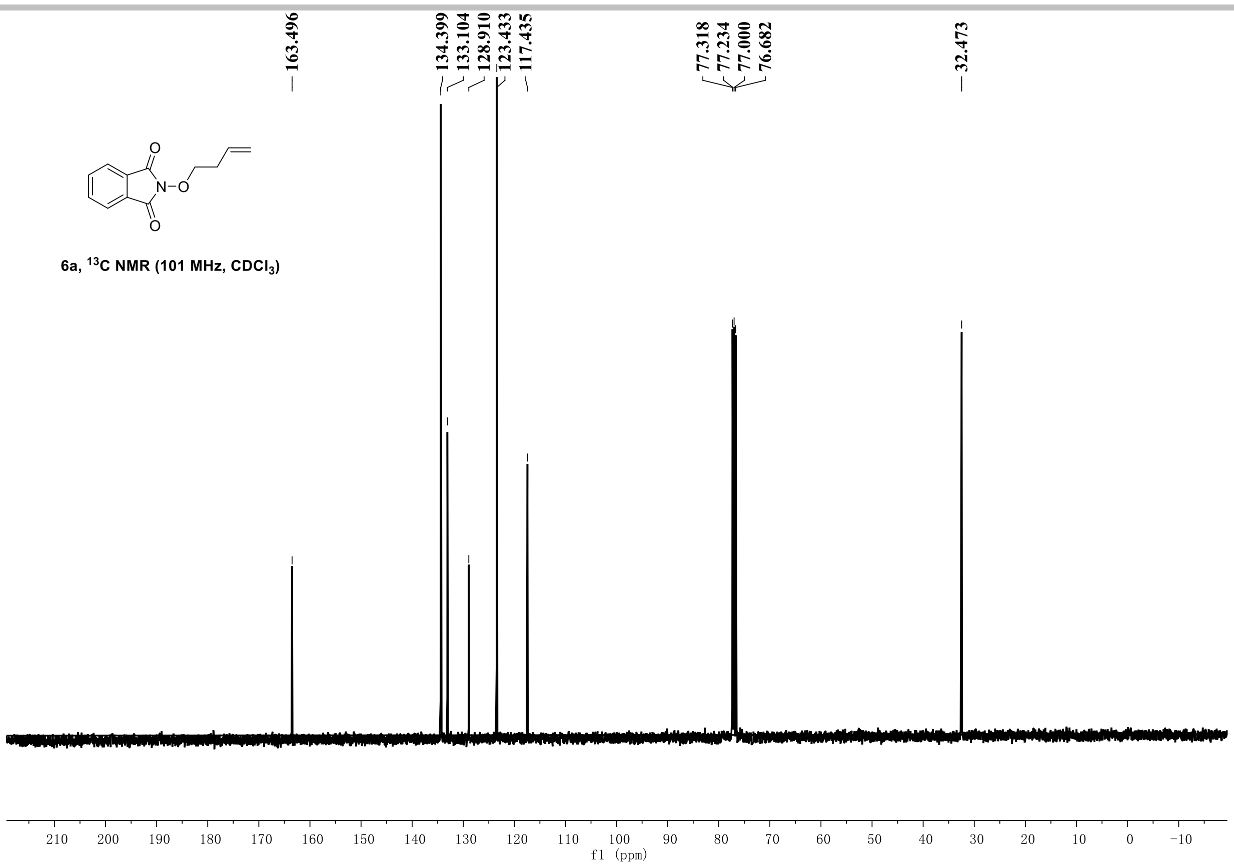
163.496
134.399
133.104
128.910
123.433
117.435
77.318
77.234
77.000
76.682
32.473
O
N
O
O
6a, 13C NMR (101 MHz, CDCl3)
210 200 190 180 170 160 150 140 130 120 110 100 90 80 70 60 50 40 30 20 10 0 −10
f1 (ppm)

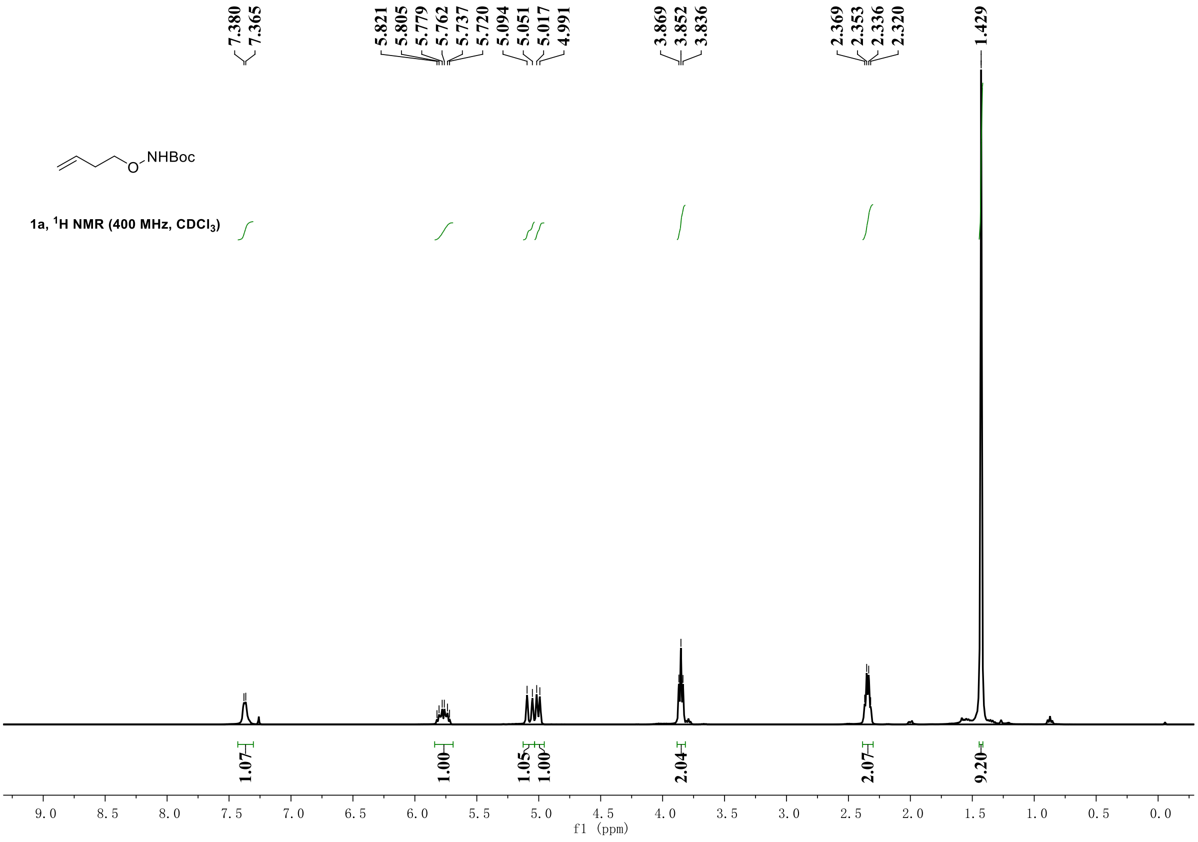
7.380
7.365
5.821
5.805
5.779
5.762
5.737
5.720
5.094
5.051
5.017
4.991
3.869
3.852
3.836
2.369
2.353
2.336
2.320
1.429
O
NHBoc
1a, 1H NMR (400 MHz, CDCl3)
1.07
1.00
1.05
1.00
2.04
2.07
9.20
9.0 8.5 8.0 7.5 7.0 6.5 6.0 5.5 5.0 4.5 4.0 3.5 3.0 2.5 2.0 1.5 1.0 0.5 0.0
f1 (ppm)

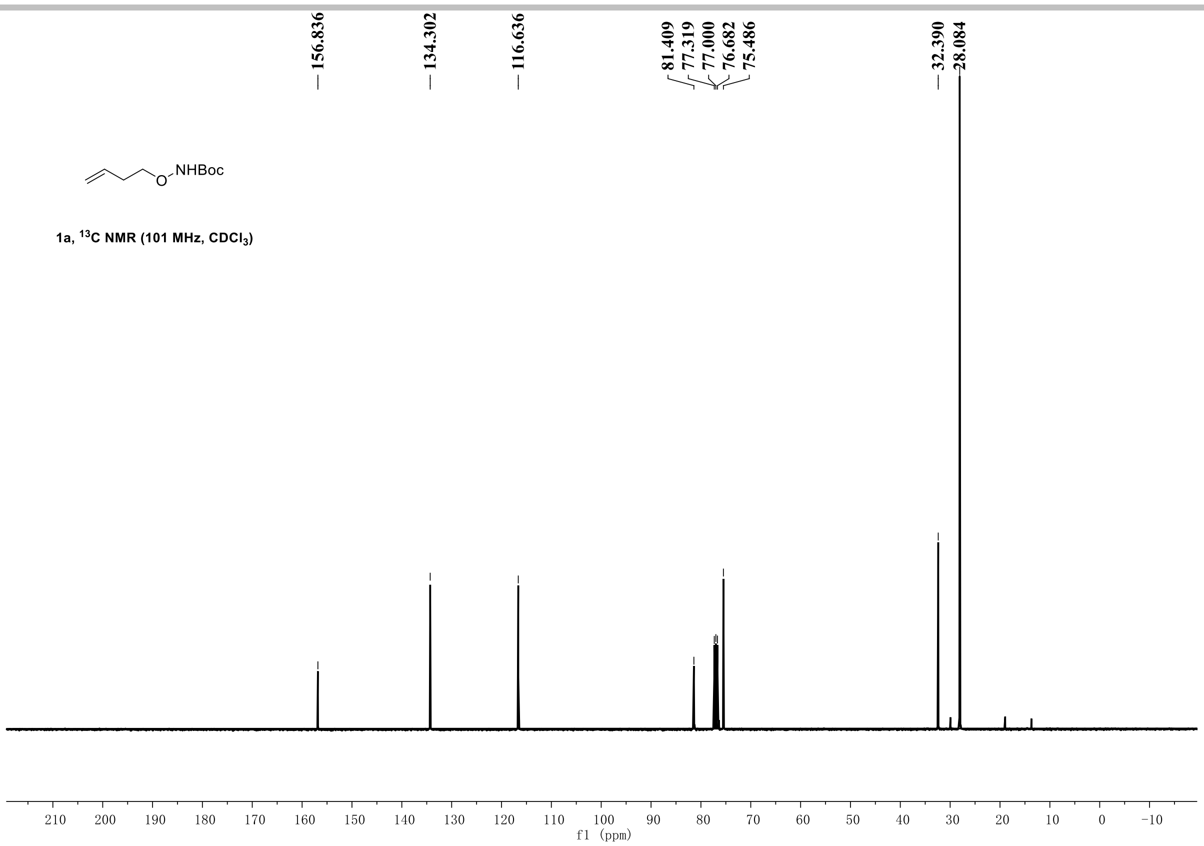

1a, ^{13}C NMR (101 MHz, $CDCl_3$)

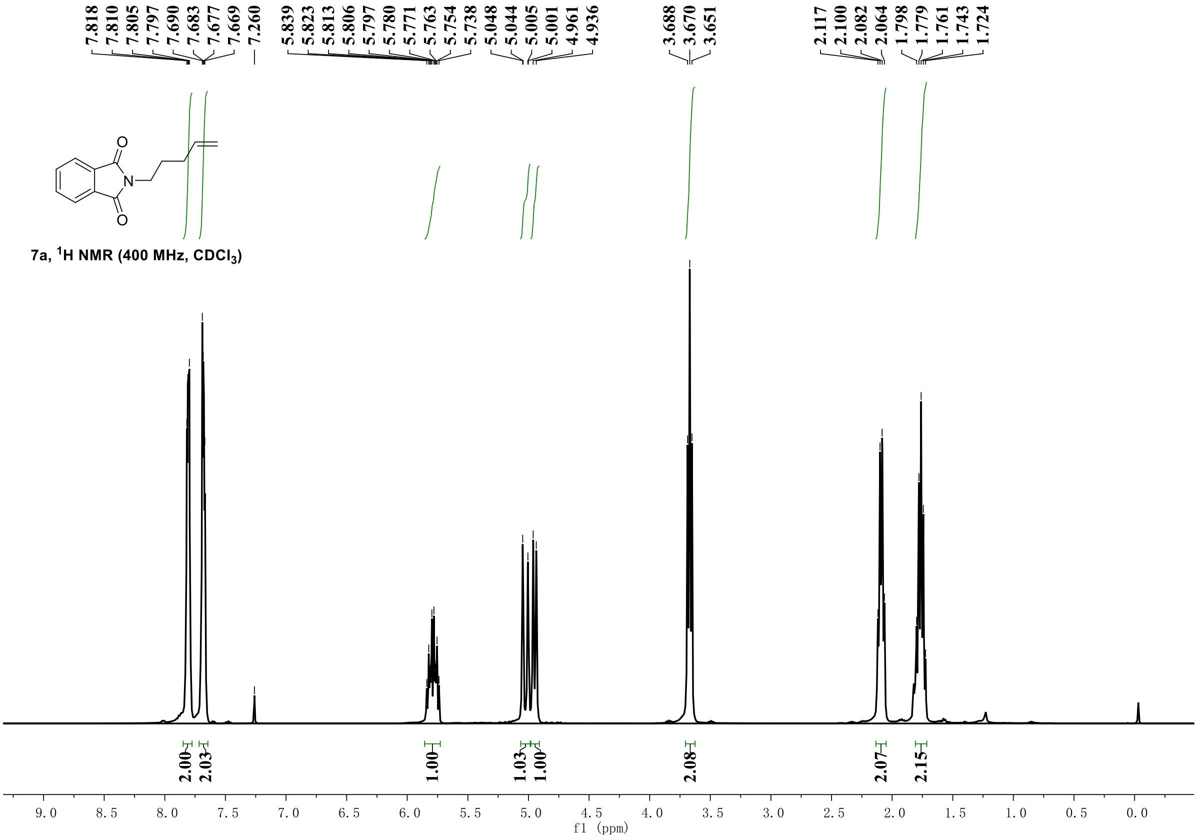

7a, ^{1}H NMR (400 MHz, $CDCl_3$)

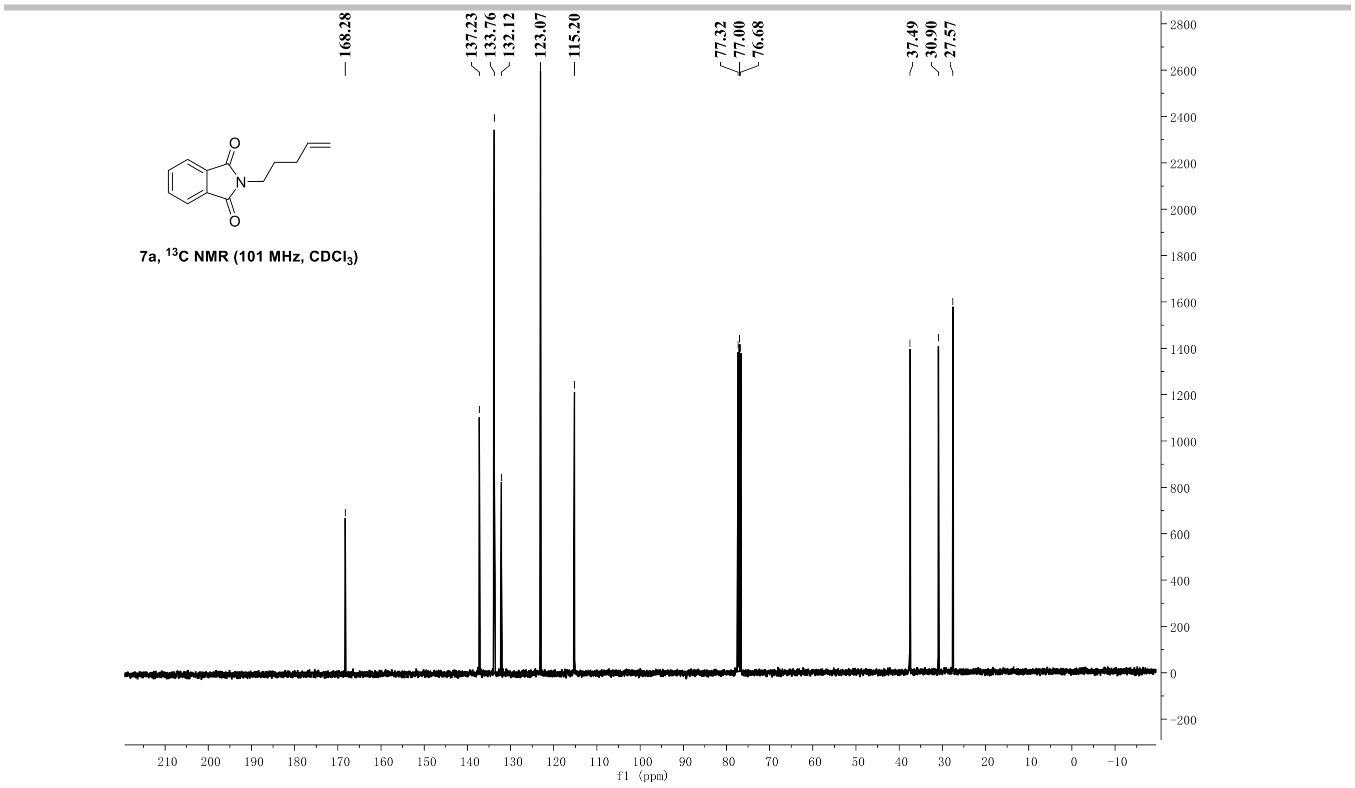
168.28
137.23
133.76
132.12
123.07
115.20
77.32
77.00
76.68
37.49
30.90
27.57
O
N
O
7a, ^{13}C NMR (101 MHz, CDCl$_3$)
f1 (ppm)

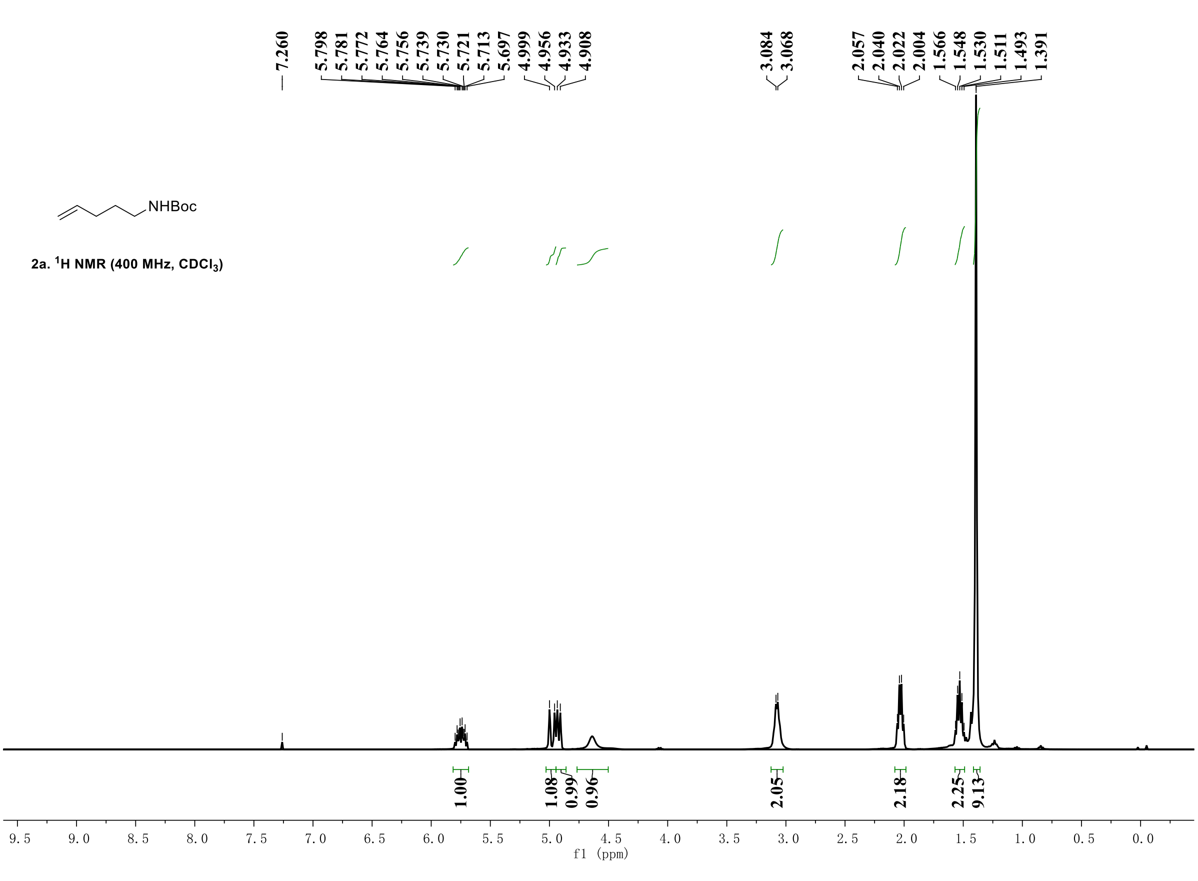
7.260
5.798
5.781
5.772
5.764
5.756
5.739
5.730
5.721
5.713
5.697
4.999
4.956
4.933
4.908
3.084
3.068
2.057
2.040
2.022
2.004
1.566
1.548
1.530
1.511
1.493
1.391
NHBoc
2a. ^{1}H NMR (400 MHz, CDCl$_3$)
1.00
1.08
0.99
0.96
2.05
2.18
2.25
9.13
f1 (ppm)

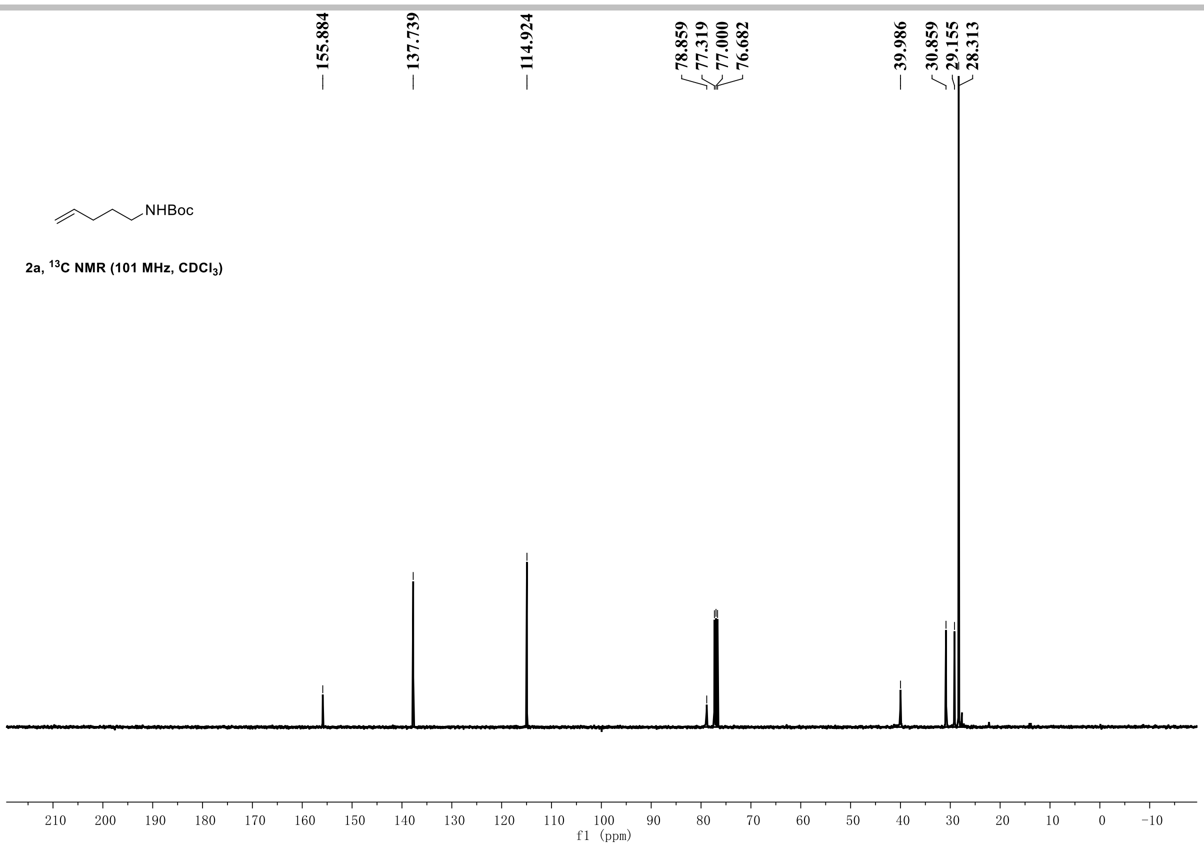
155.884
137.739
114.924
78.859
77.319
77.000
76.682
39.986
30.859
29.155
28.313
NHBoc
2a, 13C NMR (101 MHz, CDCl3)
210 200 190 180 170 160 150 140 130 120 110 100 90 80 70 60 50 40 30 20 10 0 -10
f1 (ppm)

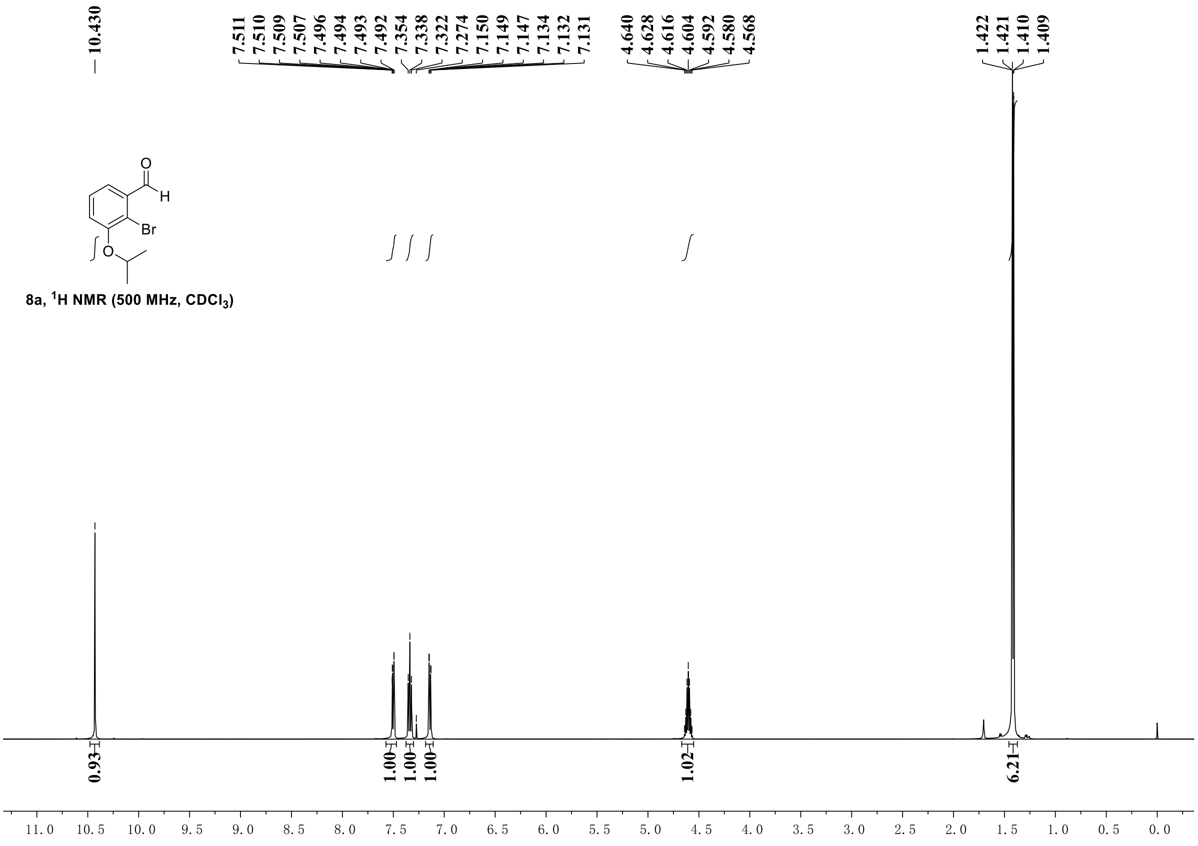
10.430
7.511
7.510
7.509
7.507
7.496
7.494
7.493
7.492
7.354
7.338
7.322
7.274
7.150
7.149
7.147
7.134
7.132
7.131
4.640
4.628
4.616
4.604
4.592
4.580
4.568
1.422
1.421
1.410
1.409
O
H
Br
O
8a, 1H NMR (500 MHz, CDCl3)
0.93
1.00
1.00
1.00
1.02
6.21
11.0 10.5 10.0 9.5 9.0 8.5 8.0 7.5 7.0 6.5 6.0 5.5 5.0 4.5 4.0 3.5 3.0 2.5 2.0 1.5 1.0 0.5 0.0

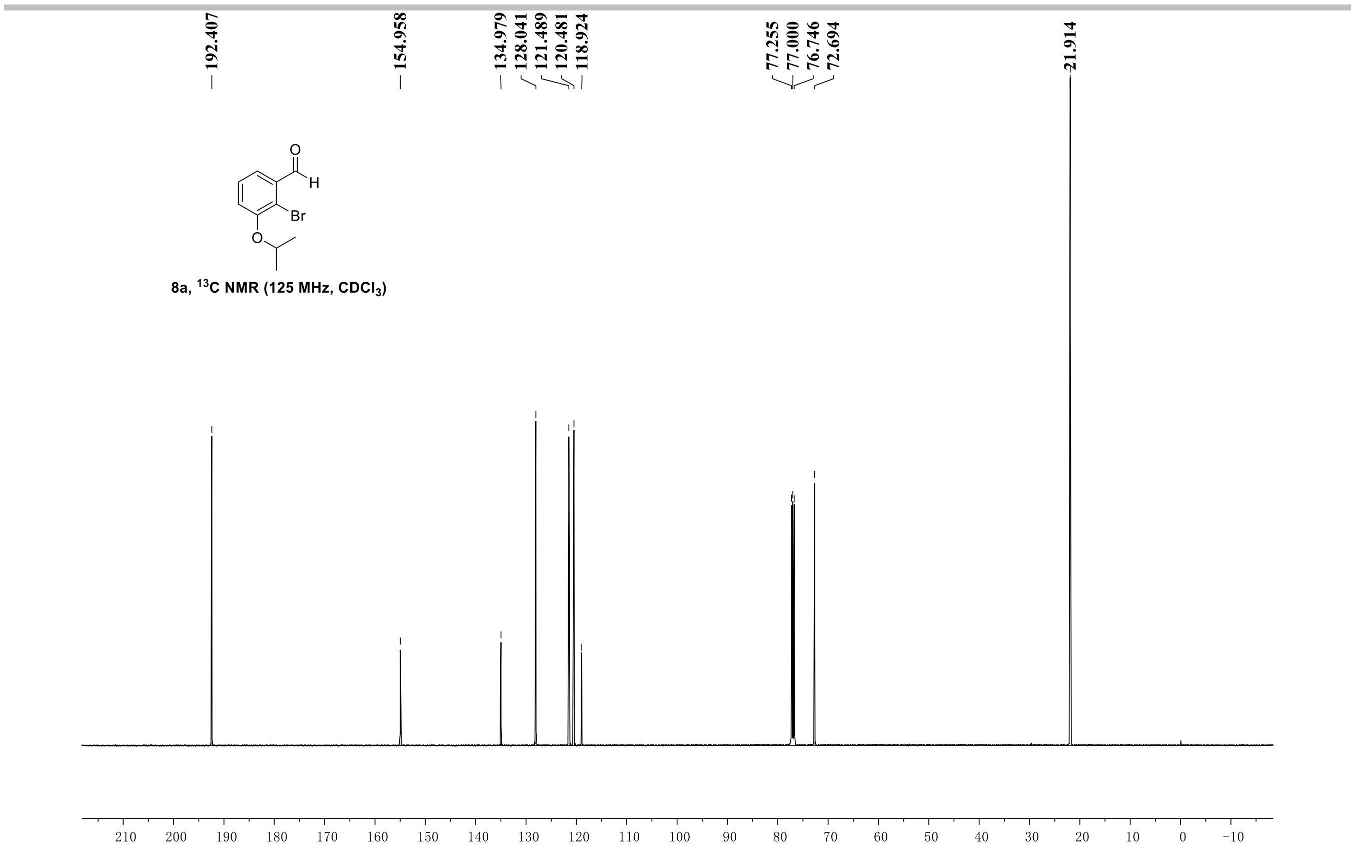
8a, ^{13}C NMR (125 MHz, CDCl$_3$)
192.407
154.958
134.979
128.041
121.489
120.481
118.924
77.255
77.000
76.746
72.694
21.914
210 200 190 180 170 160 150 140 130 120 110 100 90 80 70 60 50 40 30 20 10 0 −10

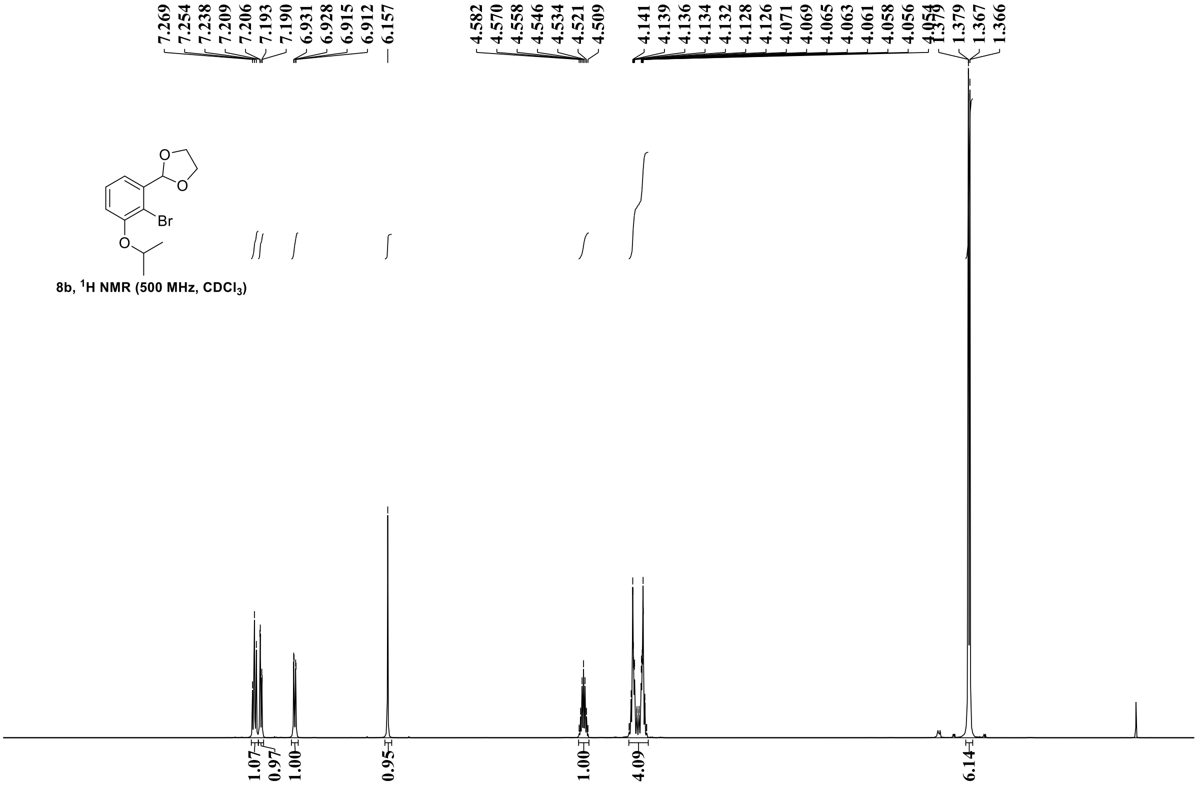
8b, ^{1}H NMR (500 MHz, CDCl$_3$)
7.269 7.254 7.238 7.209 7.206 7.193 7.190 6.931 6.928 6.915 6.912 6.157
4.582 4.570 4.558 4.546 4.534 4.521 4.509
4.141 4.139 4.136 4.134 4.132 4.128 4.126 4.071 4.069 4.065 4.063 4.061 4.058 4.056 4.054 1.379 1.367 1.366
1.07 0.97 1.00 0.95 1.00 4.09 6.14
9.0 8.5 8.0 7.5 7.0 6.5 6.0 5.5 5.0 4.5 4.0 3.5 3.0 2.5 2.0 1.5 1.0 0.5 0.0

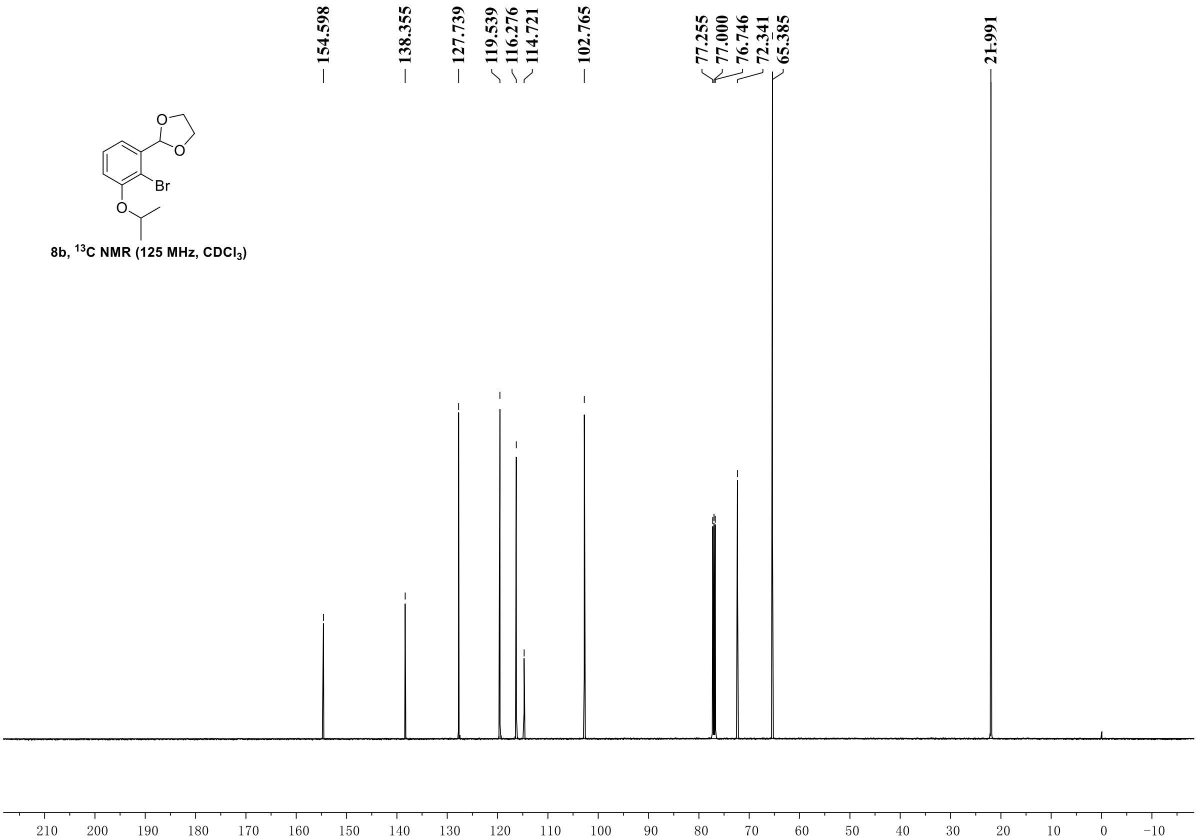

8b, ^{13}C NMR (125 MHz, $CDCl_3$)

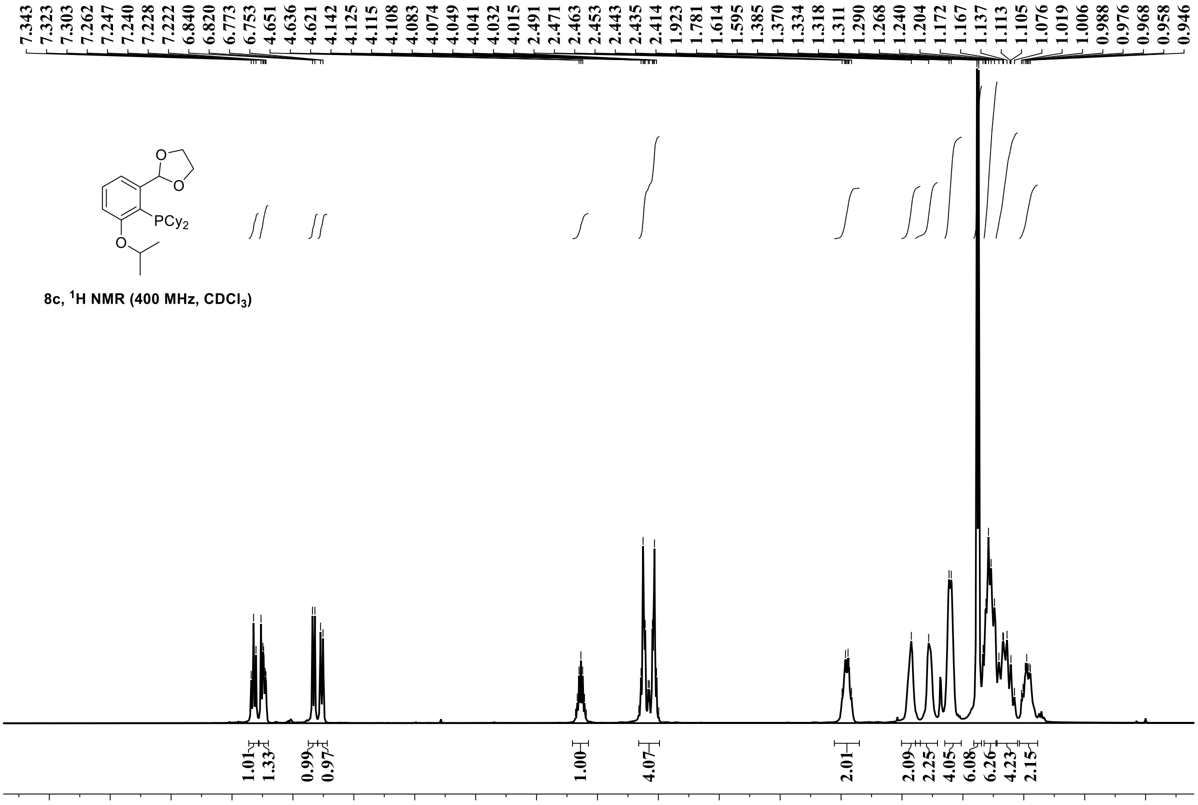

8c, ^1H NMR (400 MHz, $CDCl_3$)

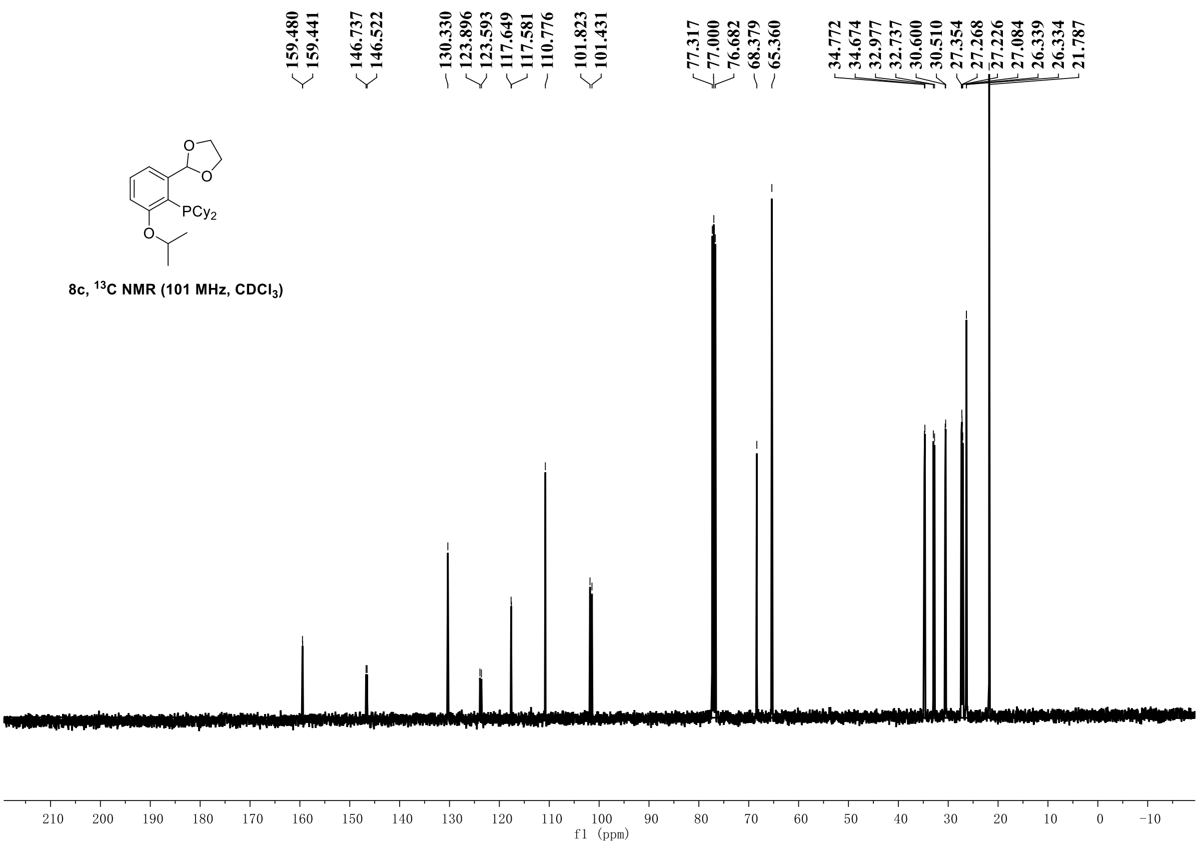

8c, ^{13}C NMR (101 MHz, $CDCl_3$)

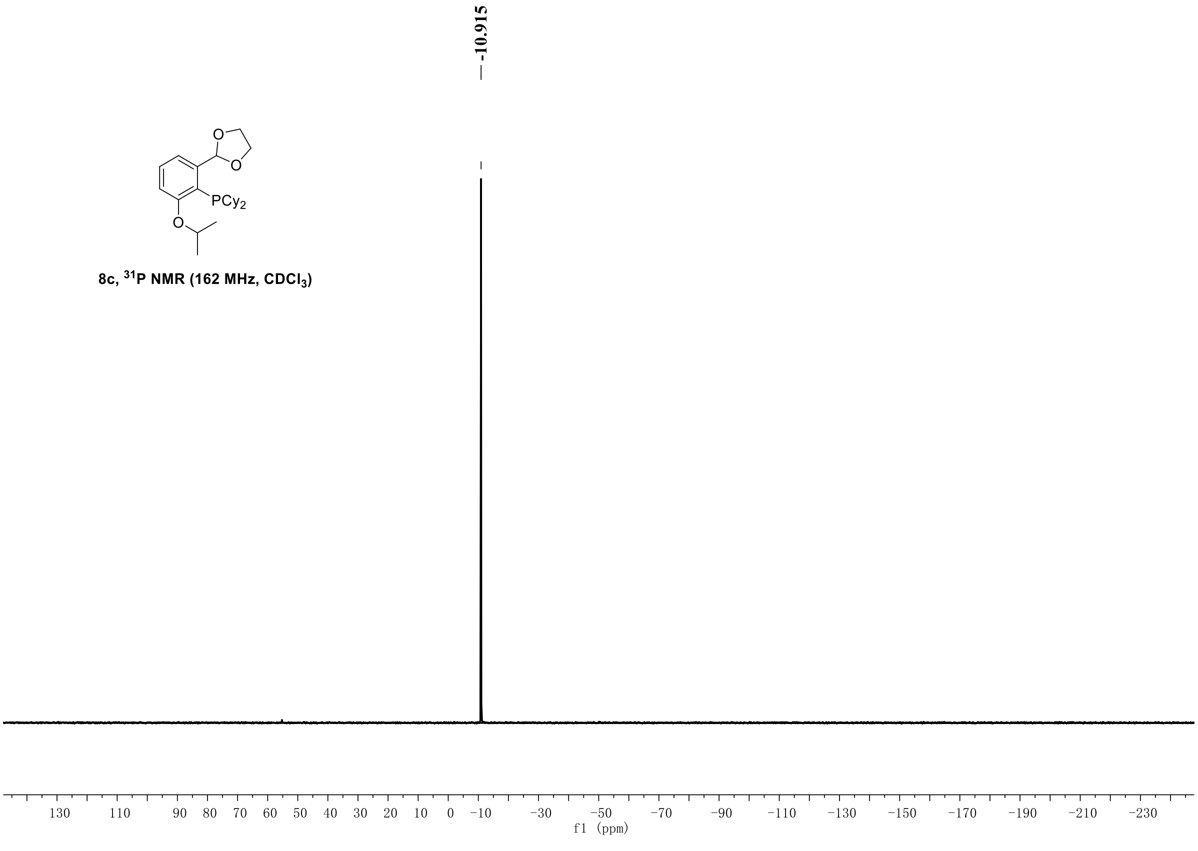

8c, ^{31}P NMR (162 MHz, $CDCl_3$)

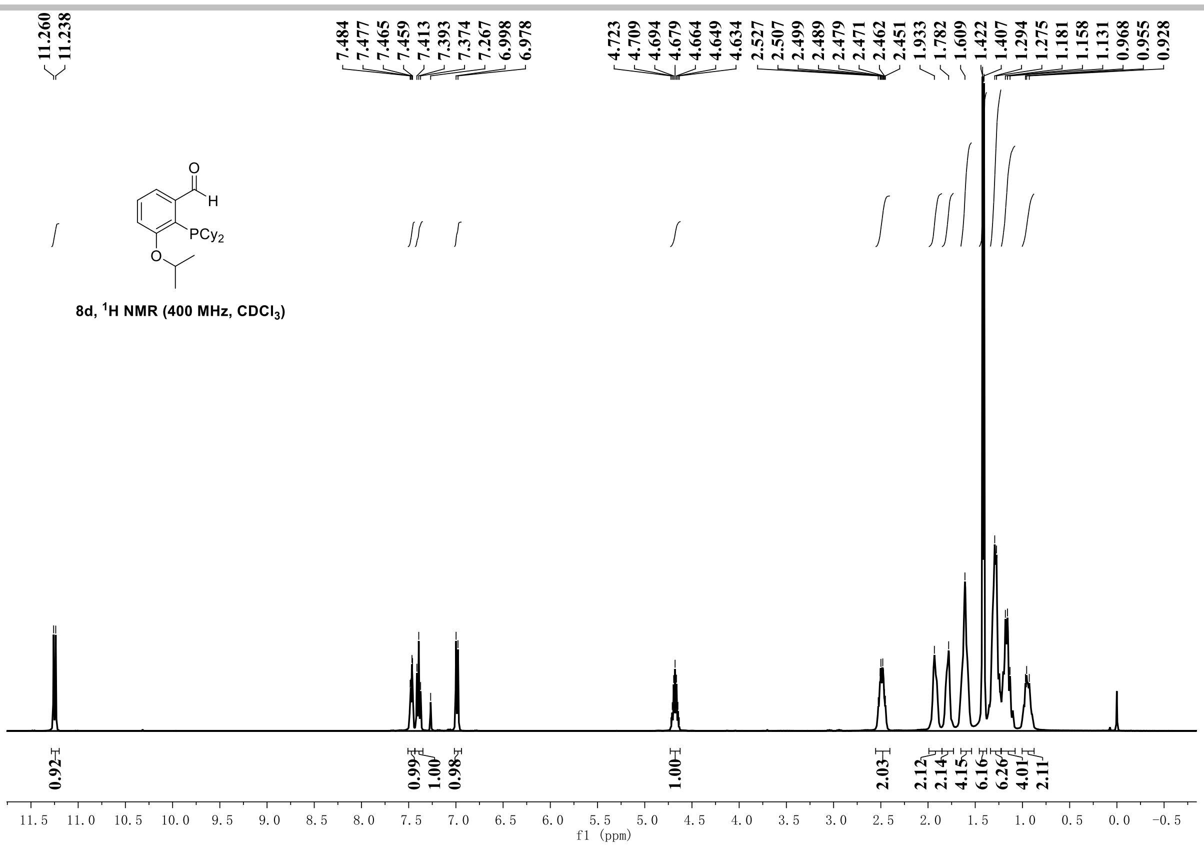

8d, ^{1}H NMR (400 MHz, $CDCl_3$)

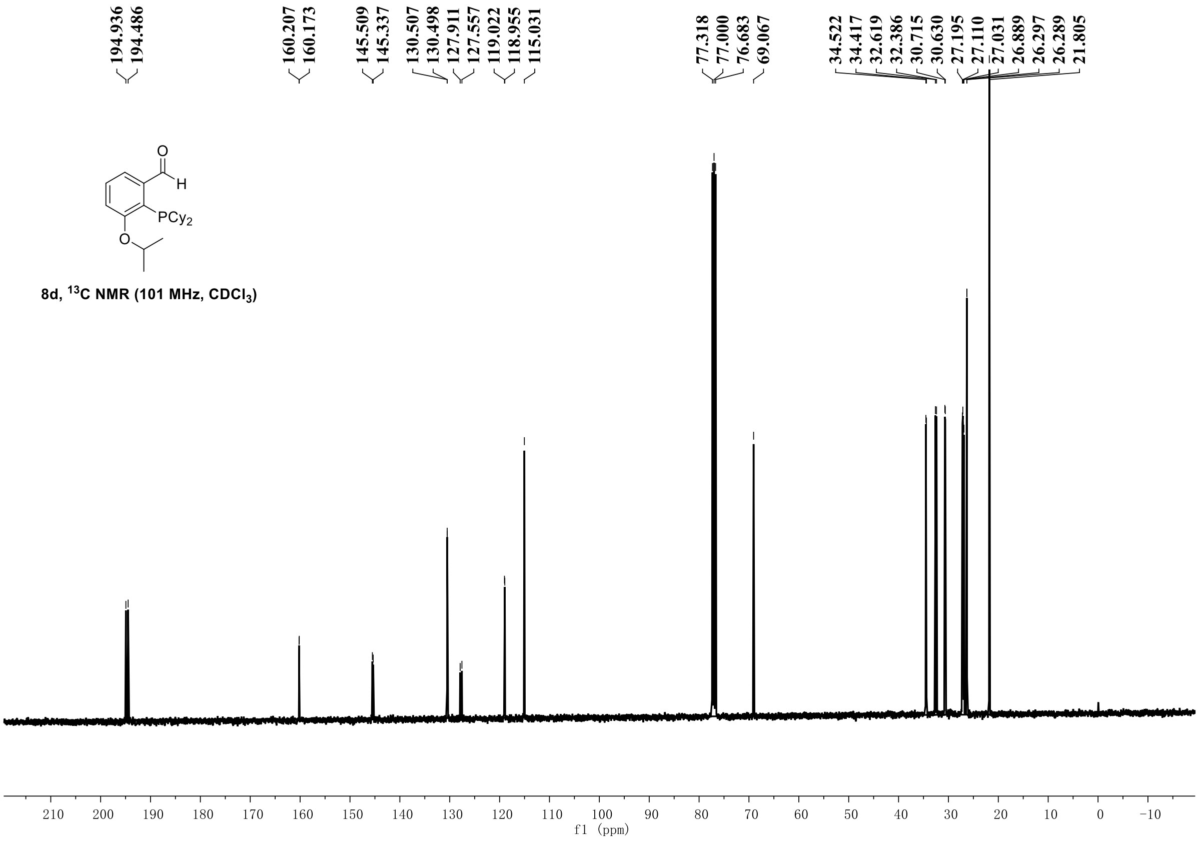

8d, ^{13}C NMR (101 MHz, $CDCl_3$)

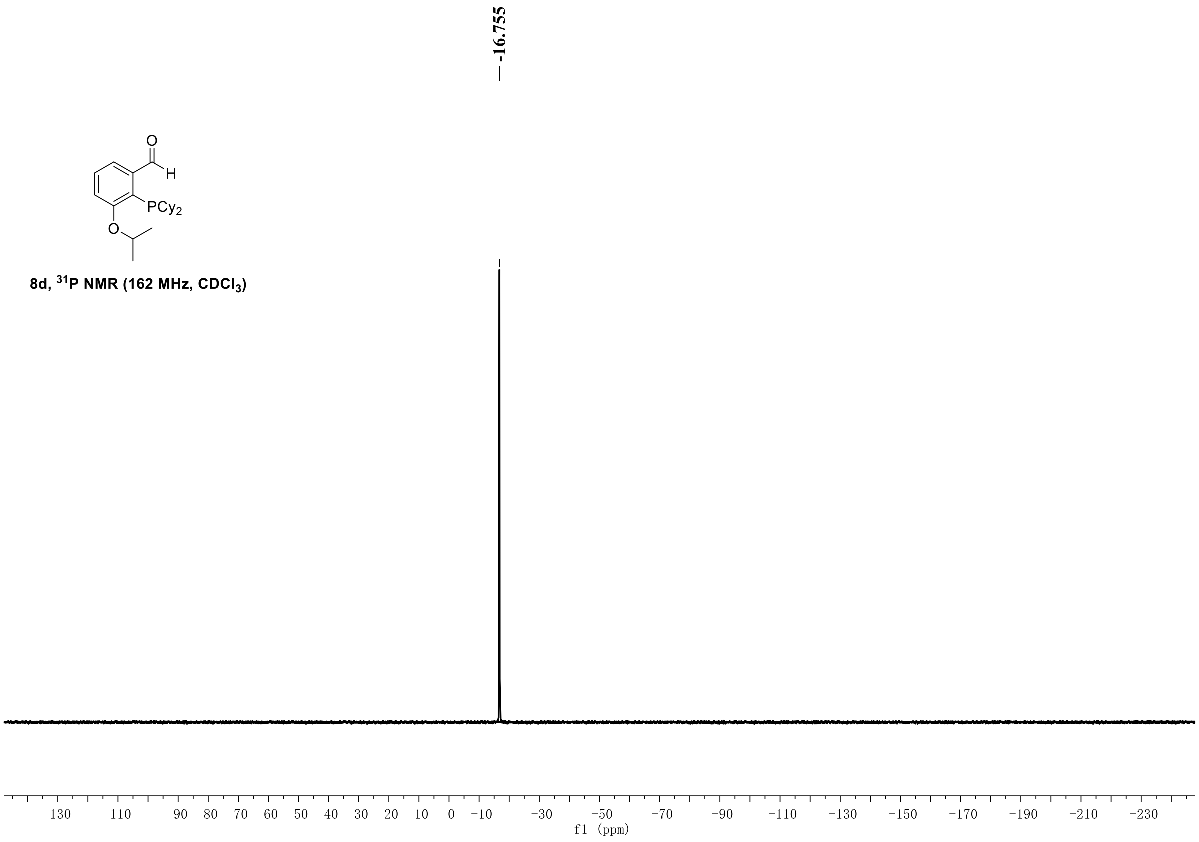
-16.755
O
H
PCy2
O
8d, 31P NMR (162 MHz, CDCl3)
130 110 90 80 70 60 50 40 30 20 10 0 −10 −30 −50 −70 −90 −110 −130 −150 −170 −190 −210 −230
f1 (ppm)

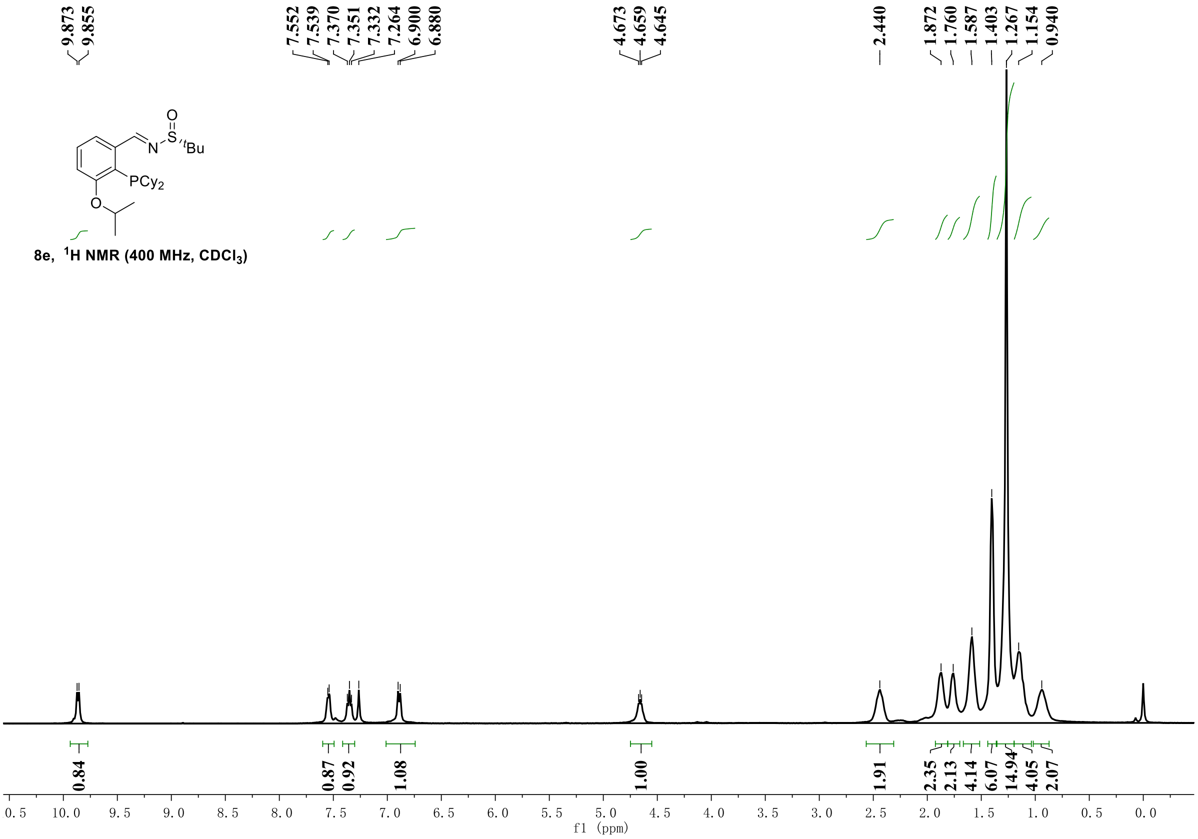
9.873
9.855
7.552
7.539
7.370
7.351
7.332
7.264
6.900
6.880
4.673
4.659
4.645
2.440
1.872
1.760
1.587
1.403
1.267
1.154
0.940
O
S
N
tBu
PCy2
O
8e, 1H NMR (400 MHz, CDCl3)
0.84
0.87
0.92
1.08
1.00
1.91
2.35
2.13
4.14
6.07
14.94
4.05
2.07
0.5 10.0 9.5 9.0 8.5 8.0 7.5 7.0 6.5 6.0 5.5 5.0 4.5 4.0 3.5 3.0 2.5 2.0 1.5 1.0 0.5 0.0
f1 (ppm)

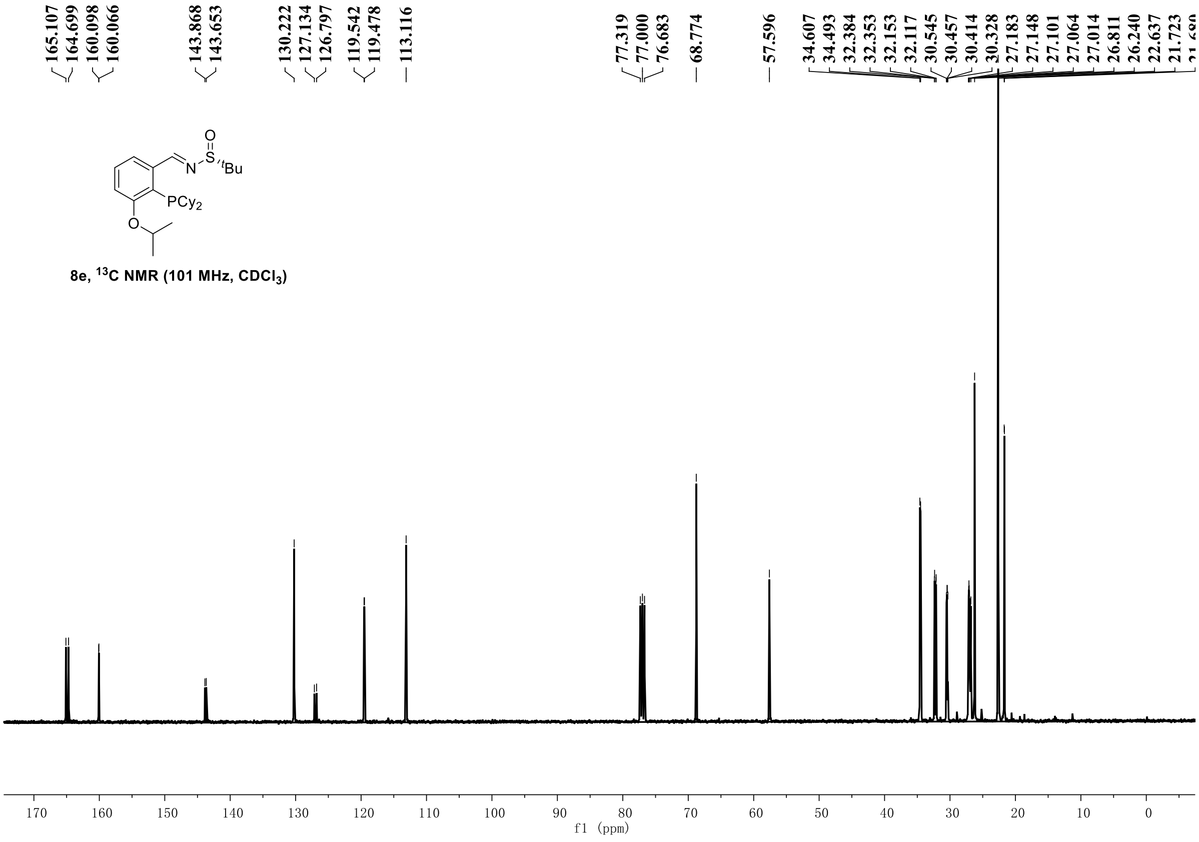
8e, 13C NMR (101 MHz, CDCl3)
f1 (ppm)

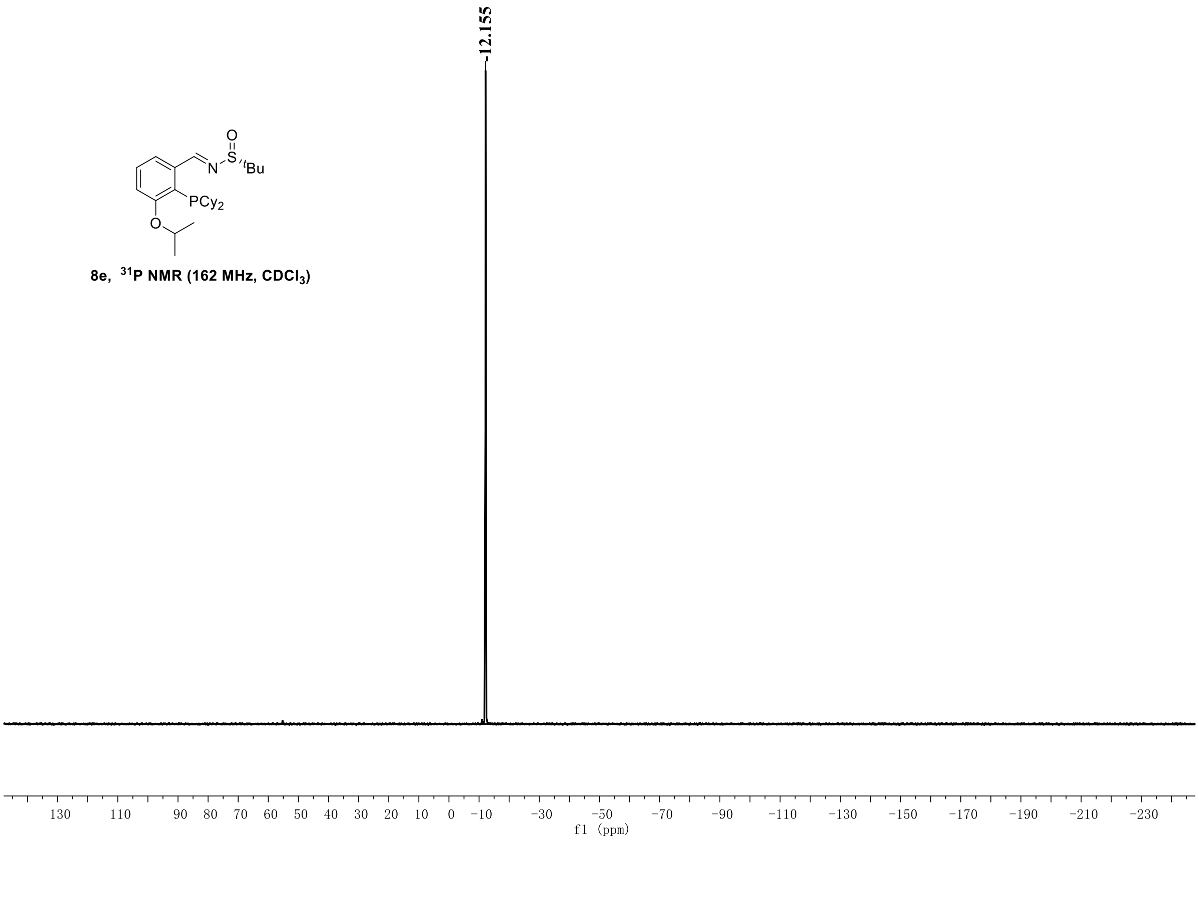
-12.155
8e, 31P NMR (162 MHz, CDCl3)
f1 (ppm)

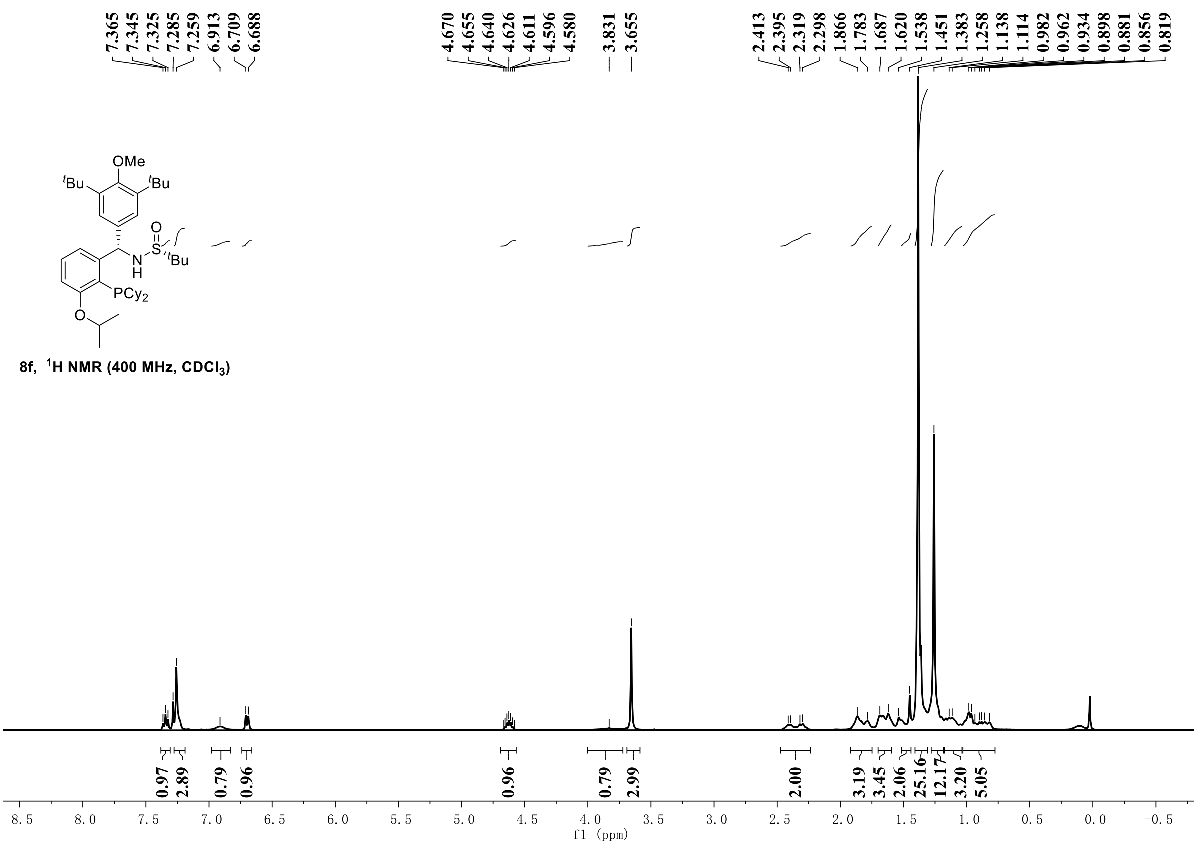

8f, ^{1}H NMR (400 MHz, CDCl$_3$)

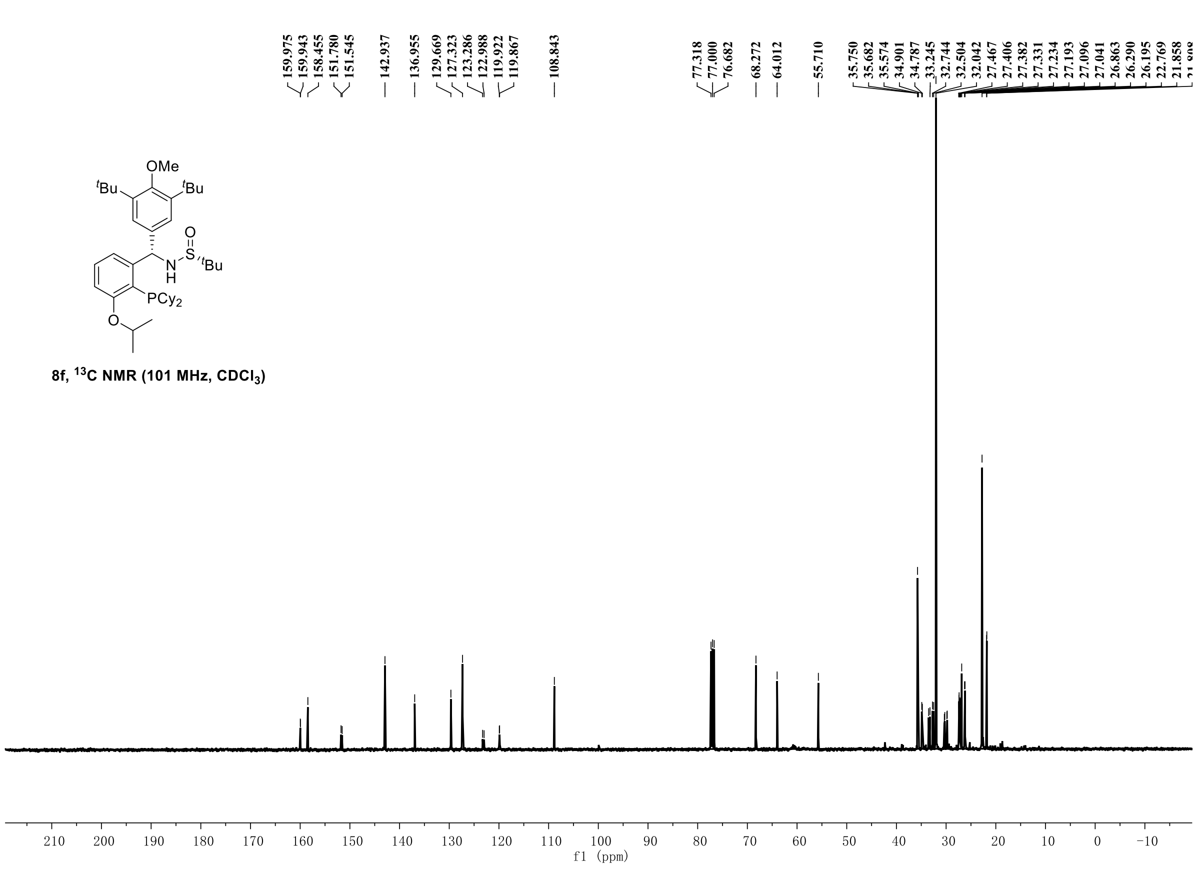

8f, ^{13}C NMR (101 MHz, CDCl$_3$)

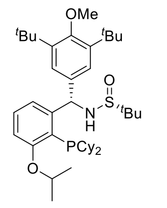

8f, ^{31}P NMR (162 MHz, $CDCl_3$)

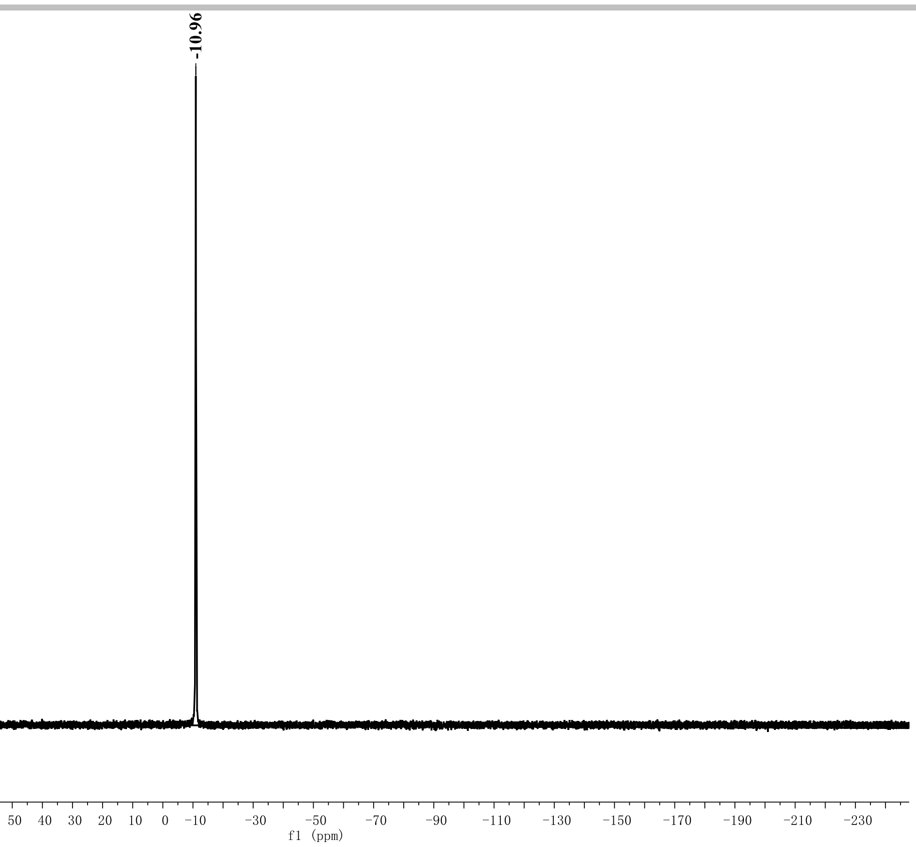

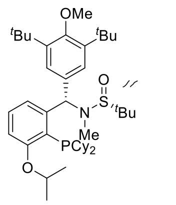

(S, Rs)-**Xu4**, ^{1}H NMR (400 MHz, $CDCl_3$)

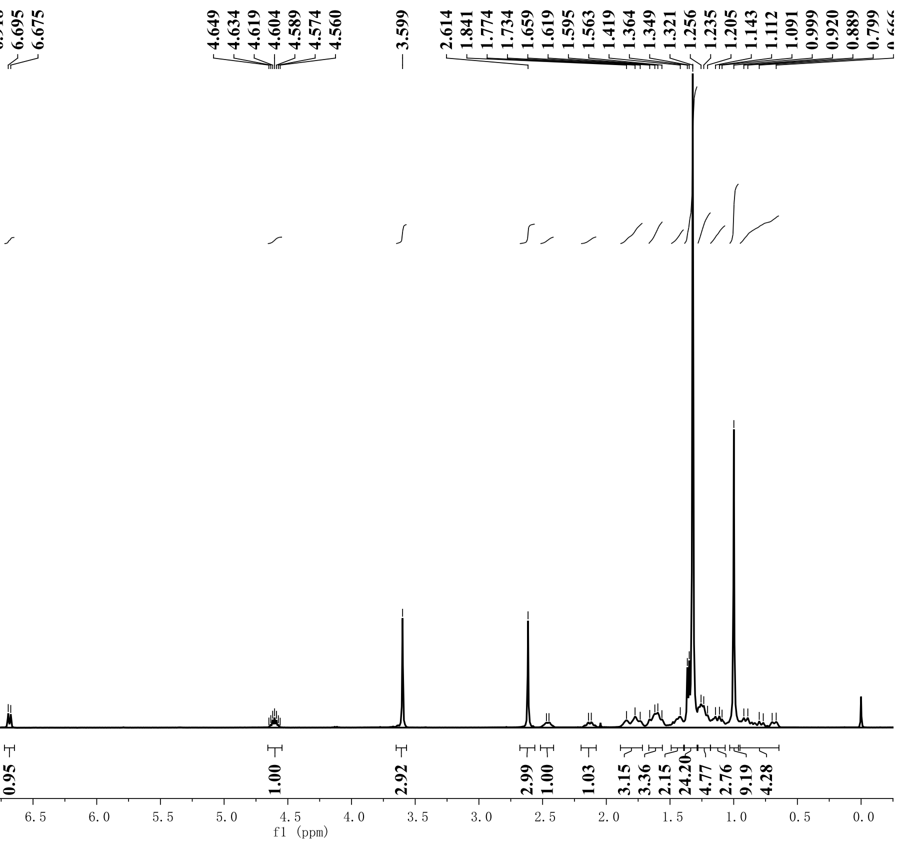

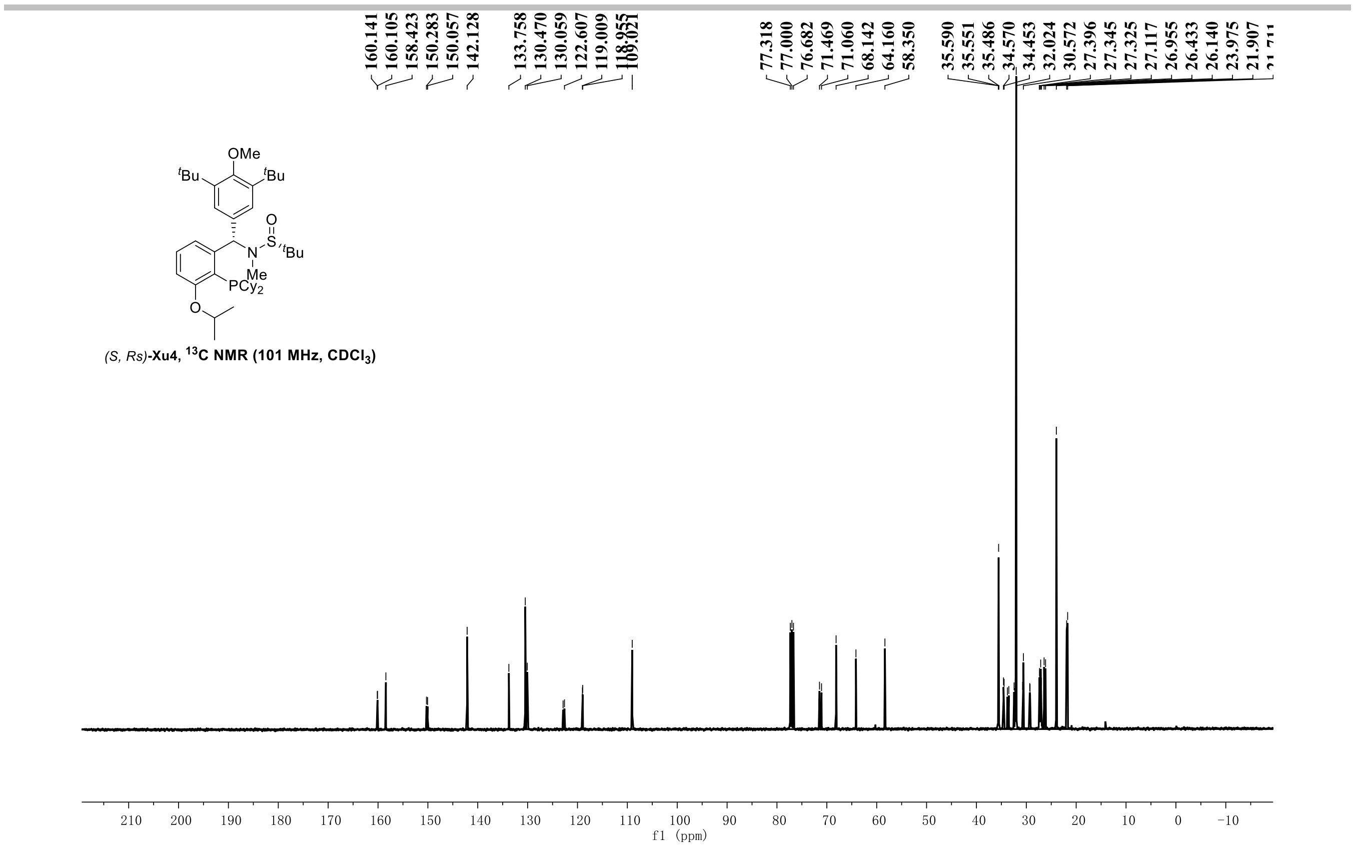

(S, Rs)-**Xu4, ^{13}C NMR (101 MHz, CDCl$_3$)**

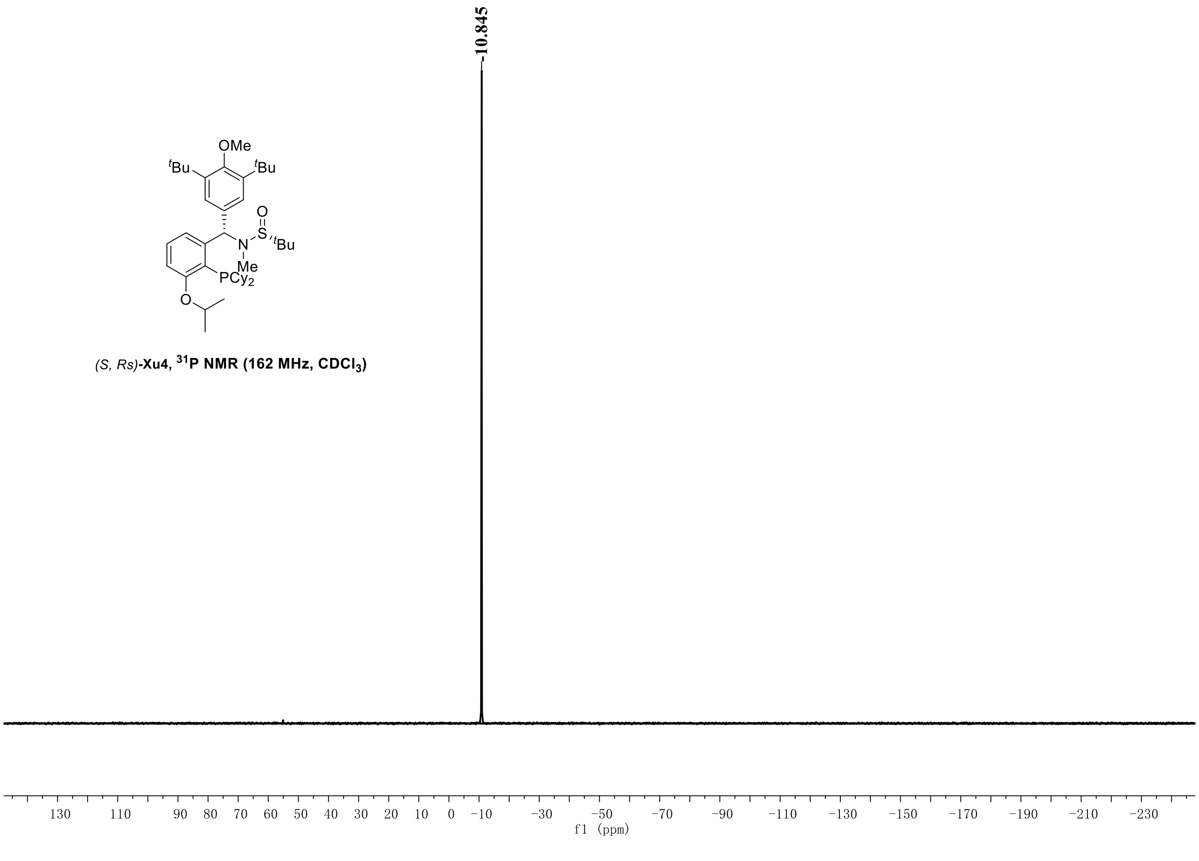

(S, Rs)-**Xu4, ^{31}P NMR (162 MHz, CDCl$_3$)**

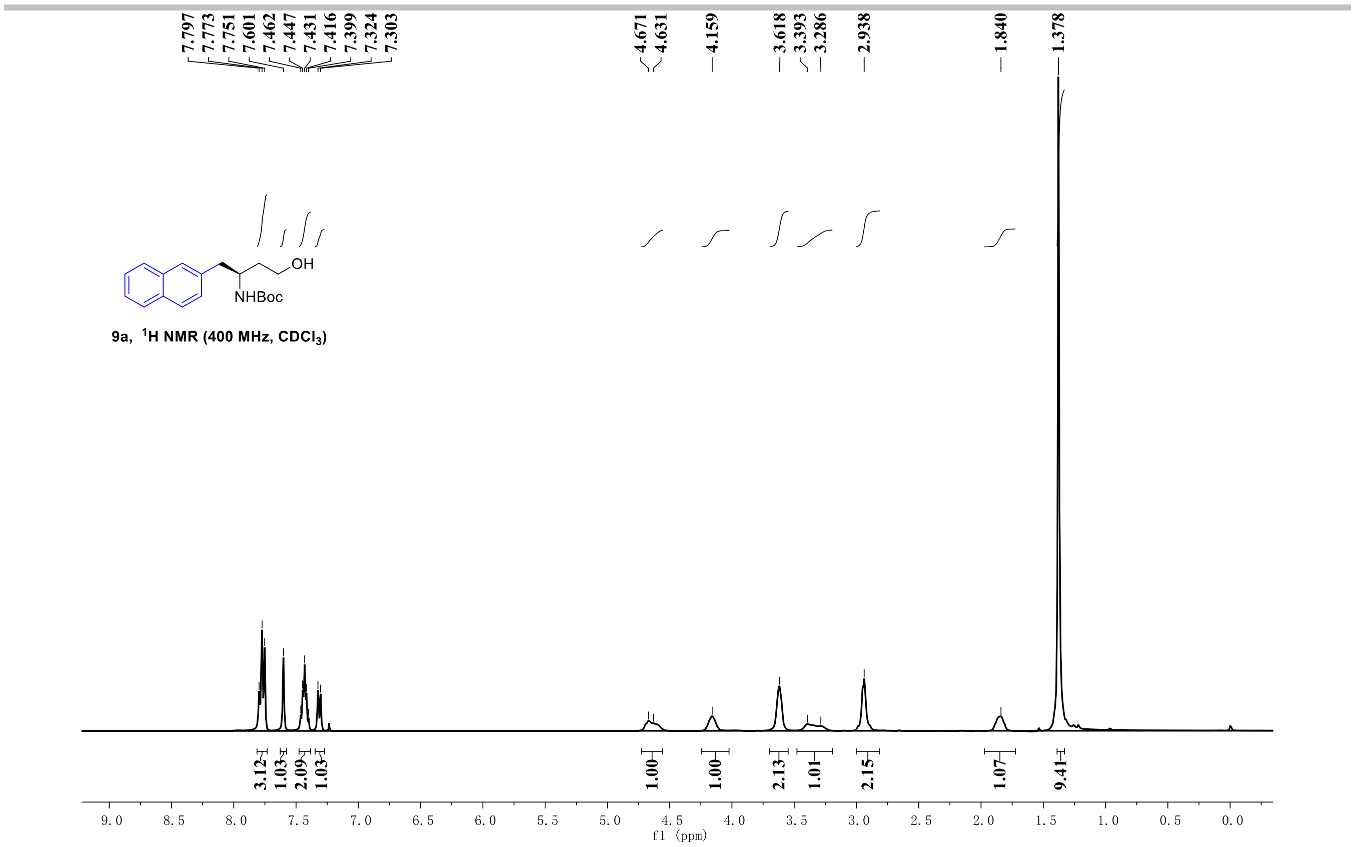

9a, ^{1}H NMR (400 MHz, $CDCl_3$)

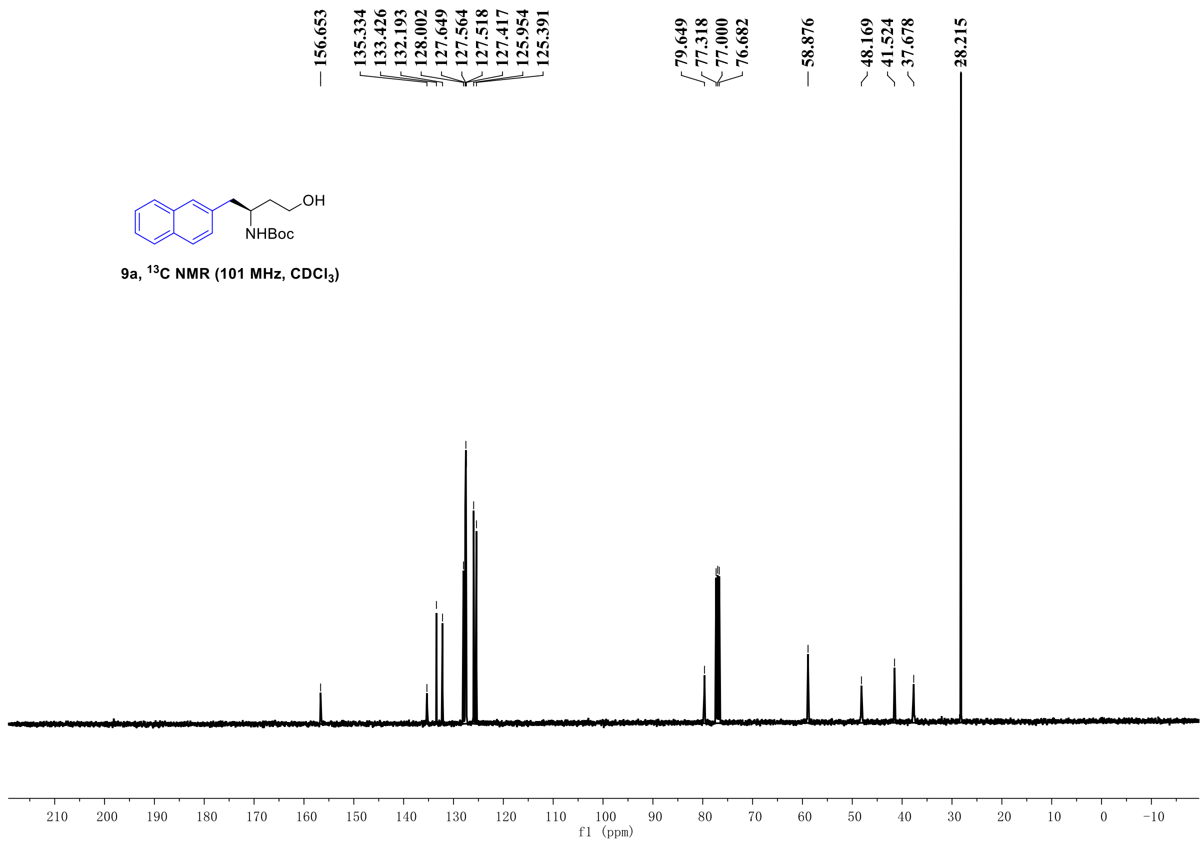

9a, ^{13}C NMR (101 MHz, $CDCl_3$)

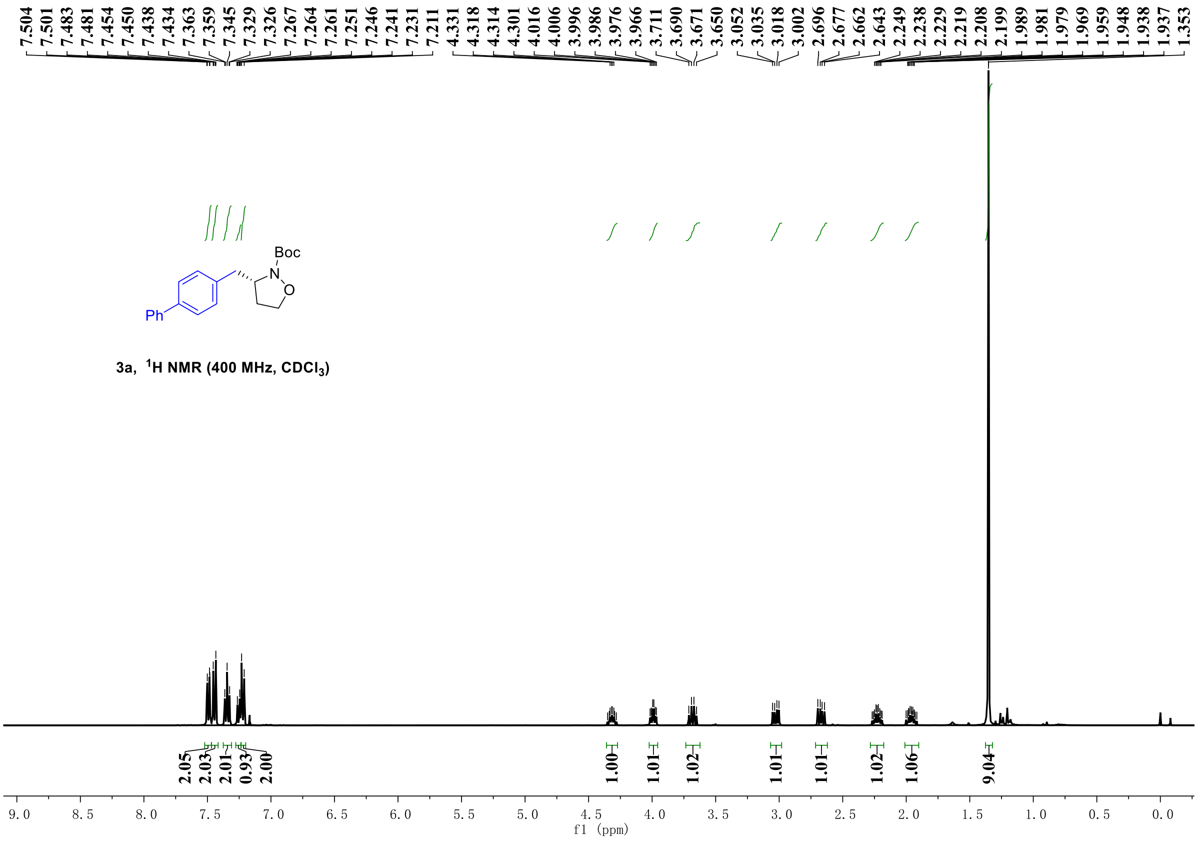
3a, 1H NMR (400 MHz, CDCl3)

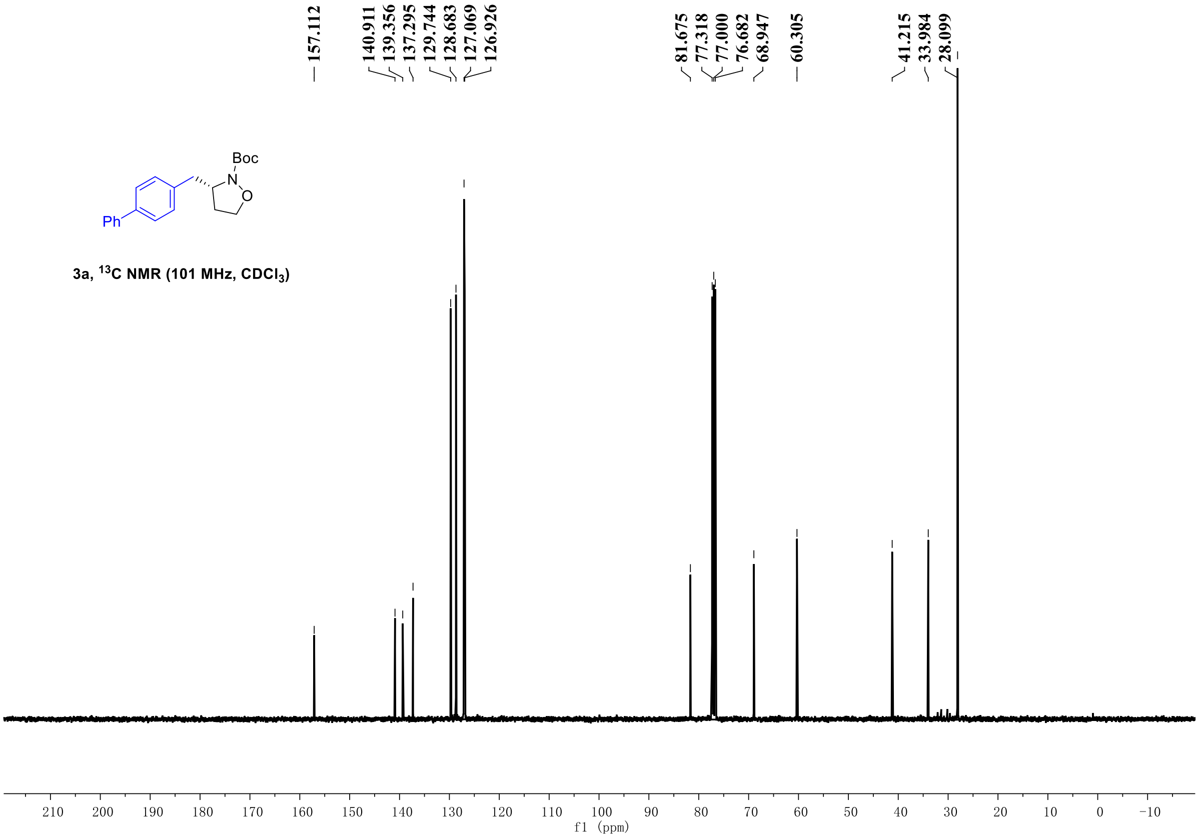
3a, 13C NMR (101 MHz, CDCl3)

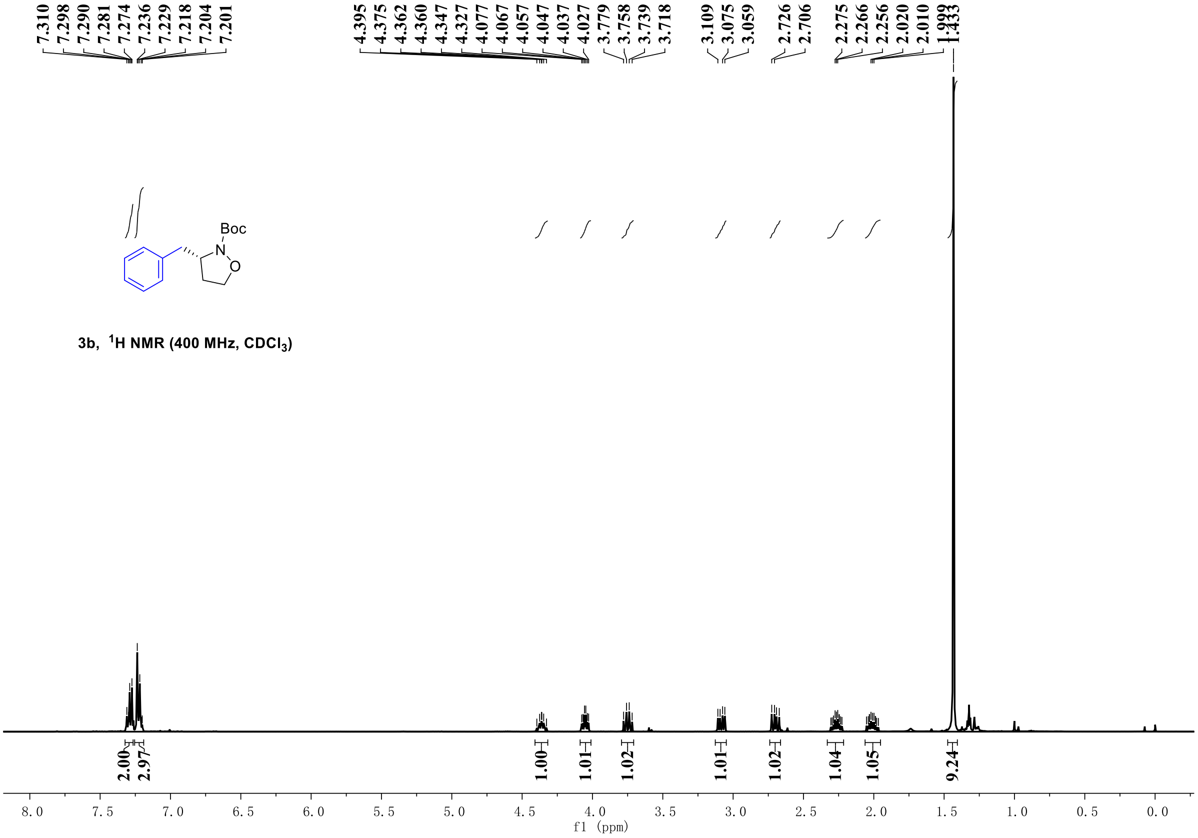
7.310
7.298
7.290
7.281
7.274
7.236
7.229
7.218
7.204
7.201
4.395
4.375
4.362
4.360
4.347
4.327
4.077
4.067
4.057
4.047
4.037
4.027
3.779
3.758
3.739
3.718
3.109
3.075
3.059
2.726
2.706
2.275
2.266
2.256
2.020
2.010
1.999
1.433
Boc
N
O
3b, 1H NMR (400 MHz, CDCl3)
2.00
2.97
1.00
1.01
1.02
1.01
1.02
1.04
1.05
9.24
f1 (ppm)

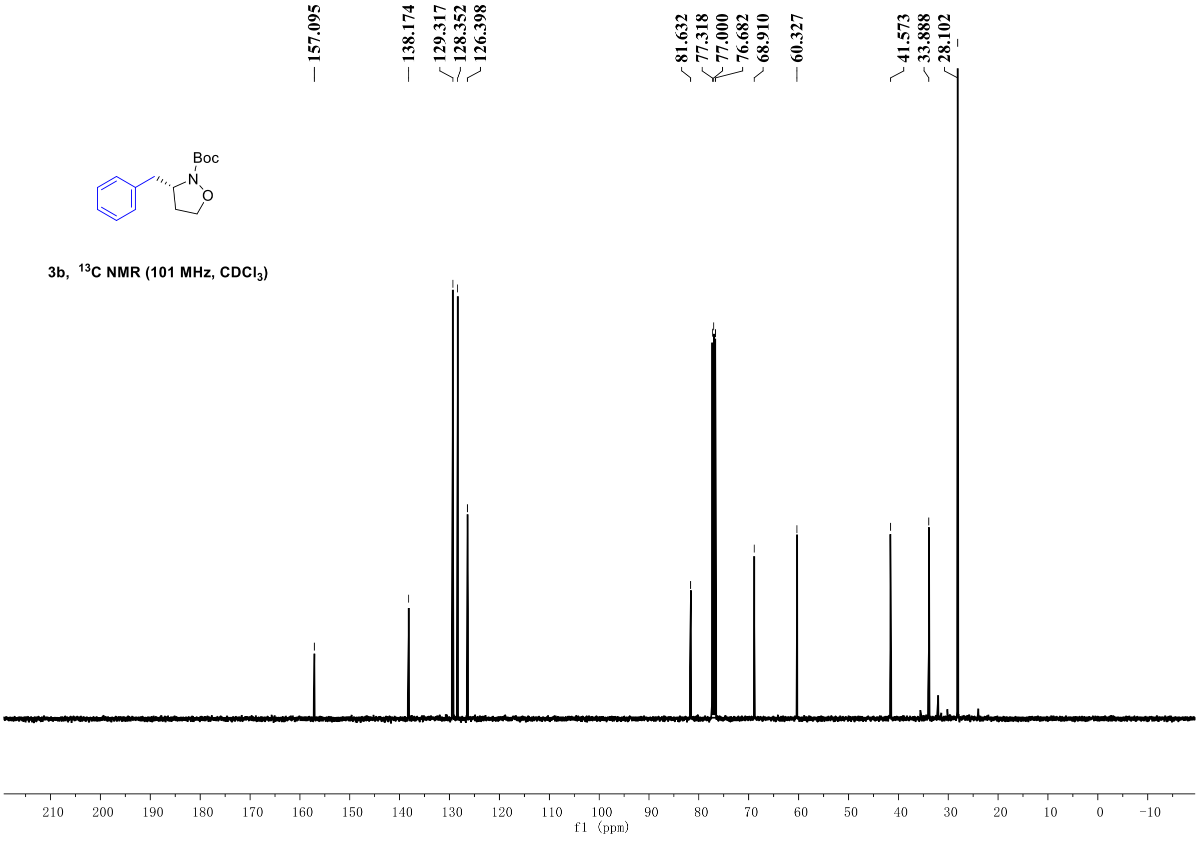
157.095
138.174
129.317
128.352
126.398
81.632
77.318
77.000
76.682
68.910
60.327
41.573
33.888
28.102
Boc
N
O
3b, 13C NMR (101 MHz, CDCl3)
f1 (ppm)

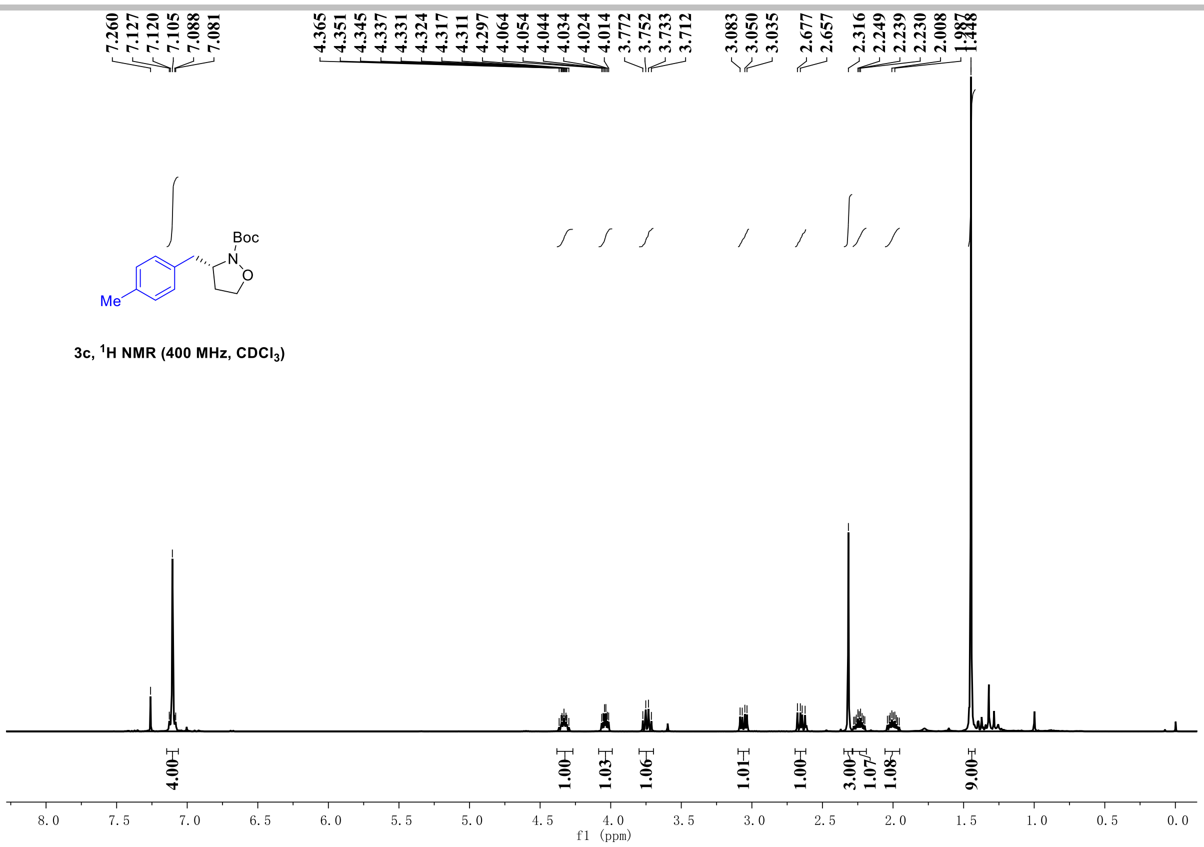

3c, ^{1}H NMR (400 MHz, $CDCl_3$)

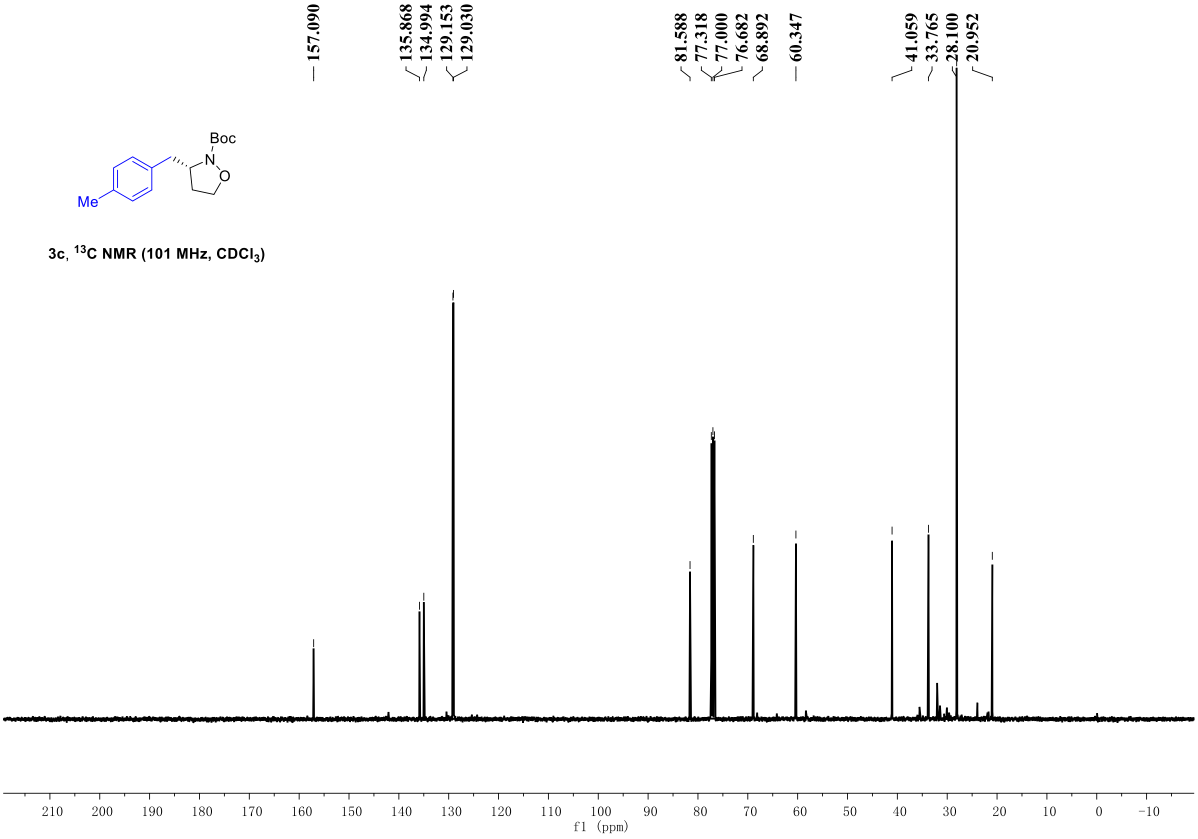

3c, ^{13}C NMR (101 MHz, $CDCl_3$)

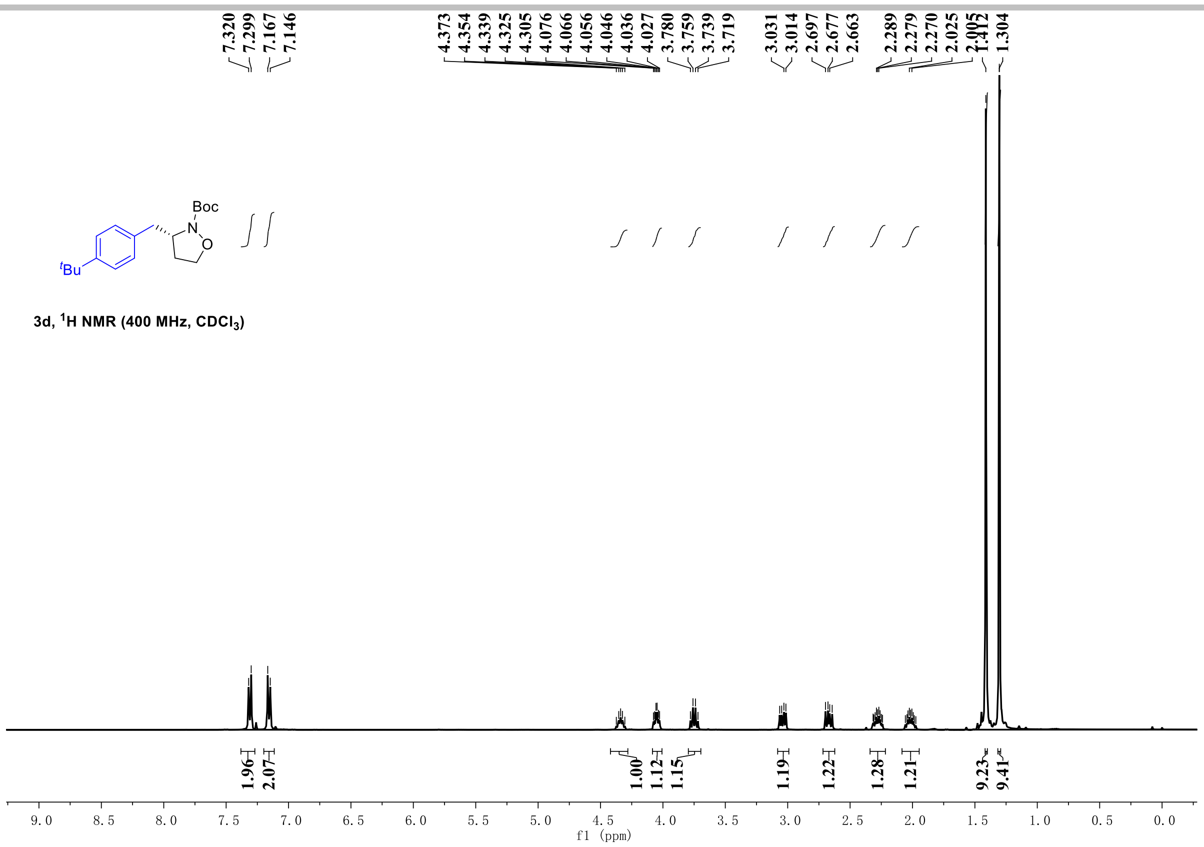

7.320
7.299
7.167
7.146
4.373
4.354
4.339
4.325
4.305
4.076
4.066
4.056
4.046
4.036
4.027
3.780
3.759
3.739
3.719
3.031
3.014
2.697
2.677
2.663
2.289
2.279
2.270
2.025
1.304
Boc
N
O
tBu
3d, 1H NMR (400 MHz, CDCl3)
1.96
2.07
1.00
1.12
1.15
1.19
1.22
1.28
1.21
9.23
9.41
9.0 8.5 8.0 7.5 7.0 6.5 6.0 5.5 5.0 4.5 4.0 3.5 3.0 2.5 2.0 1.5 1.0 0.5 0.0
f1 (ppm)

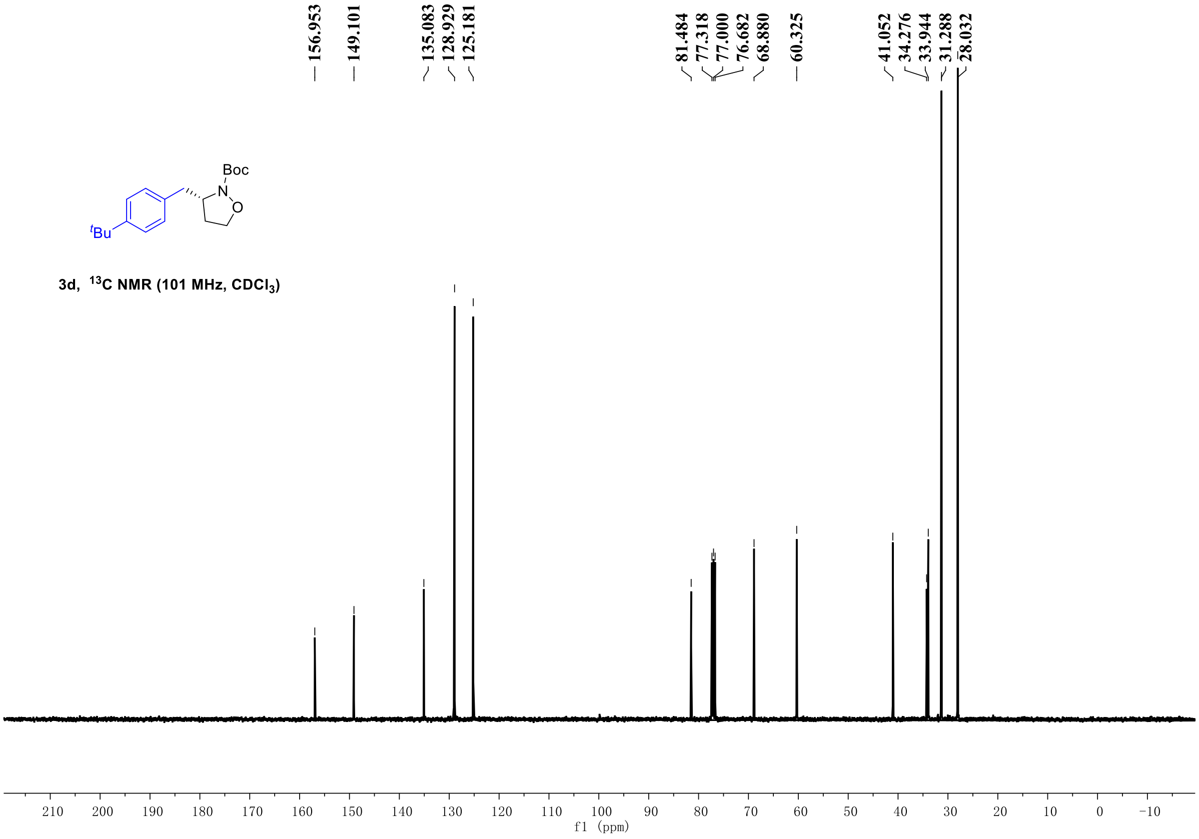

156.953
149.101
135.083
128.929
125.181
81.484
77.318
77.000
76.682
68.880
60.325
41.052
34.276
33.944
31.288
28.032
Boc
N
O
tBu
3d, 13C NMR (101 MHz, CDCl3)
210 200 190 180 170 160 150 140 130 120 110 100 90 80 70 60 50 40 30 20 10 0 -10
f1 (ppm)

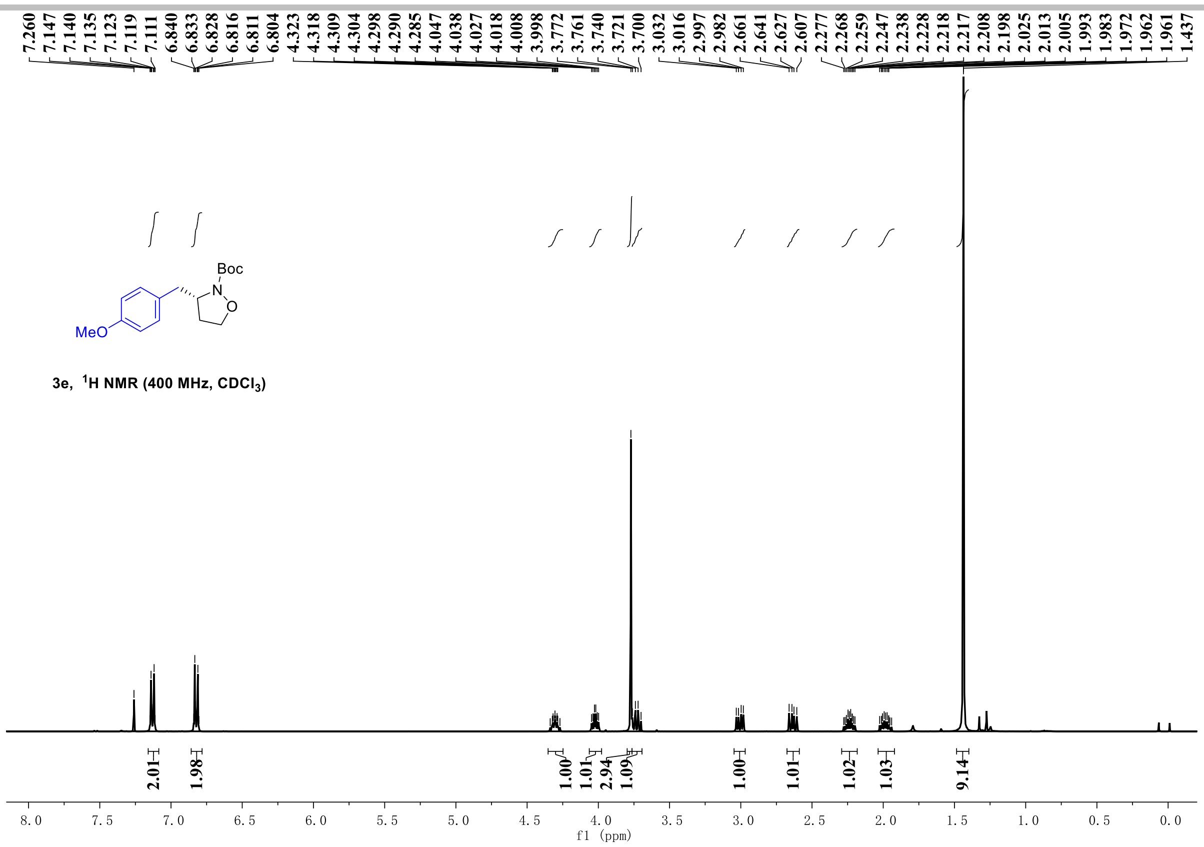
7.260
7.147
7.140
7.135
7.123
7.119
7.111
6.840
6.833
6.828
6.816
6.811
6.804
4.323
4.318
4.309
4.304
4.298
4.290
4.285
4.047
4.038
4.027
4.018
4.008
3.998
3.772
3.761
3.740
3.721
3.700
3.032
3.016
2.997
2.982
2.661
2.641
2.627
2.607
2.277
2.268
2.259
2.247
2.238
2.228
2.218
2.217
2.208
2.198
2.025
2.013
2.005
1.993
1.983
1.972
1.962
1.961
1.437
Boc
N
O
MeO
3e, 1H NMR (400 MHz, CDCl3)
2.01
1.98
1.00
1.01
2.94
1.09
1.00
1.01
1.02
1.03
9.14
f1 (ppm)

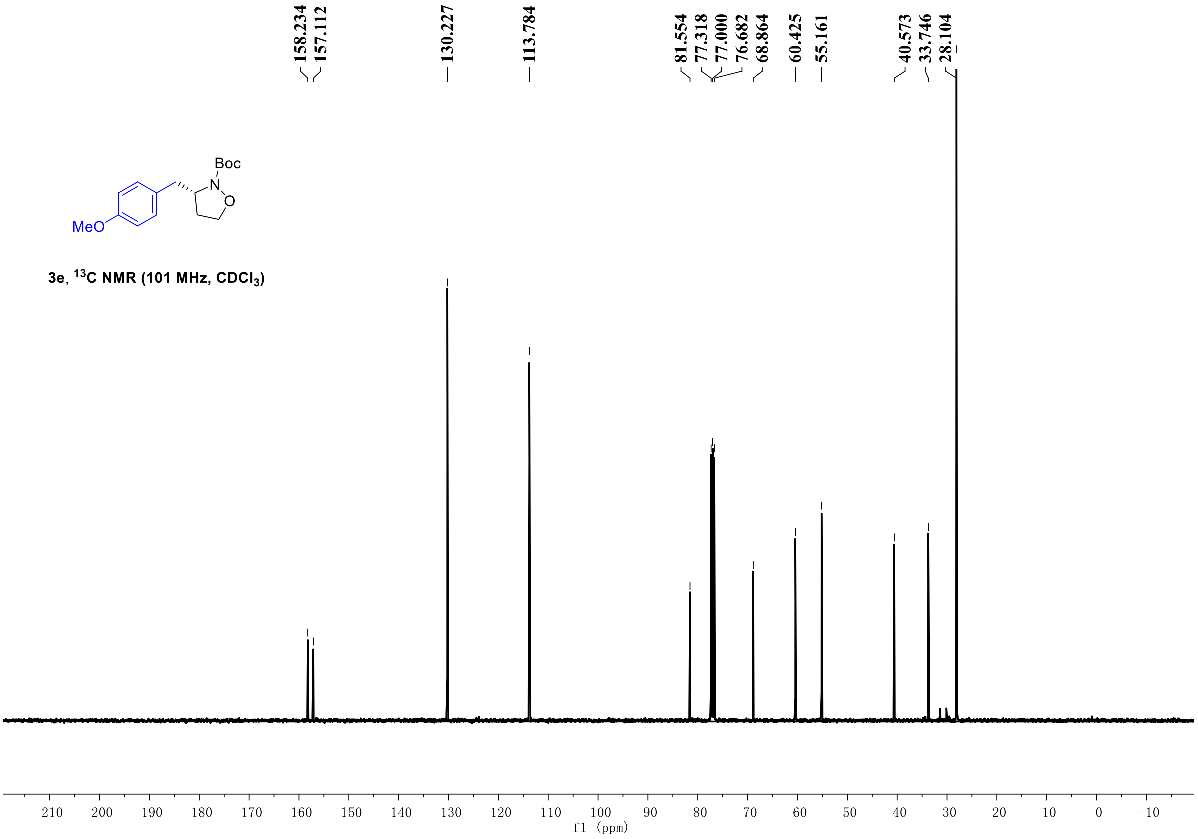
158.234
157.112
130.227
113.784
81.554
77.318
77.000
76.682
68.864
60.425
55.161
40.573
33.746
28.104
Boc
N
O
MeO
3e, 13C NMR (101 MHz, CDCl3)
f1 (ppm)

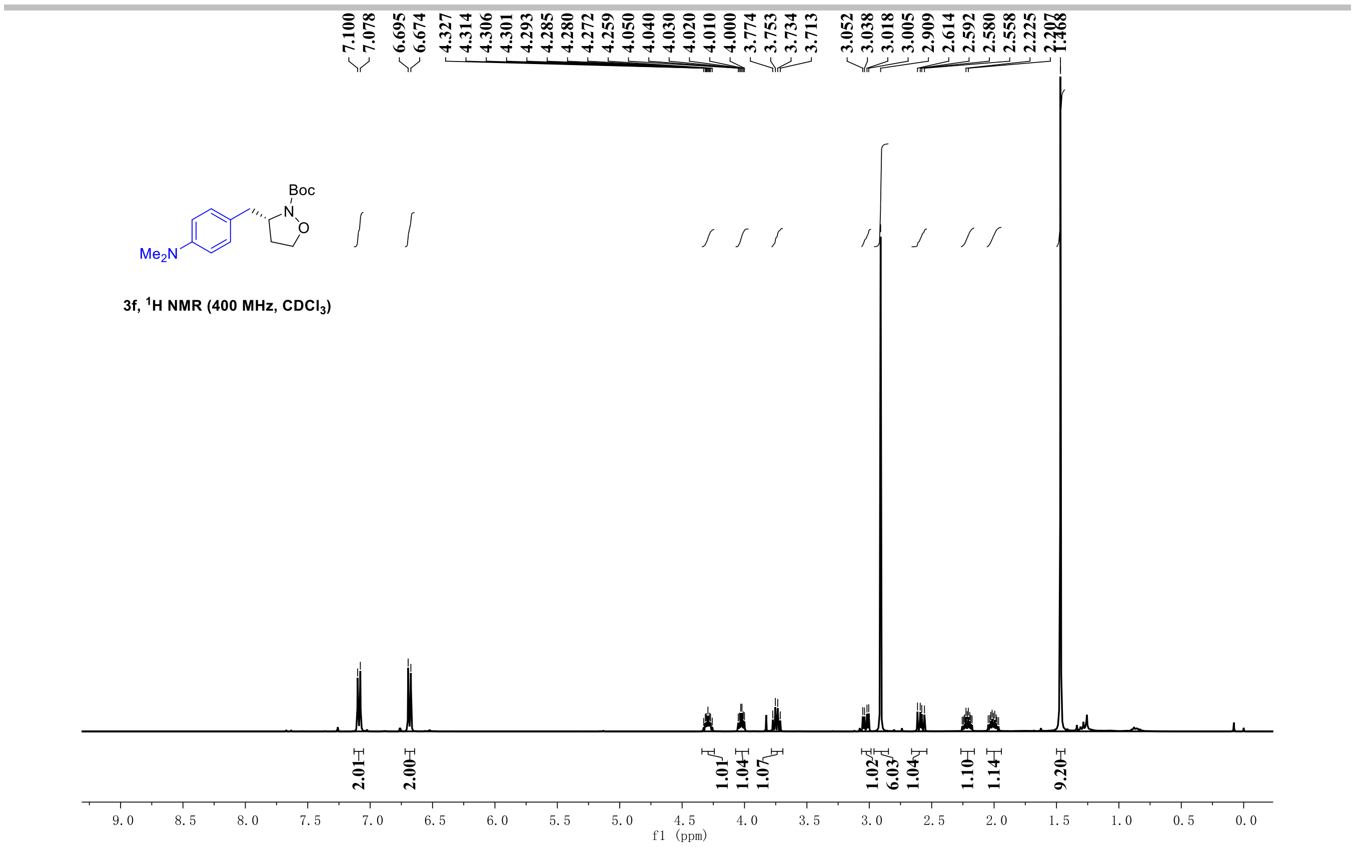
7.100
7.078
6.695
6.674
4.327
4.314
4.306
4.301
4.293
4.285
4.280
4.272
4.259
4.050
4.040
4.030
4.020
4.010
4.000
3.774
3.753
3.734
3.713
3.052
3.038
3.018
3.005
2.909
2.614
2.592
2.580
2.558
2.225
1.468
Boc
N
O
Me_2N
3f, ^{1}H NMR (400 MHz, $CDCl_3$)
2.01
2.00
1.01
1.04
1.07
1.02
6.03
1.04
1.10
1.14
9.20
9.0 8.5 8.0 7.5 7.0 6.5 6.0 5.5 5.0 4.5 4.0 3.5 3.0 2.5 2.0 1.5 1.0 0.5 0.0
f1 (ppm)

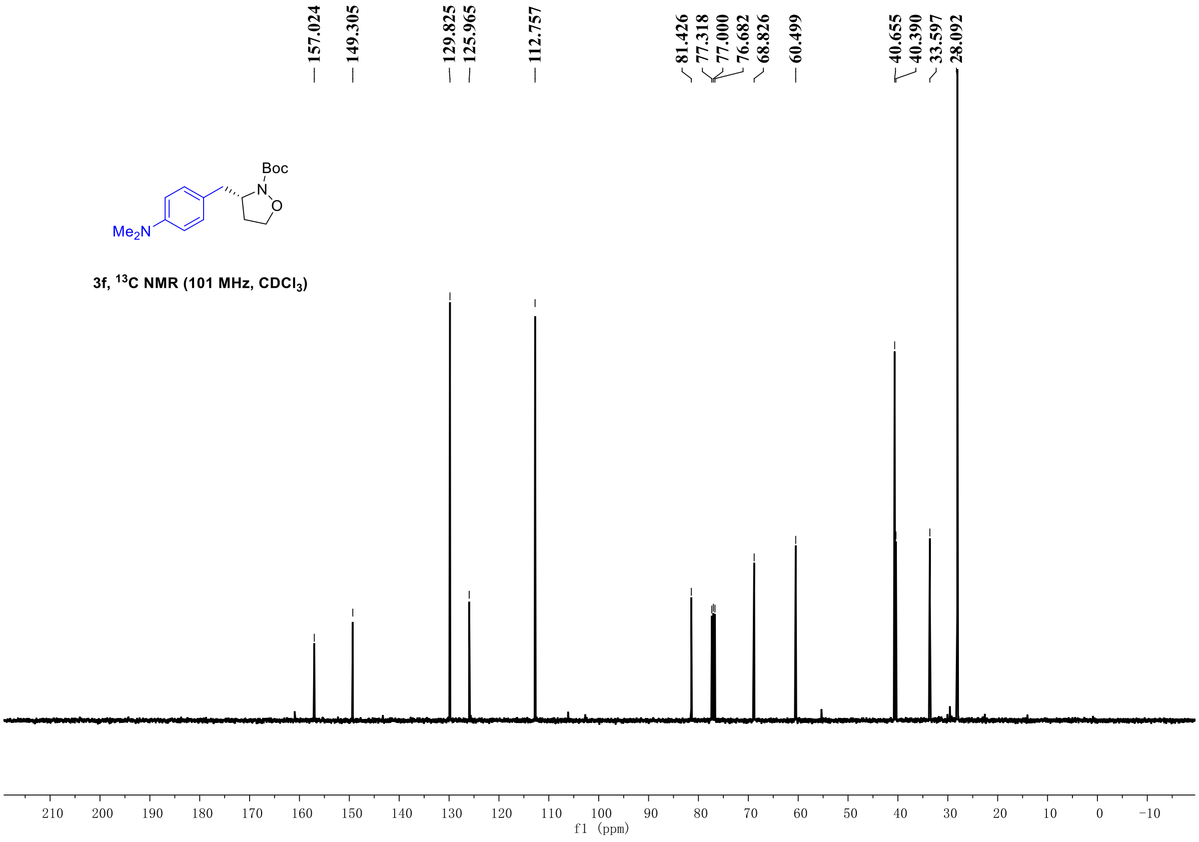
157.024
149.305
129.825
125.965
112.757
81.426
77.318
77.000
76.682
68.826
60.499
40.655
40.390
33.597
28.092
Boc
N
O
Me_2N
3f, ^{13}C NMR (101 MHz, $CDCl_3$)
210 200 190 180 170 160 150 140 130 120 110 100 90 80 70 60 50 40 30 20 10 0 -10
f1 (ppm)

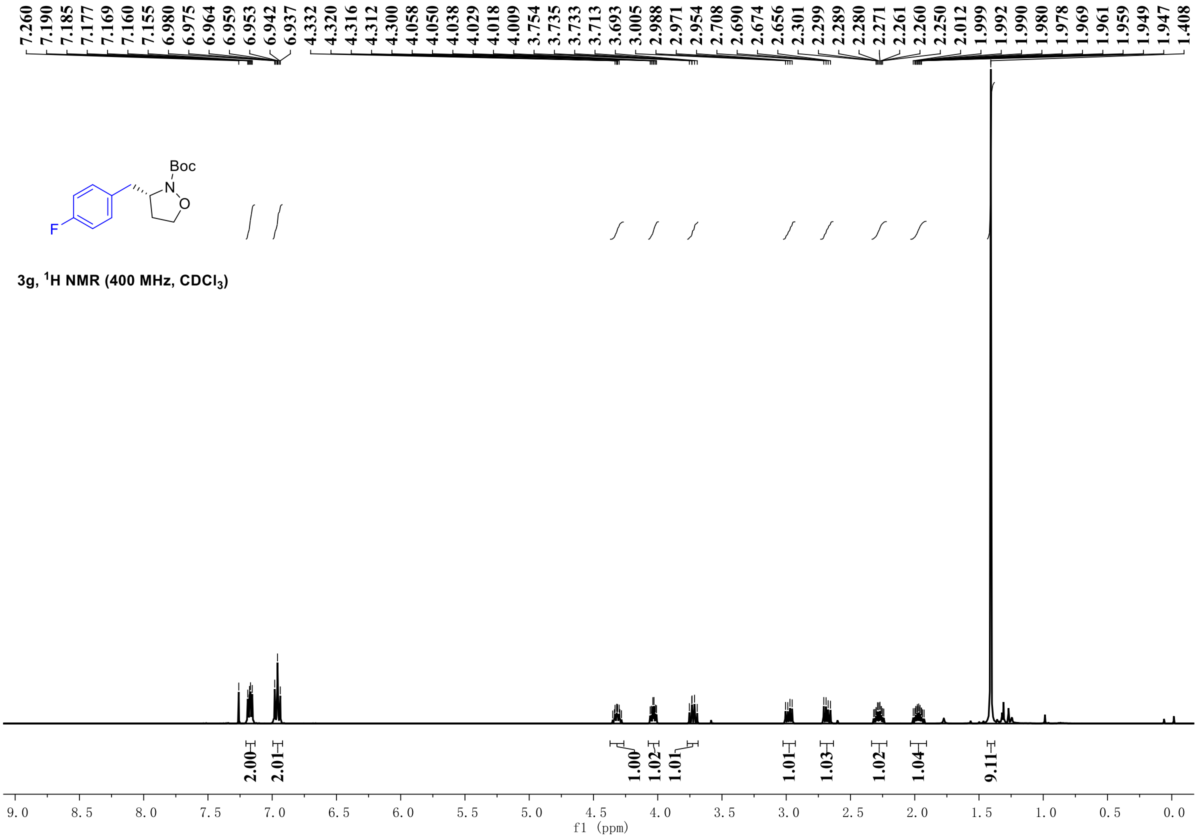
7.260
7.190
7.185
7.177
7.169
7.160
7.155
6.980
6.975
6.964
6.959
6.953
6.942
6.937
4.332
4.320
4.316
4.312
4.300
4.058
4.050
4.038
4.029
4.018
4.009
3.754
3.735
3.733
3.713
3.693
3.005
2.988
2.971
2.954
2.708
2.690
2.674
2.656
2.301
2.299
2.289
2.280
2.271
2.261
2.260
2.250
2.012
1.999
1.992
1.990
1.980
1.978
1.969
1.961
1.959
1.949
1.947
1.408
Boc
N
O
F
3g, 1H NMR (400 MHz, CDCl3)
2.00
2.01
1.00
1.02
1.01
1.01
1.03
1.02
1.04
9.11
f1 (ppm)

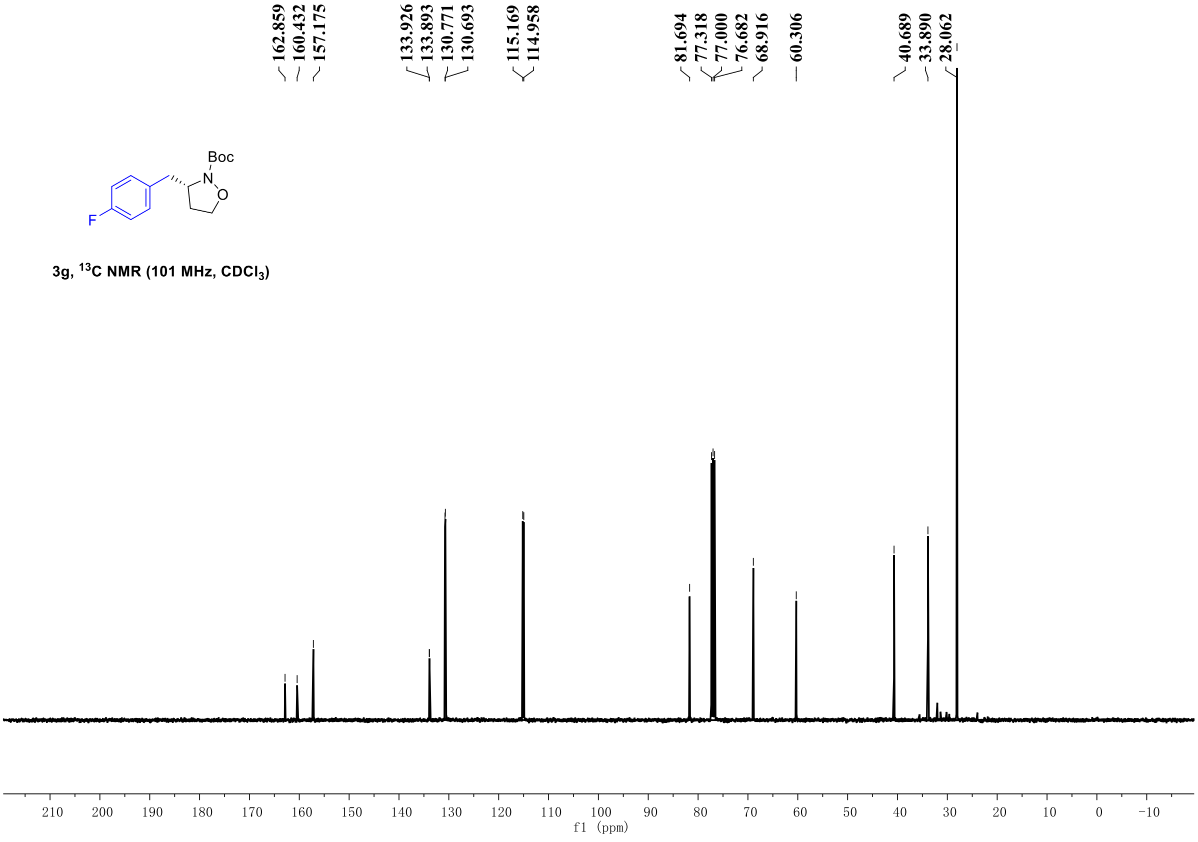
162.859
160.432
157.175
133.926
133.893
130.771
130.693
115.169
114.958
81.694
77.318
77.000
76.682
68.916
60.306
40.689
33.890
28.062
Boc
N
O
F
3g, 13C NMR (101 MHz, CDCl3)
f1 (ppm)

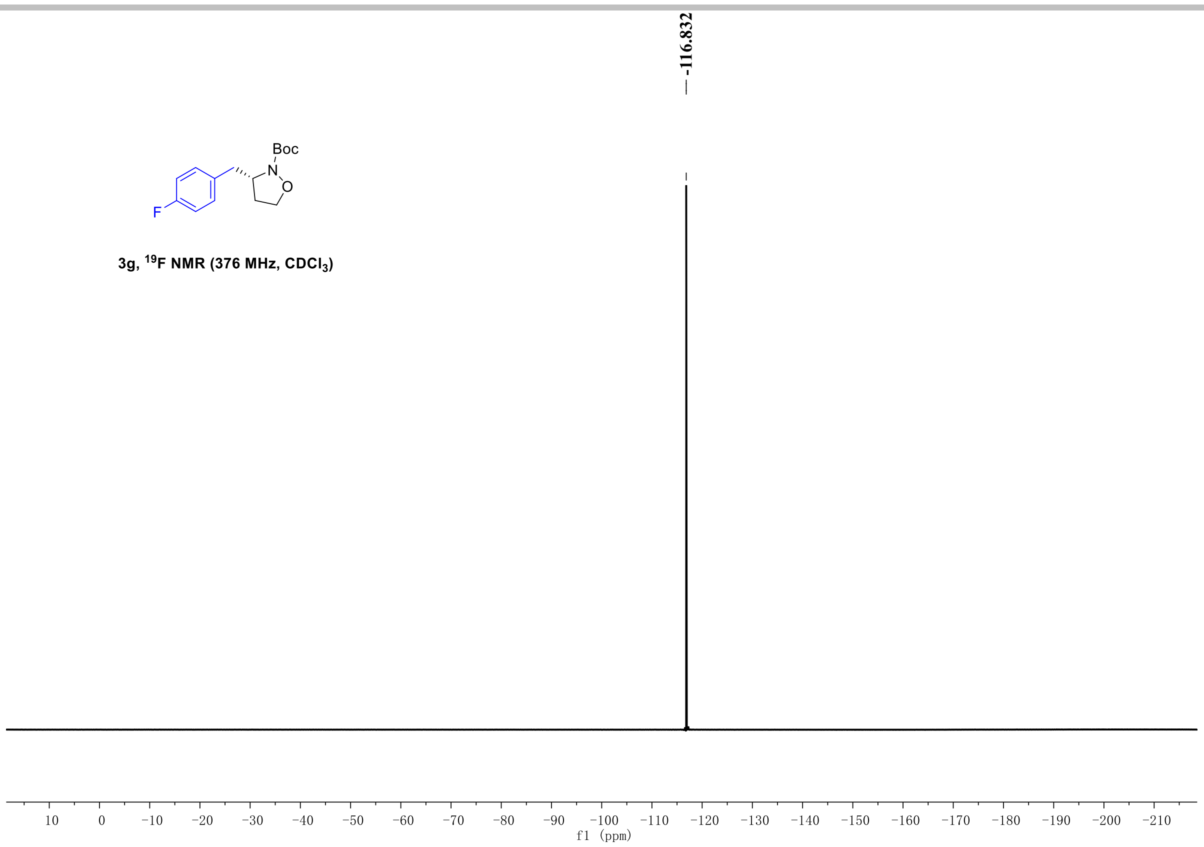

3g, ^{19}F NMR (376 MHz, $CDCl_3$)

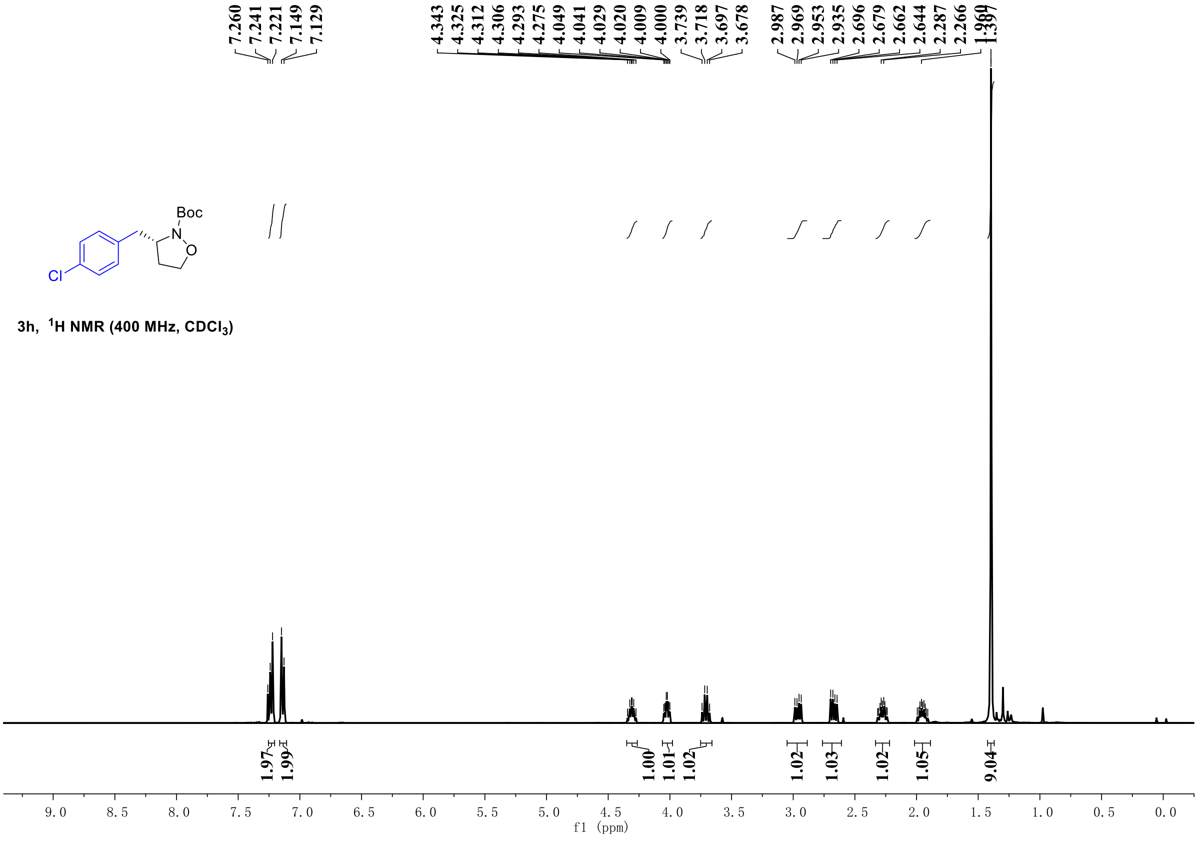

3h, ^{1}H NMR (400 MHz, $CDCl_3$)

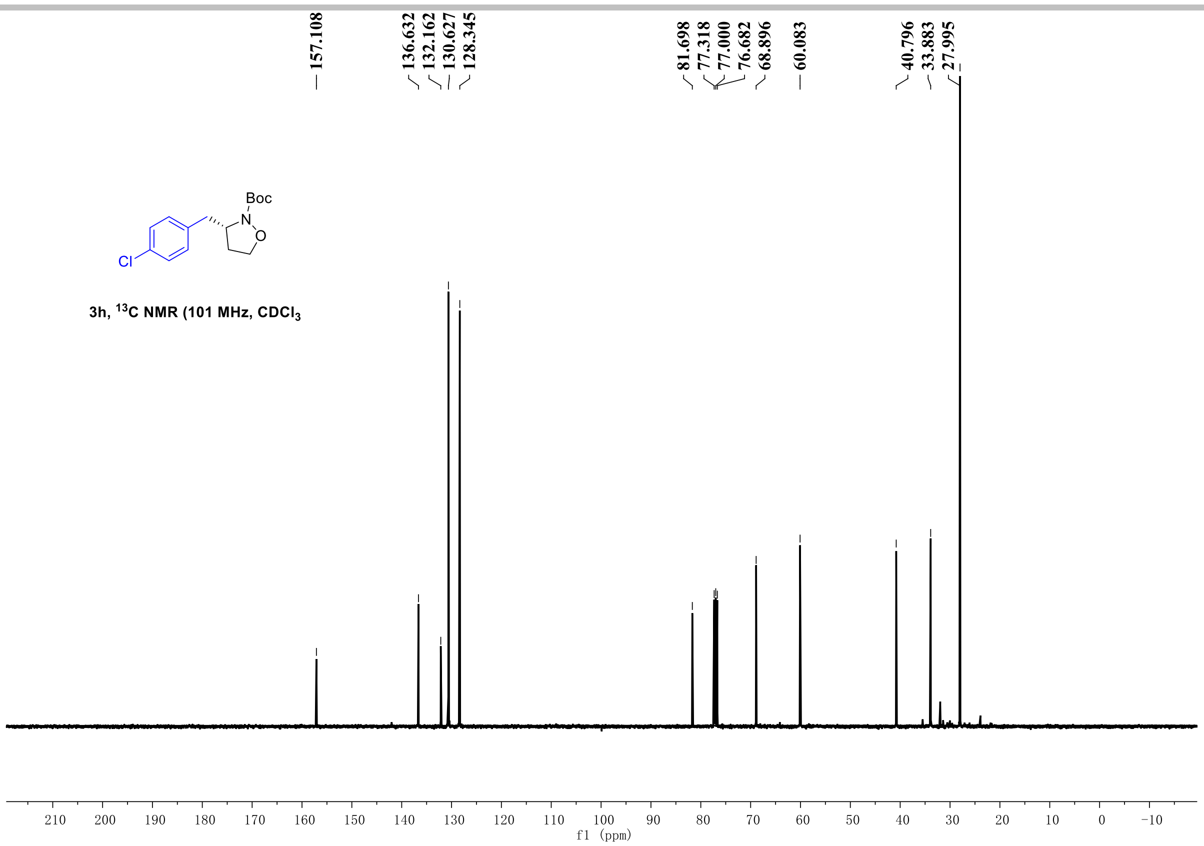
157.108
136.632
132.162
130.627
128.345
81.698
77.318
77.000
76.682
68.896
60.083
40.796
33.883
27.995
Boc
N
O
Cl
3h, 13C NMR (101 MHz, CDCl3)
210 200 190 180 170 160 150 140 130 120 110 100 90 80 70 60 50 40 30 20 10 0 -10
f1 (ppm)

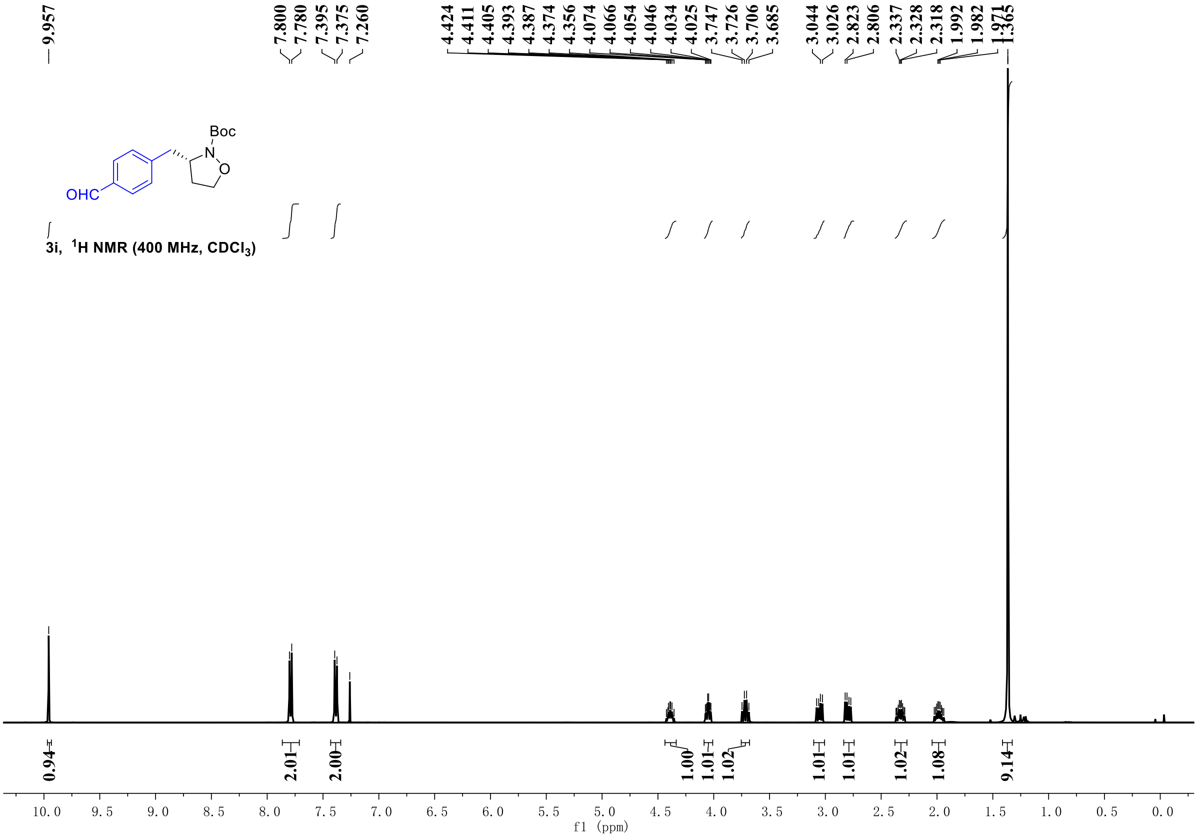
9.957
7.800
7.780
7.395
7.375
7.260
4.424
4.411
4.405
4.393
4.387
4.374
4.356
4.074
4.066
4.054
4.046
4.034
4.025
3.747
3.726
3.706
3.685
3.044
3.026
2.823
2.806
2.337
2.328
2.318
1.992
1.982
1.971
1.365
Boc
N
O
OHC
3i, 1H NMR (400 MHz, CDCl3)
0.94
2.01
2.00
1.00
1.01
1.02
1.01
1.01
1.02
1.08
9.14
10.0 9.5 9.0 8.5 8.0 7.5 7.0 6.5 6.0 5.5 5.0 4.5 4.0 3.5 3.0 2.5 2.0 1.5 1.0 0.5 0.0
f1 (ppm)

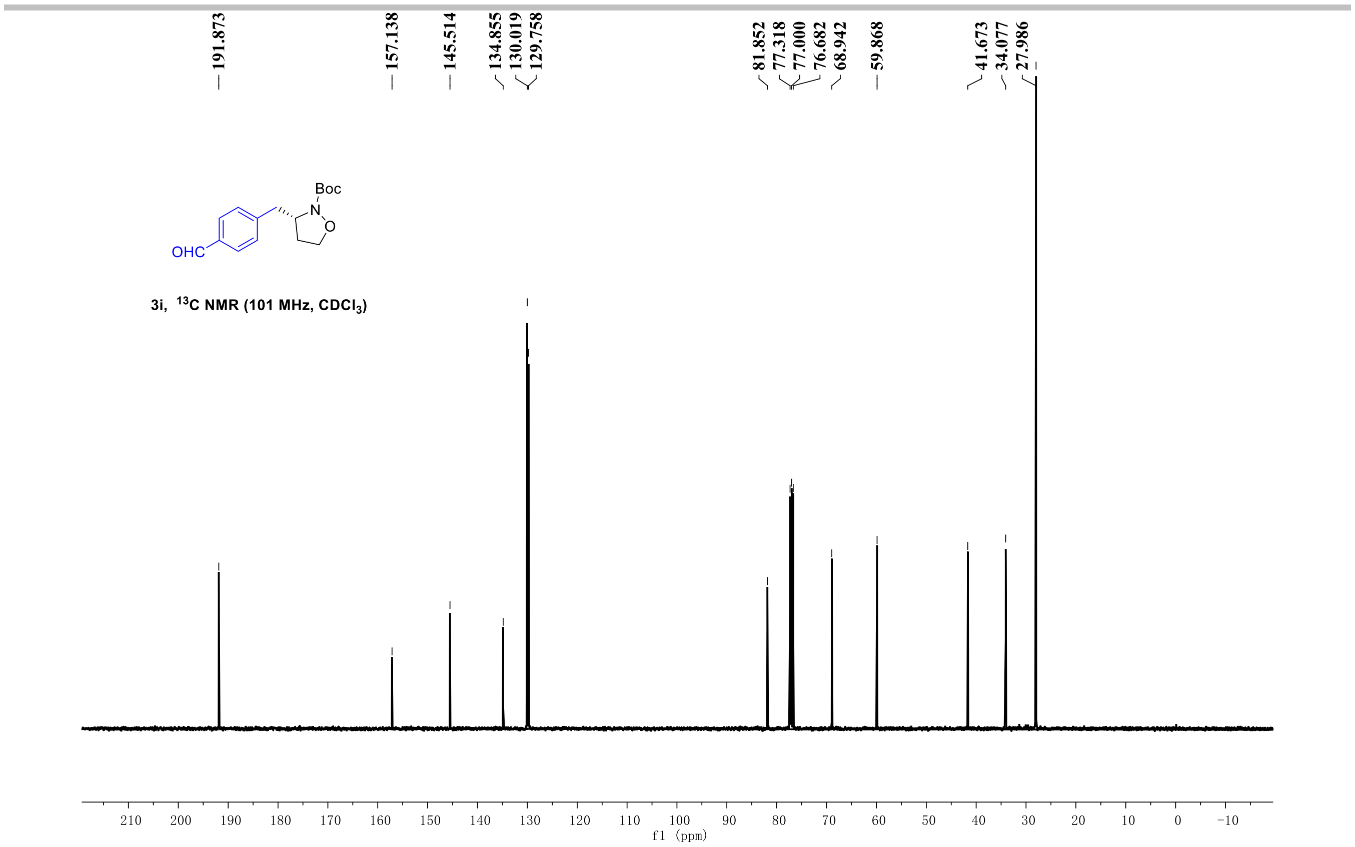
191.873
157.138
145.514
134.855
130.019
129.758
81.852
77.318
77.000
76.682
68.942
59.868
41.673
34.077
27.986
Boc
N
O
OHC
3i, ^{13}C NMR (101 MHz, $CDCl_3$)
f1 (ppm)

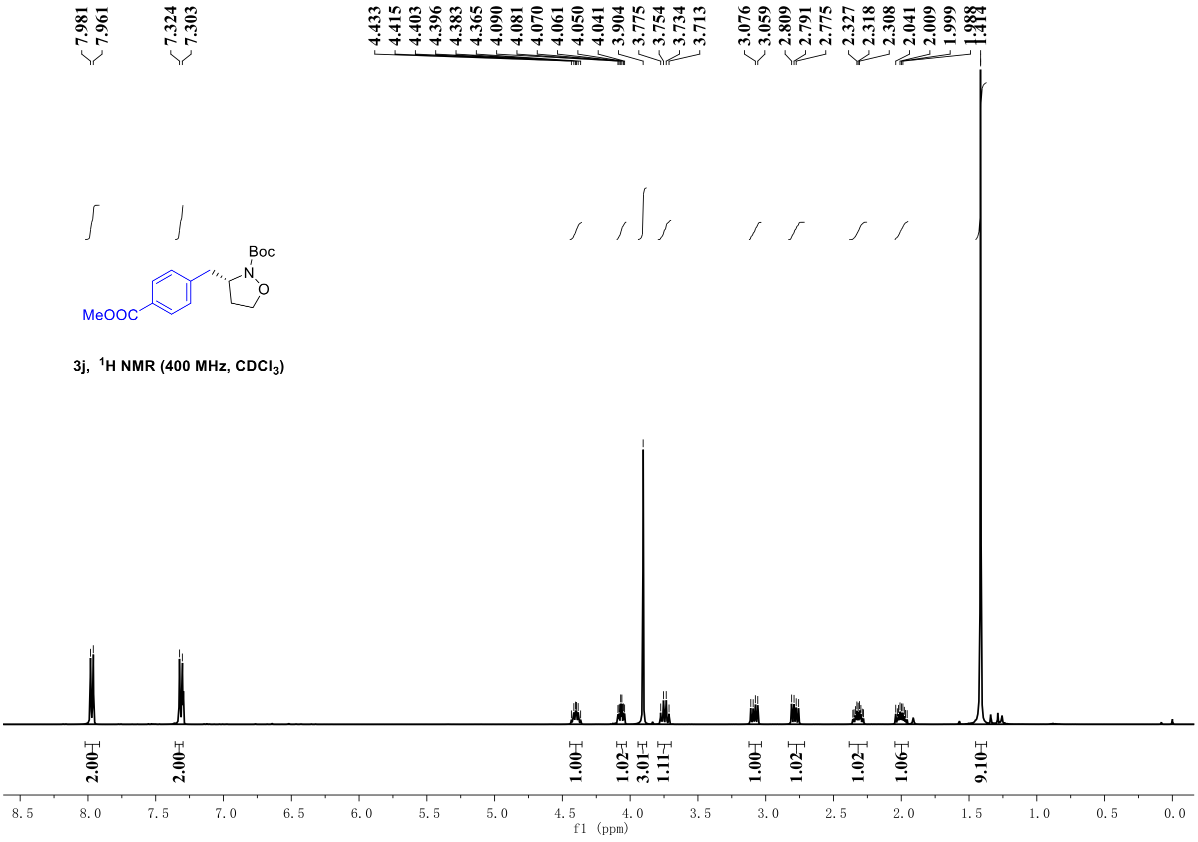
7.981
7.961
7.324
7.303
4.433
4.415
4.403
4.396
4.383
4.365
4.090
4.081
4.070
4.061
4.050
4.041
3.904
3.775
3.754
3.734
3.713
3.076
3.059
2.809
2.791
2.775
2.327
2.318
2.308
2.041
2.009
1.999
1.988
1.414
Boc
N
O
MeOOC
3j, ^{1}H NMR (400 MHz, $CDCl_3$)
2.00
2.00
1.00
1.02
3.01
1.11
1.00
1.02
1.02
1.06
9.10
f1 (ppm)

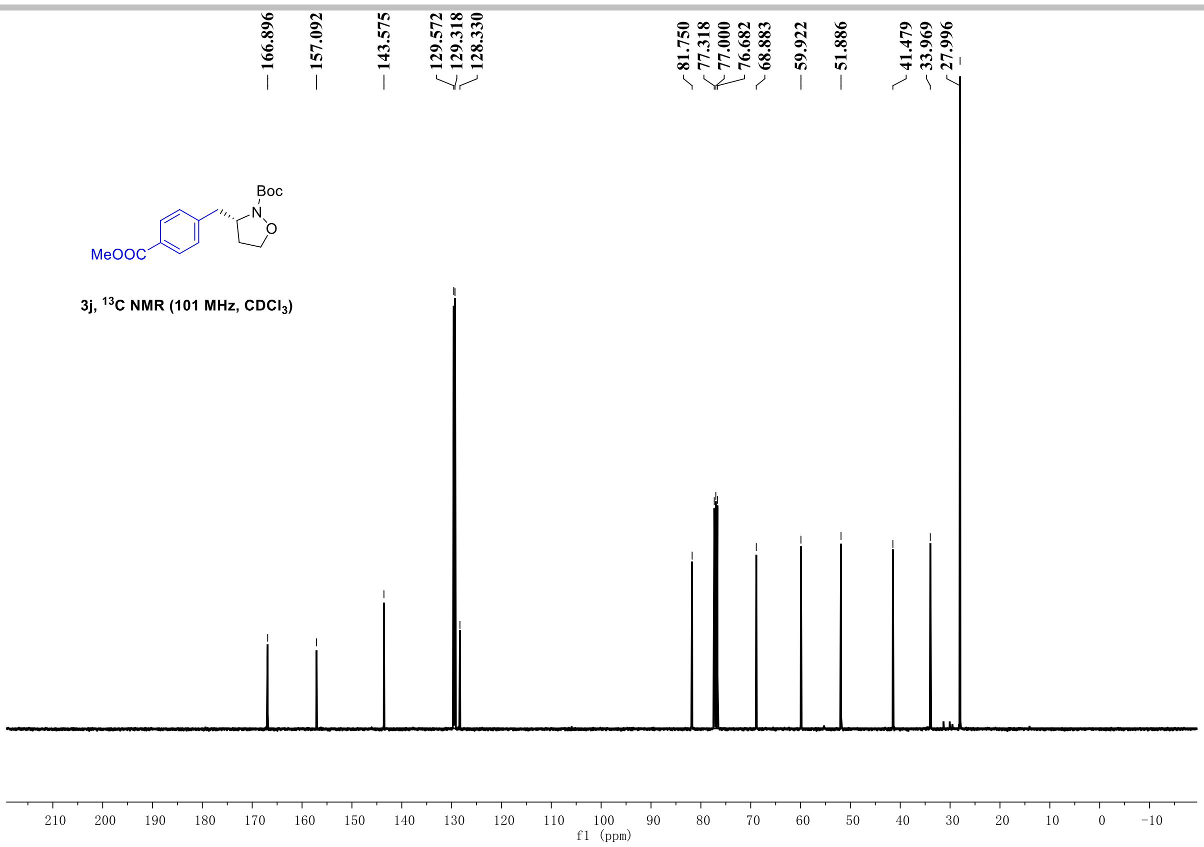
166.896
157.092
143.575
129.572
129.318
128.330
81.750
77.318
77.000
76.682
68.883
59.922
51.886
41.479
33.969
27.996
Boc
N
O
MeOOC
3j, 13C NMR (101 MHz, CDCl3)
f1 (ppm)

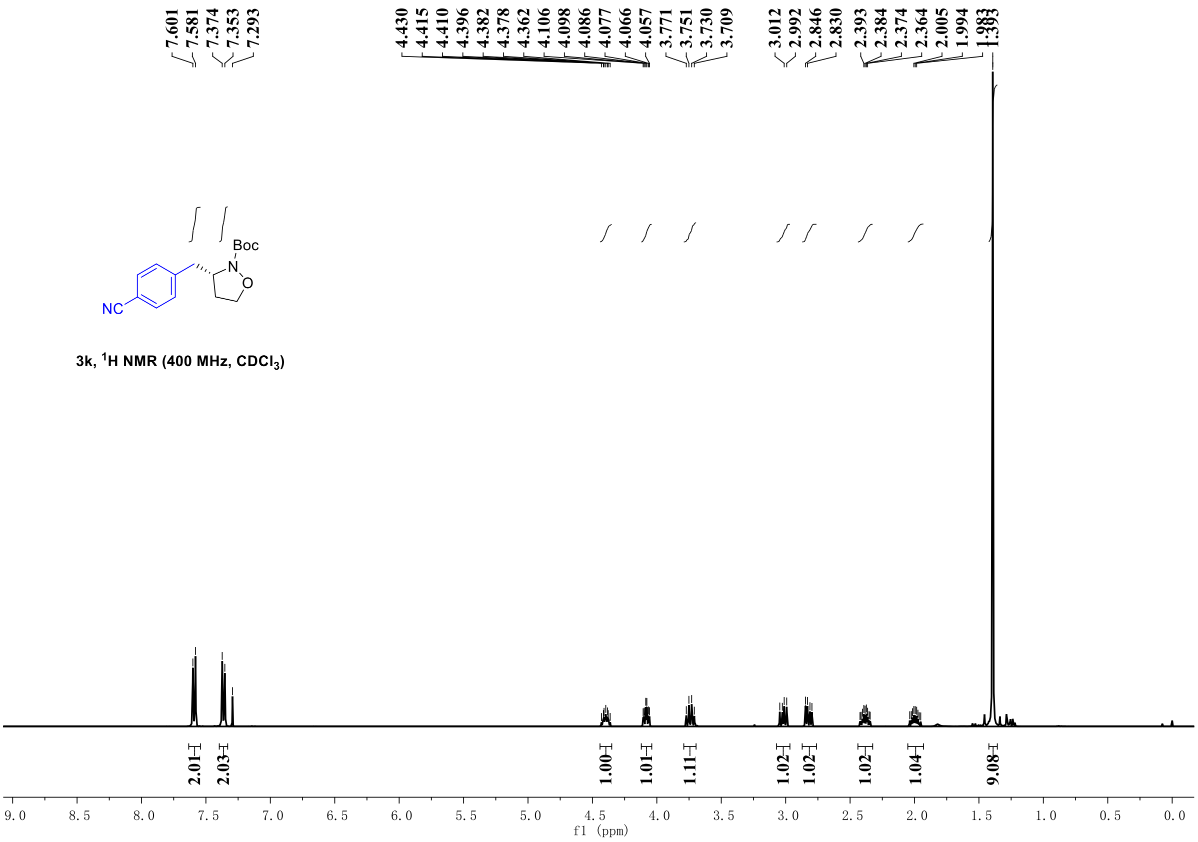
7.601
7.581
7.374
7.353
7.293
4.430
4.415
4.410
4.396
4.382
4.378
4.362
4.106
4.098
4.086
4.077
4.066
4.057
3.771
3.751
3.730
3.709
3.012
2.992
2.846
2.830
2.393
2.384
2.374
2.364
2.005
1.994
1.983
1.393
Boc
N
O
NC
3k, 1H NMR (400 MHz, CDCl3)
2.01
2.03
1.00
1.01
1.11
1.02
1.02
1.02
1.04
9.08
f1 (ppm)

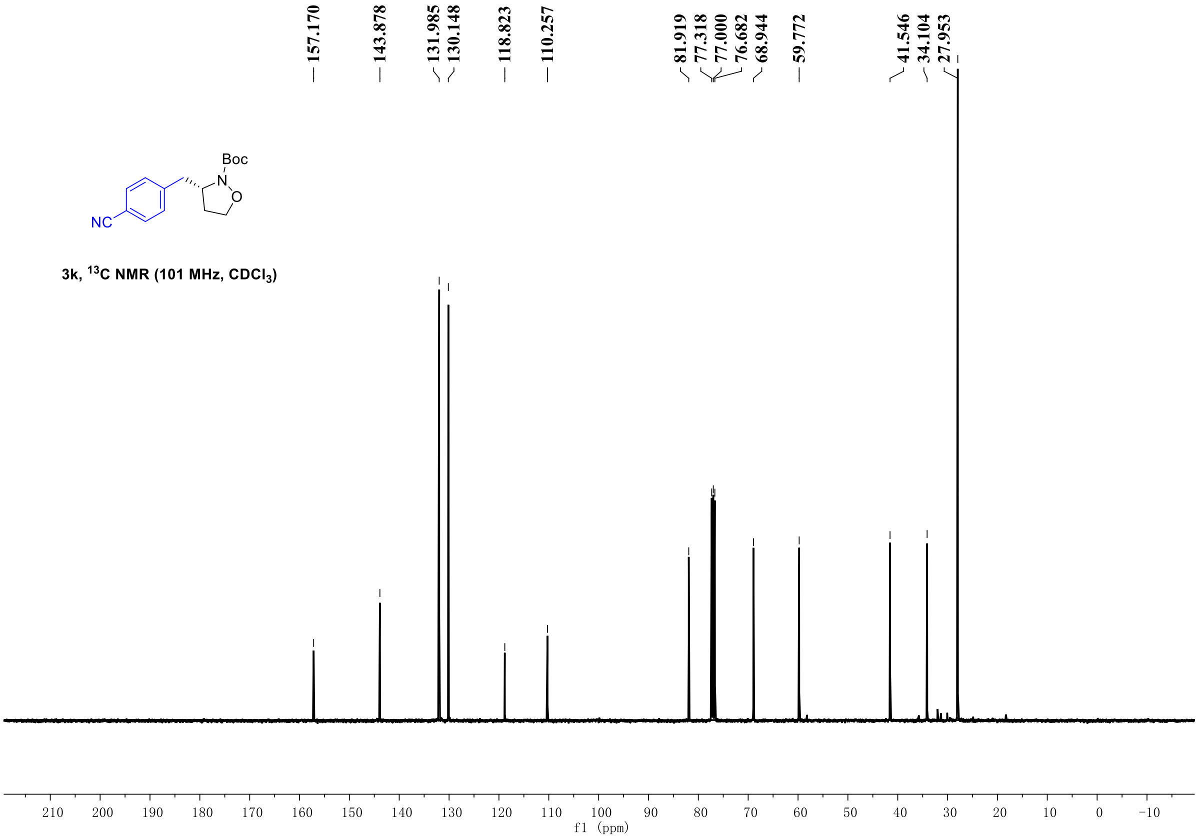

3k, ^{13}C NMR (101 MHz, $CDCl_3$)

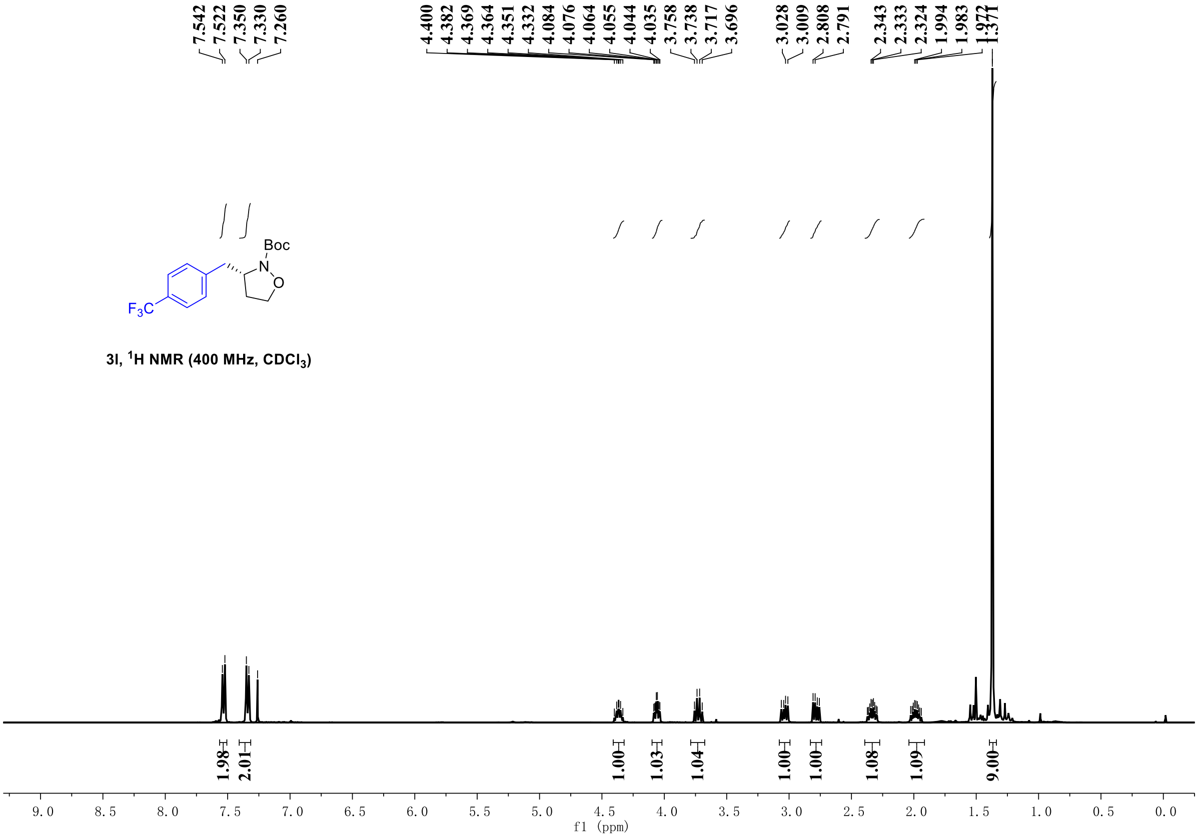

3l, ^{1}H NMR (400 MHz, $CDCl_3$)

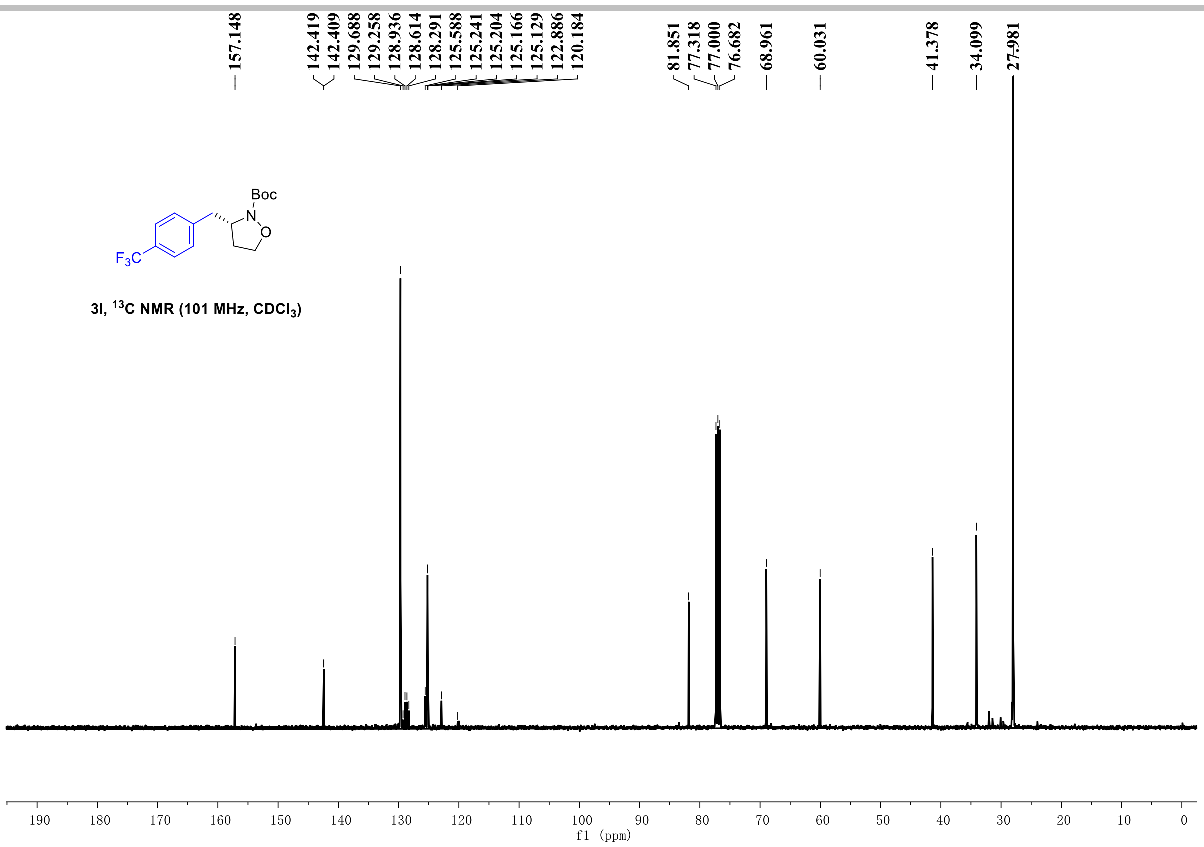
157.148
142.419
142.409
129.688
129.258
128.936
128.614
128.291
125.588
125.241
125.204
125.166
125.129
122.886
120.184
81.851
77.318
77.000
76.682
68.961
60.031
41.378
34.099
27.981
Boc
N
O
F_3C
3l, ^{13}C NMR (101 MHz, $CDCl_3$)
f1 (ppm)

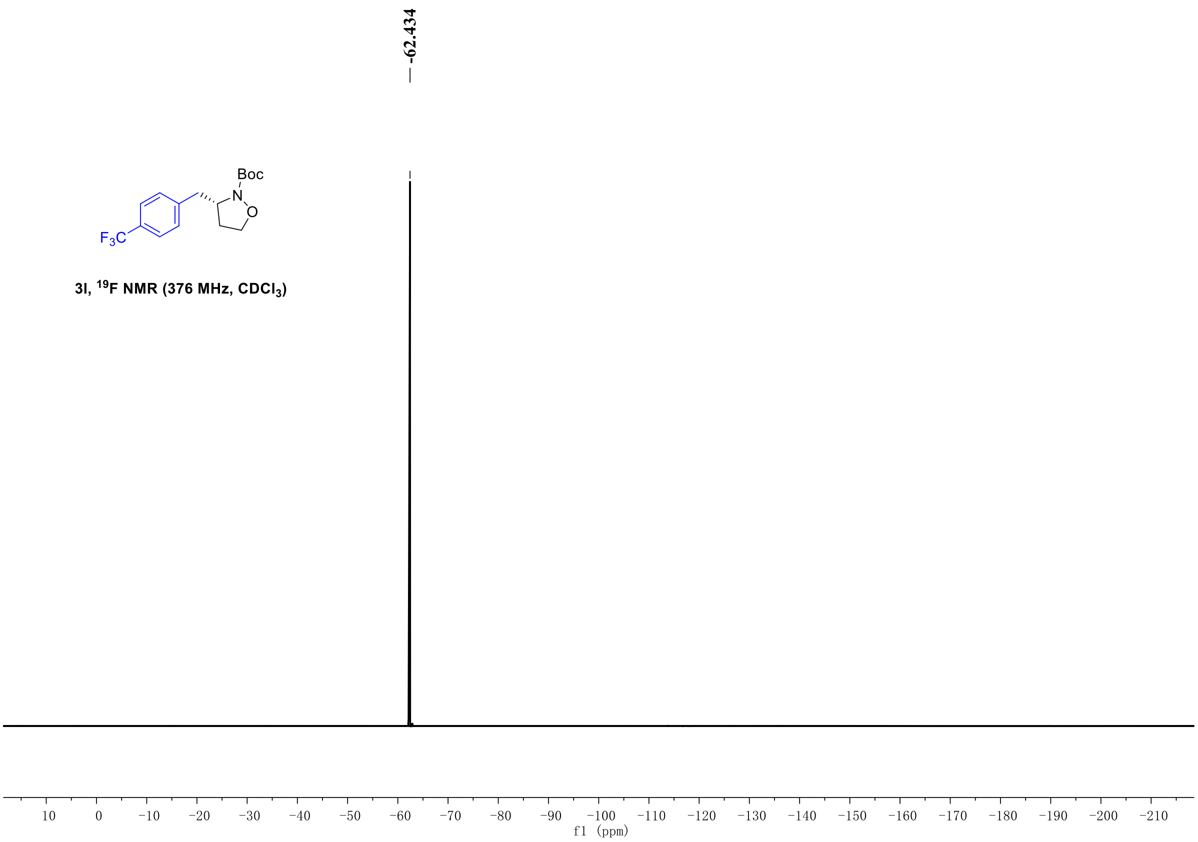
-62.434
Boc
N
O
F_3C
3l, ^{19}F NMR (376 MHz, $CDCl_3$)
f1 (ppm)

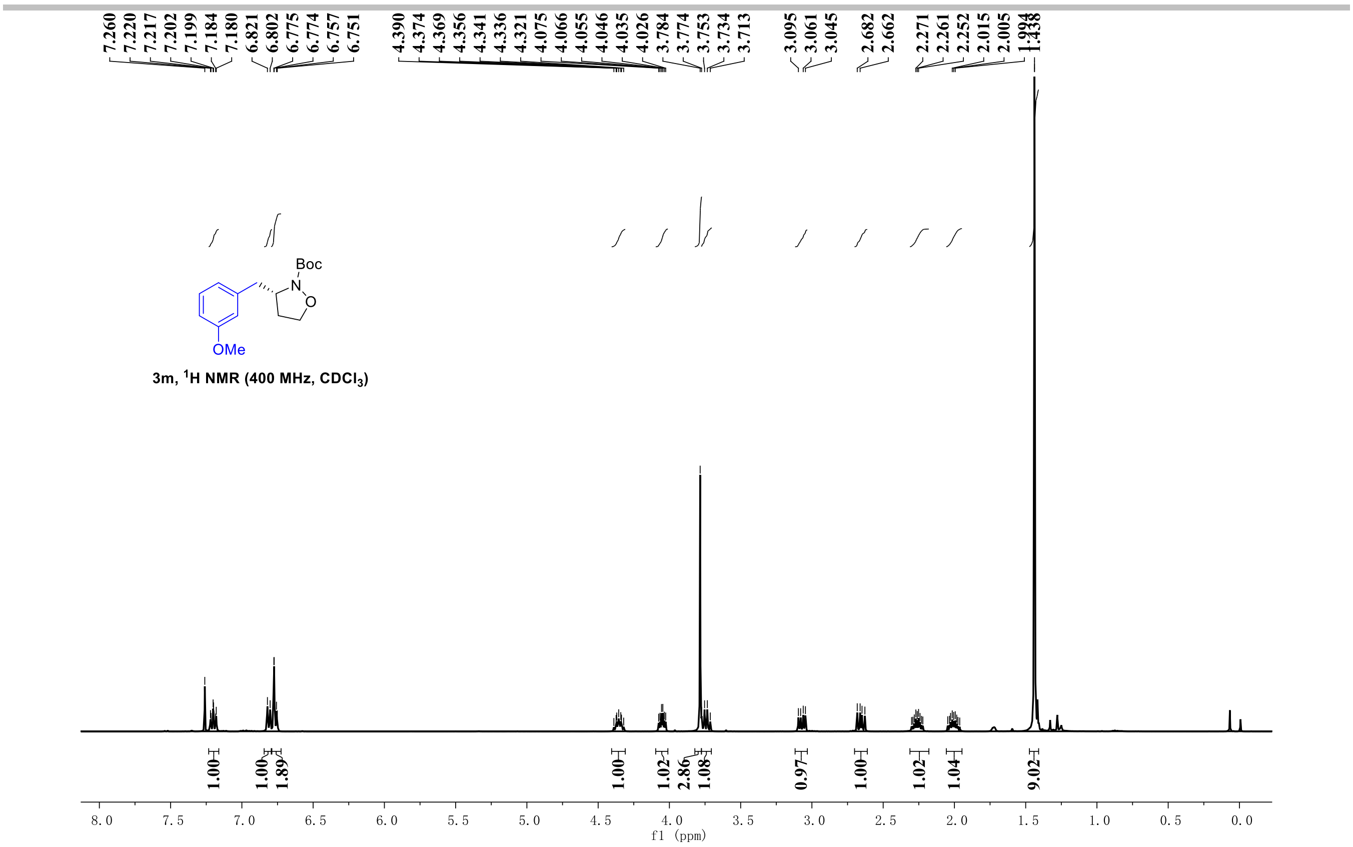
7.260
7.220
7.217
7.202
7.199
7.184
7.180
6.821
6.802
6.775
6.774
6.757
6.751
4.390
4.374
4.369
4.356
4.341
4.336
4.321
4.075
4.066
4.055
4.046
4.035
4.026
3.784
3.774
3.753
3.734
3.713
3.095
3.061
3.045
2.682
2.662
2.271
2.261
2.252
2.015
2.005
1.994
1.438
Boc
N
O
OMe
3m, ¹H NMR (400 MHz, CDCl₃)
1.00
1.00
1.89
1.00
1.02
2.86
1.08
0.97
1.00
1.02
1.04
9.02
f1 (ppm)

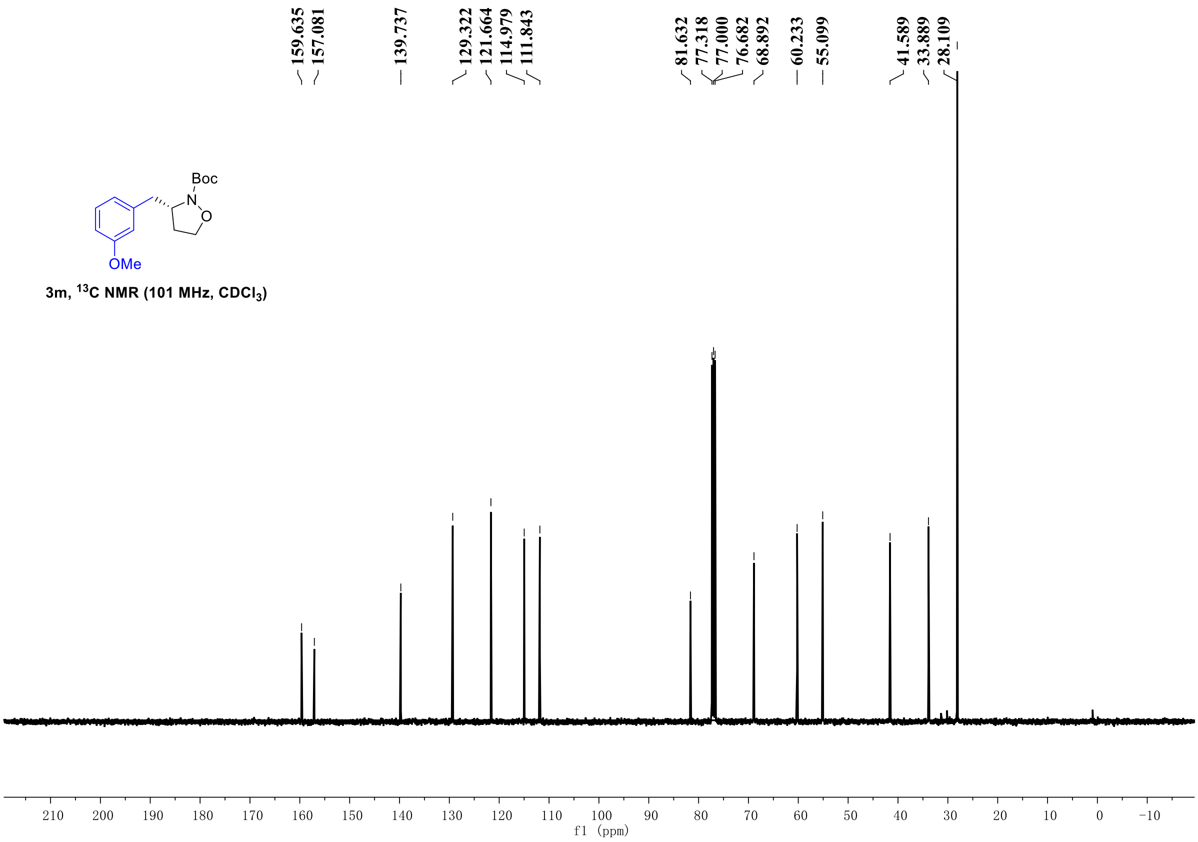
159.635
157.081
139.737
129.322
121.664
114.979
111.843
81.632
77.318
77.000
76.682
68.892
60.233
55.099
41.589
33.889
28.109
Boc
N
O
OMe
3m, ¹³C NMR (101 MHz, CDCl₃)
f1 (ppm)

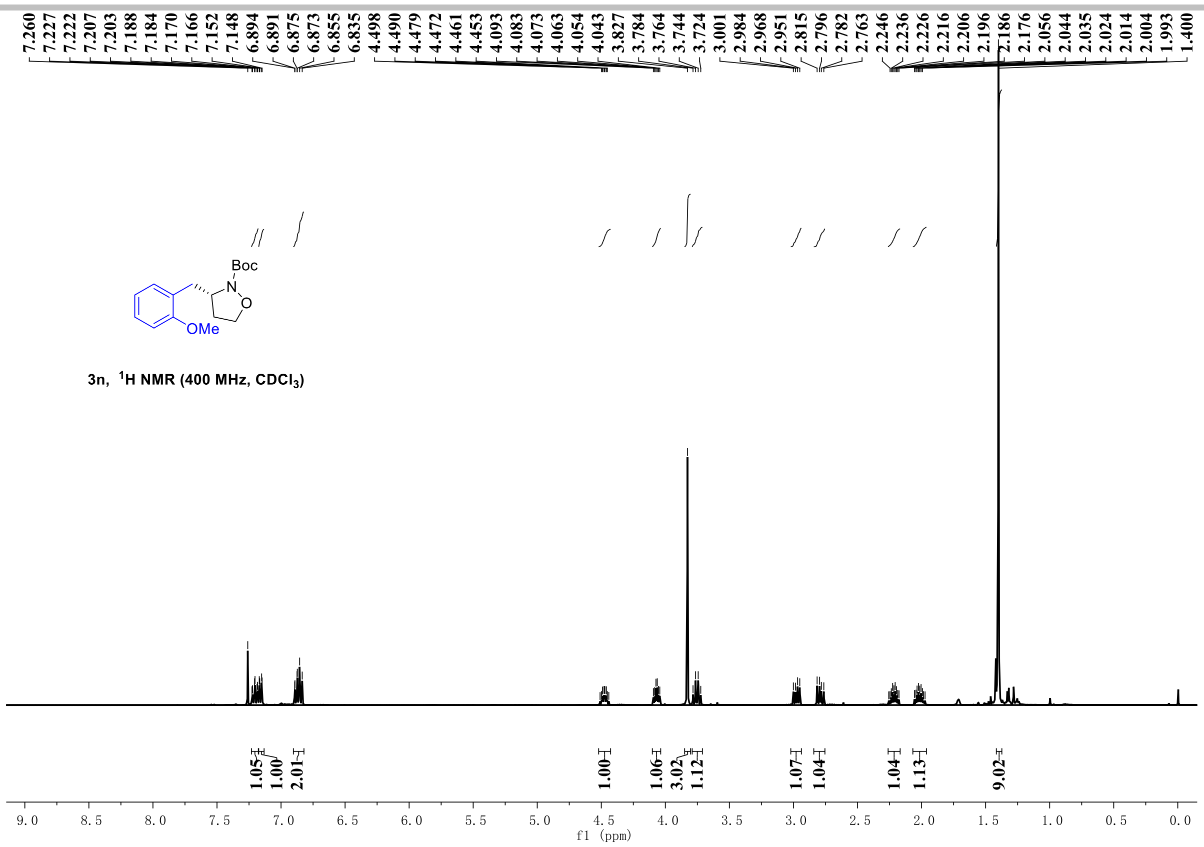

3n, ^{1}H NMR (400 MHz, $CDCl_3$)

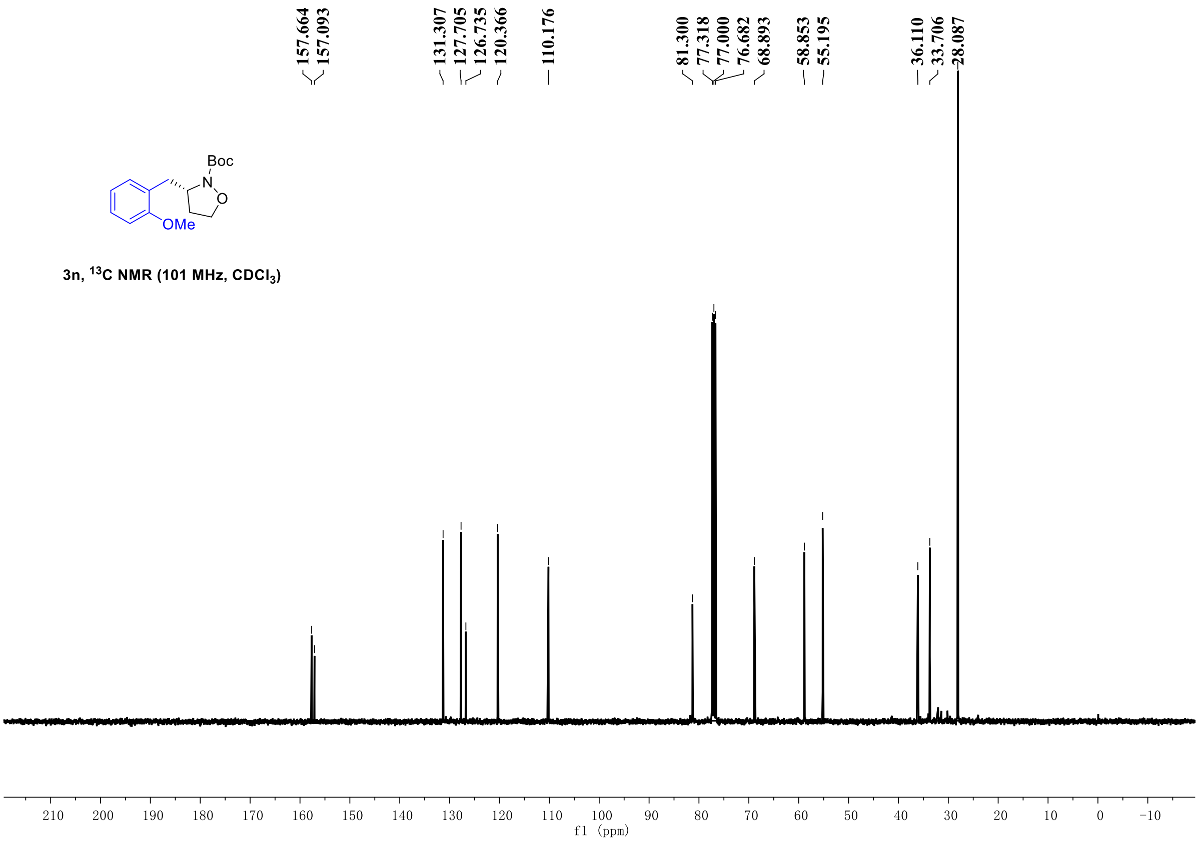

3n, ^{13}C NMR (101 MHz, $CDCl_3$)

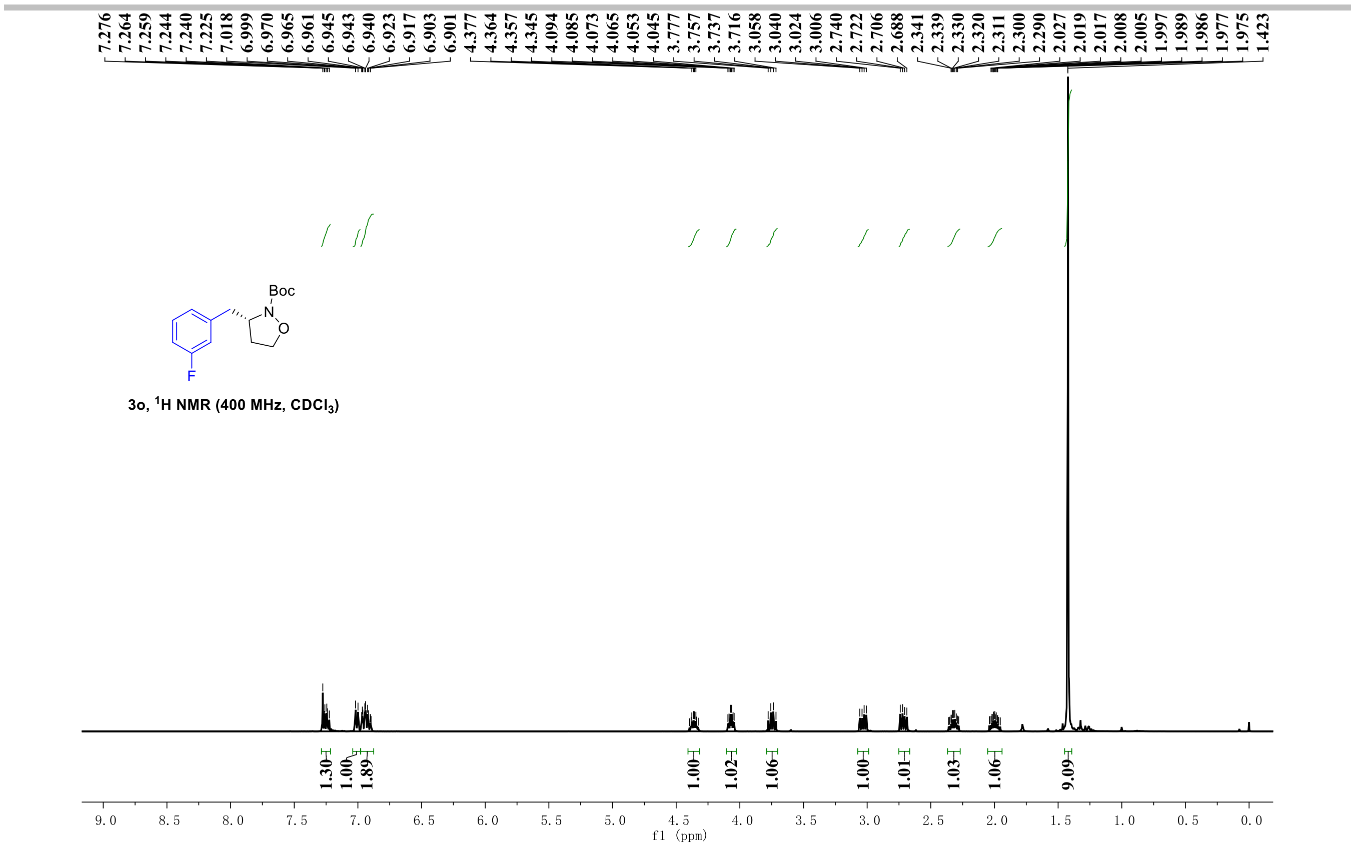

3o, ^{1}H NMR (400 MHz, $CDCl_3$)

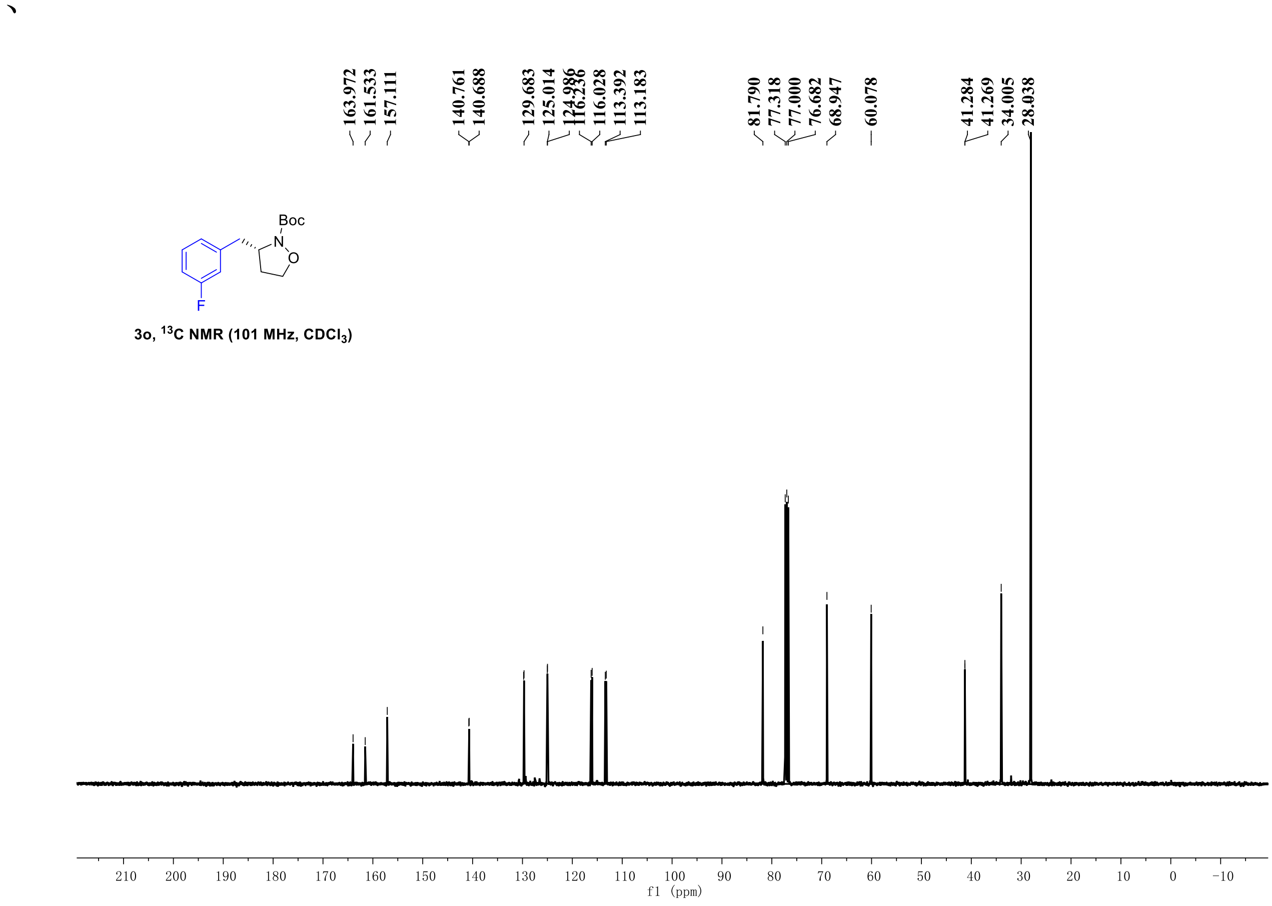

3o, ^{13}C NMR (101 MHz, $CDCl_3$)

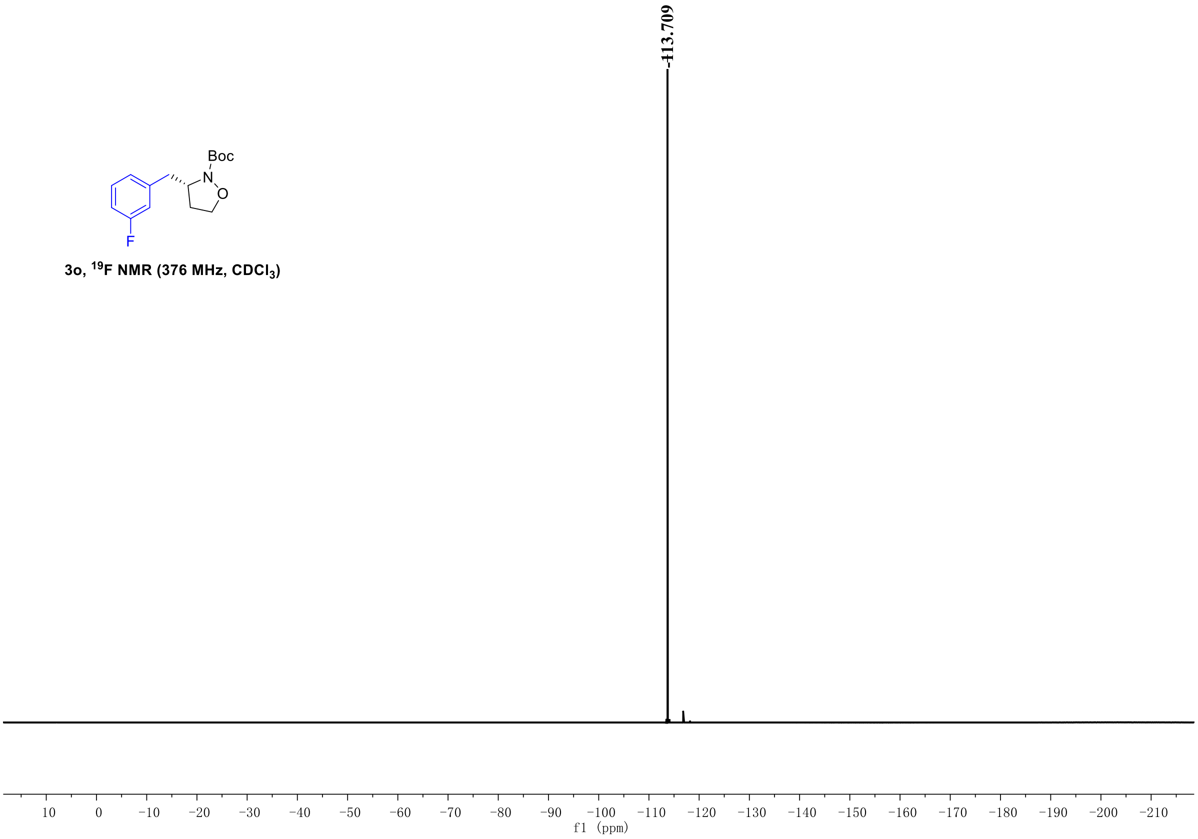

3o, ^{19}F NMR (376 MHz, $CDCl_3$)

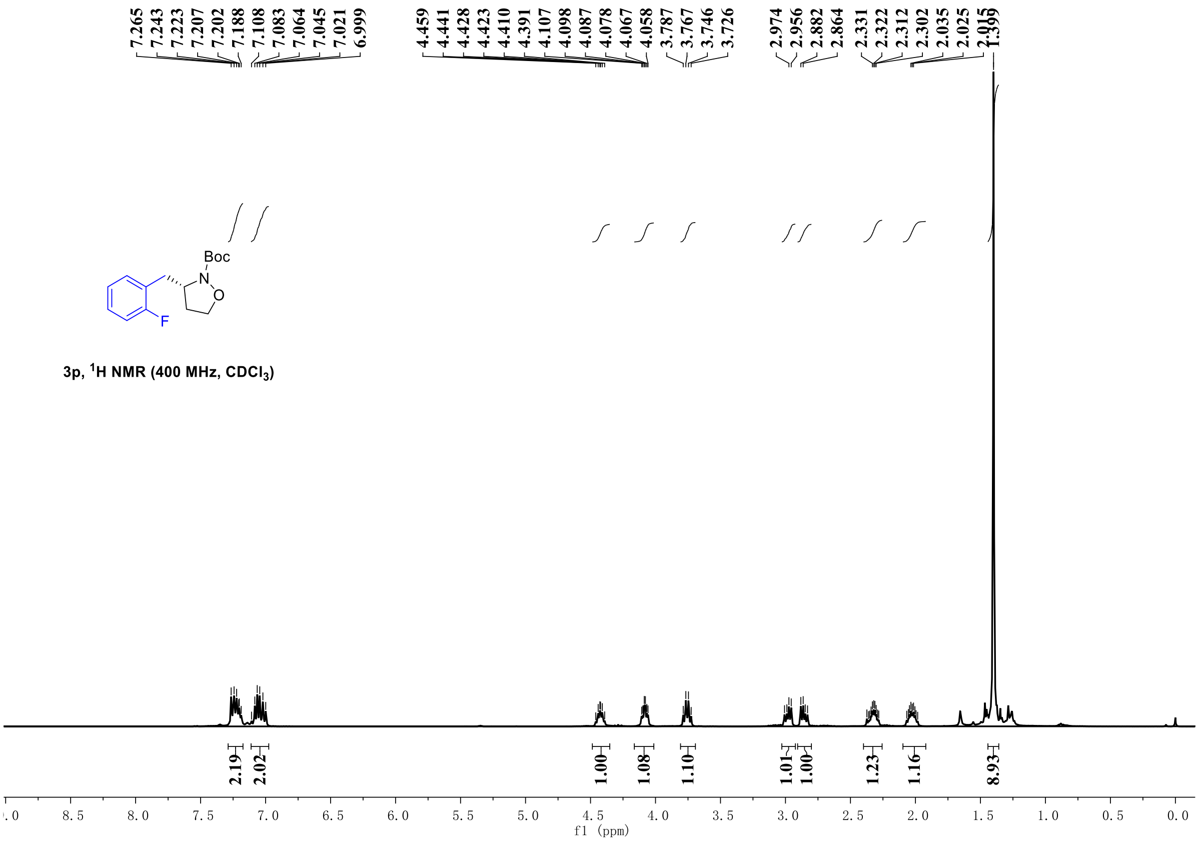

3p, ^{1}H NMR (400 MHz, $CDCl_3$)

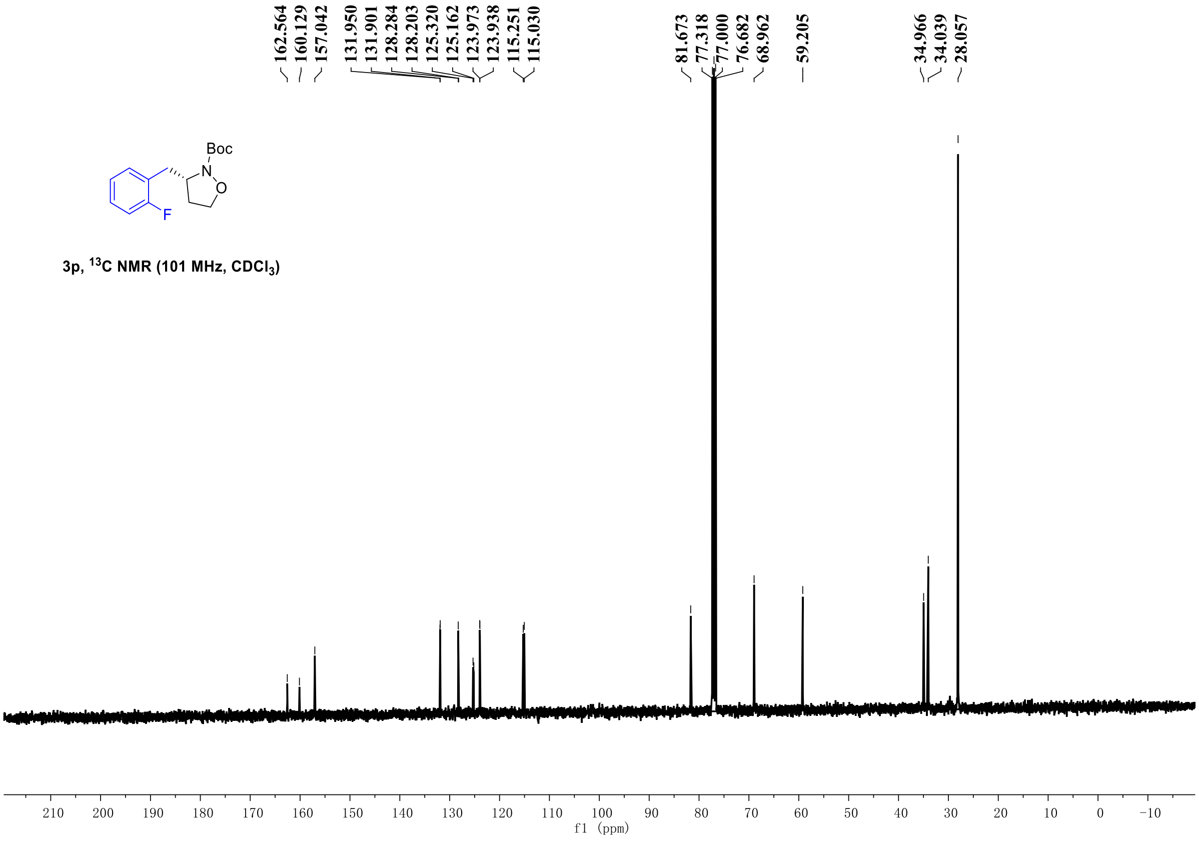

3p, ^{13}C NMR (101 MHz, $CDCl_3$)

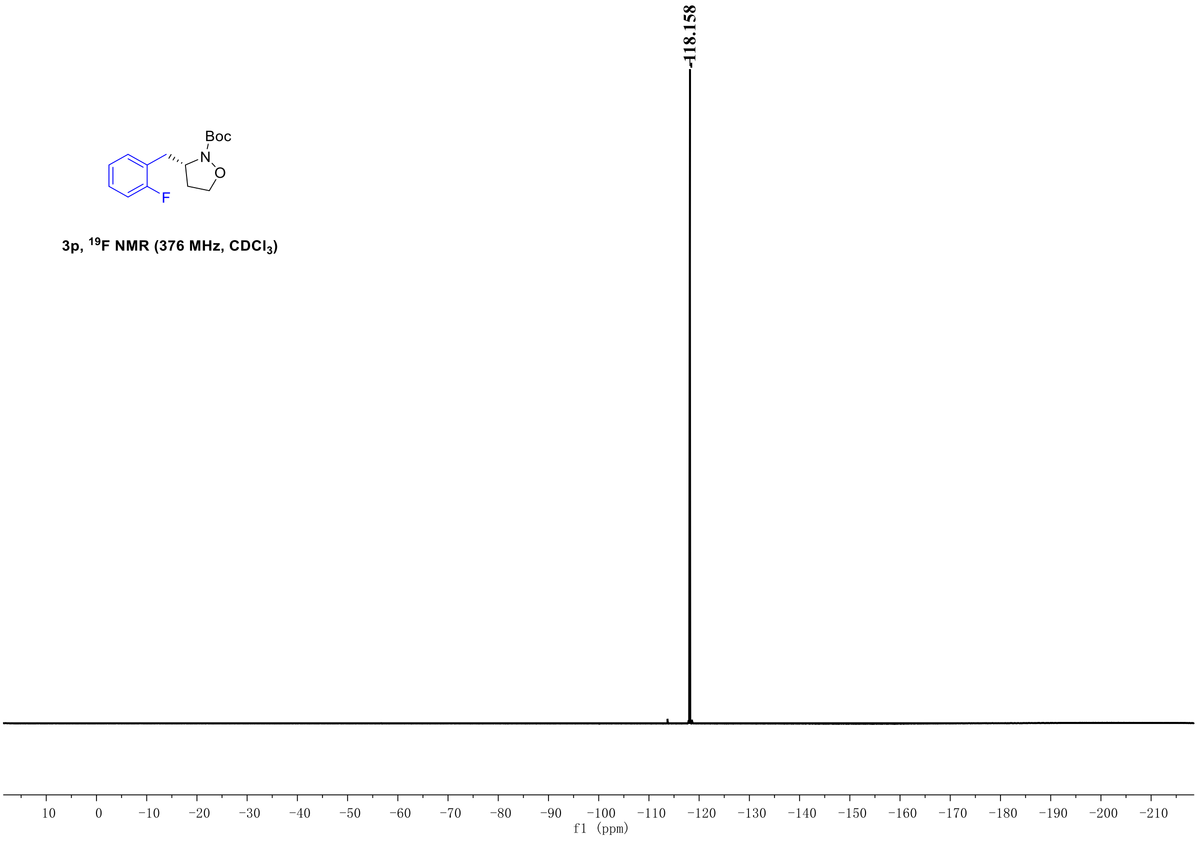

3p, ^{19}F NMR (376 MHz, $CDCl_3$)

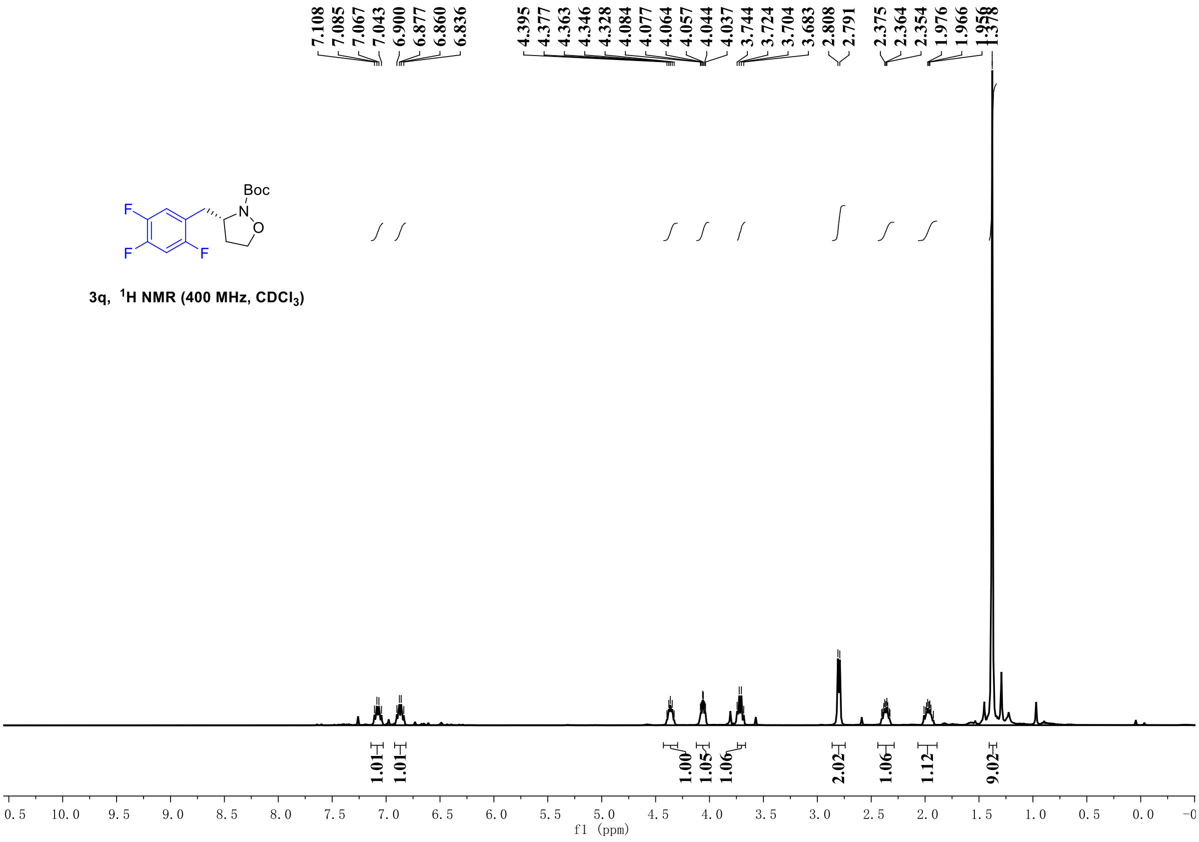

3q, ^{1}H NMR (400 MHz, $CDCl_3$)

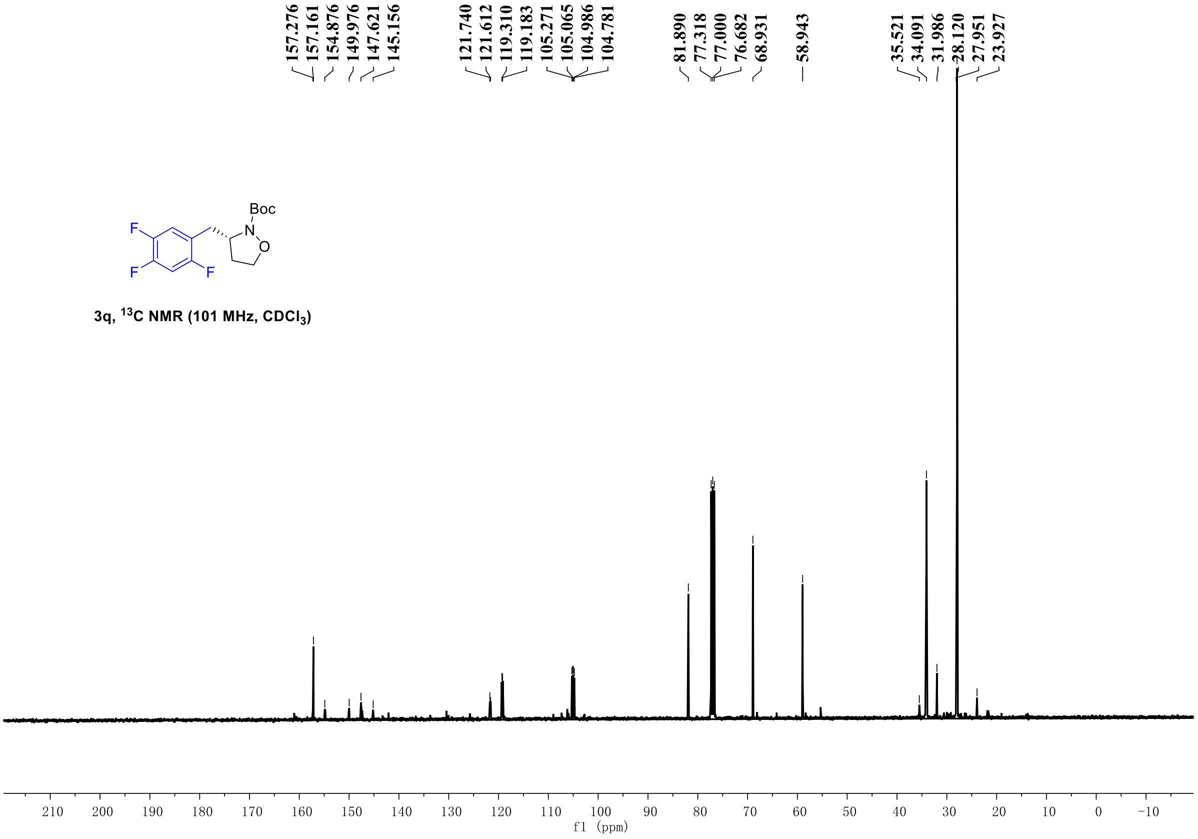

3q, ^{13}C NMR (101 MHz, $CDCl_3$)

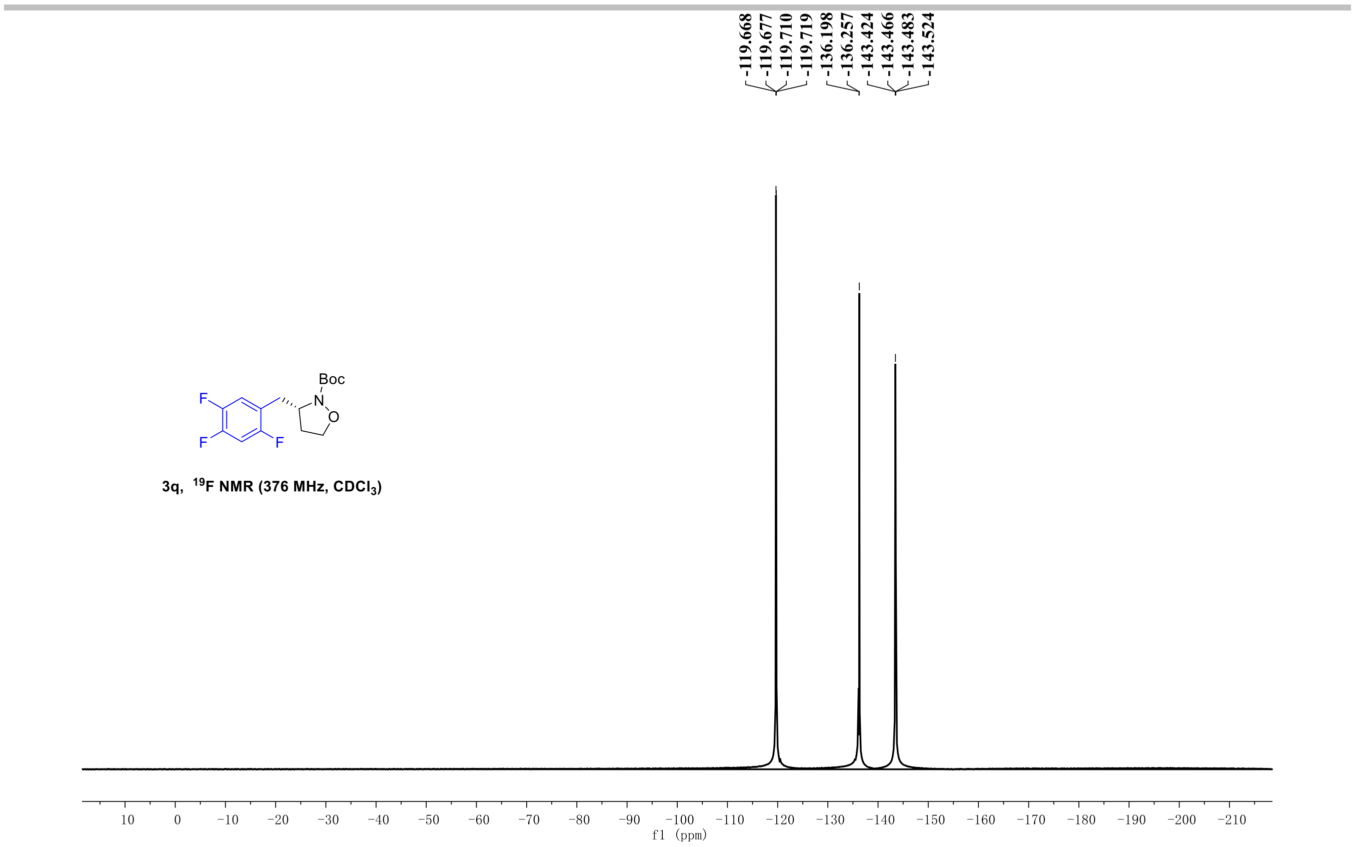
-119.668
-119.677
-119.710
-119.719
-136.198
-136.257
-143.424
-143.466
-143.483
-143.524
Boc
N
O
F
F
F
3q, ^{19}F NMR (376 MHz, $CDCl_3$)
10 0 −10 −20 −30 −40 −50 −60 −70 −80 −90 −100 −110 −120 −130 −140 −150 −160 −170 −180 −190 −200 −210
f1 (ppm)

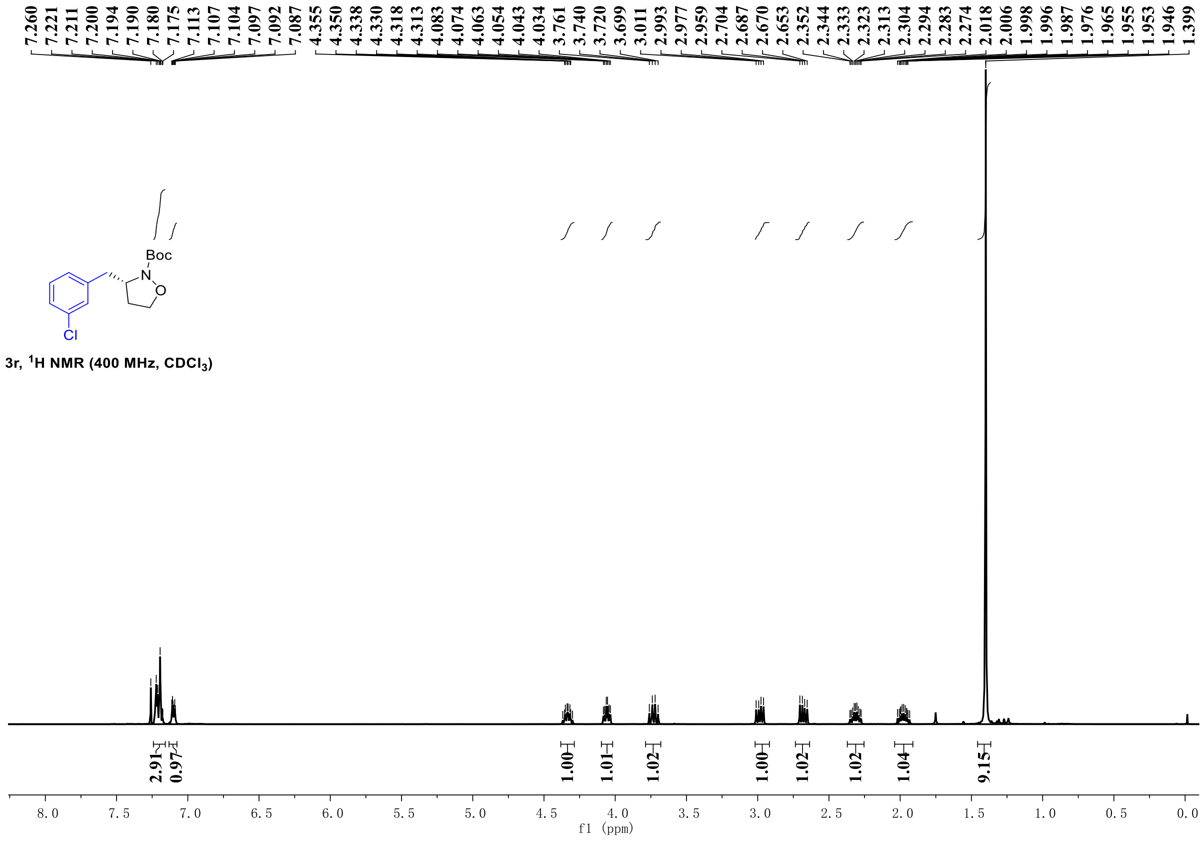
7.260
7.221
7.211
7.200
7.194
7.190
7.180
7.175
7.113
7.107
7.104
7.097
7.092
7.087
4.355
4.350
4.338
4.330
4.318
4.313
4.083
4.074
4.063
4.054
4.043
4.034
3.761
3.740
3.720
3.699
3.011
2.993
2.977
2.959
2.704
2.687
2.670
2.653
2.352
2.344
2.333
2.323
2.313
2.304
2.294
2.283
2.274
2.018
2.006
1.998
1.996
1.987
1.976
1.965
1.955
1.953
1.946
1.399
Boc
N
O
Cl
3r, ^{1}H NMR (400 MHz, $CDCl_3$)
2.91
0.97
1.00
1.01
1.02
1.00
1.02
1.02
1.04
9.15
8.0 7.5 7.0 6.5 6.0 5.5 5.0 4.5 4.0 3.5 3.0 2.5 2.0 1.5 1.0 0.5 0.0
f1 (ppm)

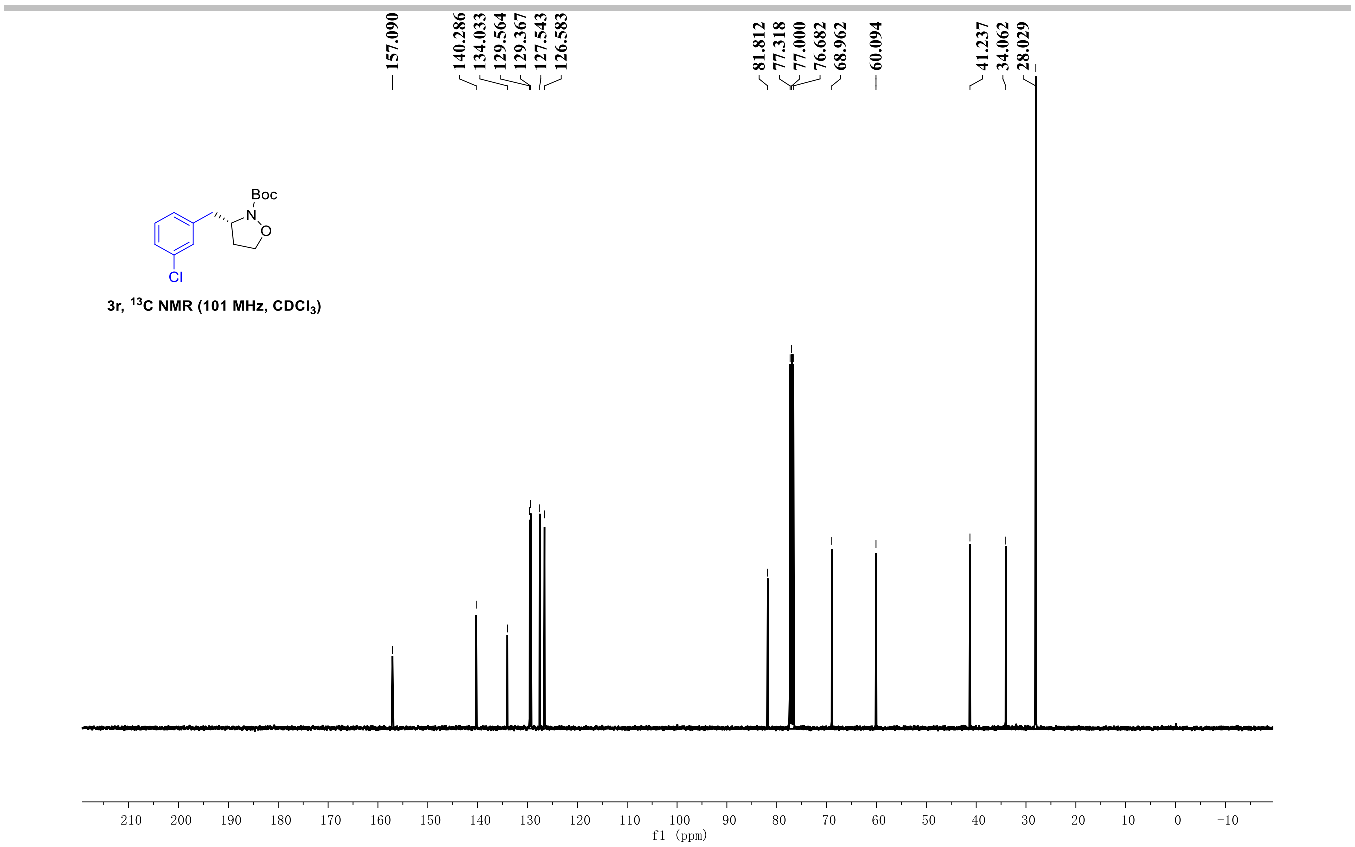

3r, ^{13}C NMR (101 MHz, $CDCl_3$)

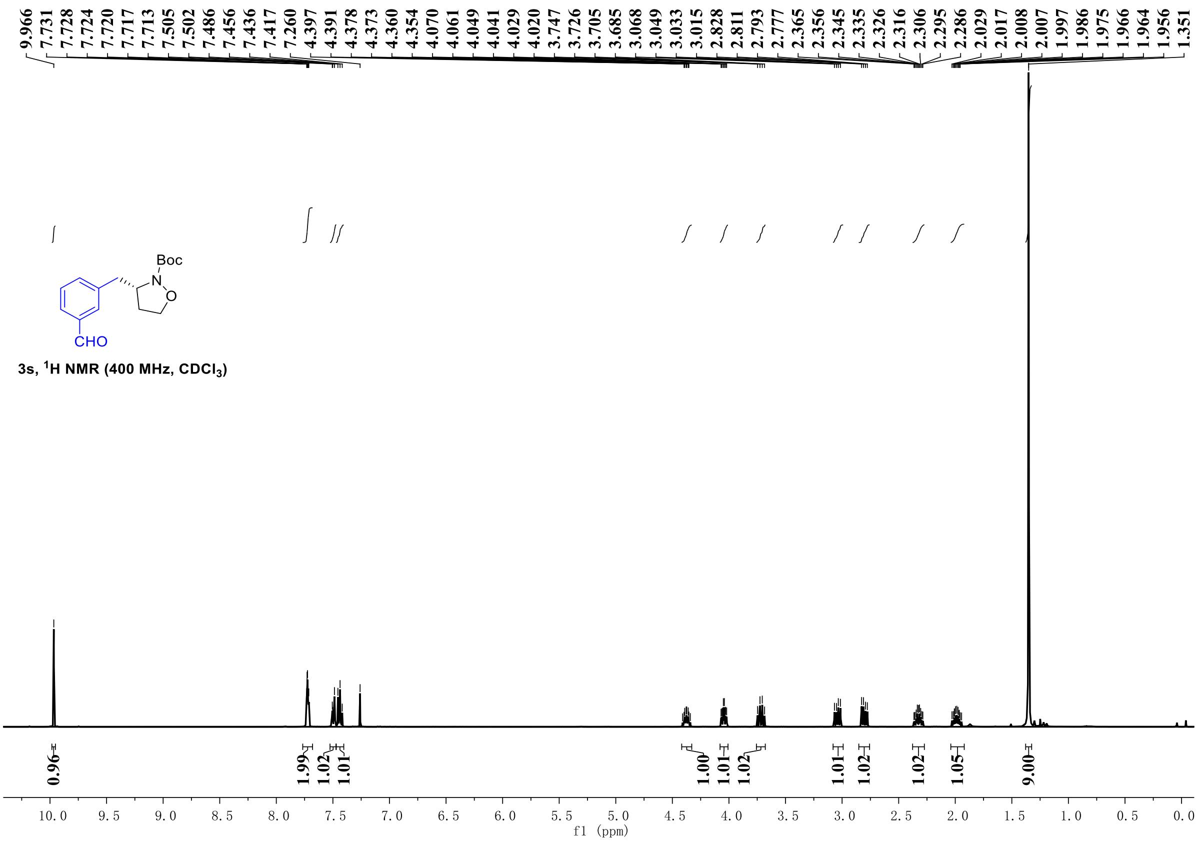

3s, ^{1}H NMR (400 MHz, $CDCl_3$)

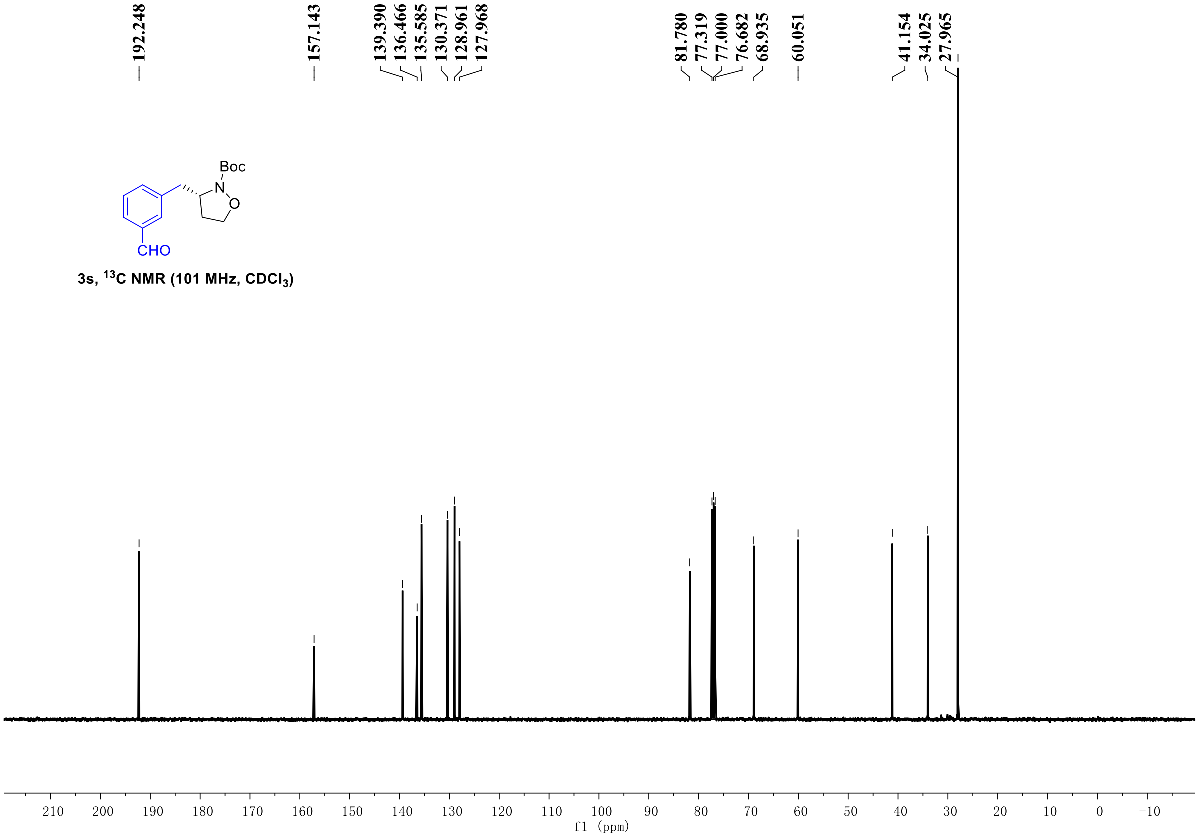
192.248
157.143
139.390
136.466
135.585
130.371
128.961
127.968
81.780
77.319
77.000
76.682
68.935
60.051
41.154
34.025
27.965
Boc
N
O
CHO
3s, 13C NMR (101 MHz, CDCl3)
210 200 190 180 170 160 150 140 130 120 110 100 90 80 70 60 50 40 30 20 10 0 -10
f1 (ppm)

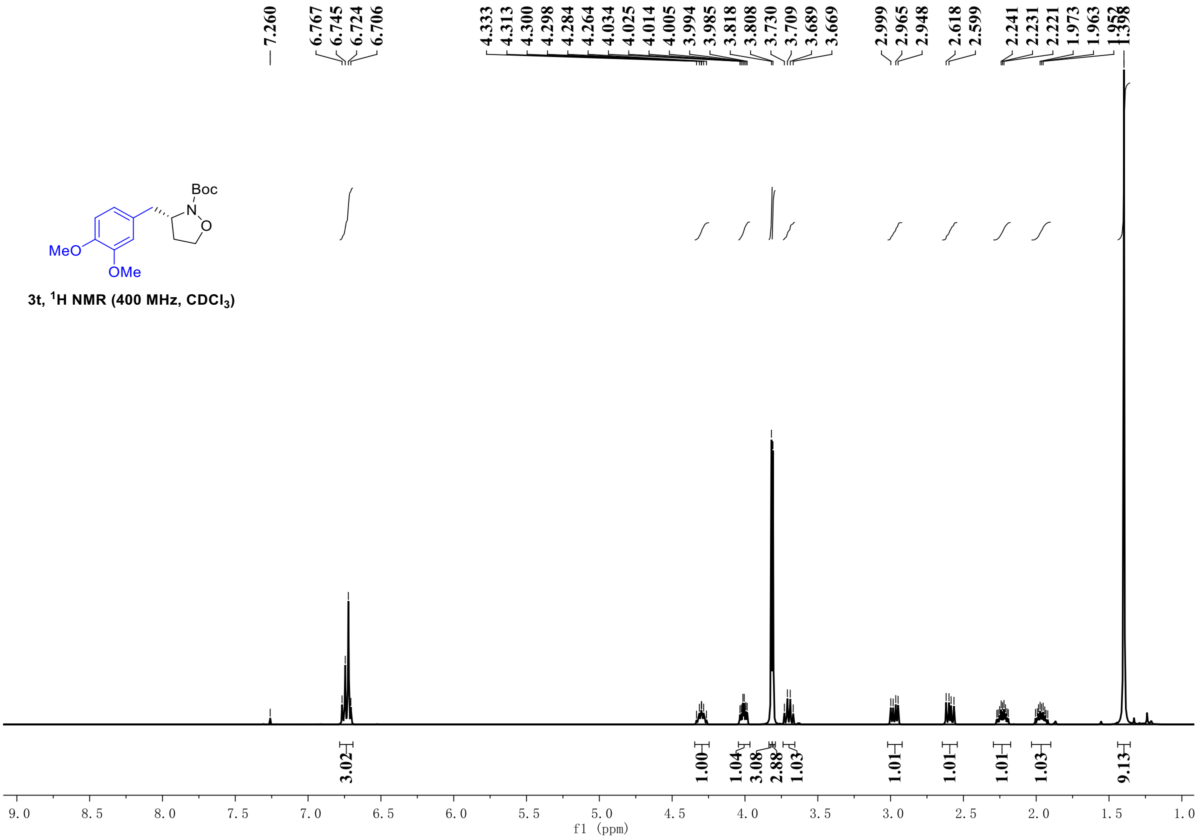
7.260
6.767
6.745
6.724
6.706
4.333
4.313
4.300
4.298
4.284
4.264
4.034
4.025
4.014
4.005
3.994
3.985
3.818
3.808
3.730
3.709
3.689
3.669
2.999
2.965
2.948
2.618
2.599
2.241
2.231
2.221
1.973
1.963
1.952
1.398
Boc
N
O
MeO
OMe
3t, 1H NMR (400 MHz, CDCl3)
3.02
1.00
1.04
3.08
2.88
1.03
1.01
1.01
1.01
1.03
9.13
9.0 8.5 8.0 7.5 7.0 6.5 6.0 5.5 5.0 4.5 4.0 3.5 3.0 2.5 2.0 1.5 1.0
f1 (ppm)

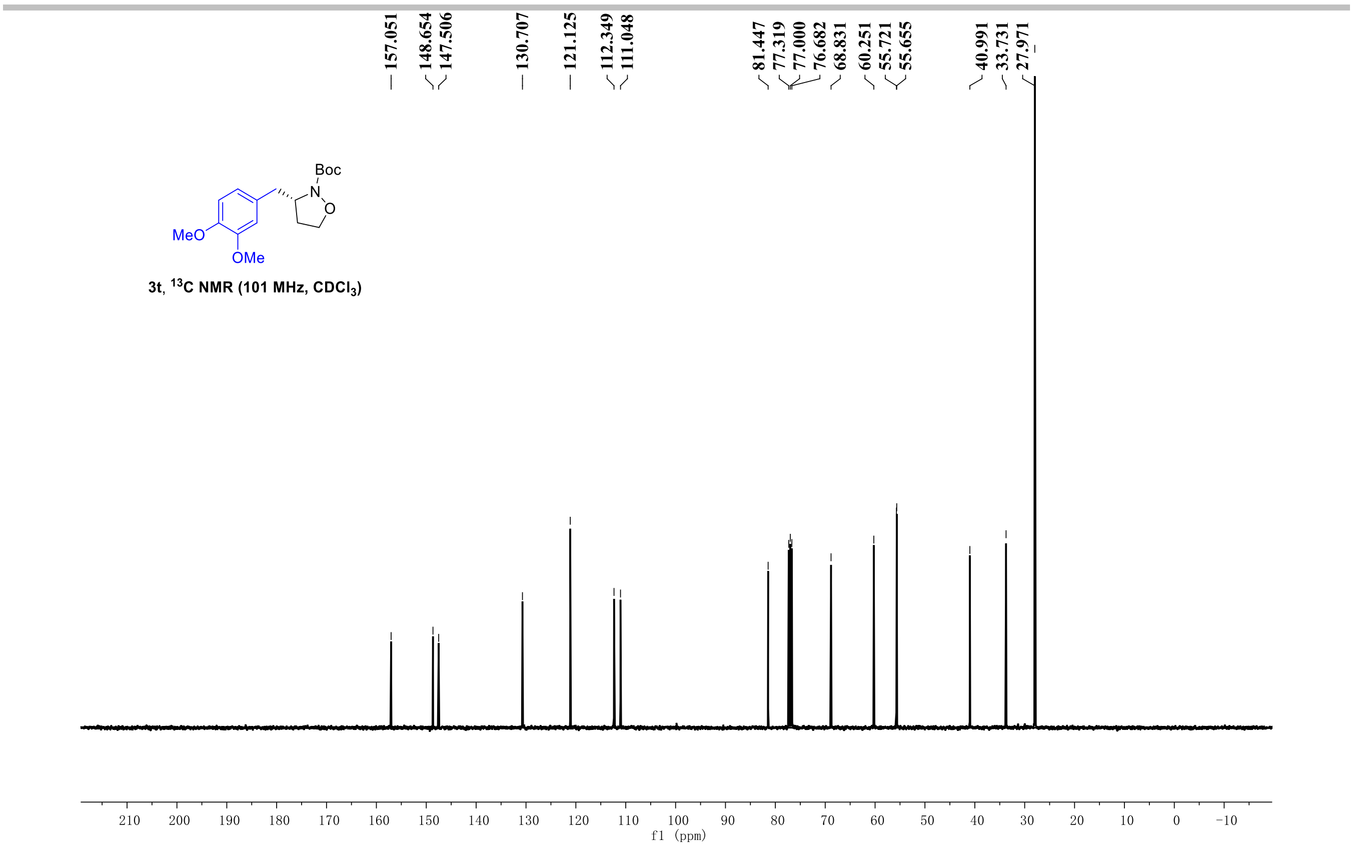

3t, ^{13}C NMR (101 MHz, $CDCl_3$)

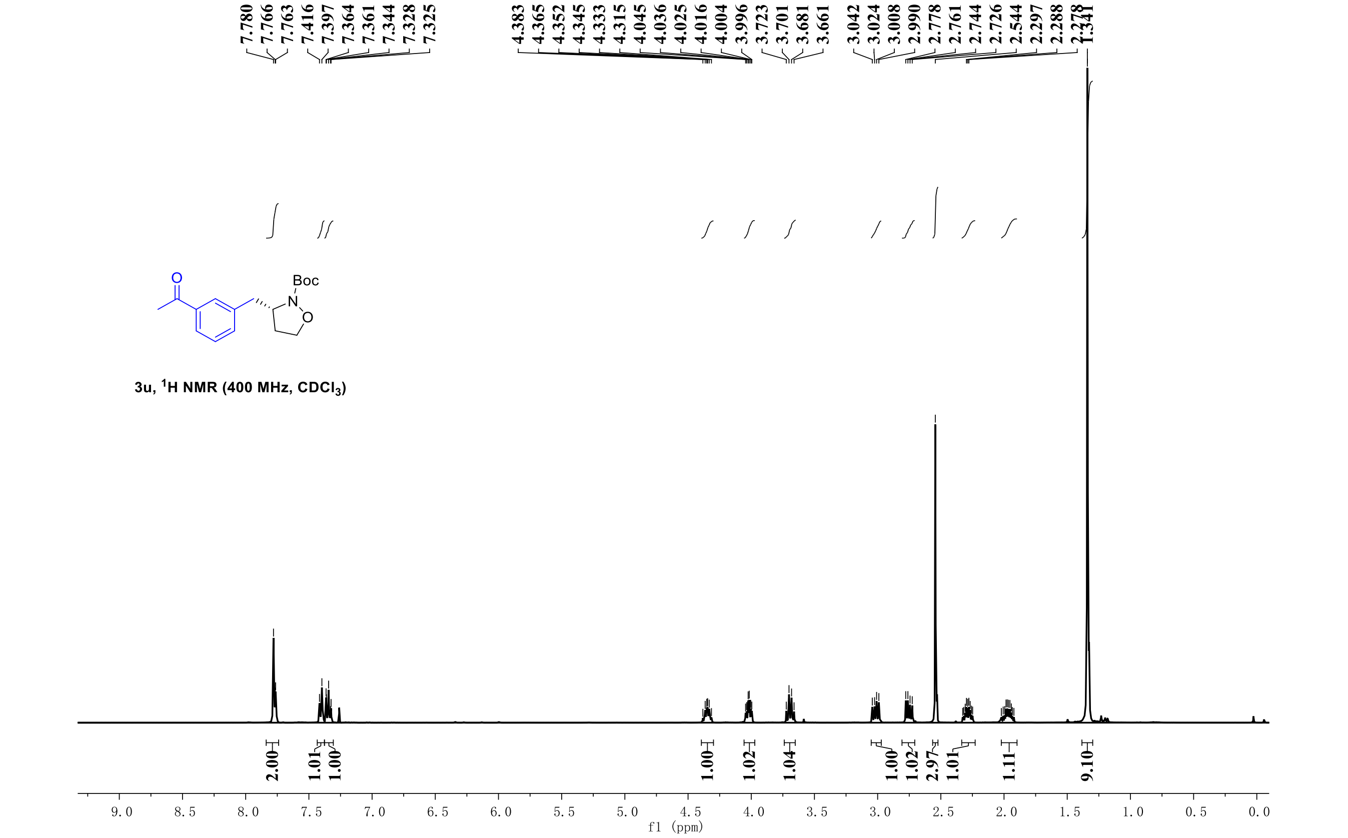

3u, ^{1}H NMR (400 MHz, $CDCl_3$)

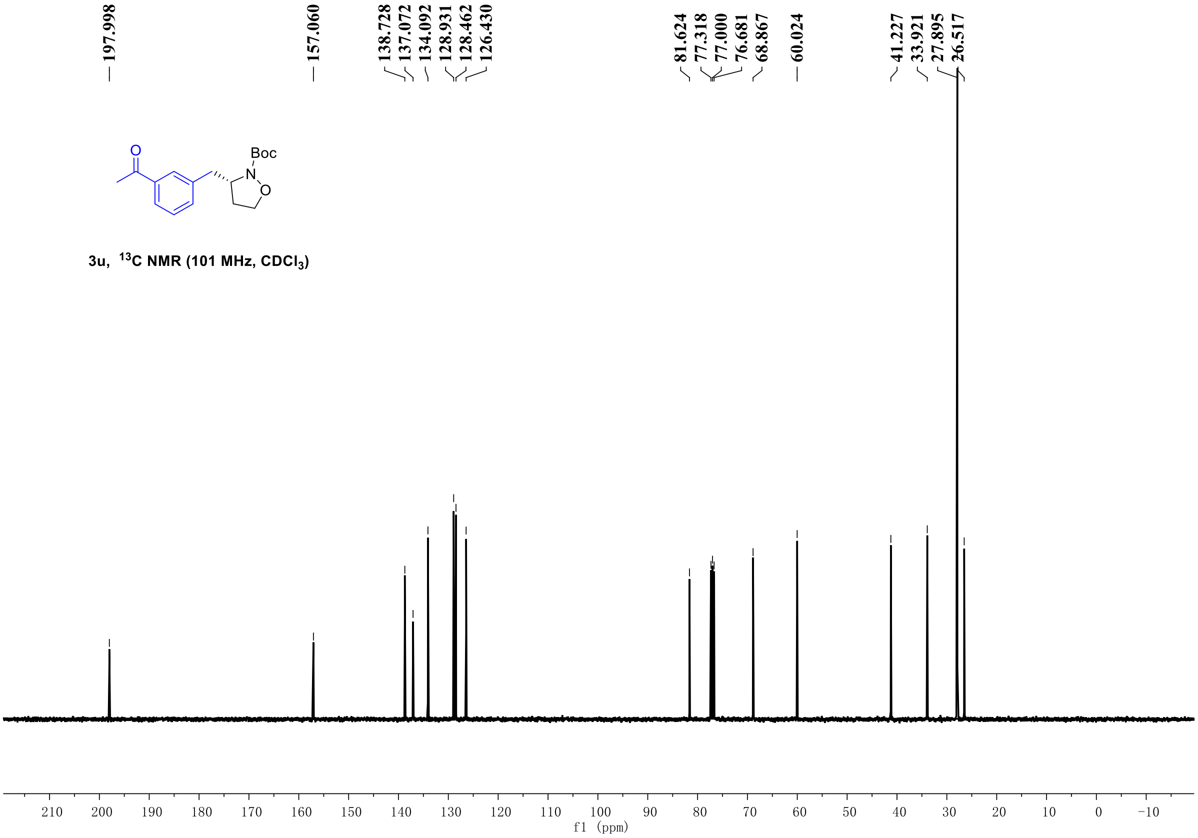

3u, ^{13}C NMR (101 MHz, $CDCl_3$)

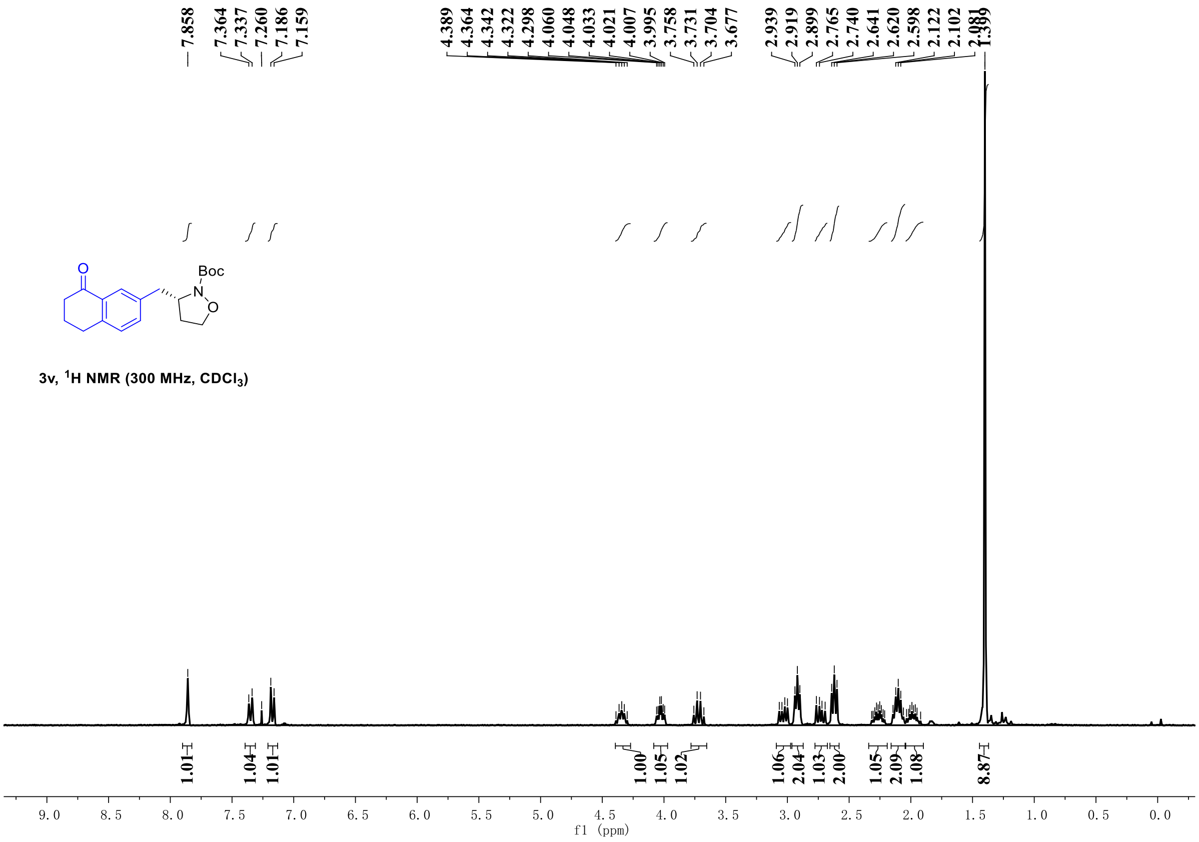

3v, ^{1}H NMR (300 MHz, $CDCl_3$)

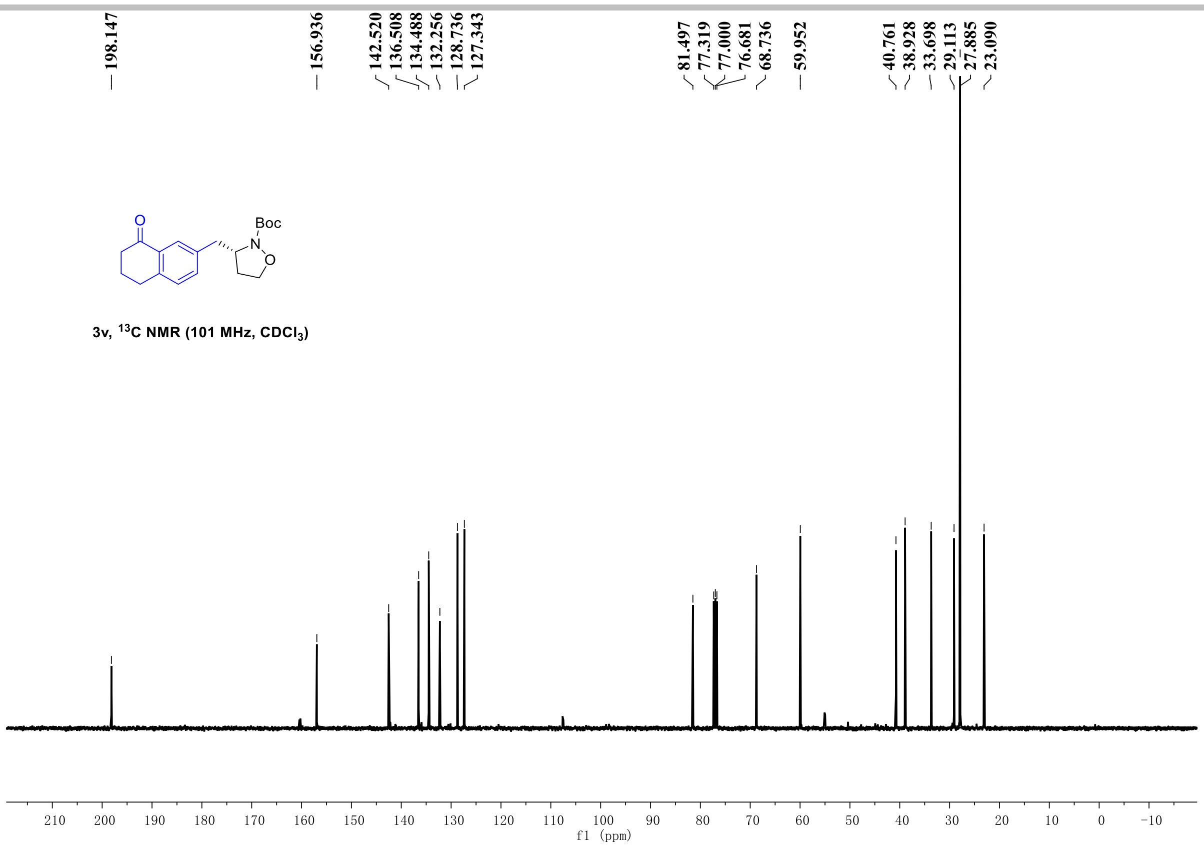

3v, ^{13}C NMR (101 MHz, $CDCl_3$)

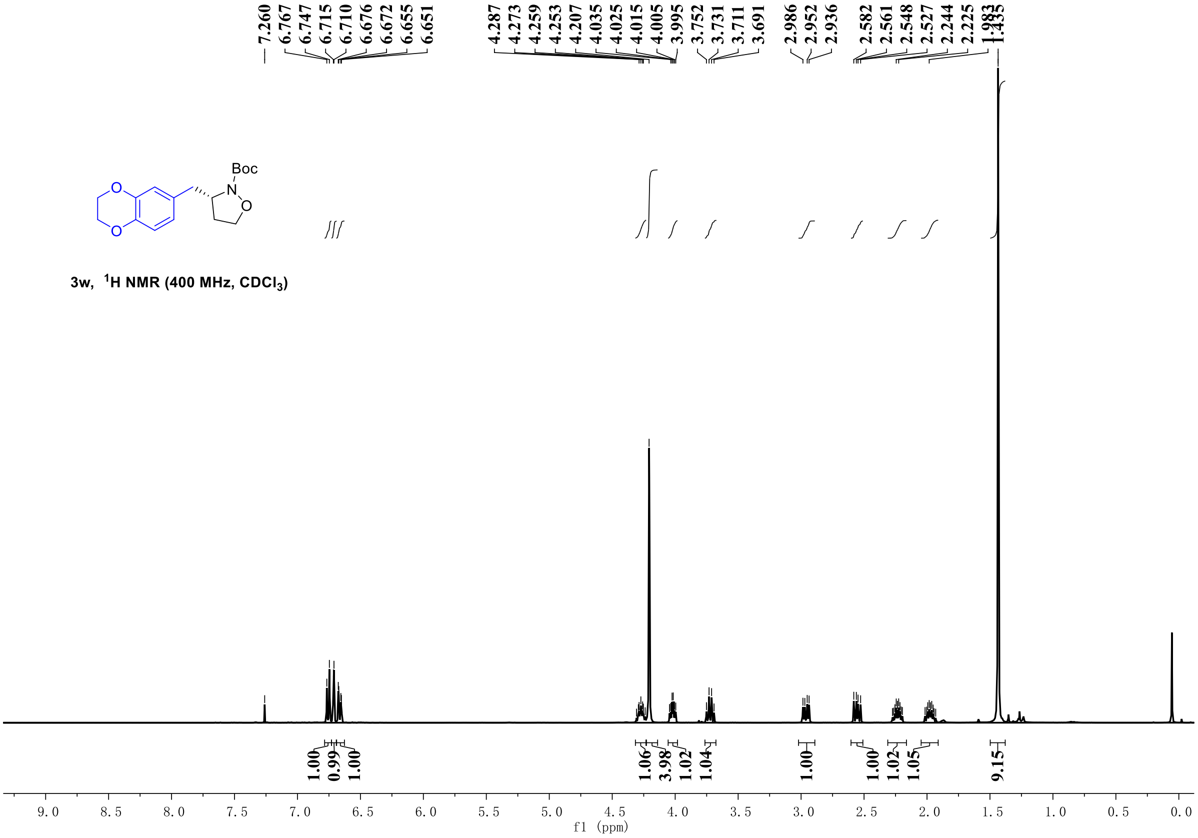

3w, ^{1}H NMR (400 MHz, $CDCl_3$)

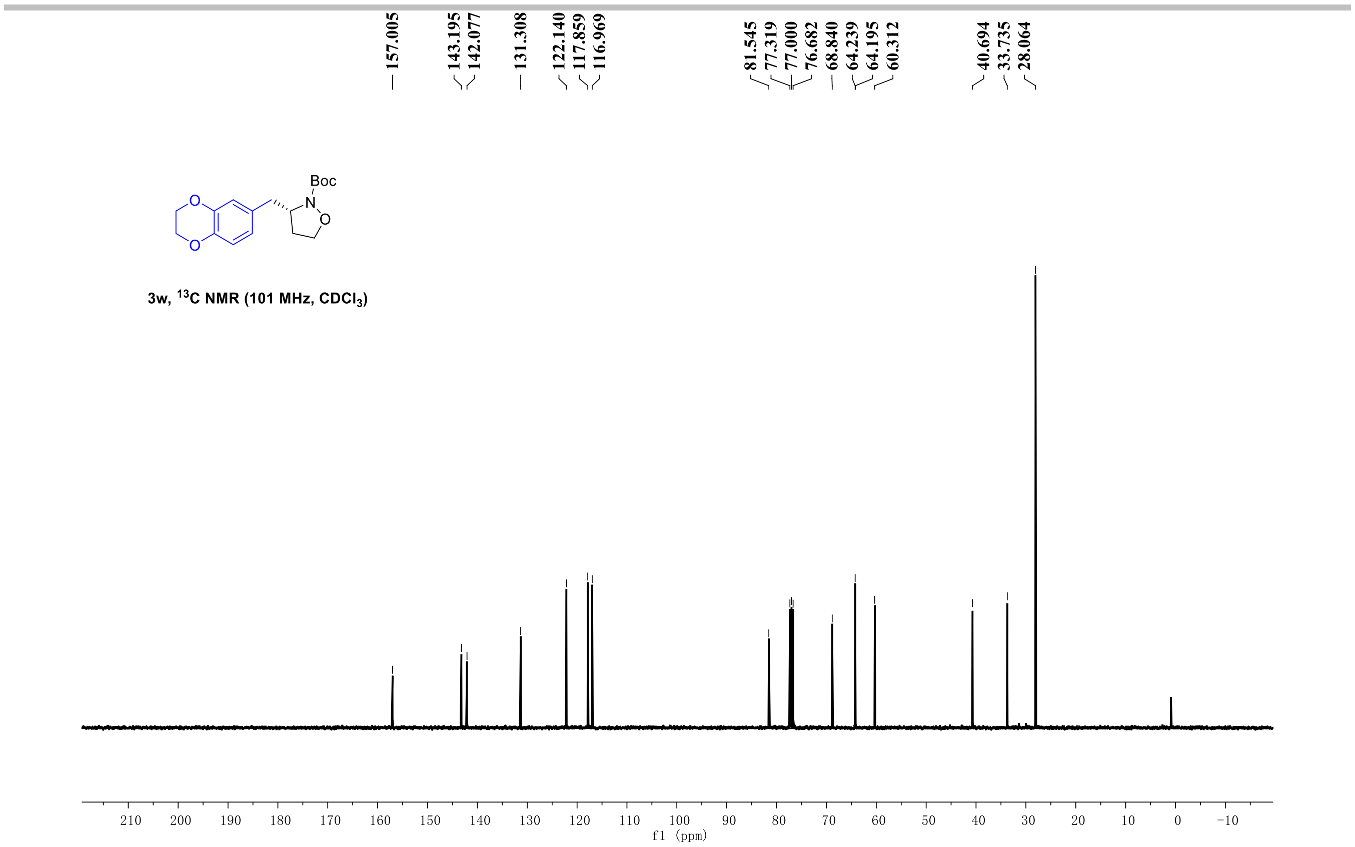
157.005
143.195
142.077
131.308
122.140
117.859
116.969
81.545
77.319
77.000
76.682
68.840
64.239
64.195
60.312
40.694
33.735
28.064
Boc
N
O
3w, ^{13}C NMR (101 MHz, CDCl$_3$)
f1 (ppm)

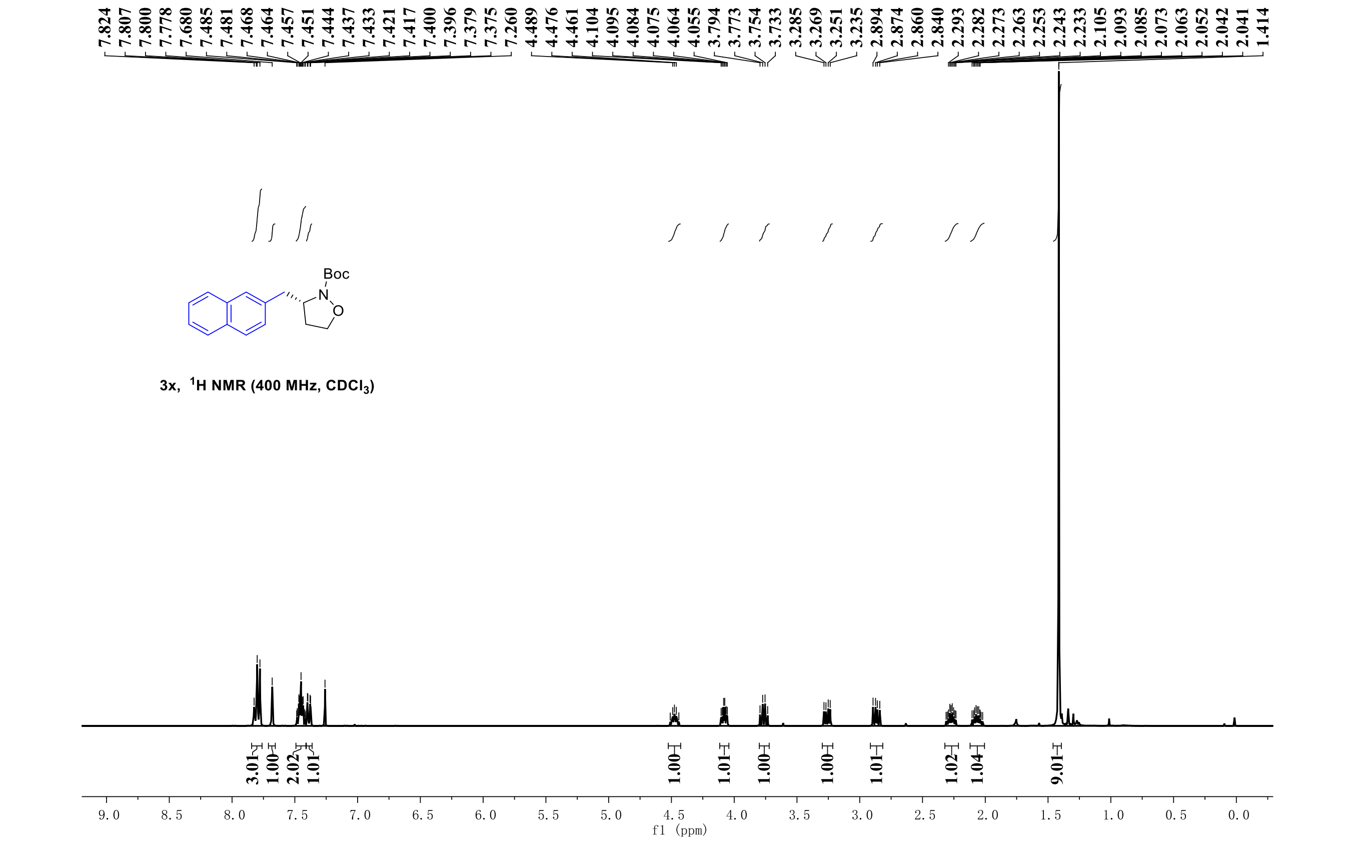
7.824
7.807
7.800
7.778
7.680
7.485
7.481
7.468
7.464
7.457
7.451
7.444
7.437
7.433
7.421
7.417
7.400
7.396
7.379
7.375
7.260
4.489
4.476
4.461
4.104
4.095
4.084
4.075
4.064
4.055
3.794
3.773
3.754
3.733
3.285
3.269
3.251
3.235
2.894
2.874
2.860
2.840
2.293
2.282
2.273
2.263
2.253
2.243
2.233
2.105
2.093
2.085
2.073
2.063
2.052
2.042
2.041
1.414
Boc
N
O
3x, ^{1}H NMR (400 MHz, CDCl$_3$)
3.01
1.00
2.02
1.01
1.00
1.01
1.00
1.00
1.01
1.02
1.04
9.01
f1 (ppm)

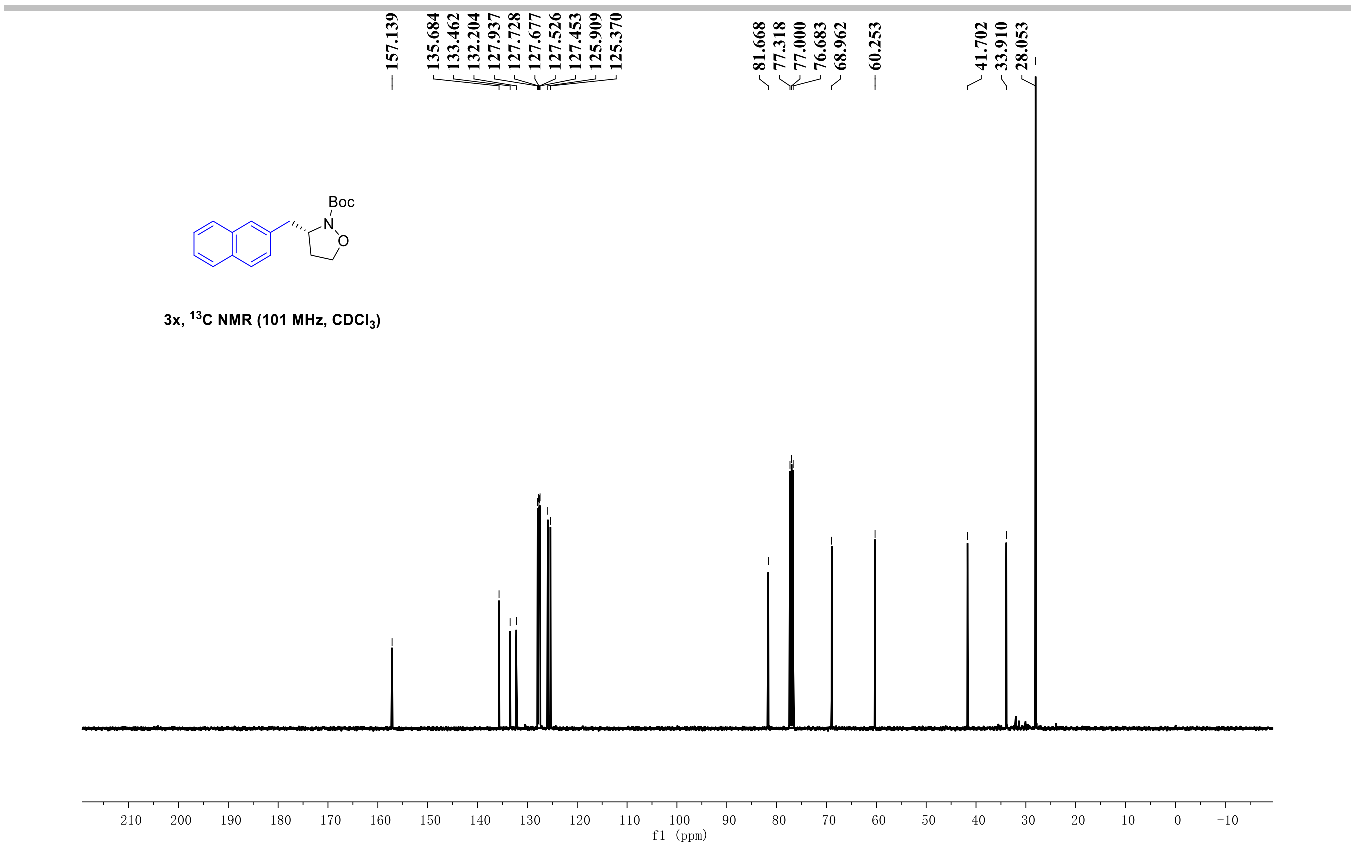

3x, ^{13}C NMR (101 MHz, $CDCl_3$)

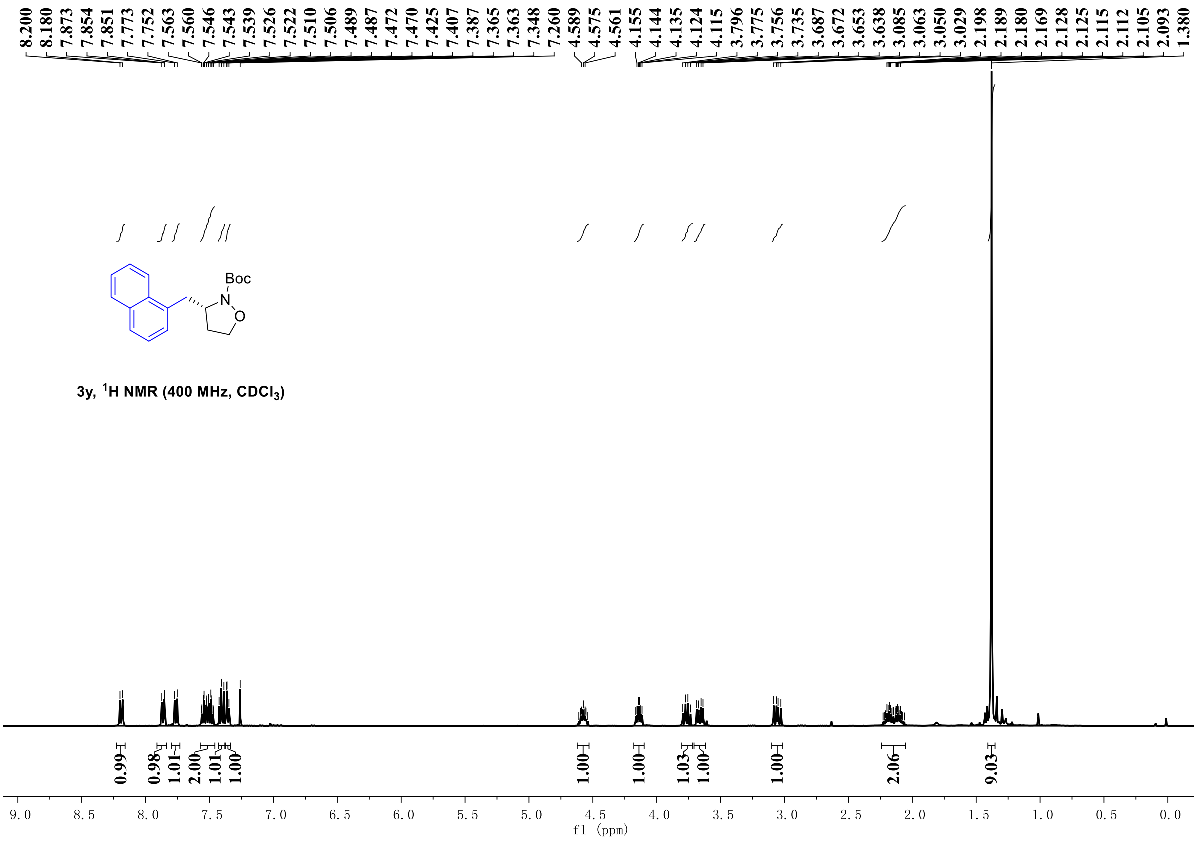

3y, ^{1}H NMR (400 MHz, $CDCl_3$)

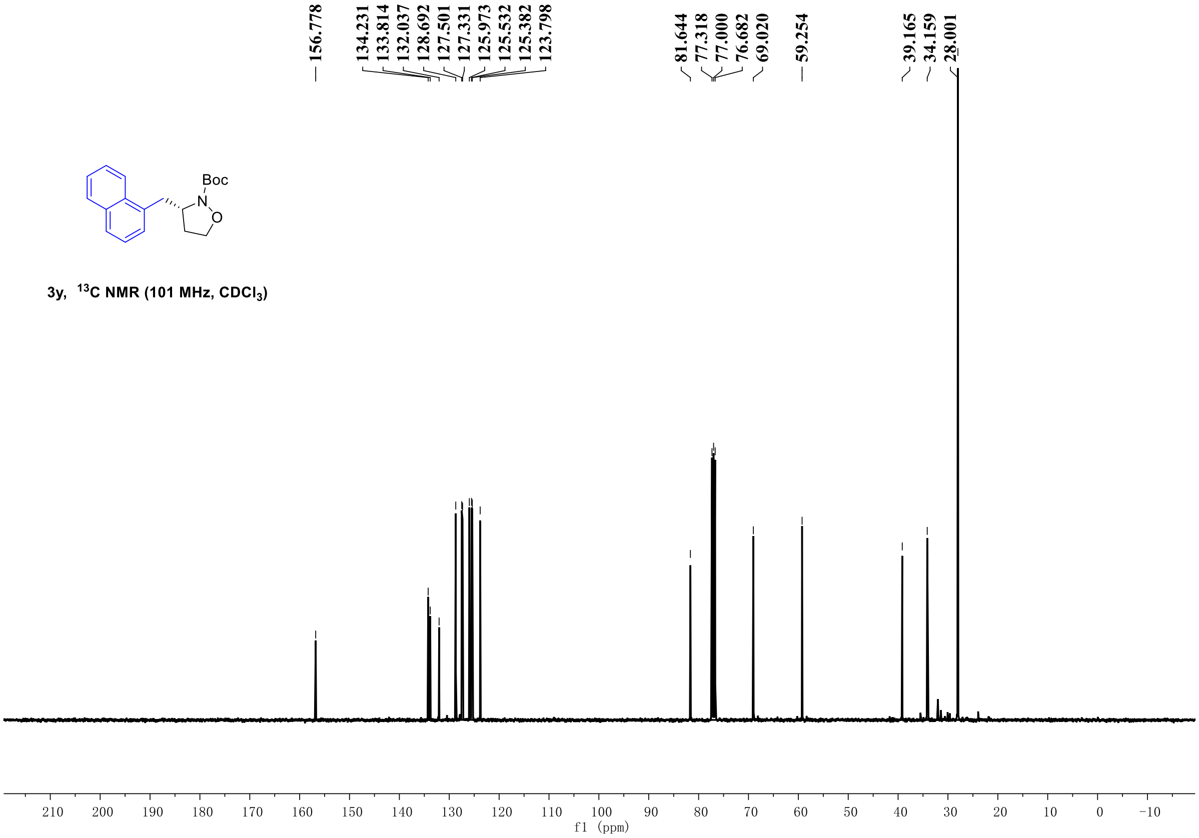

3y, ^{13}C NMR (101 MHz, CDCl$_3$)

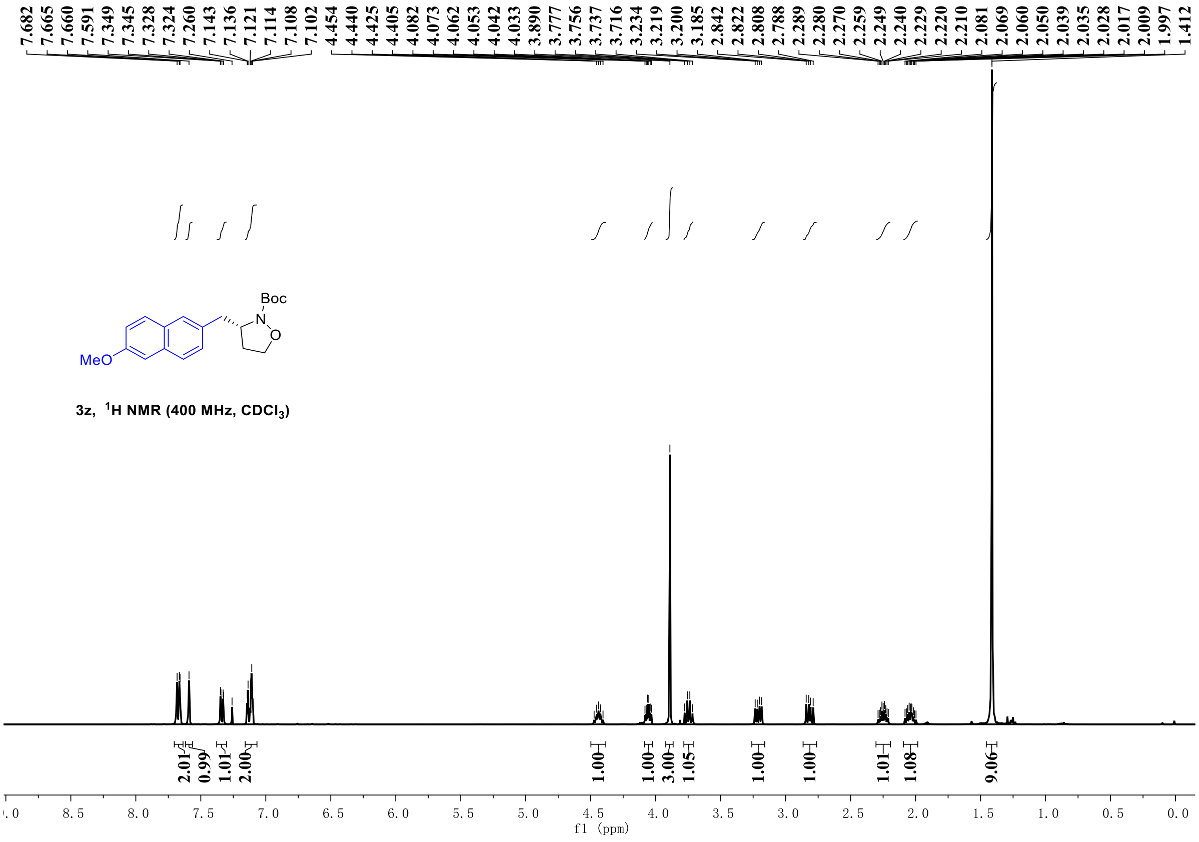

3z, ^{1}H NMR (400 MHz, CDCl$_3$)

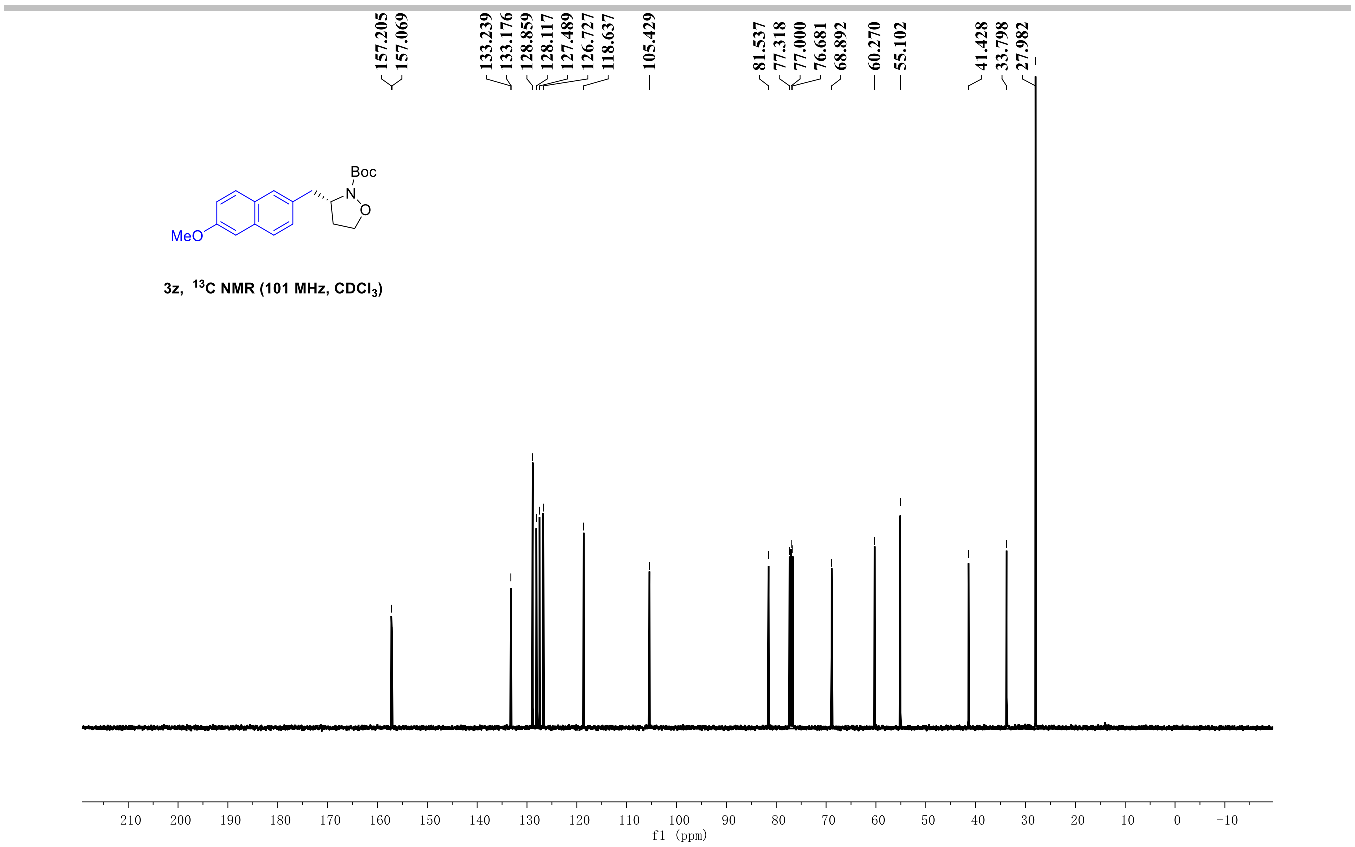
157.205
157.069
133.239
133.176
128.859
128.117
127.489
126.727
118.637
105.429
81.537
77.318
77.000
76.681
68.892
60.270
55.102
41.428
33.798
27.982
Boc
N
O
MeO
3z, ^{13}C NMR (101 MHz, CDCl$_3$)
210 200 190 180 170 160 150 140 130 120 110 100 90 80 70 60 50 40 30 20 10 0 −10
f1 (ppm)

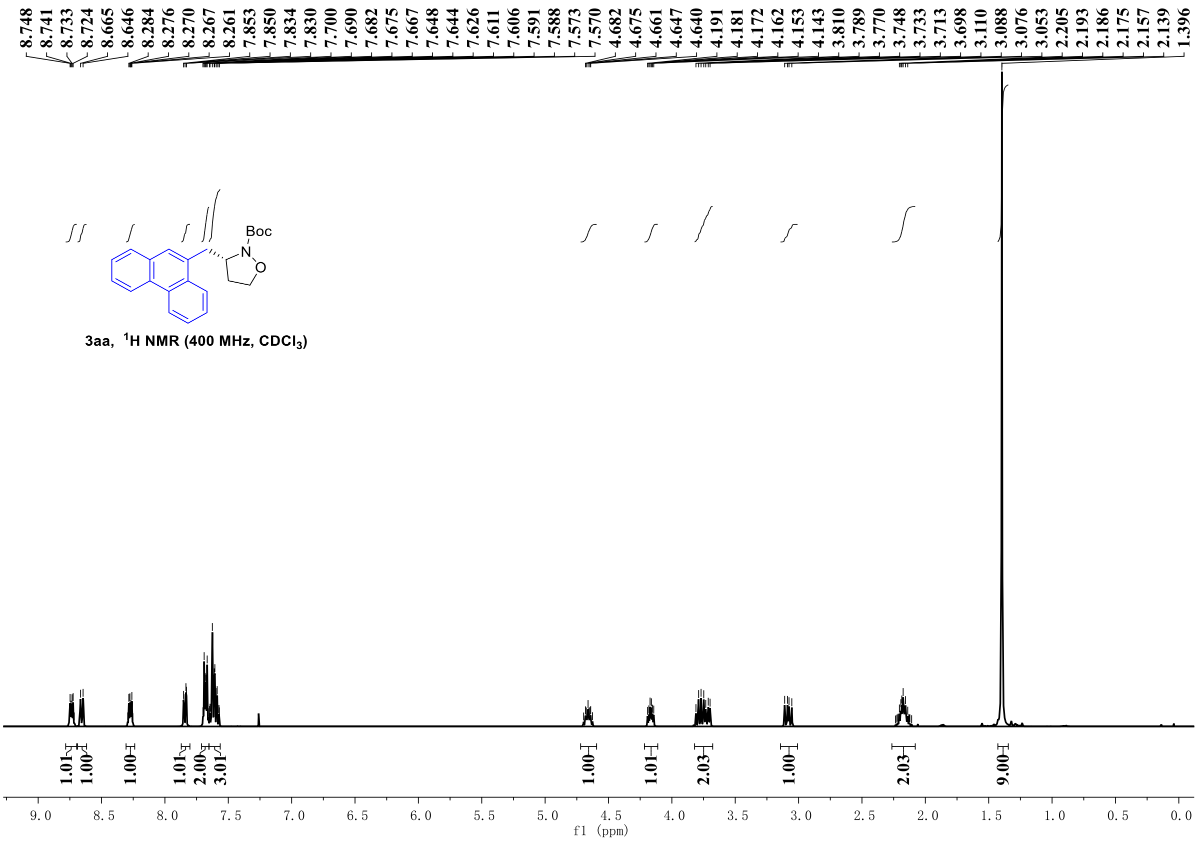
8.748
8.741
8.733
8.724
8.665
8.646
8.284
8.276
8.270
8.267
8.261
7.853
7.850
7.834
7.830
7.700
7.690
7.682
7.675
7.667
7.648
7.644
7.626
7.611
7.606
7.591
7.588
7.573
7.570
4.682
4.675
4.661
4.647
4.640
4.191
4.181
4.172
4.162
4.153
4.143
3.810
3.789
3.770
3.748
3.733
3.713
3.698
3.110
3.088
3.076
3.053
2.205
2.193
2.186
2.175
2.157
2.139
1.396
Boc
N
O
3aa, ^{1}H NMR (400 MHz, CDCl$_3$)
1.01
1.00
1.00
1.01
2.00
3.01
1.00
1.01
2.03
1.00
2.03
9.00
9.0 8.5 8.0 7.5 7.0 6.5 6.0 5.5 5.0 4.5 4.0 3.5 3.0 2.5 2.0 1.5 1.0 0.5 0.0
f1 (ppm)

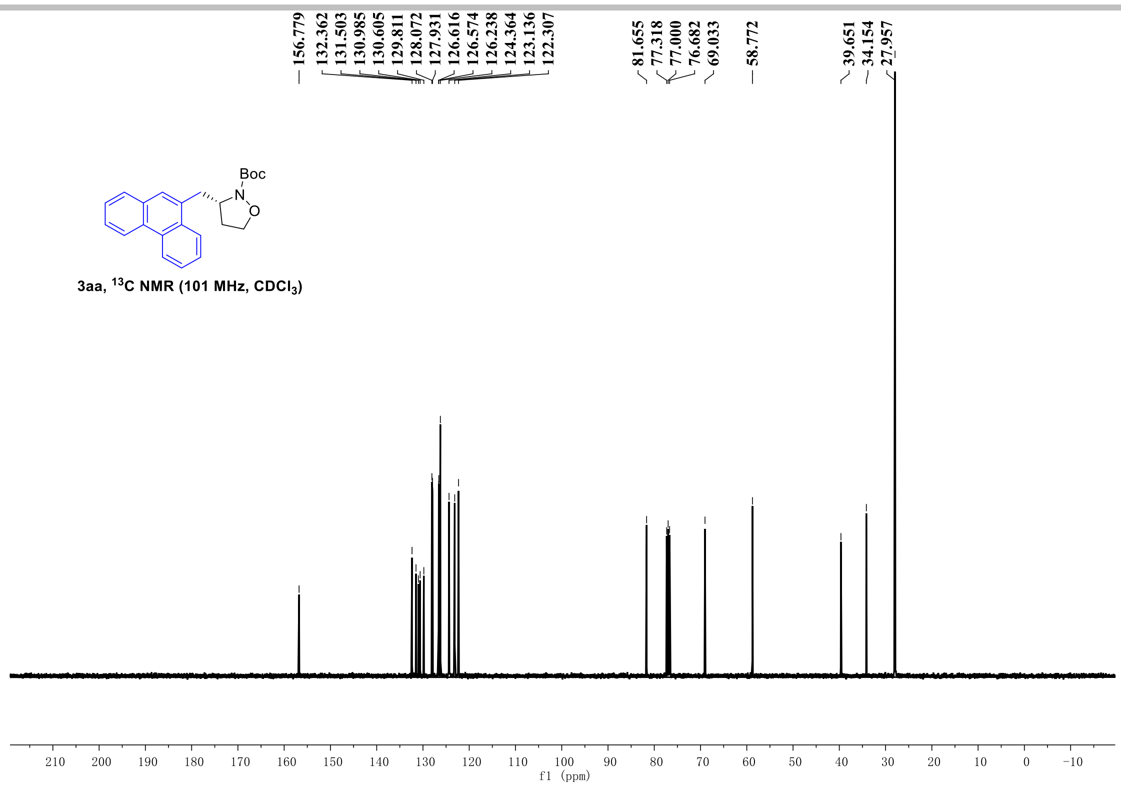

3aa, ^{13}C NMR (101 MHz, $CDCl_3$)

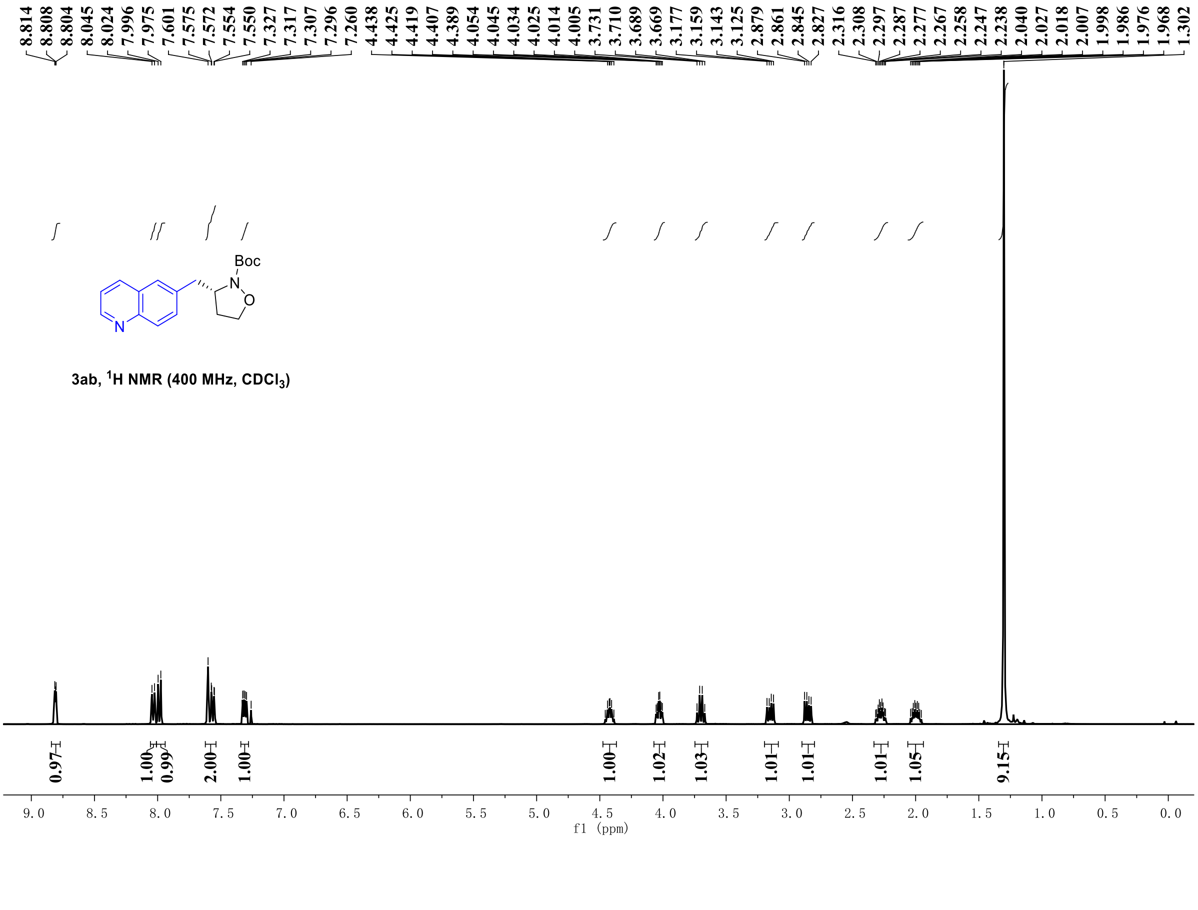

3ab, ^{1}H NMR (400 MHz, $CDCl_3$)

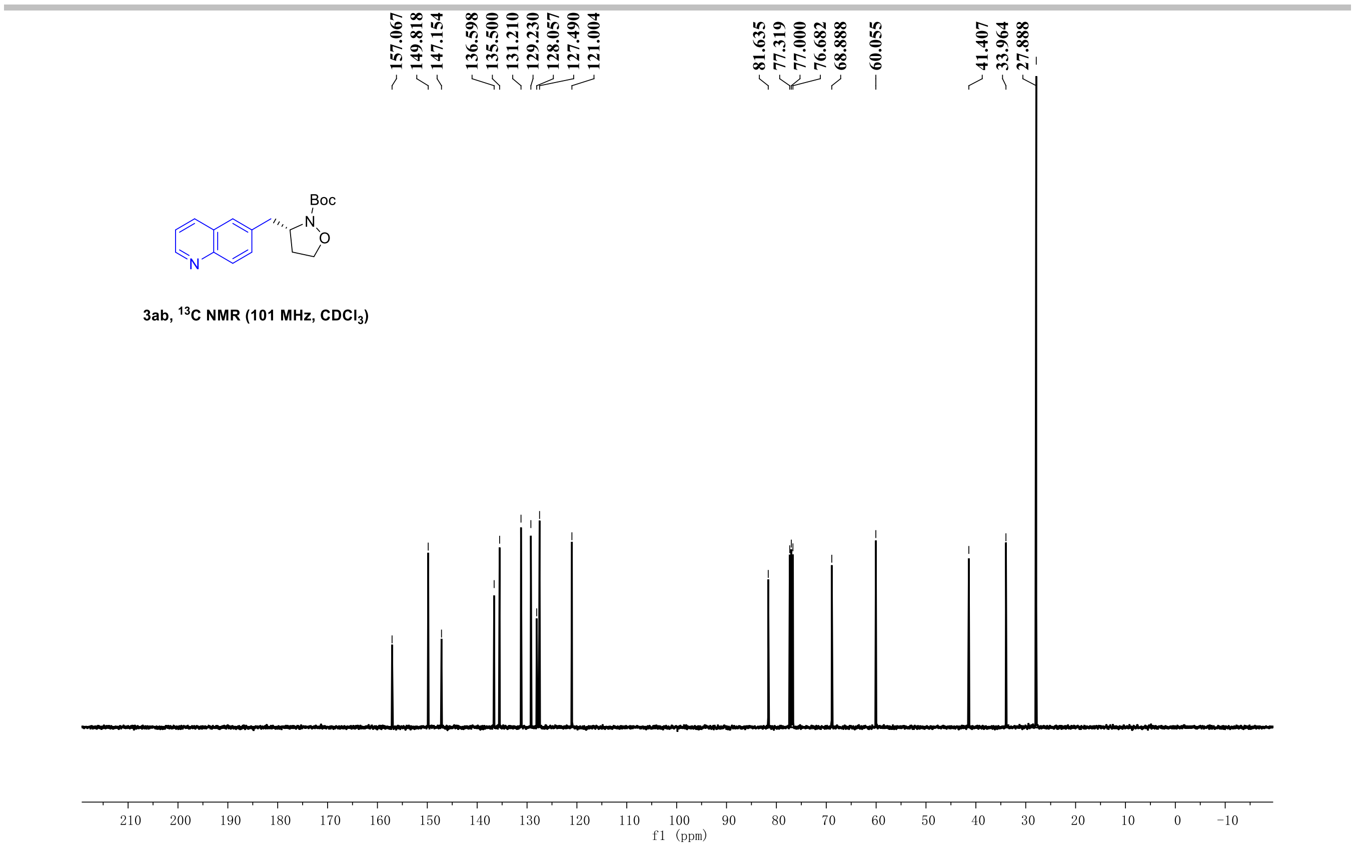

3ab, ^{13}C NMR (101 MHz, $CDCl_3$)

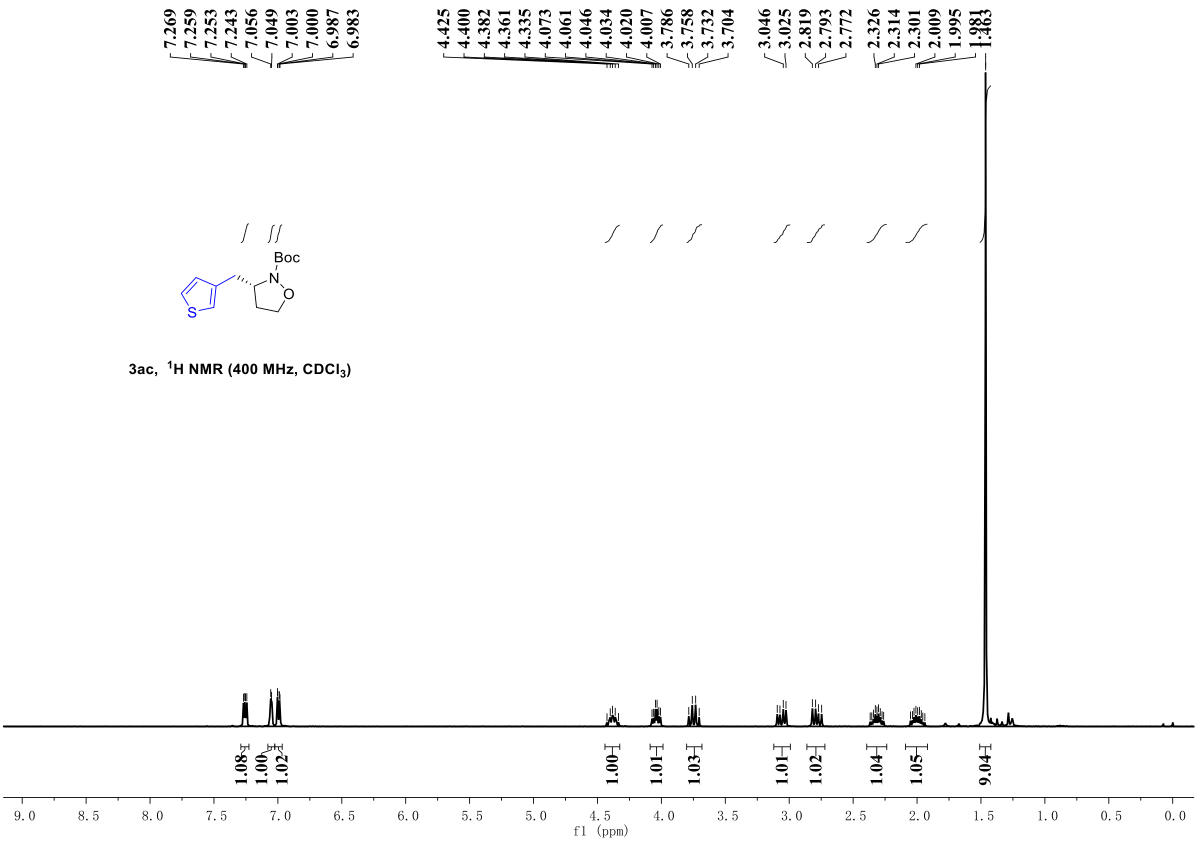

3ac, ^{1}H NMR (400 MHz, $CDCl_3$)

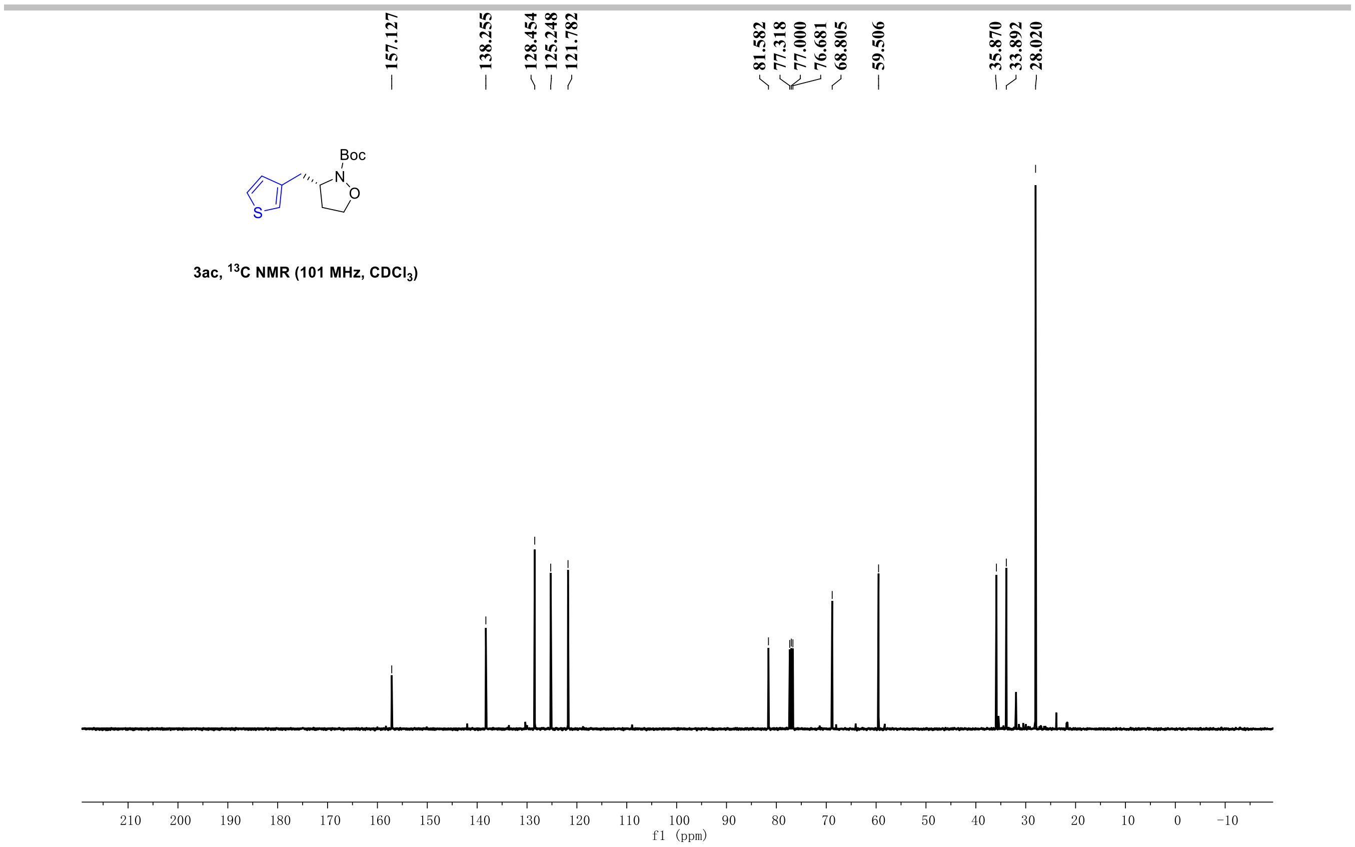
3ac, ^{13}C NMR (101 MHz, CDCl$_3$)
Boc
N
O
S
157.127
138.255
128.454
125.248
121.782
81.582
77.318
77.000
76.681
68.805
59.506
35.870
33.892
28.020
f1 (ppm)

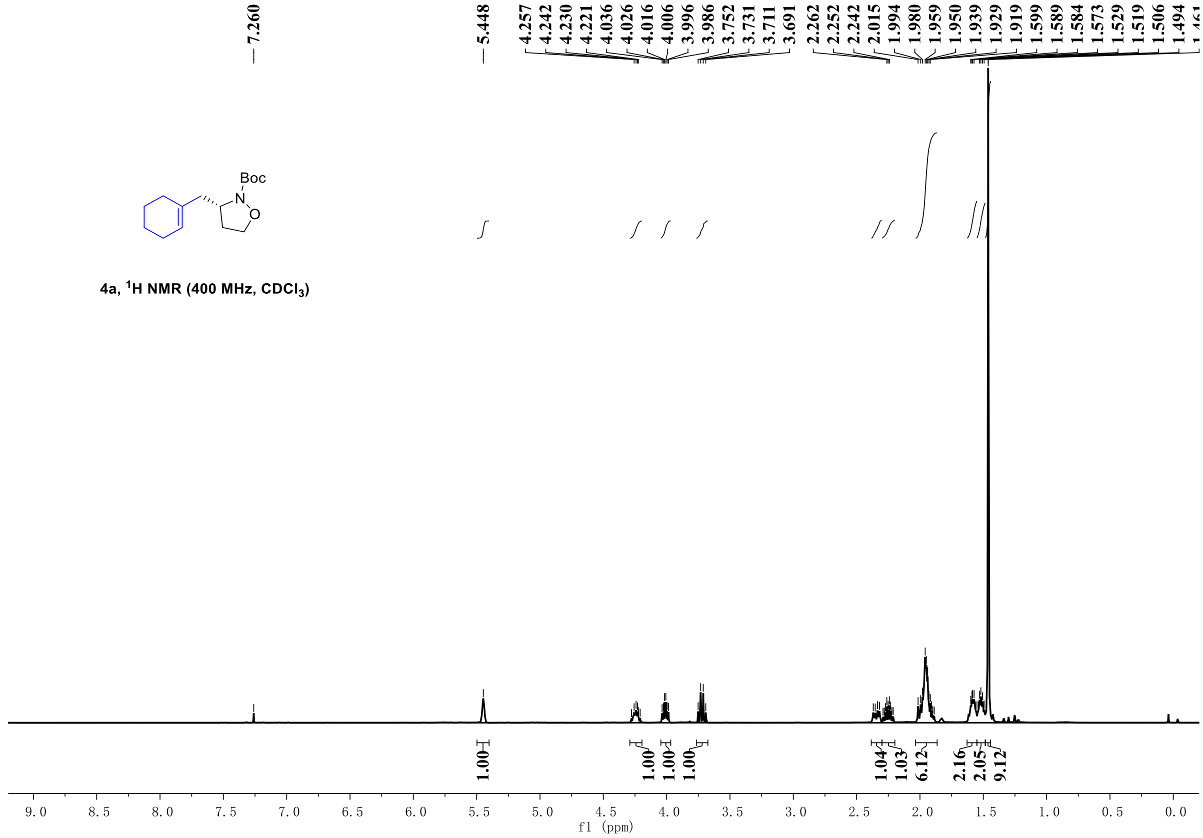
4a, ^{1}H NMR (400 MHz, CDCl$_3$)
Boc
N
O
7.260
5.448
4.257
4.242
4.230
4.221
4.036
4.026
4.016
4.006
3.996
3.986
3.752
3.731
3.711
3.691
2.262
2.252
2.242
2.015
1.994
1.980
1.959
1.950
1.939
1.929
1.919
1.599
1.589
1.584
1.573
1.529
1.519
1.506
1.494
1.00
1.00
1.00
1.00
1.04
1.03
6.12
2.16
2.05
9.12
f1 (ppm)

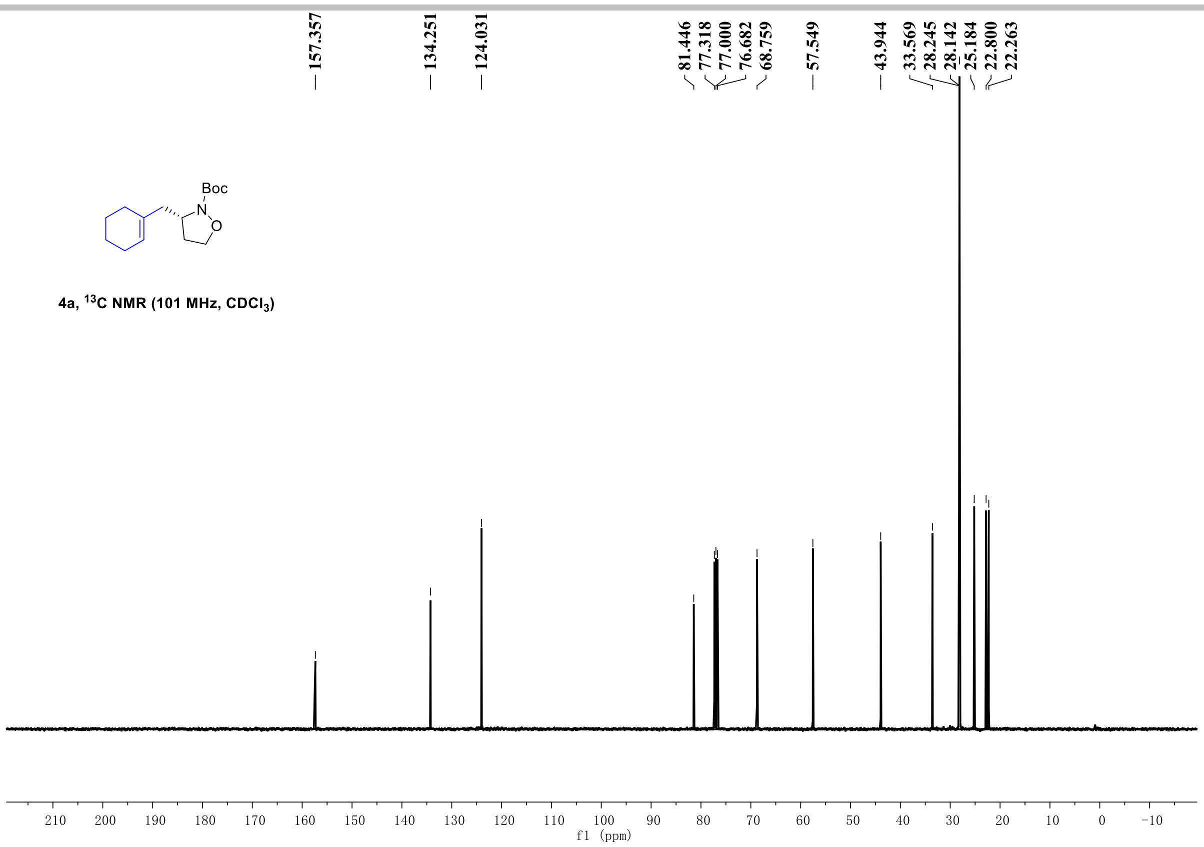

4a, ^{13}C NMR (101 MHz, CDCl$_3$)

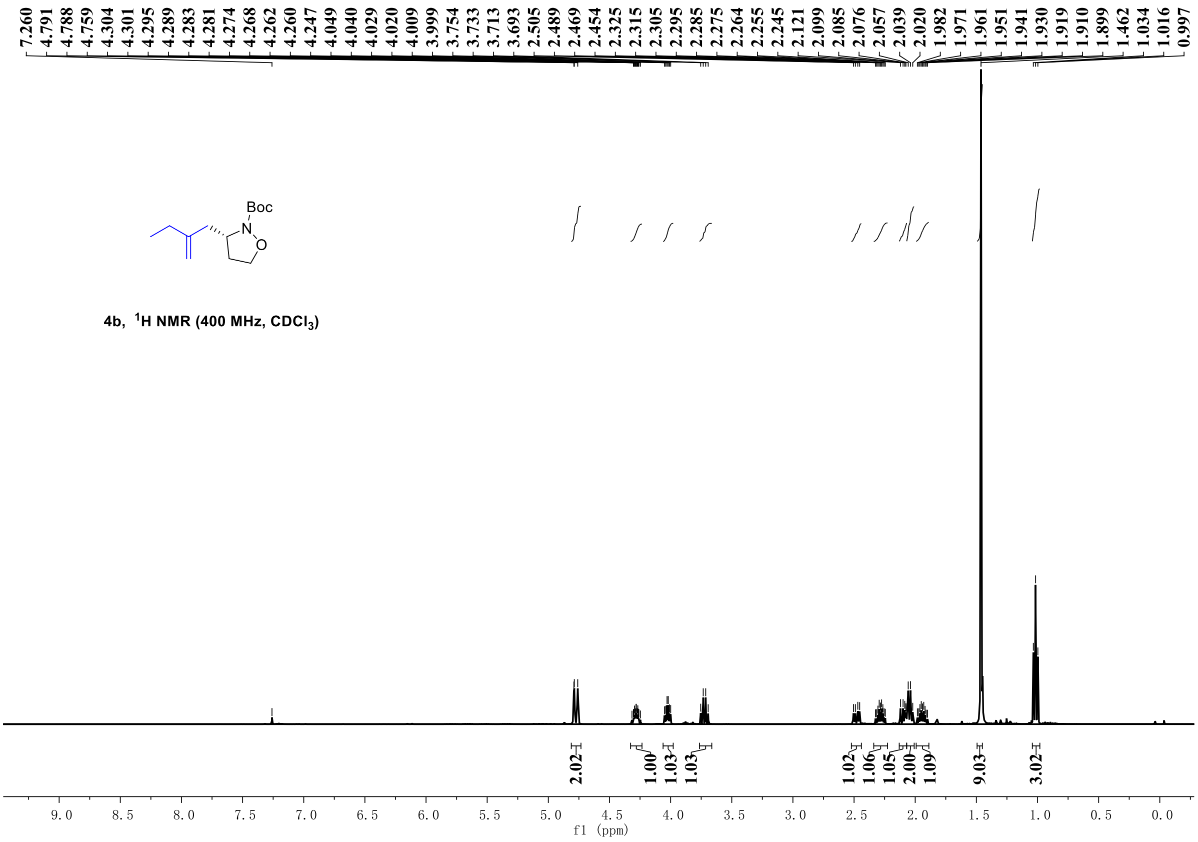

4b, ^{1}H NMR (400 MHz, CDCl$_3$)

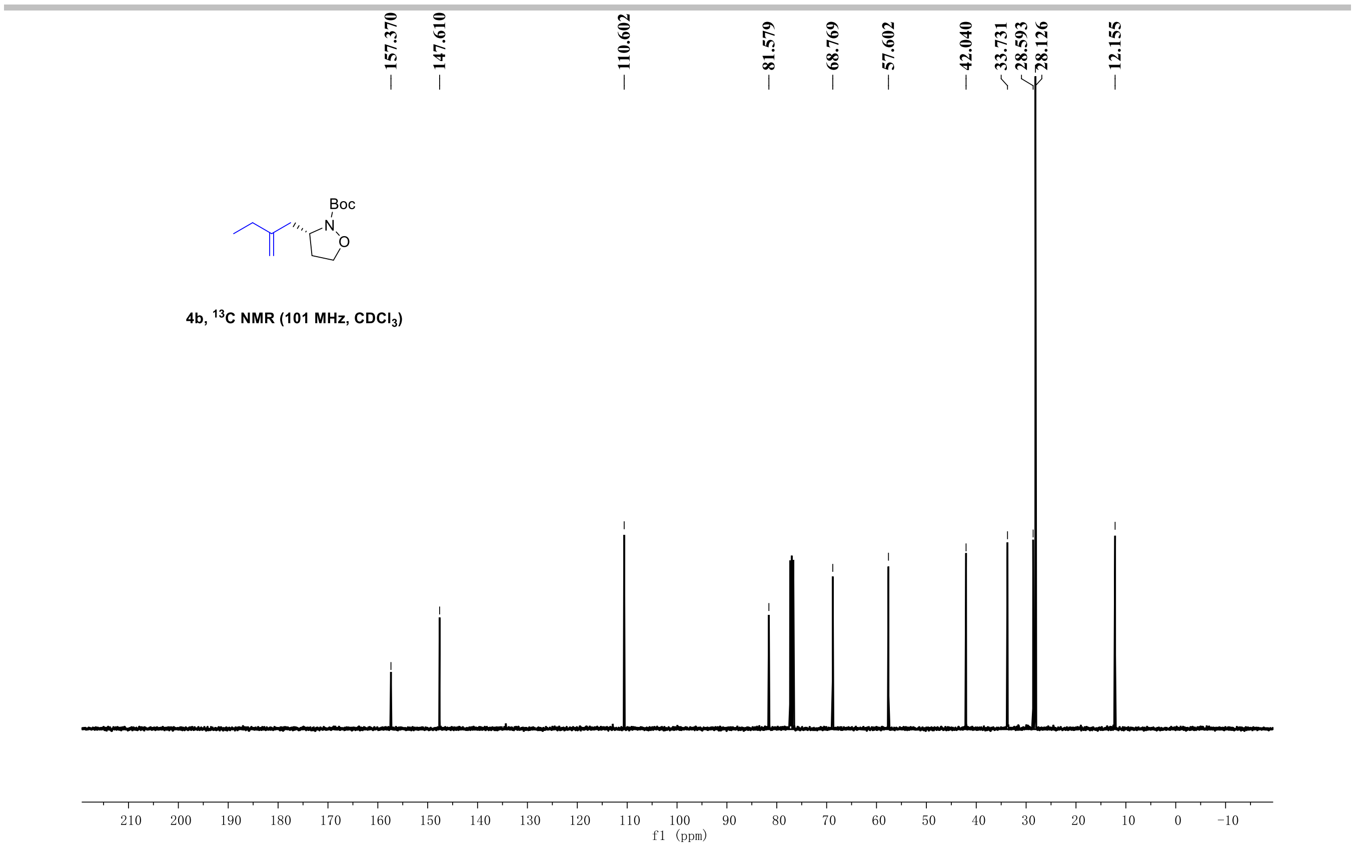
157.370
147.610
110.602
81.579
68.769
57.602
42.040
33.731
28.593
28.126
12.155
Boc
N
O
4b, ^{13}C NMR (101 MHz, CDCl$_3$)
210 200 190 180 170 160 150 140 130 120 110 100 90 80 70 60 50 40 30 20 10 0 −10
f1 (ppm)

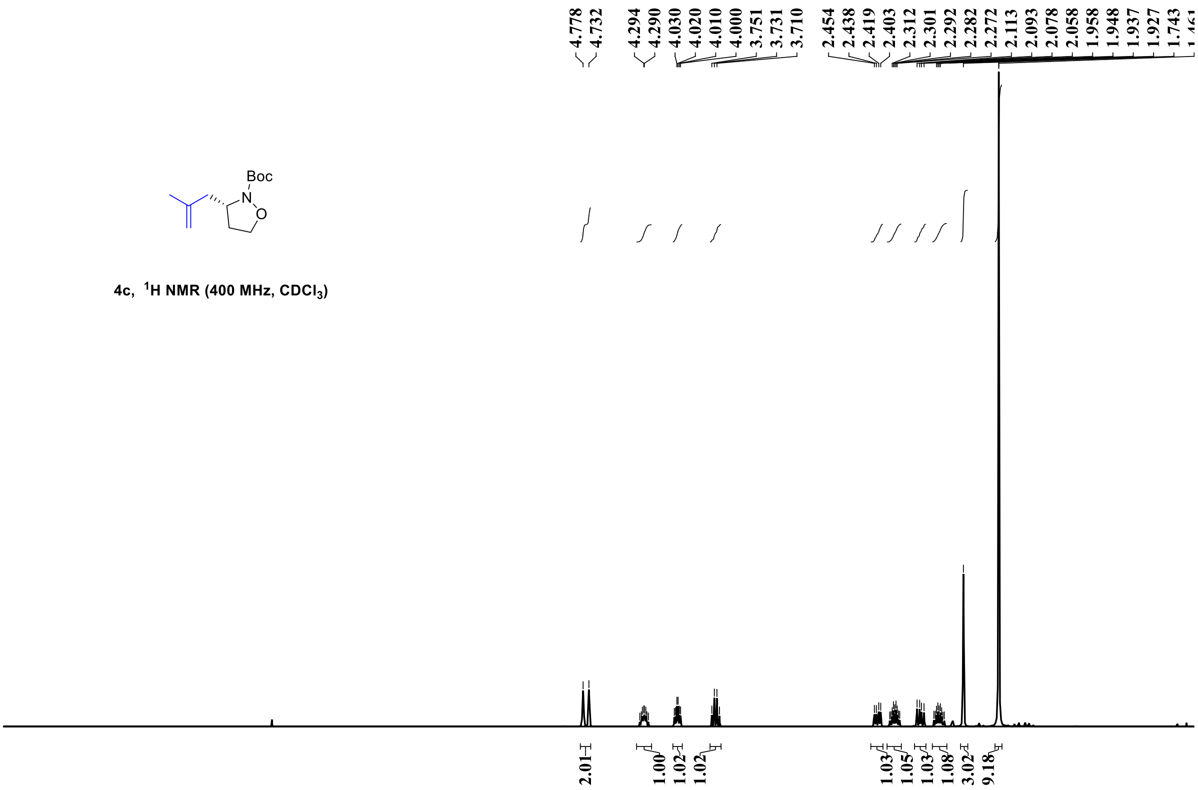
4.778
4.732
4.294
4.290
4.030
4.020
4.010
4.000
3.751
3.731
3.710
2.454
2.438
2.419
2.403
2.312
2.301
2.292
2.282
2.272
2.113
2.093
2.078
2.058
1.958
1.948
1.937
1.927
1.743
Boc
N
O
4c, ^{1}H NMR (400 MHz, CDCl$_3$)
2.01
1.00
1.02
1.02
1.03
1.05
1.03
1.08
3.02
9.18
9.0 8.5 8.0 7.5 7.0 6.5 6.0 5.5 5.0 4.5 4.0 3.5 3.0 2.5 2.0 1.5 1.0 0.5 0.0
f1 (ppm)

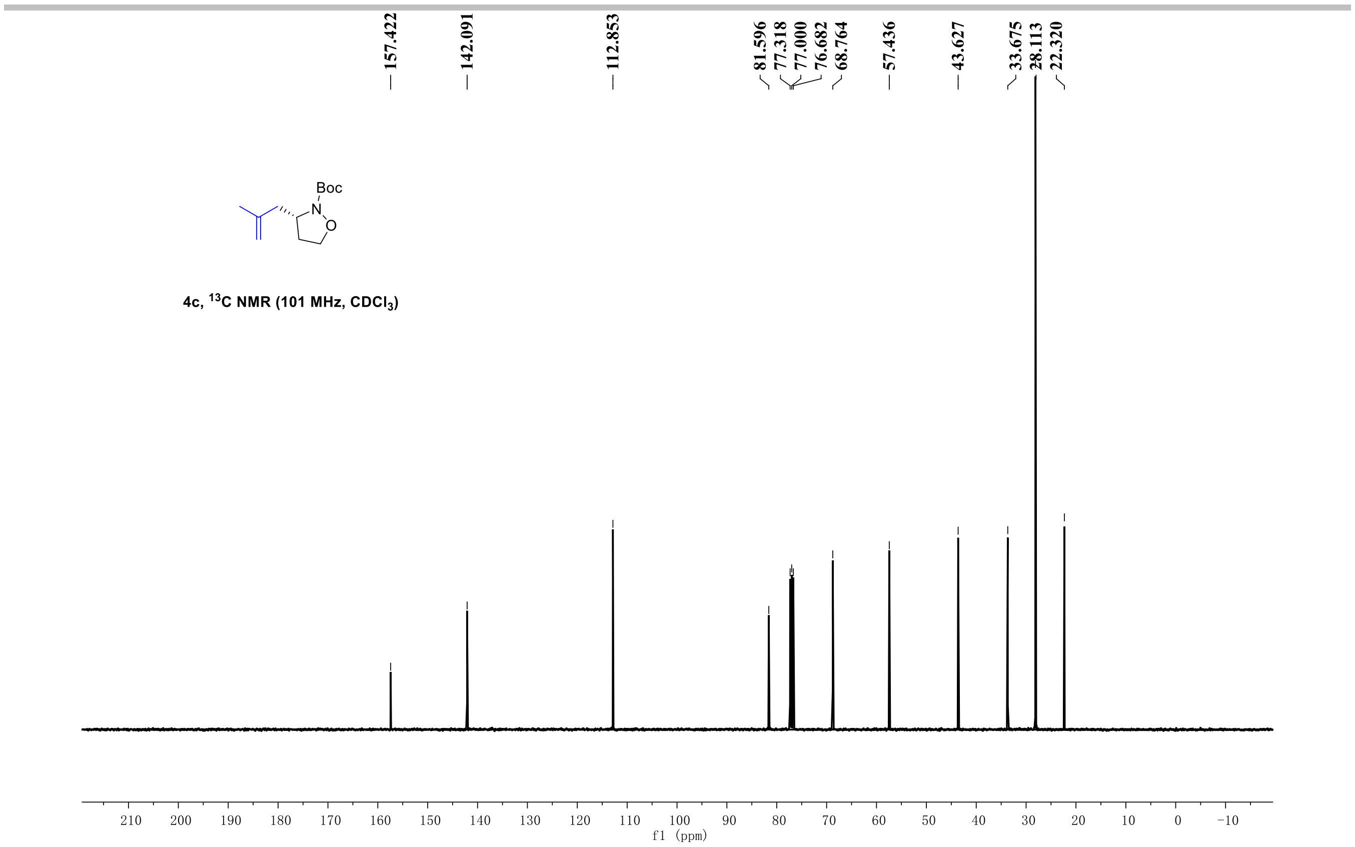
157.422
142.091
112.853
81.596
77.318
77.000
76.682
68.764
57.436
43.627
33.675
28.113
22.320
Boc
N
O
4c, 13C NMR (101 MHz, CDCl3)
f1 (ppm)

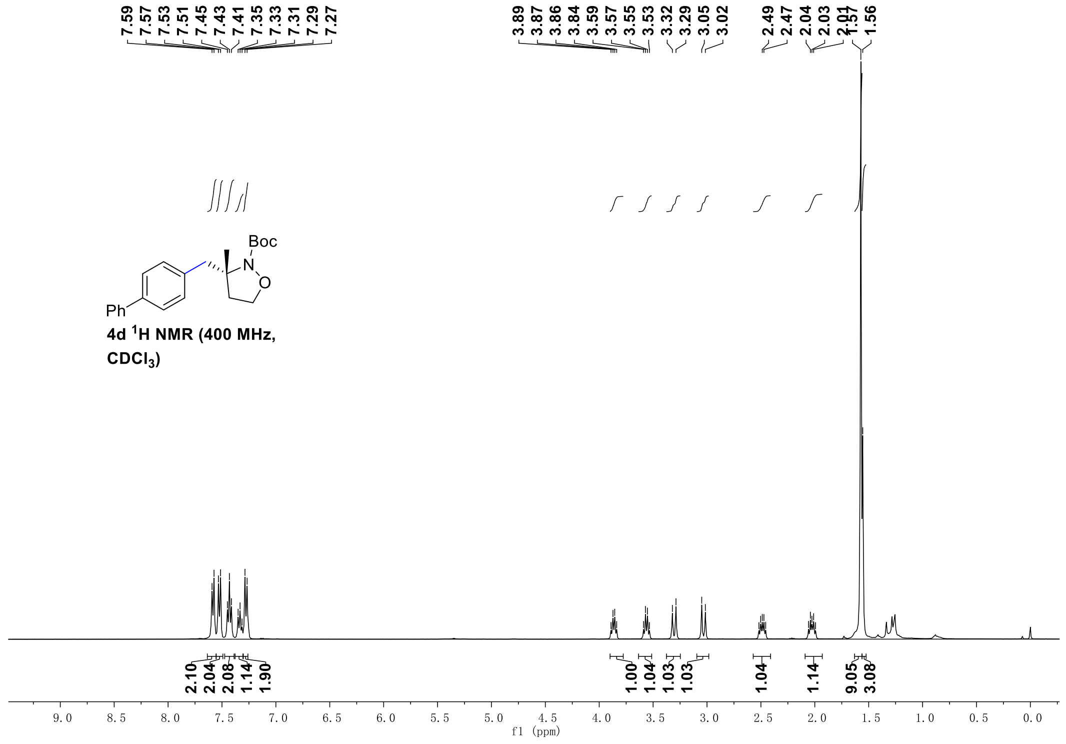
7.59
7.57
7.53
7.51
7.45
7.43
7.41
7.35
7.33
7.31
7.29
7.27
3.89
3.87
3.86
3.84
3.59
3.57
3.55
3.53
3.32
3.29
3.05
3.02
2.49
2.47
2.04
2.03
2.01
1.57
1.56
Boc
N
O
Ph
4d 1H NMR (400 MHz, CDCl3)
2.10
2.04
2.08
1.14
1.90
1.00
1.04
1.03
1.03
1.04
1.14
9.05
3.08
f1 (ppm)

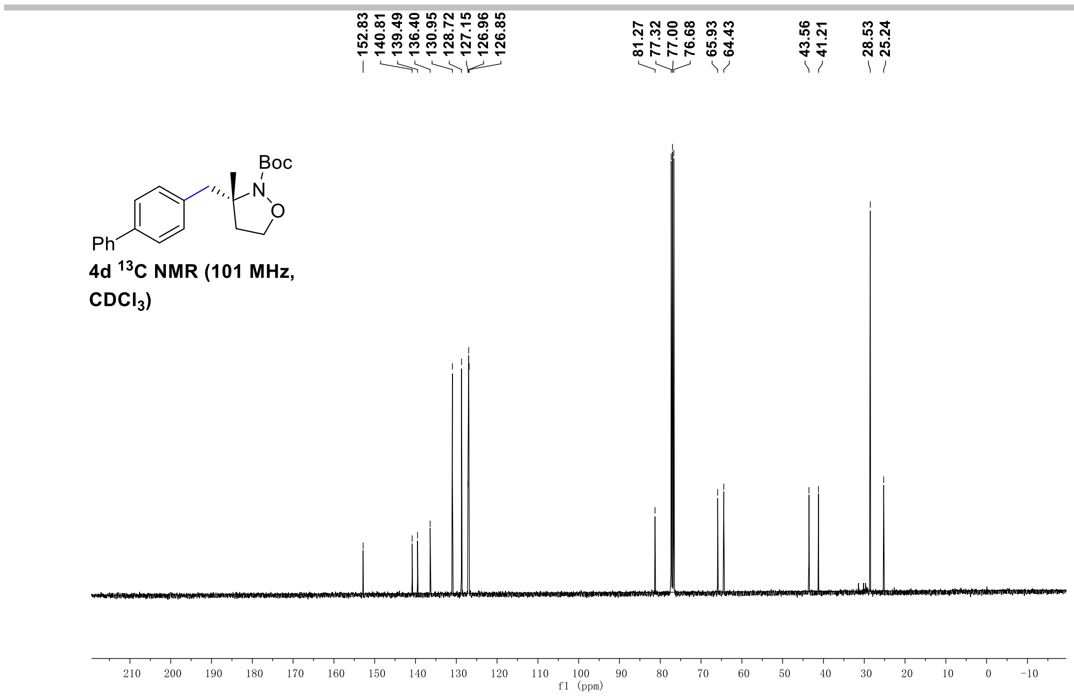

4d ^{13}C NMR (101 MHz, $CDCl_3$)

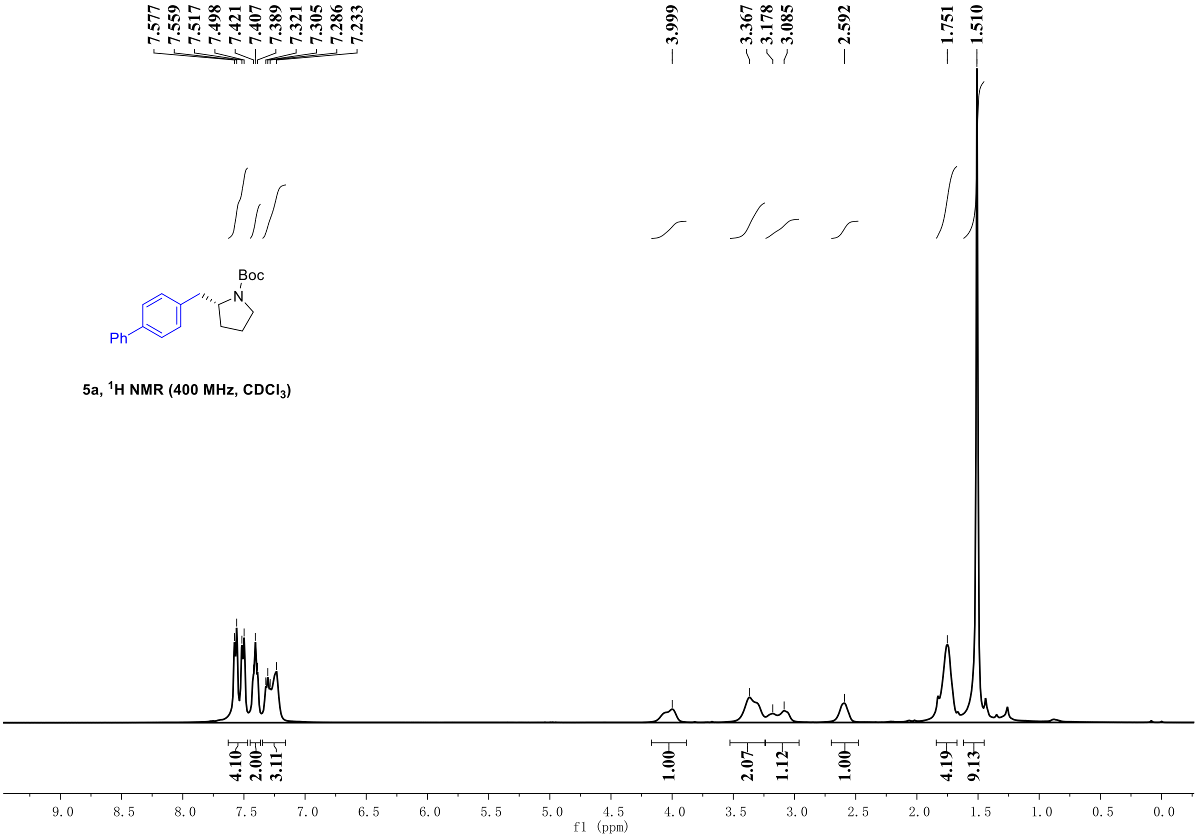

5a, ^{1}H NMR (400 MHz, $CDCl_3$)

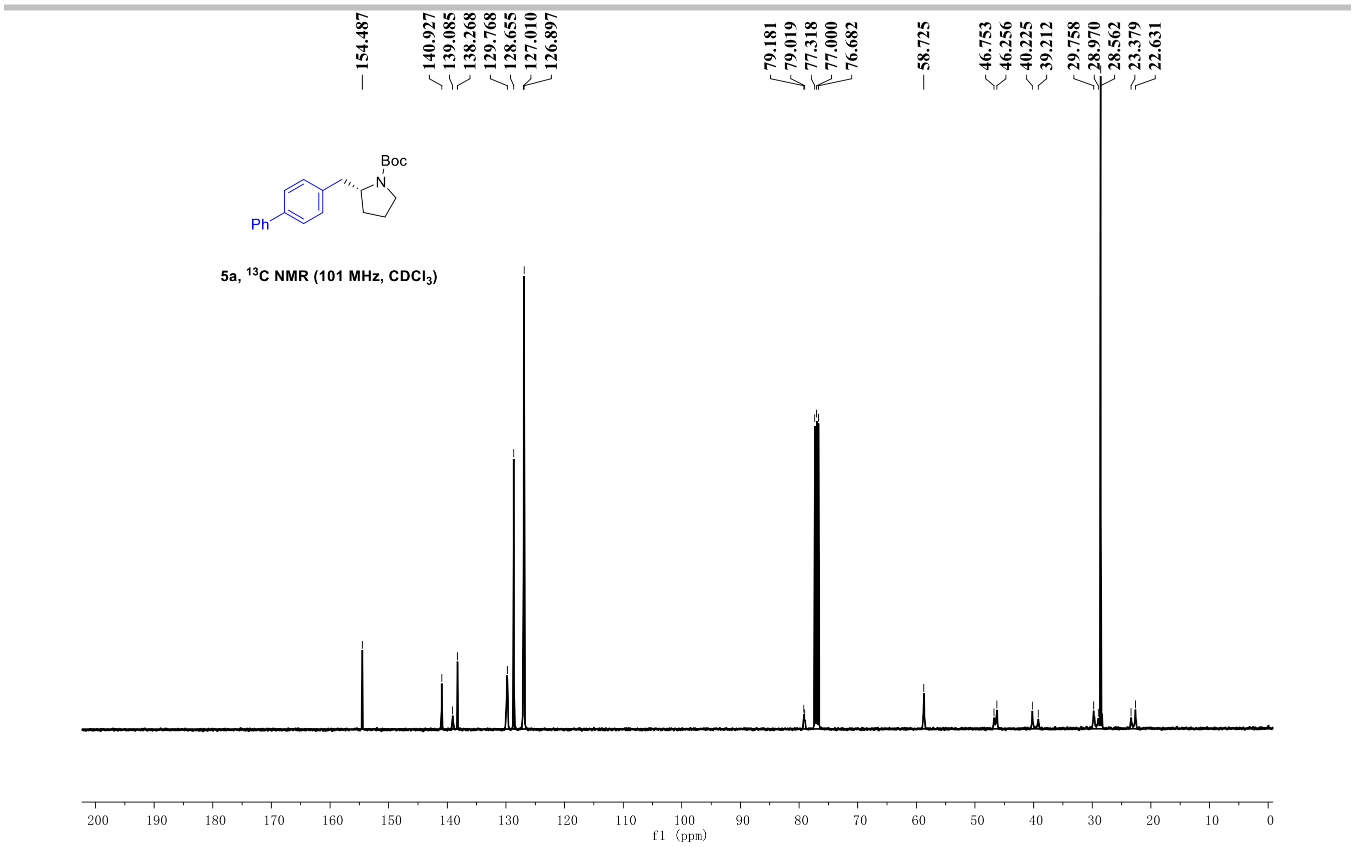
154.487
140.927
139.085
138.268
129.768
128.655
127.010
126.897
79.181
79.019
77.318
77.000
76.682
58.725
46.753
46.256
40.225
39.212
29.758
28.970
28.562
23.379
22.631
Boc
N
Ph
5a, ^{13}C NMR (101 MHz, $CDCl_3$)
200 190 180 170 160 150 140 130 120 110 100 90 80 70 60 50 40 30 20 10 0
f1 (ppm)

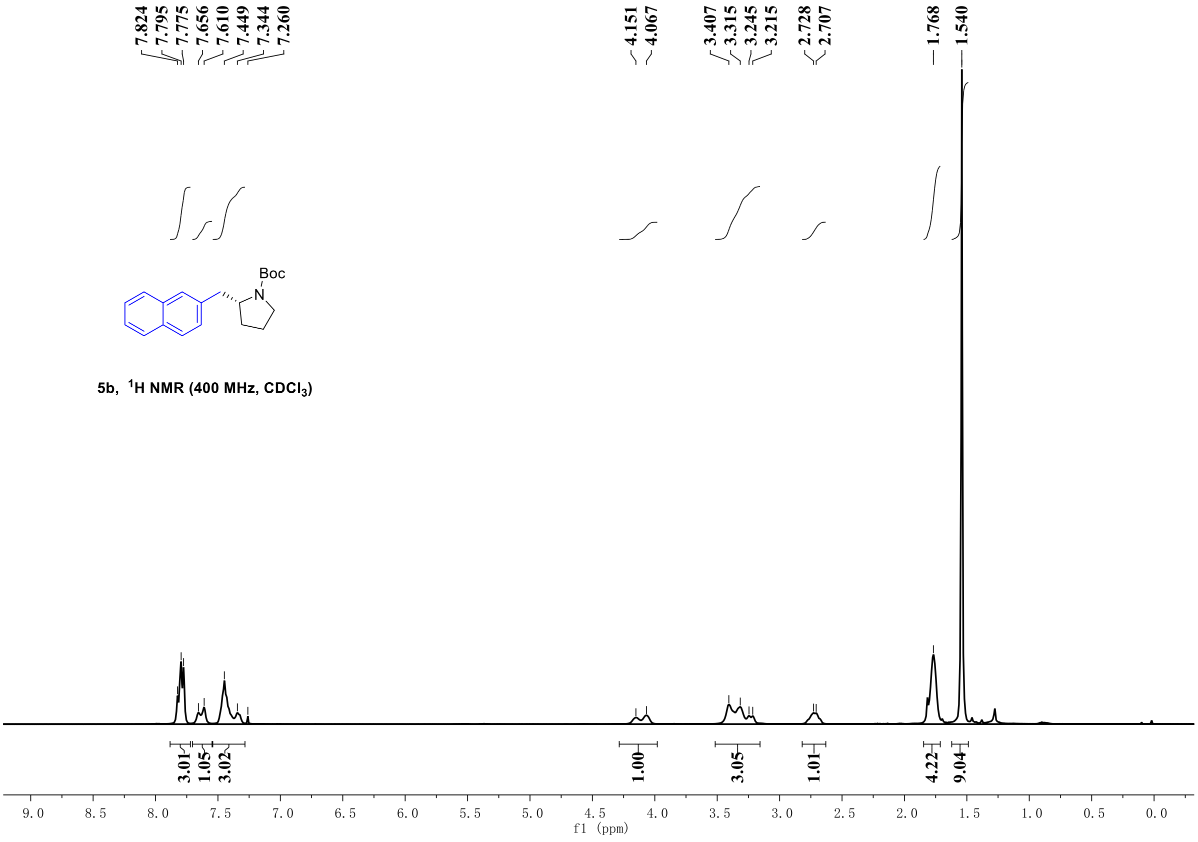
7.824
7.795
7.775
7.656
7.610
7.449
7.344
7.260
4.151
4.067
3.407
3.315
3.245
3.215
2.728
2.707
1.768
1.540
Boc
N
5b, ^{1}H NMR (400 MHz, $CDCl_3$)
3.01
1.05
3.02
1.00
3.05
1.01
4.22
9.04
9.0 8.5 8.0 7.5 7.0 6.5 6.0 5.5 5.0 4.5 4.0 3.5 3.0 2.5 2.0 1.5 1.0 0.5 0.0
f1 (ppm)

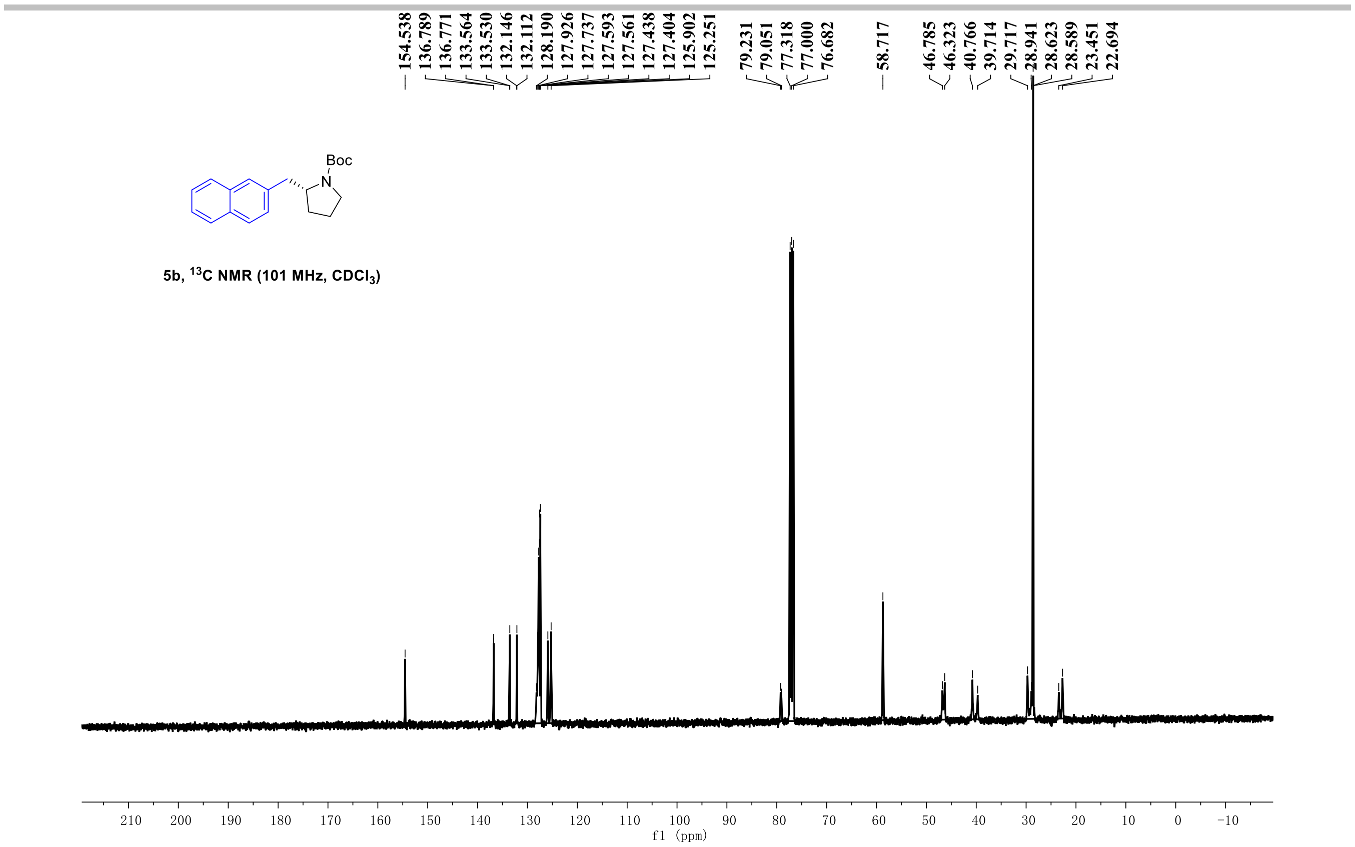
154.538
136.789
136.771
133.564
133.530
132.146
132.112
128.190
127.926
127.737
127.593
127.561
127.438
127.404
125.902
125.251
79.231
79.051
77.318
77.000
76.682
58.717
46.785
46.323
40.766
39.714
29.717
28.941
28.623
28.589
23.451
22.694
Boc
N
5b, 13C NMR (101 MHz, CDCl3)
f1 (ppm)

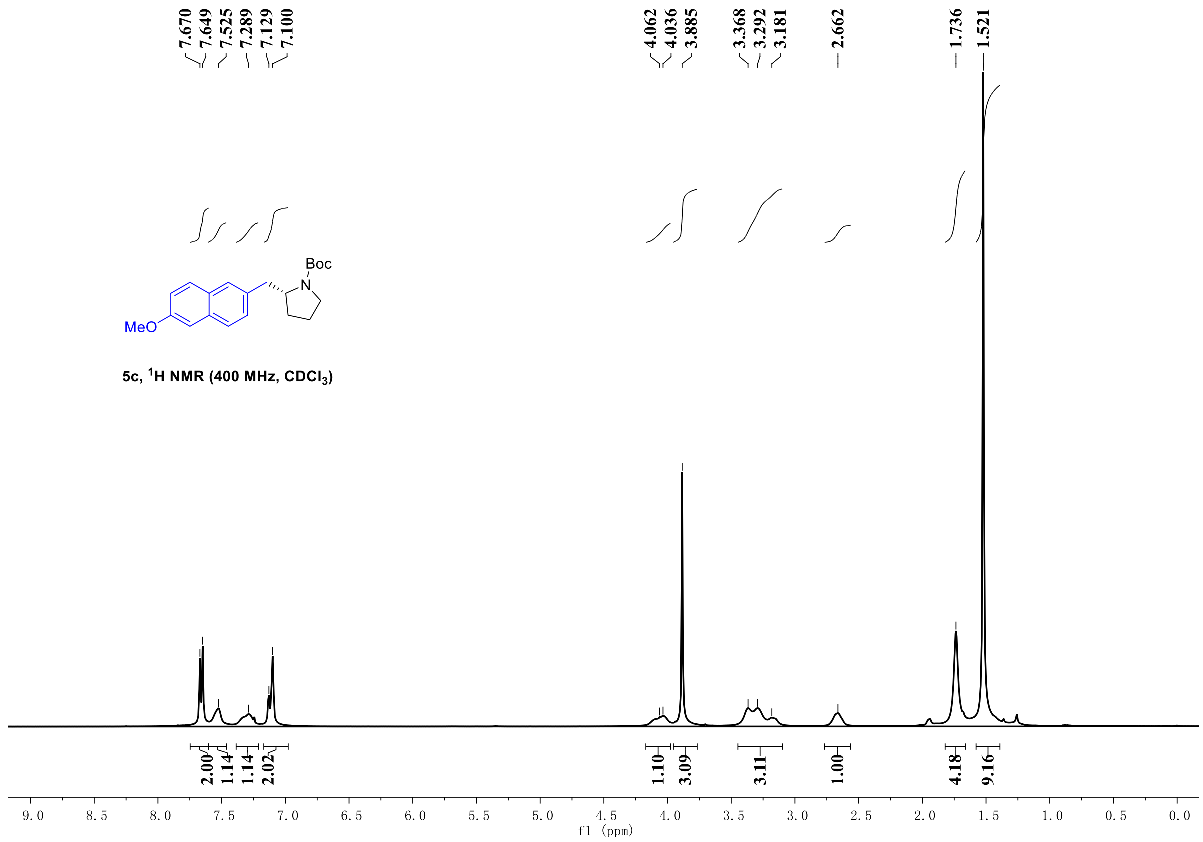
7.670
7.649
7.525
7.289
7.129
7.100
4.062
4.036
3.885
3.368
3.292
3.181
2.662
1.736
1.521
Boc
N
MeO
5c, 1H NMR (400 MHz, CDCl3)
2.00
1.14
1.14
2.02
1.10
3.09
3.11
1.00
4.18
9.16
f1 (ppm)

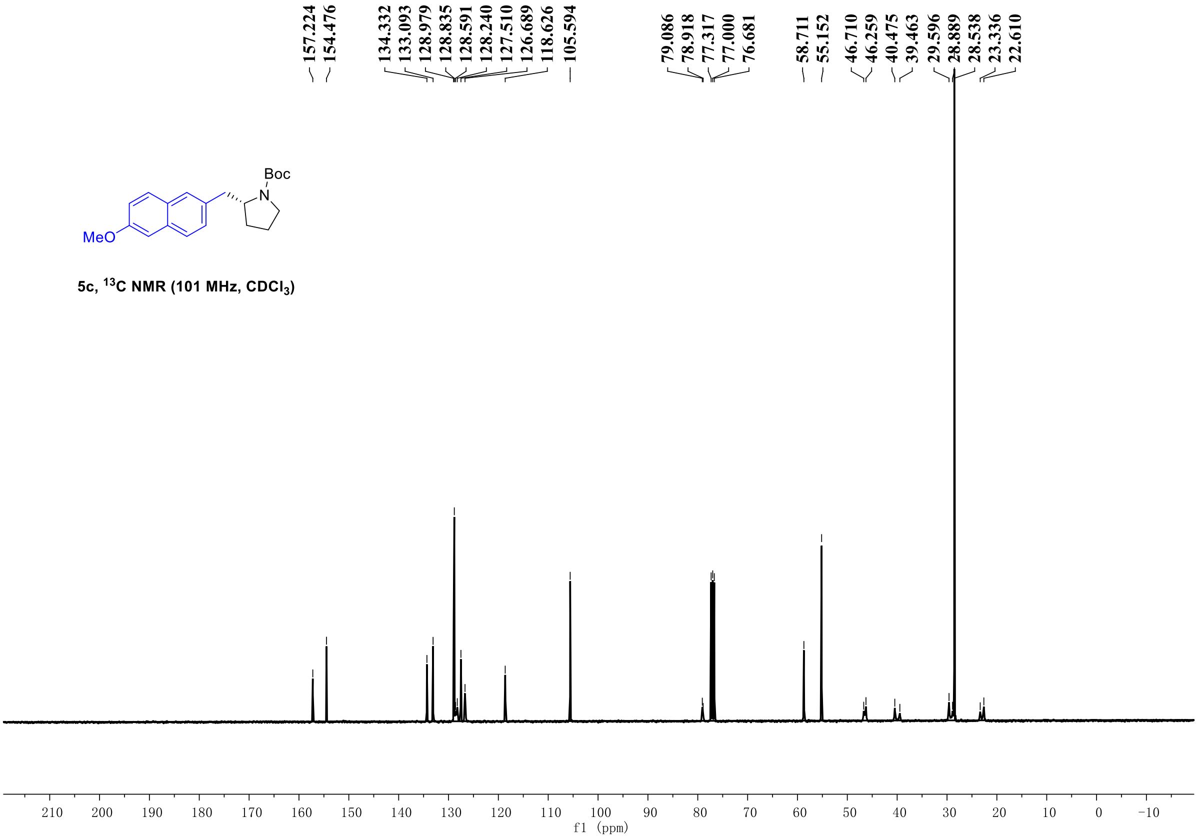
157.224
154.476
134.332
133.093
128.979
128.835
128.591
128.240
127.510
126.689
118.626
105.594
79.086
78.918
77.317
77.000
76.681
58.711
55.152
46.710
46.259
40.475
39.463
29.596
28.889
28.538
23.336
22.610
Boc
N
MeO
5c, ^{13}C NMR (101 MHz, CDCl$_3$)
210 200 190 180 170 160 150 140 130 120 110 100 90 80 70 60 50 40 30 20 10 0 -10
f1 (ppm)

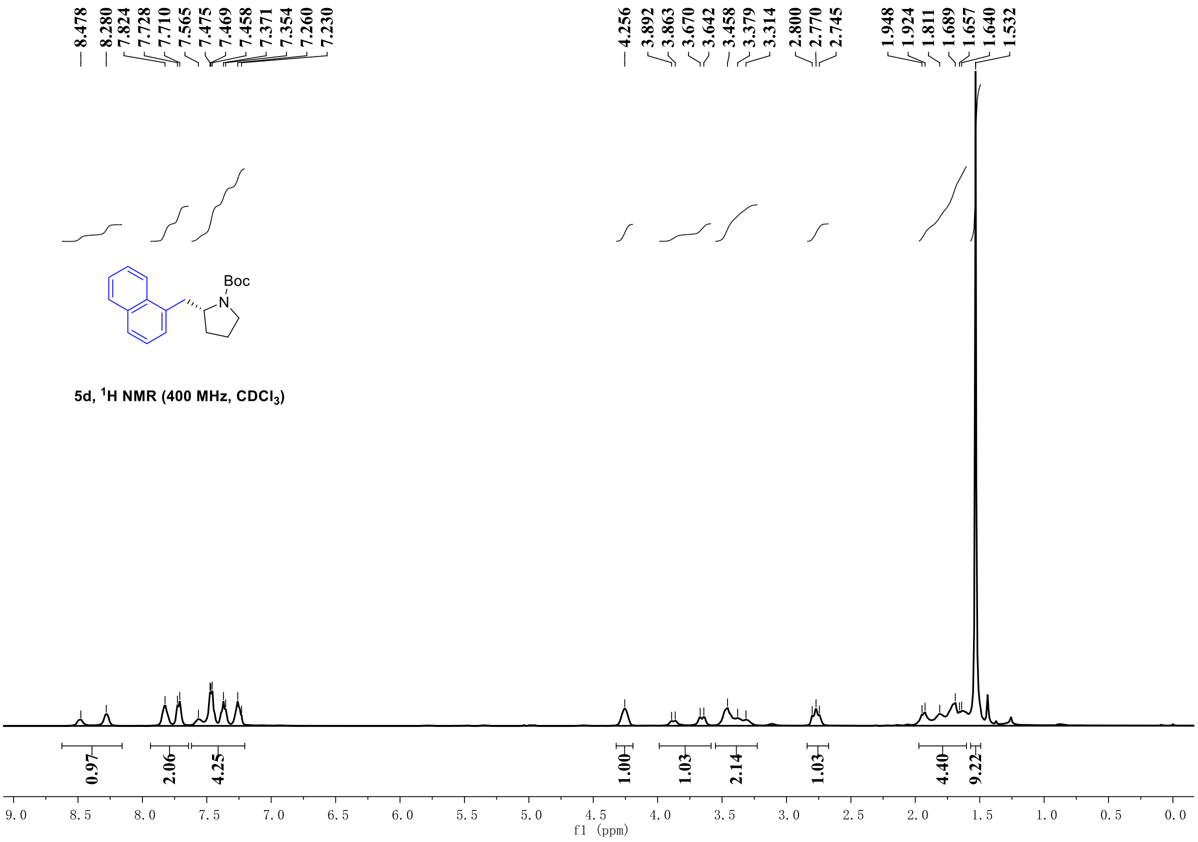
8.478
8.280
7.824
7.728
7.710
7.565
7.475
7.469
7.458
7.371
7.354
7.260
7.230
4.256
3.892
3.863
3.670
3.642
3.458
3.379
3.314
2.800
2.770
2.745
1.948
1.924
1.811
1.689
1.657
1.640
1.532
Boc
N
5d, ^{1}H NMR (400 MHz, CDCl$_3$)
0.97
2.06
4.25
1.00
1.03
2.14
1.03
4.40
9.22
9.0 8.5 8.0 7.5 7.0 6.5 6.0 5.5 5.0 4.5 4.0 3.5 3.0 2.5 2.0 1.5 1.0 0.5 0.0
f1 (ppm)

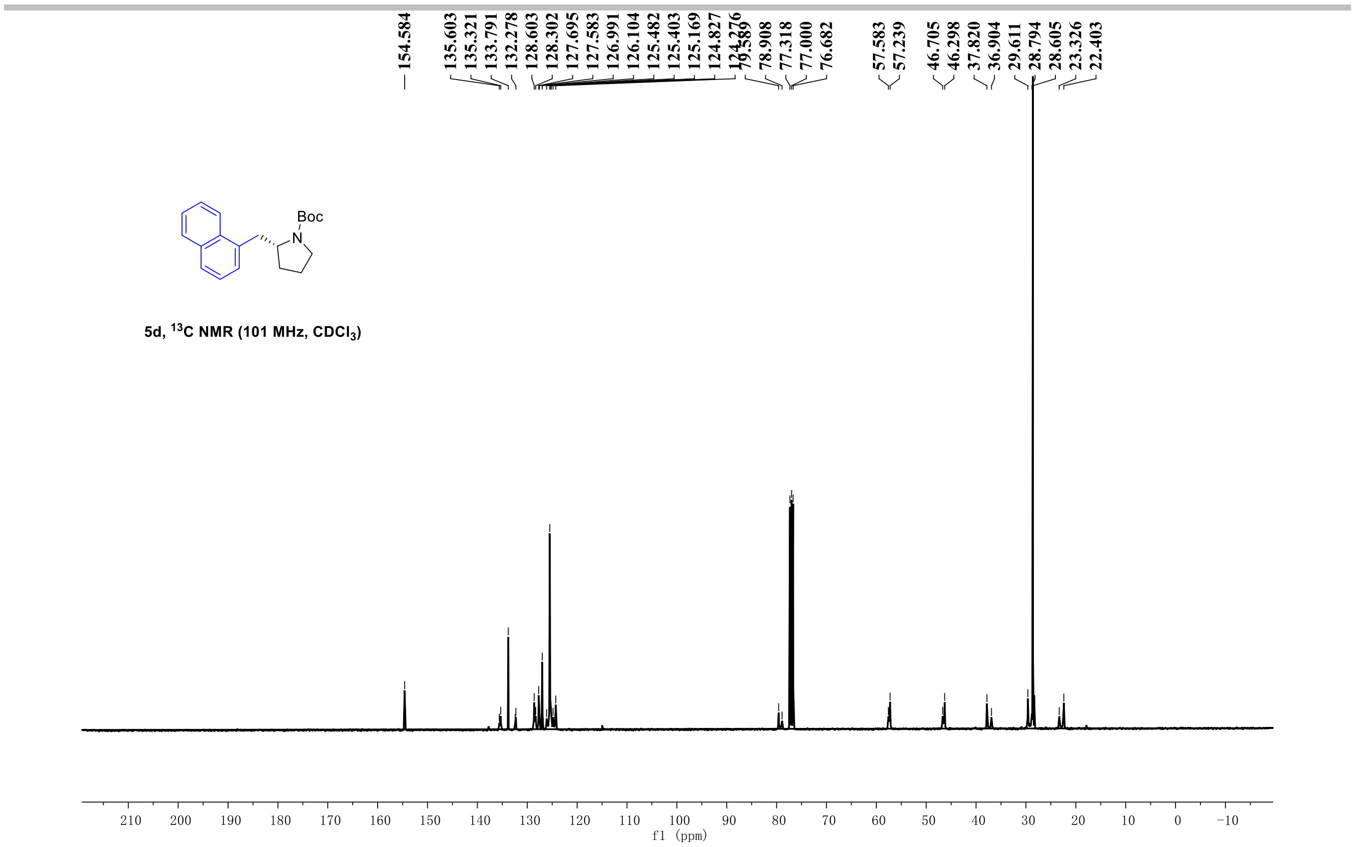

5d, ^{13}C NMR (101 MHz, $CDCl_3$)

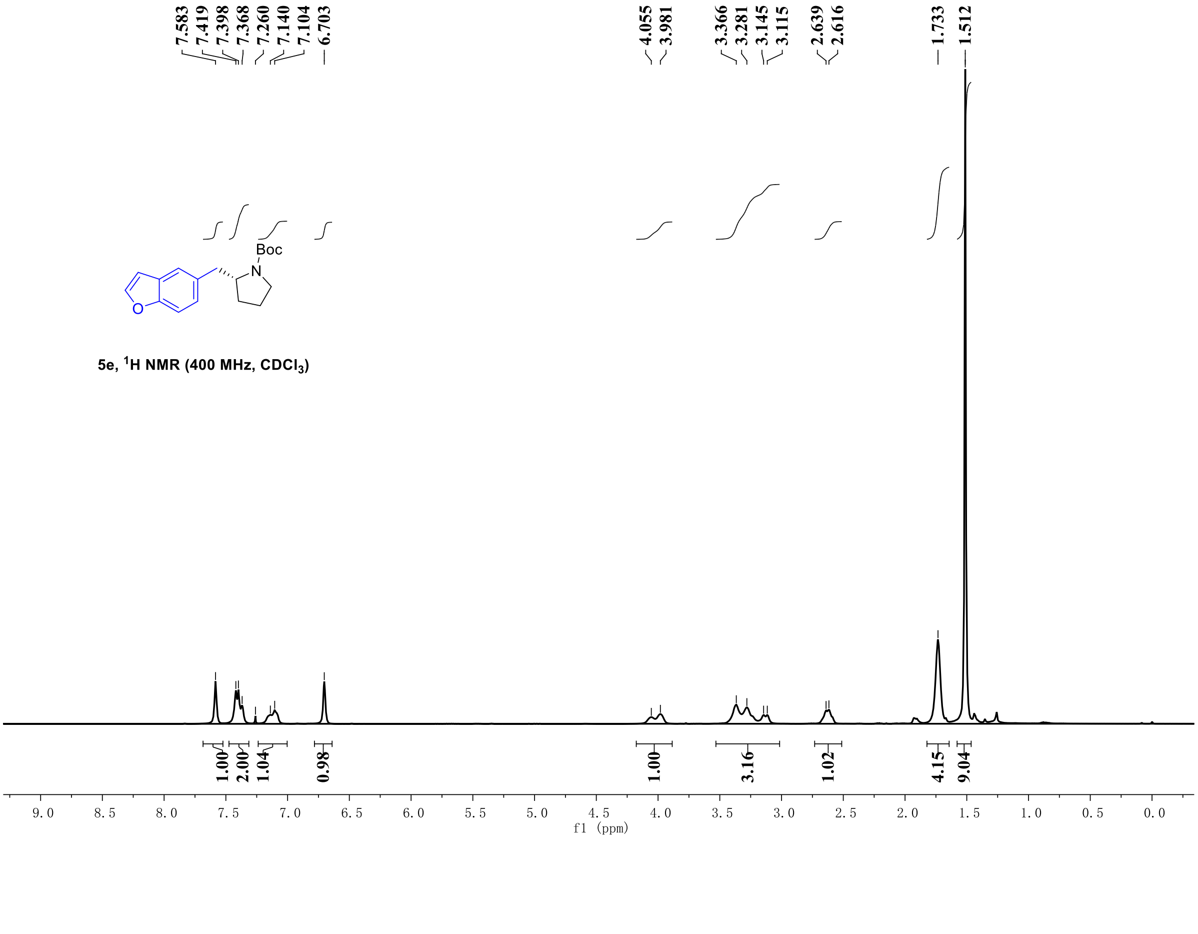

5e, ^{1}H NMR (400 MHz, $CDCl_3$)

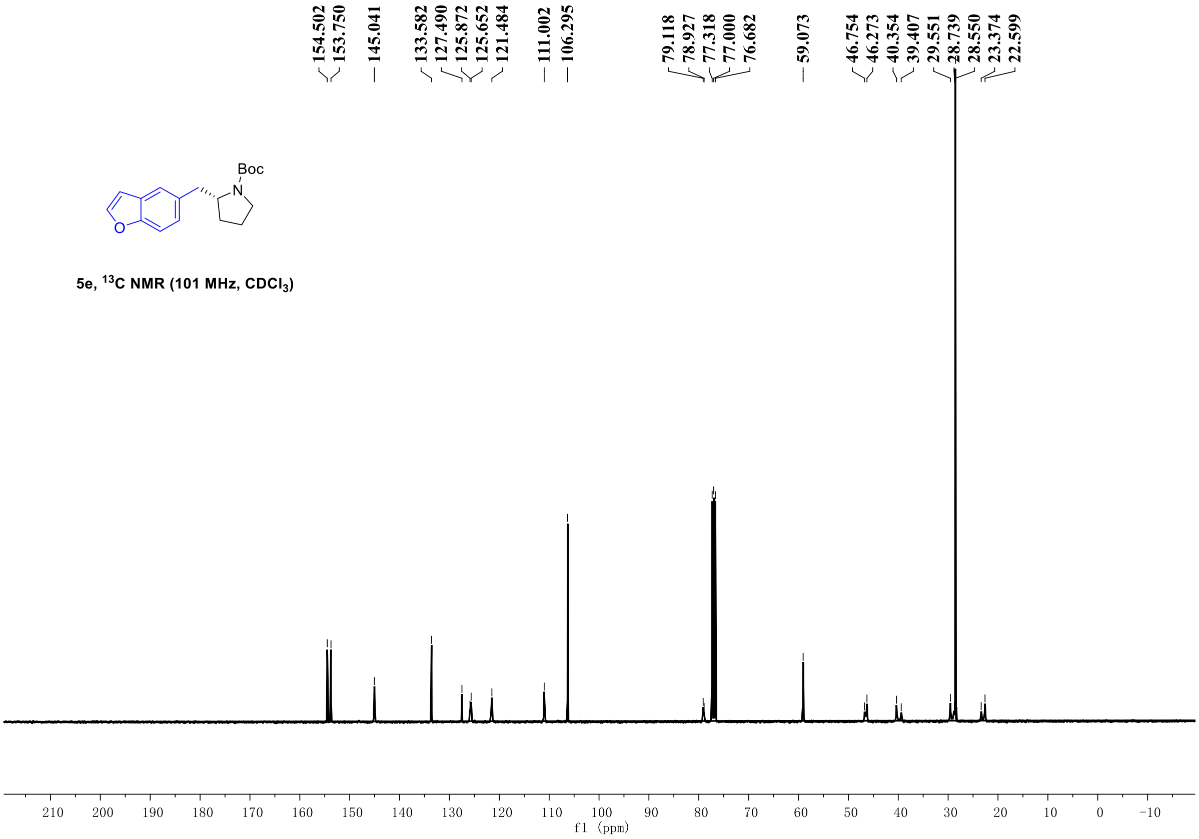

5e, ^{13}C NMR (101 MHz, CDCl$_3$)

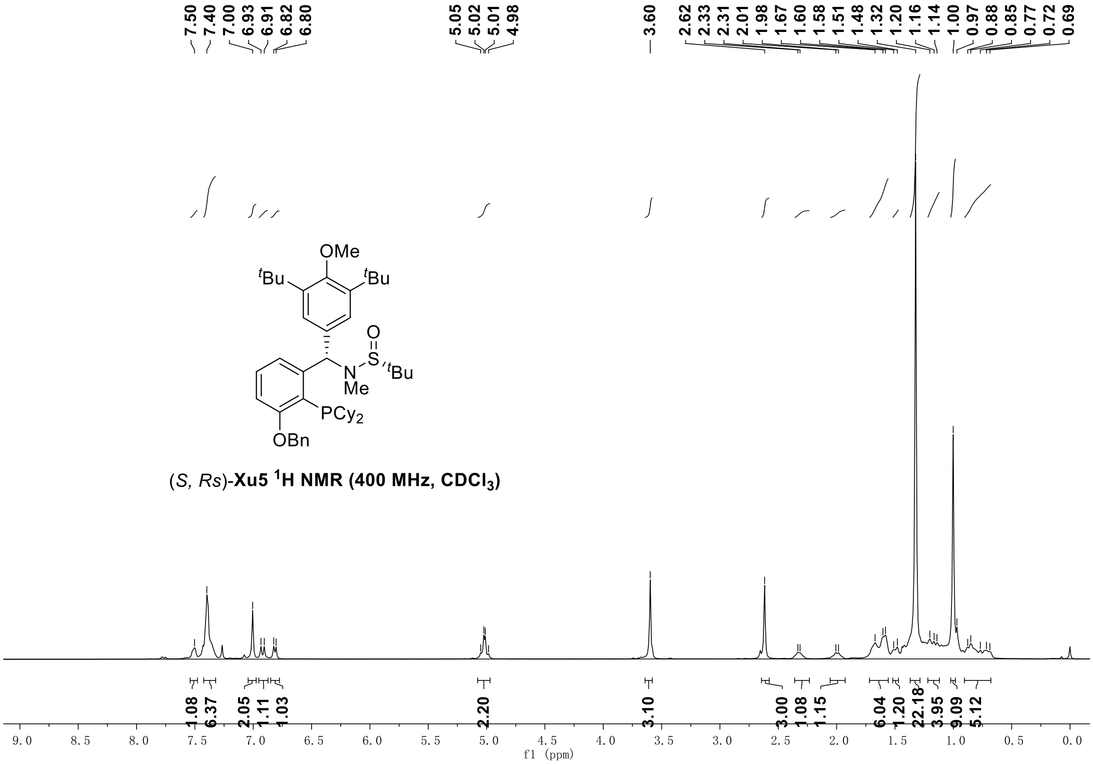

(S, Rs)-**Xu5 ^{1}H NMR (400 MHz, CDCl$_3$)**

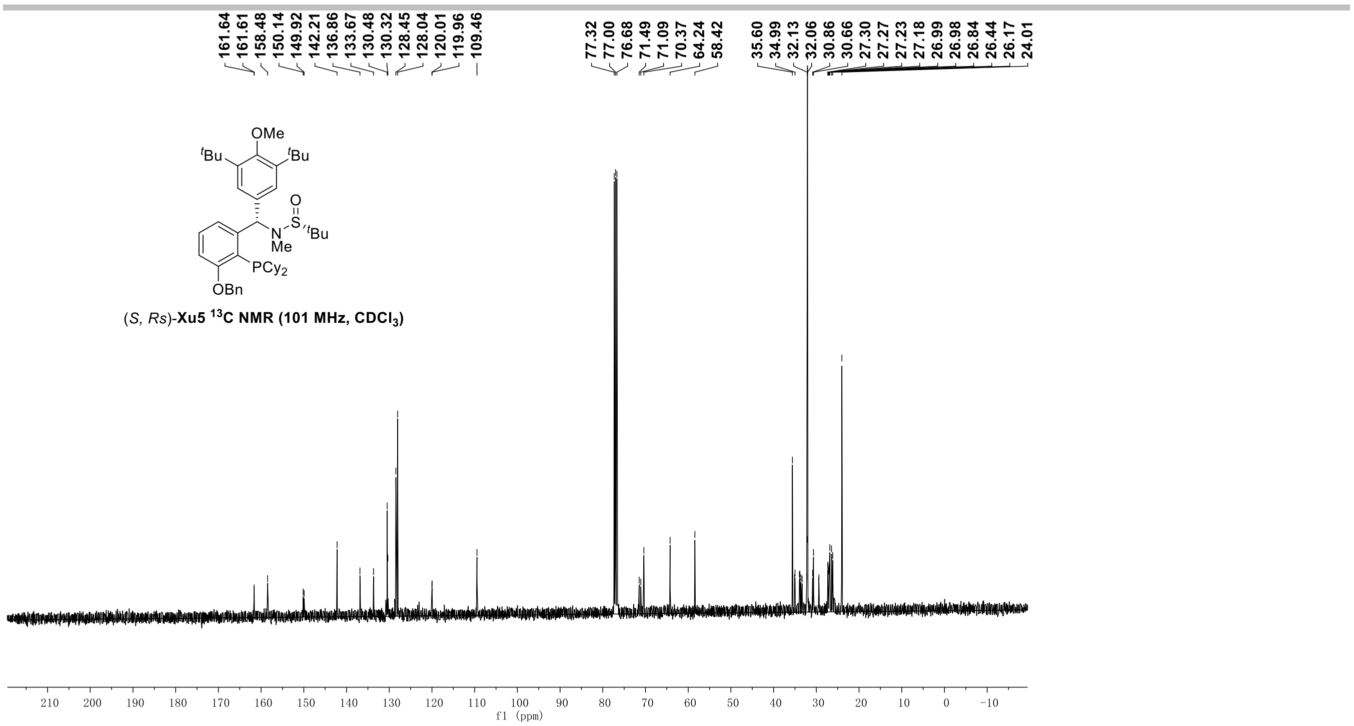
161.64
161.61
158.48
150.14
149.92
142.21
136.86
133.67
130.48
130.32
128.45
128.04
120.01
119.96
109.46
77.32
77.00
76.68
71.49
71.09
70.37
64.24
58.42
35.60
34.99
32.13
32.06
30.86
30.66
27.30
27.27
27.23
27.18
26.99
26.98
26.84
26.44
26.17
24.01
OMe
tBu
tBu
O
S
tBu
N
Me
PCy2
OBn
(S, Rs)-Xu5 13C NMR (101 MHz, CDCl3)
f1 (ppm)

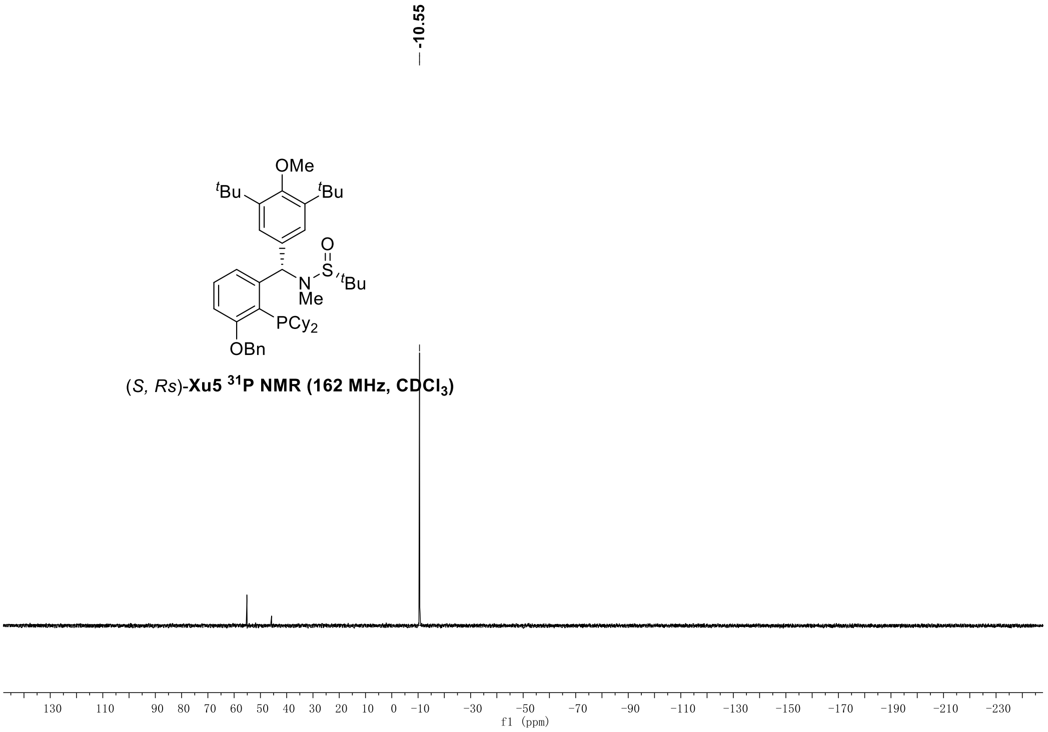
-10.55
OMe
tBu
tBu
O
S
tBu
N
Me
PCy2
OBn
(S, Rs)-Xu5 31P NMR (162 MHz, CDCl3)
f1 (ppm)

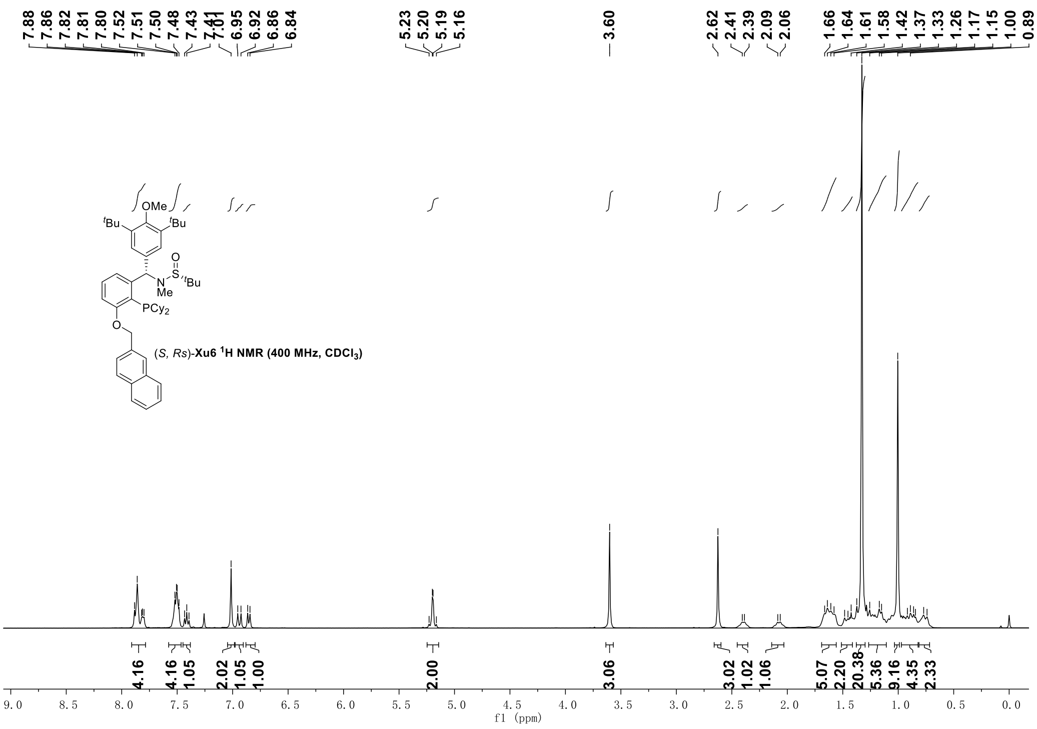
(S, Rs)-Xu6 1H NMR (400 MHz, CDCl3)
OMe
tBu
N
Me
S
O
PCy2
O
f1 (ppm)

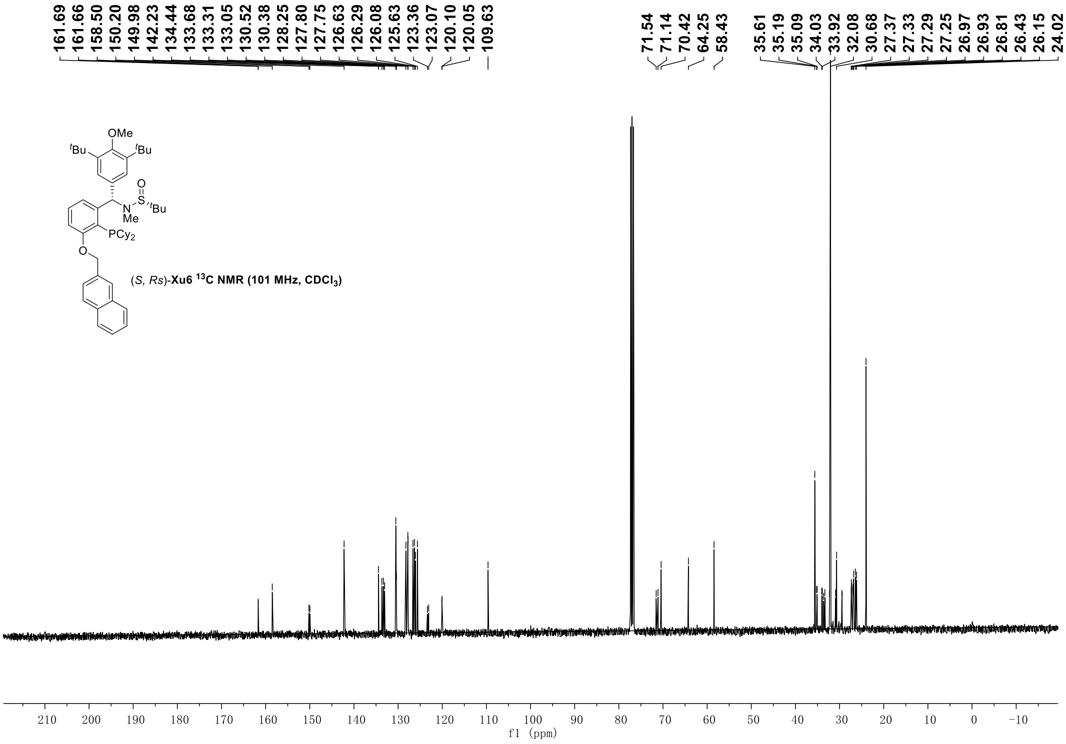
(S, Rs)-Xu6 13C NMR (101 MHz, CDCl3)
OMe
tBu
N
Me
S
O
PCy2
O
f1 (ppm)

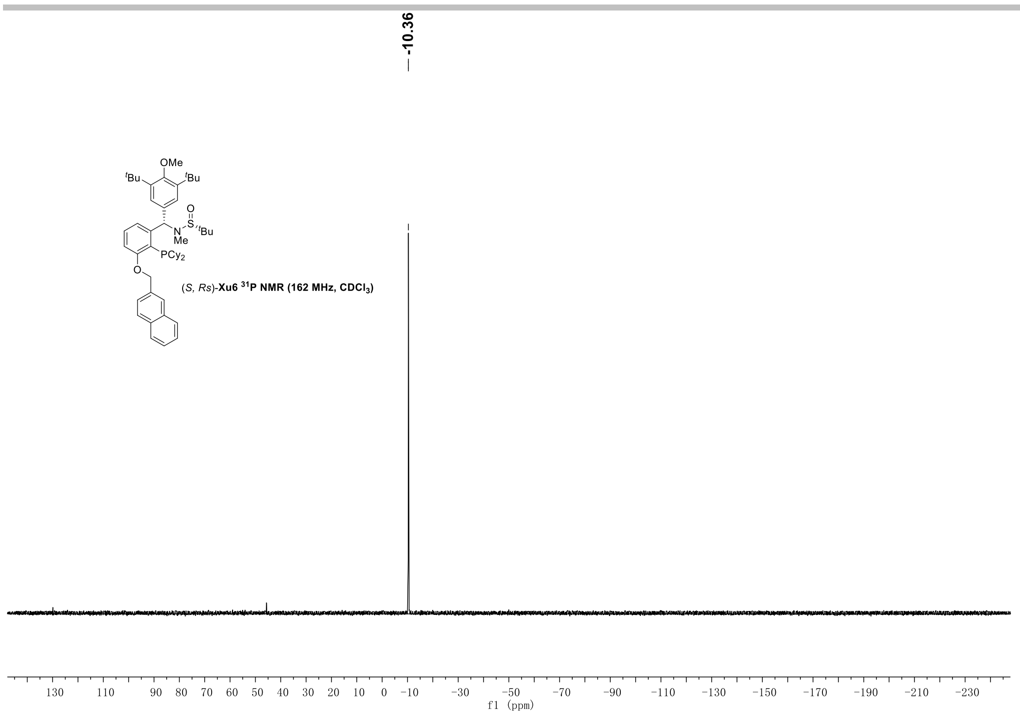
-10.36
OMe
tBu
tBu
O
S
tBu
N
Me
PCy2
O
(S, Rs)-Xu6 31P NMR (162 MHz, CDCl3)
130 110 90 80 70 60 50 40 30 20 10 0 -10 -30 -50 -70 -90 -110 -130 -150 -170 -190 -210 -230
f1 (ppm)

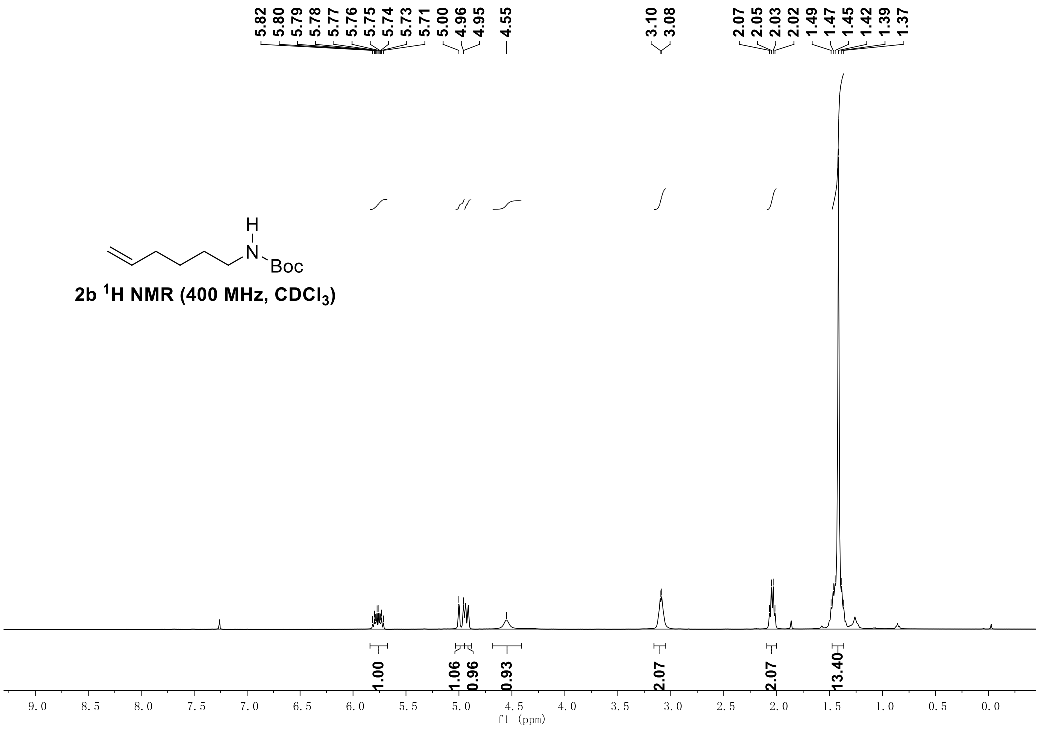
5.82 5.80 5.79 5.78 5.77 5.76 5.75 5.74 5.73 5.71 5.00 4.96 4.95 4.55
3.10 3.08
2.07 2.05 2.03 2.02 1.49 1.47 1.45 1.42 1.39 1.37
H
N
Boc
2b 1H NMR (400 MHz, CDCl3)
1.00 1.06 0.96 0.93 2.07 2.07 13.40
9.0 8.5 8.0 7.5 7.0 6.5 6.0 5.5 5.0 4.5 4.0 3.5 3.0 2.5 2.0 1.5 1.0 0.5 0.0
f1 (ppm)

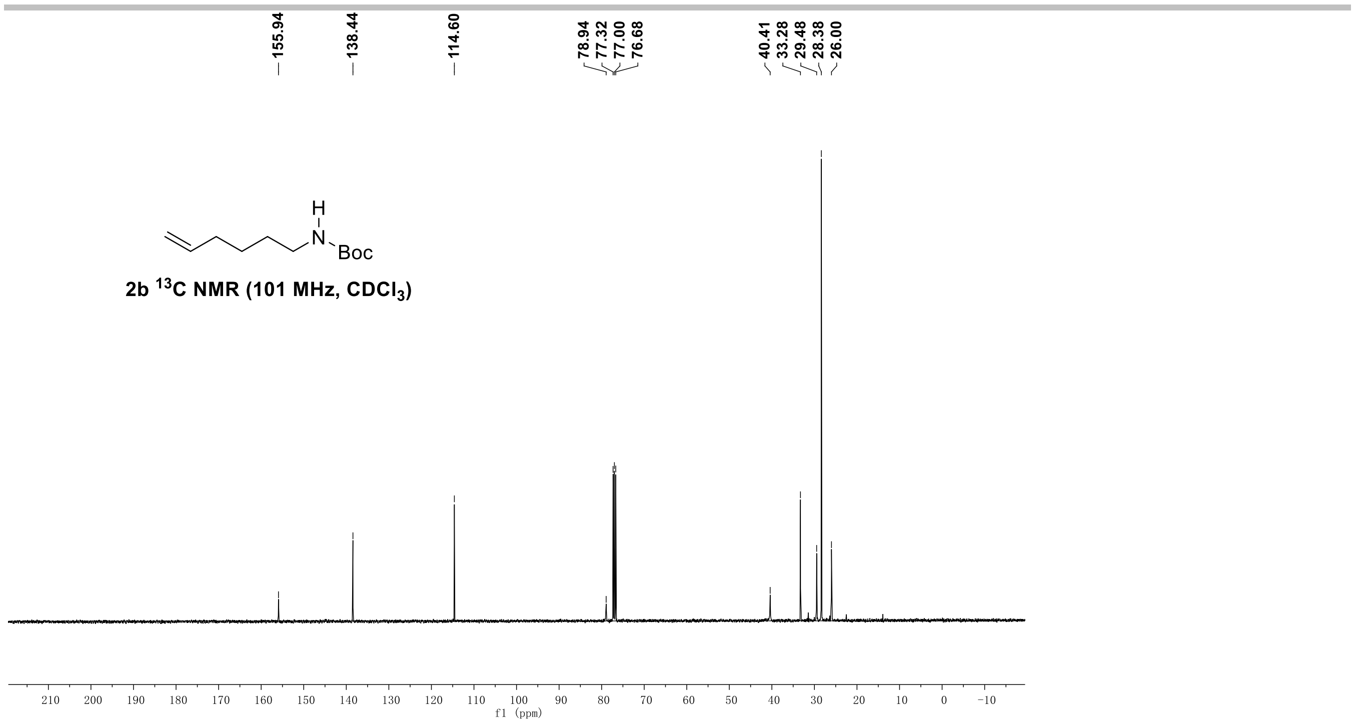
155.94
138.44
114.60
78.94
77.32
77.00
76.68
40.41
33.28
29.48
28.38
26.00
H
N
Boc
2b 13C NMR (101 MHz, CDCl3)
210 200 190 180 170 160 150 140 130 120 110 100 90 80 70 60 50 40 30 20 10 0 -10
f1 (ppm)

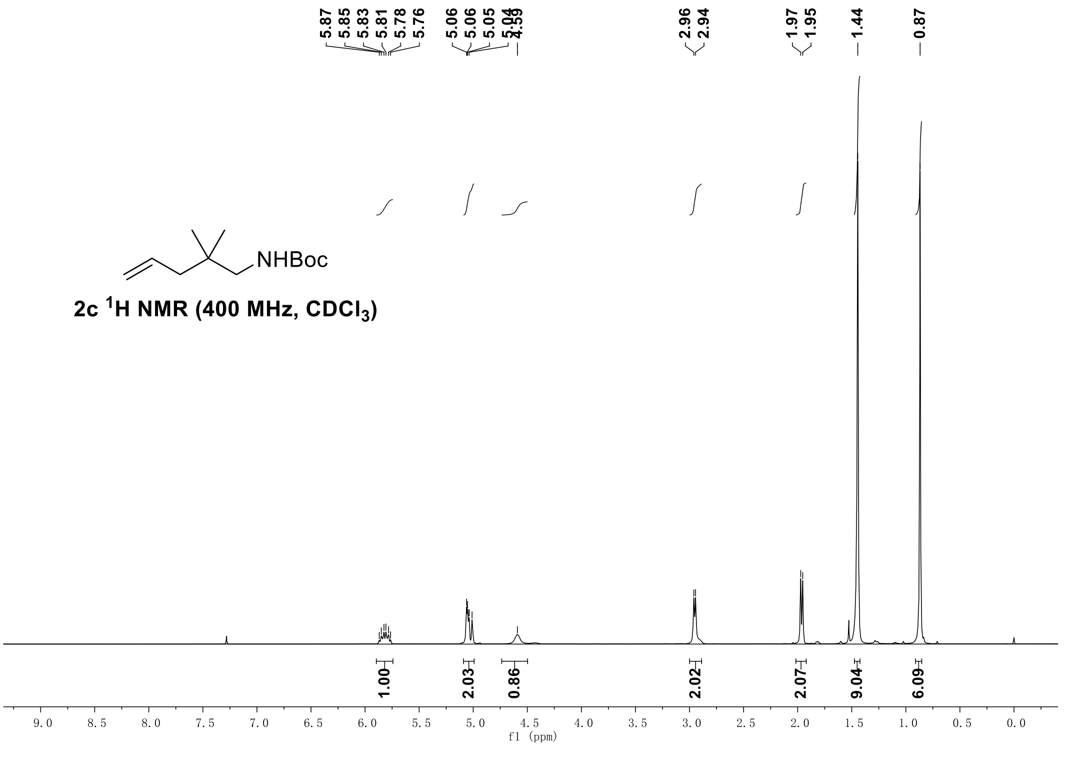
5.87
5.85
5.83
5.81
5.78
5.76
5.06
5.06
5.05
5.04
4.59
2.96
2.94
1.97
1.95
1.44
0.87
NHBoc
2c 1H NMR (400 MHz, CDCl3)
1.00
2.03
0.86
2.02
2.07
9.04
6.09
9.0 8.5 8.0 7.5 7.0 6.5 6.0 5.5 5.0 4.5 4.0 3.5 3.0 2.5 2.0 1.5 1.0 0.5 0.0
f1 (ppm)

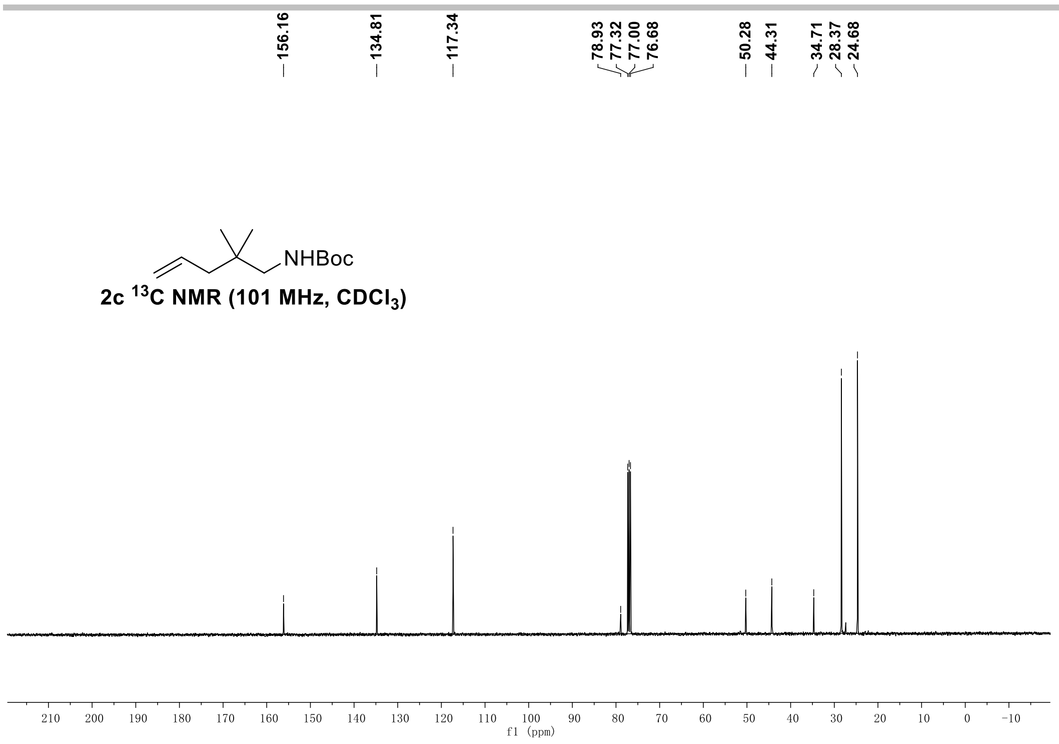

2c ^{13}C NMR (101 MHz, CDCl$_3$)

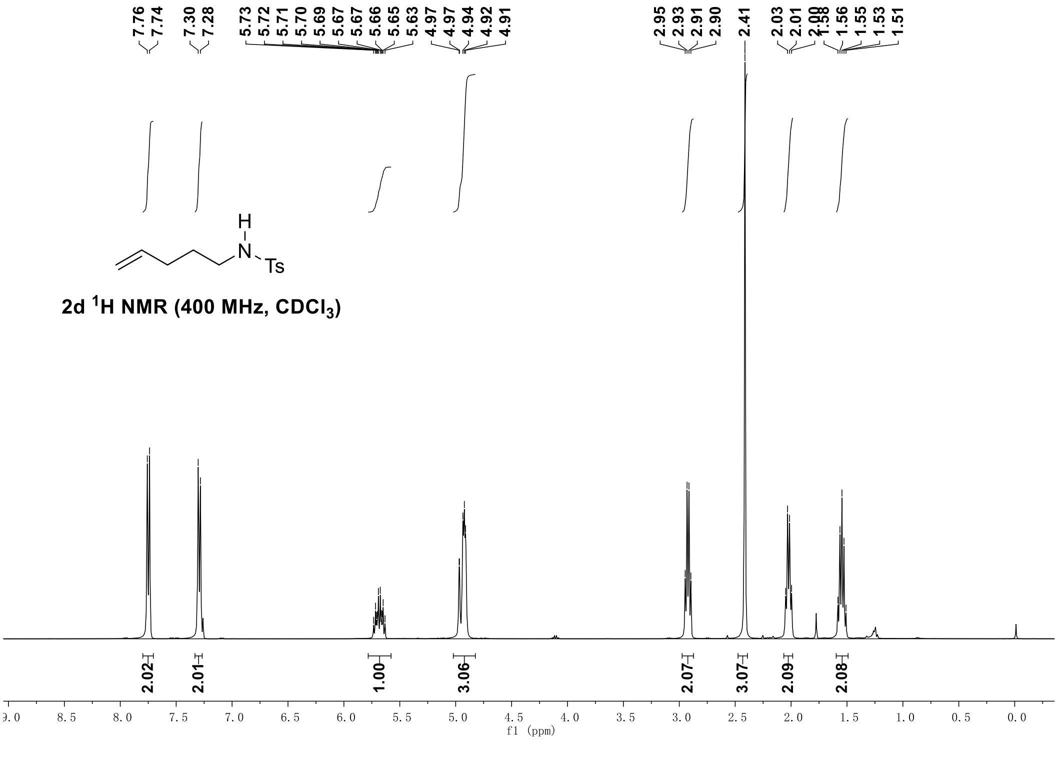

2d ^{1}H NMR (400 MHz, CDCl$_3$)

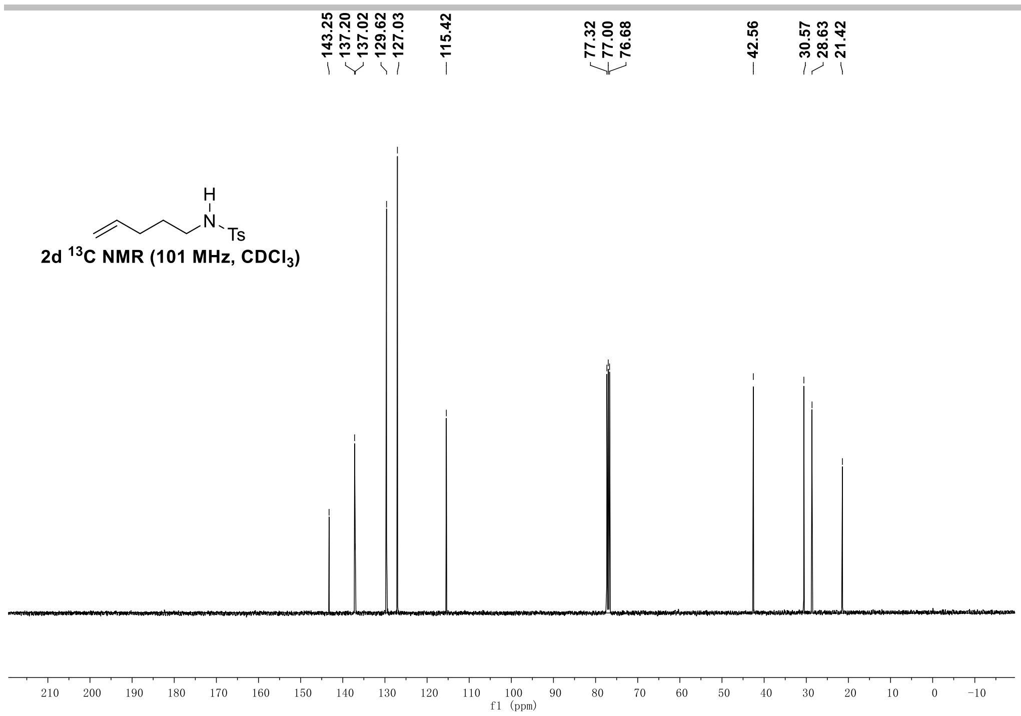
143.25
137.20
137.02
129.62
127.03
115.42
77.32
77.00
76.68
42.56
30.57
28.63
21.42
H
N
Ts
2d 13C NMR (101 MHz, CDCl3)
210 200 190 180 170 160 150 140 130 120 110 100 90 80 70 60 50 40 30 20 10 0 -10
f1 (ppm)

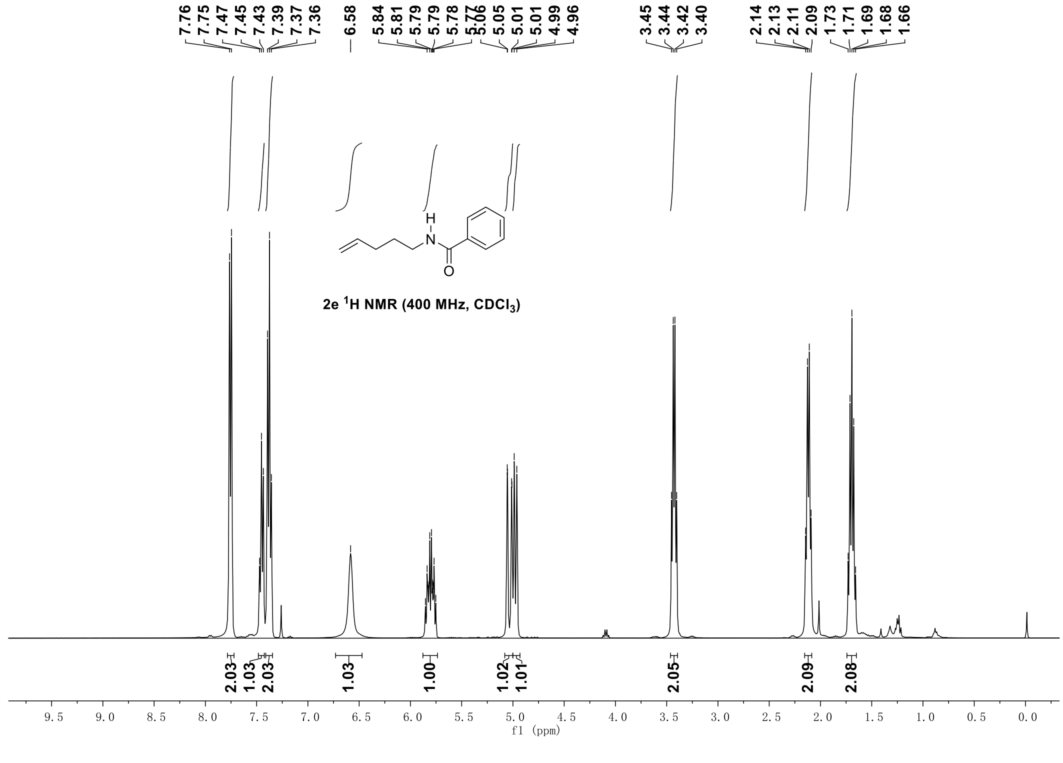
7.76
7.75
7.47
7.45
7.43
7.39
7.37
7.36
6.58
5.84
5.81
5.79
5.79
5.78
5.77
5.06
5.05
5.01
5.01
4.99
4.96
3.45
3.44
3.42
3.40
2.14
2.13
2.11
2.09
1.73
1.71
1.69
1.68
1.66
H
N
O
2e 1H NMR (400 MHz, CDCl3)
2.03
1.03
2.03
1.03
1.00
1.02
1.01
2.05
2.09
2.08
9.5 9.0 8.5 8.0 7.5 7.0 6.5 6.0 5.5 5.0 4.5 4.0 3.5 3.0 2.5 2.0 1.5 1.0 0.5 0.0
f1 (ppm)

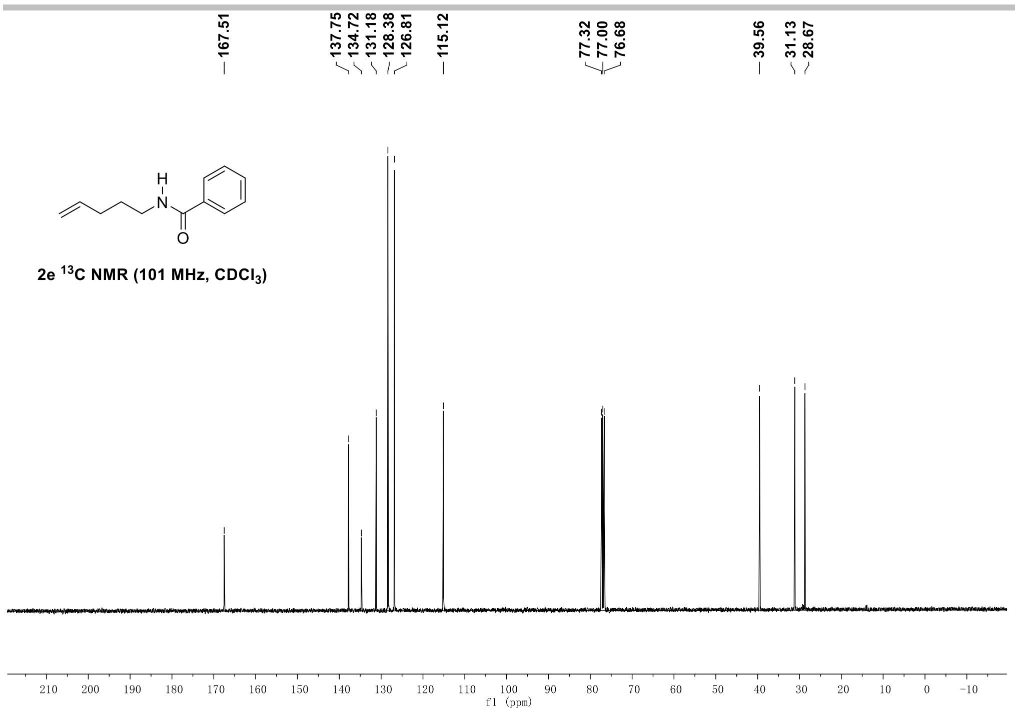
167.51
137.75
134.72
131.18
128.38
126.81
115.12
77.32
77.00
76.68
39.56
31.13
28.67
H
N
O
2e 13C NMR (101 MHz, CDCl3)
210 200 190 180 170 160 150 140 130 120 110 100 90 80 70 60 50 40 30 20 10 0 -10
f1 (ppm)

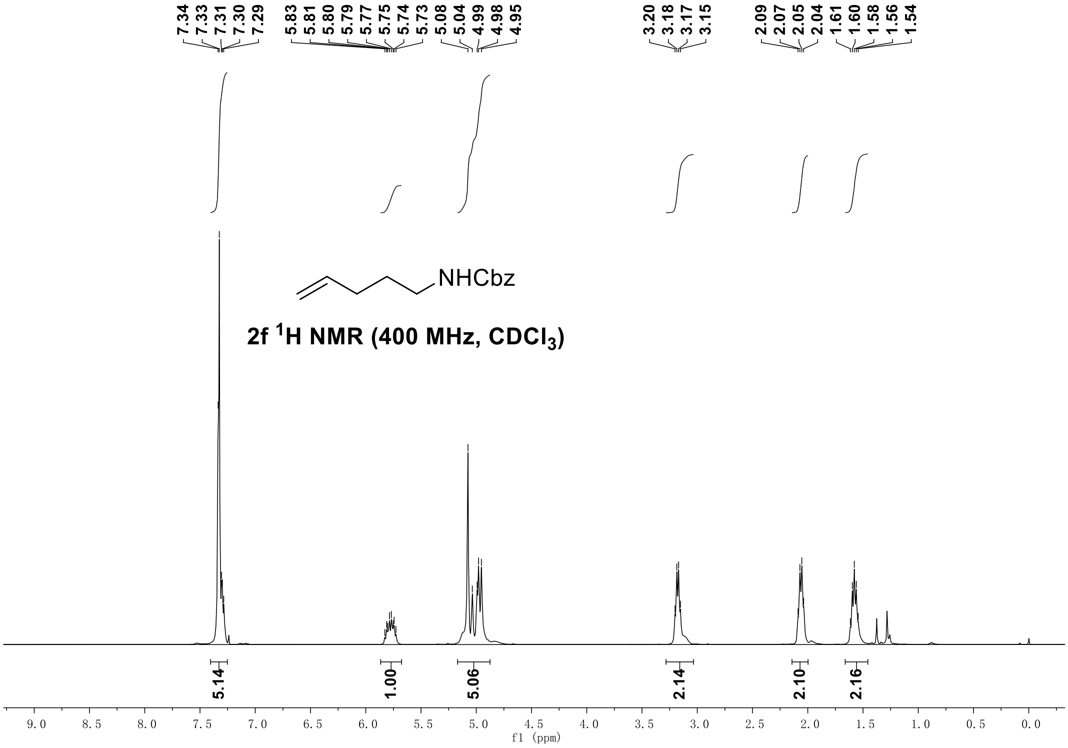
7.34
7.33
7.31
7.30
7.29
5.83
5.81
5.80
5.79
5.77
5.75
5.74
5.73
5.08
5.04
4.99
4.98
4.95
3.20
3.18
3.17
3.15
2.09
2.07
2.05
2.04
1.61
1.60
1.58
1.56
1.54
NHCbz
2f 1H NMR (400 MHz, CDCl3)
5.14
1.00
5.06
2.14
2.10
2.16
9.0 8.5 8.0 7.5 7.0 6.5 6.0 5.5 5.0 4.5 4.0 3.5 3.0 2.5 2.0 1.5 1.0 0.5 0.0
f1 (ppm)

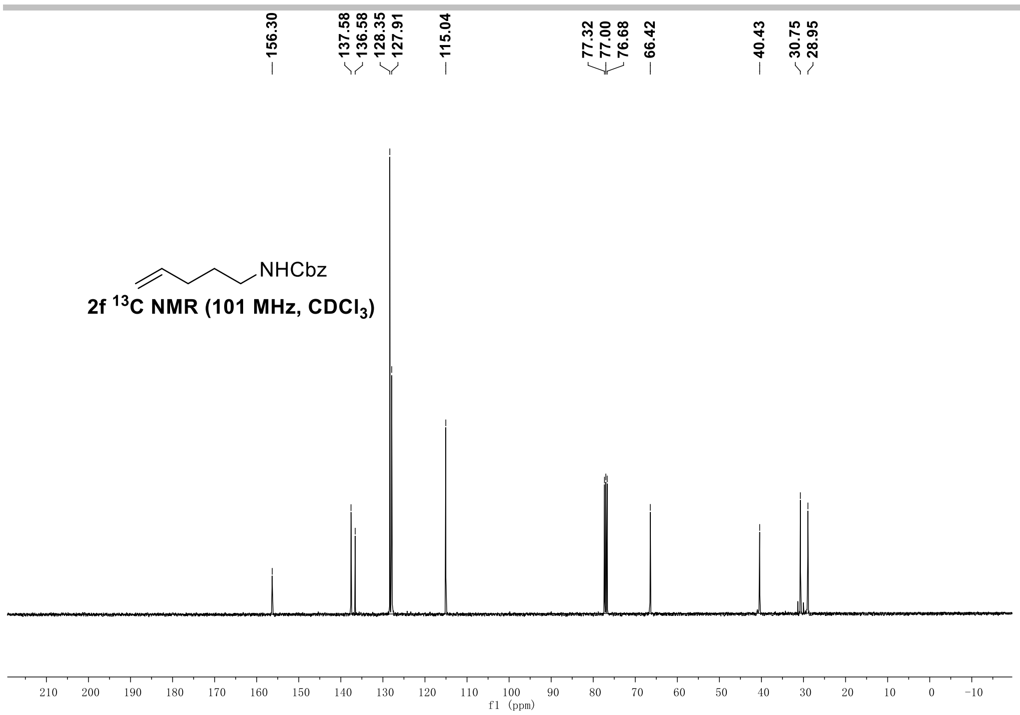
156.30
137.58
136.58
128.35
127.91
115.04
77.32
77.00
76.68
66.42
40.43
30.75
28.95
NHCbz
2f ¹³C NMR (101 MHz, CDCl3)
f1 (ppm)

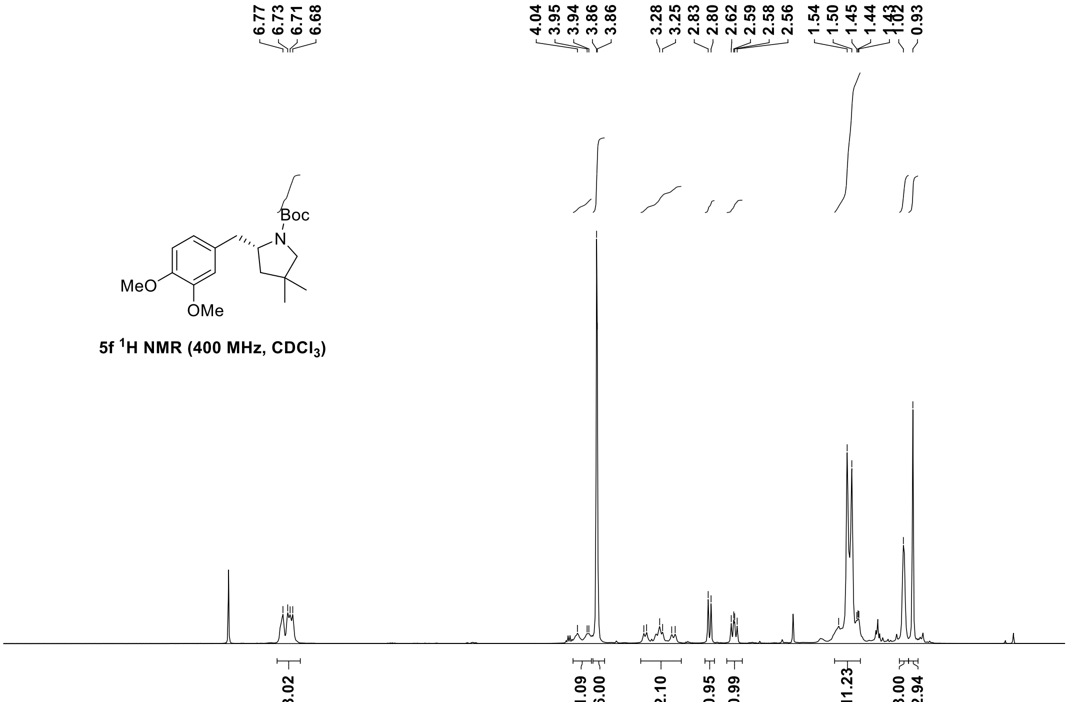
6.77
6.73
6.71
6.68
4.04
3.95
3.94
3.86
3.86
3.28
3.25
2.83
2.80
2.62
2.59
2.58
2.56
1.54
1.50
1.45
1.44
1.43
1.02
0.93
Boc
N
MeO
OMe
5f ¹H NMR (400 MHz, CDCl3)
3.02
1.09
6.00
2.10
0.95
0.99
11.23
3.00
2.94
f1 (ppm)

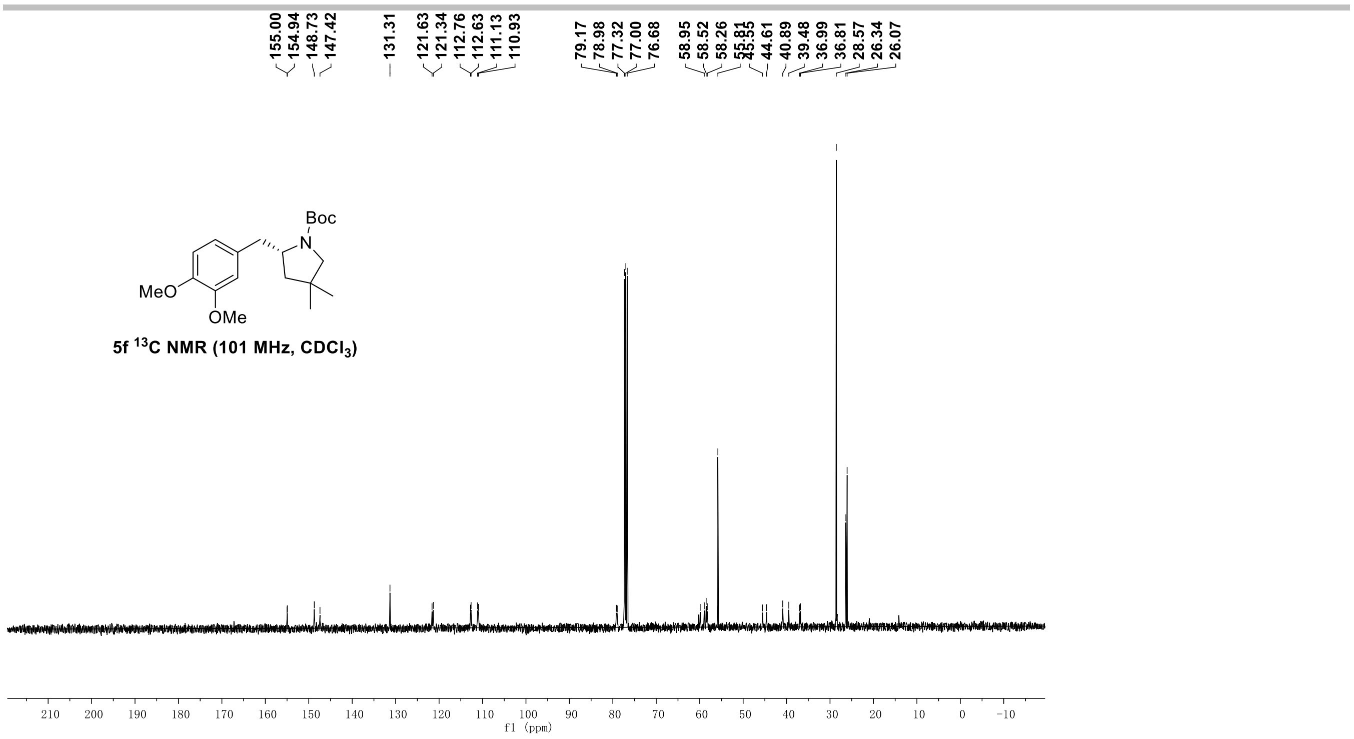
155.00
154.94
148.73
147.42
131.31
121.63
121.34
112.76
112.63
111.13
110.93
79.17
78.98
77.32
77.00
76.68
58.95
58.52
58.26
55.81
55.55
44.61
40.89
39.48
36.99
36.81
28.57
26.34
26.07
Boc
N
MeO
OMe
5f 13C NMR (101 MHz, CDCl3)
210 200 190 180 170 160 150 140 130 120 110 100 90 80 70 60 50 40 30 20 10 0 -10
f1 (ppm)

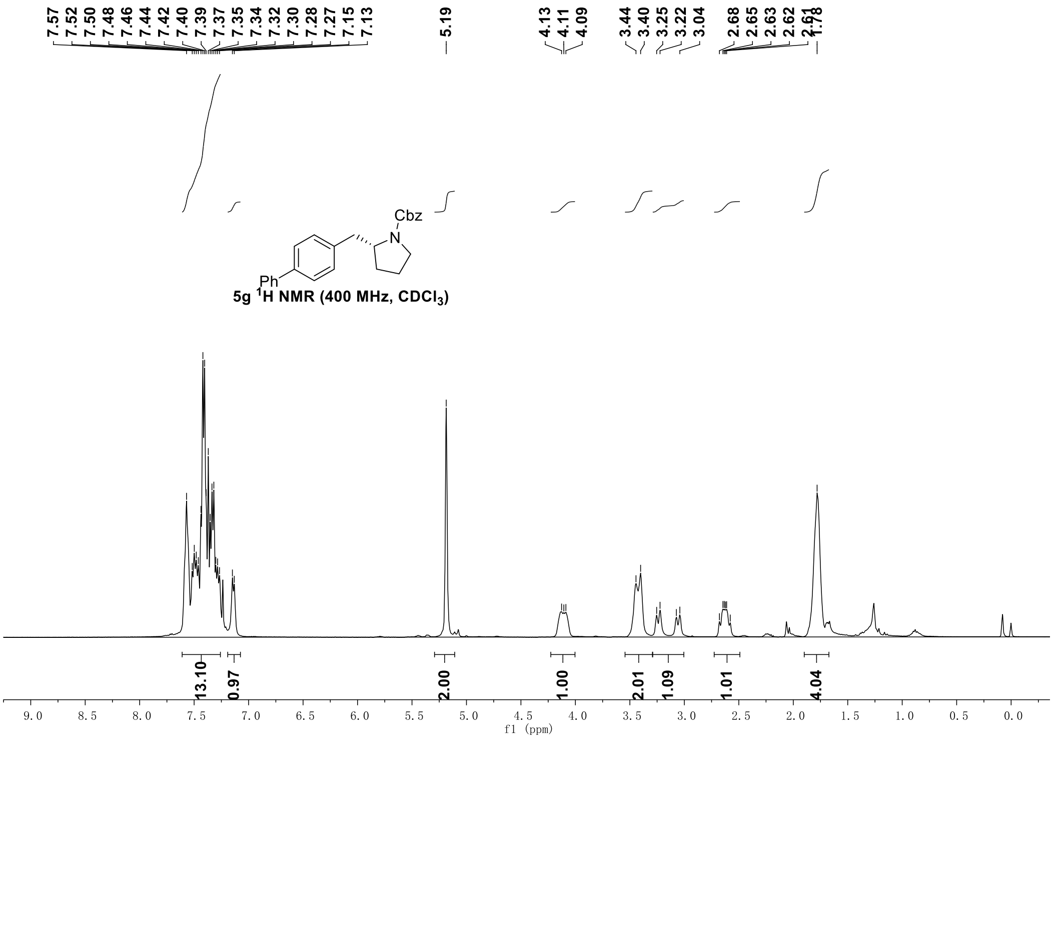
7.57
7.52
7.50
7.48
7.46
7.44
7.42
7.40
7.39
7.37
7.35
7.34
7.32
7.30
7.28
7.27
7.15
7.13
5.19
4.13
4.11
4.09
3.44
3.40
3.25
3.22
3.04
2.68
2.65
2.63
2.62
2.61
1.78
Cbz
N
Ph
5g 1H NMR (400 MHz, CDCl3)
13.10
0.97
2.00
1.00
2.01
1.09
1.01
4.04
9.0 8.5 8.0 7.5 7.0 6.5 6.0 5.5 5.0 4.5 4.0 3.5 3.0 2.5 2.0 1.5 1.0 0.5 0.0
f1 (ppm)

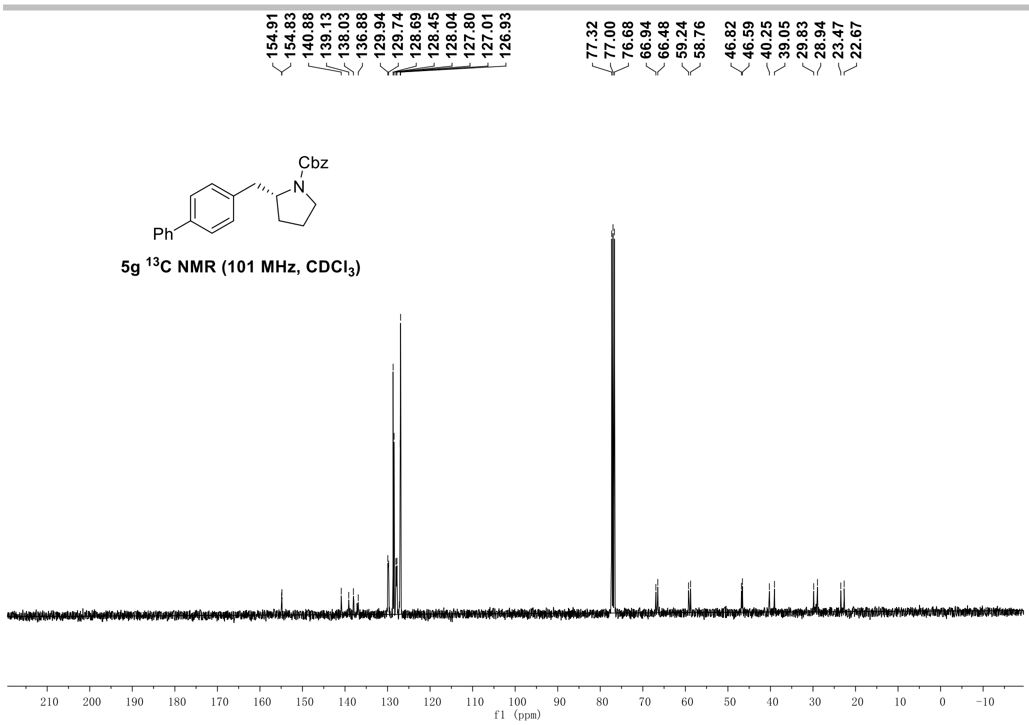

5g ^{13}C NMR (101 MHz, CDCl$_3$)

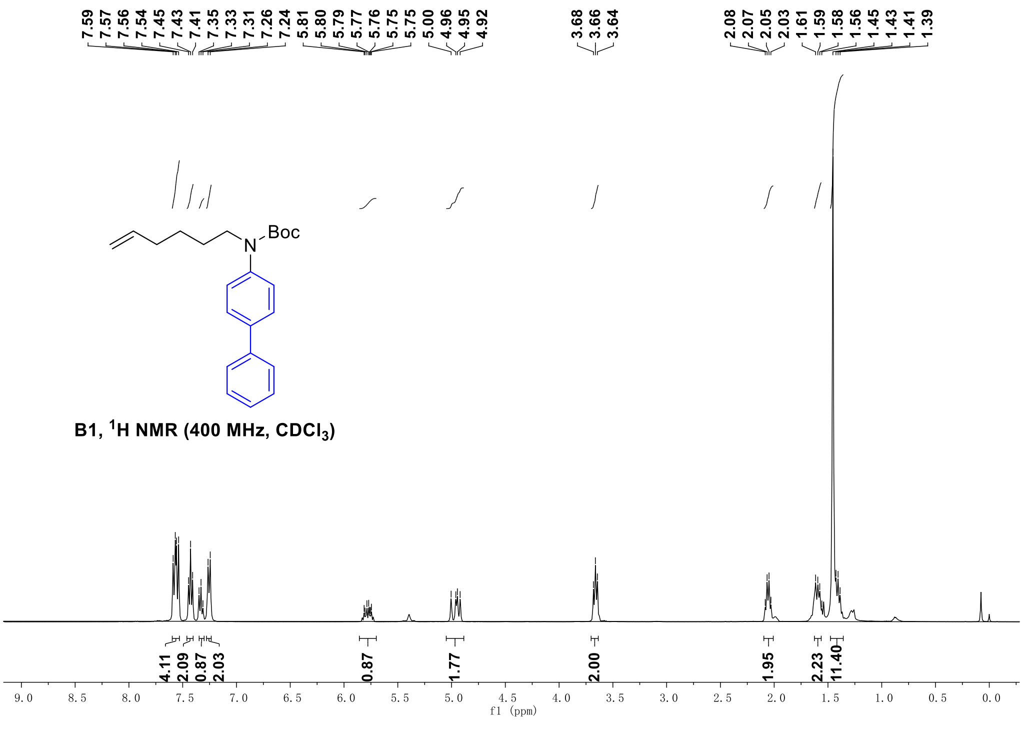

B1, ^{1}H NMR (400 MHz, CDCl$_3$)

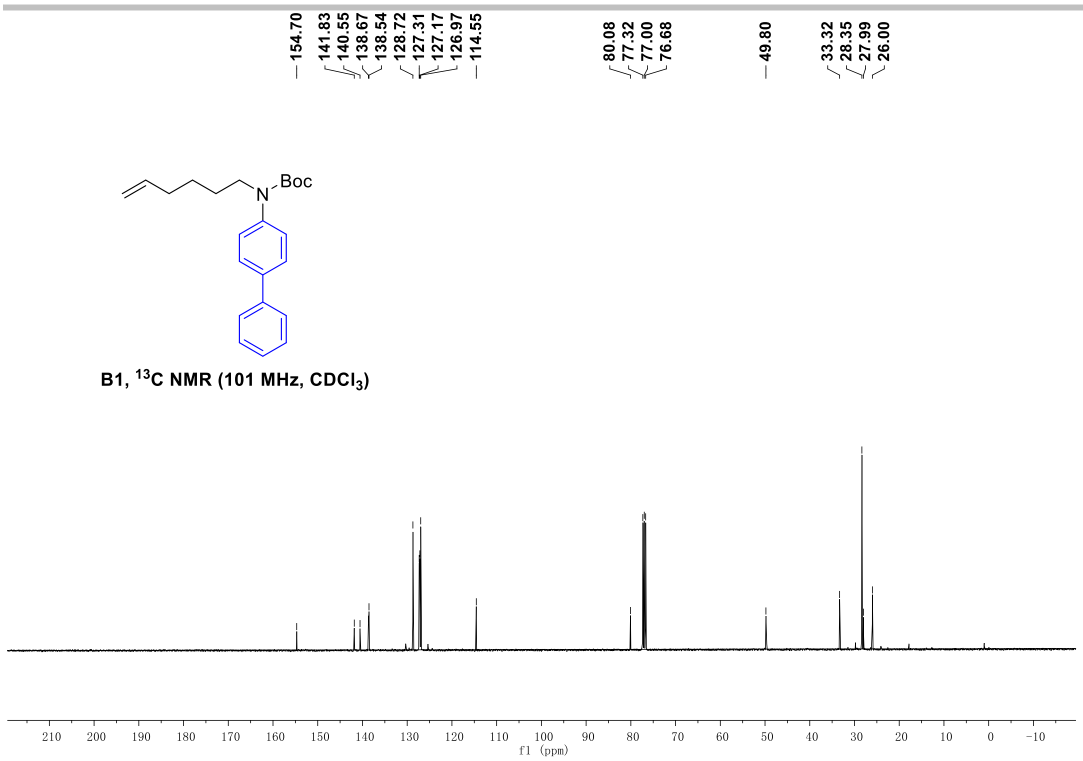

B1, ^{13}C NMR (101 MHz, $CDCl_3$)

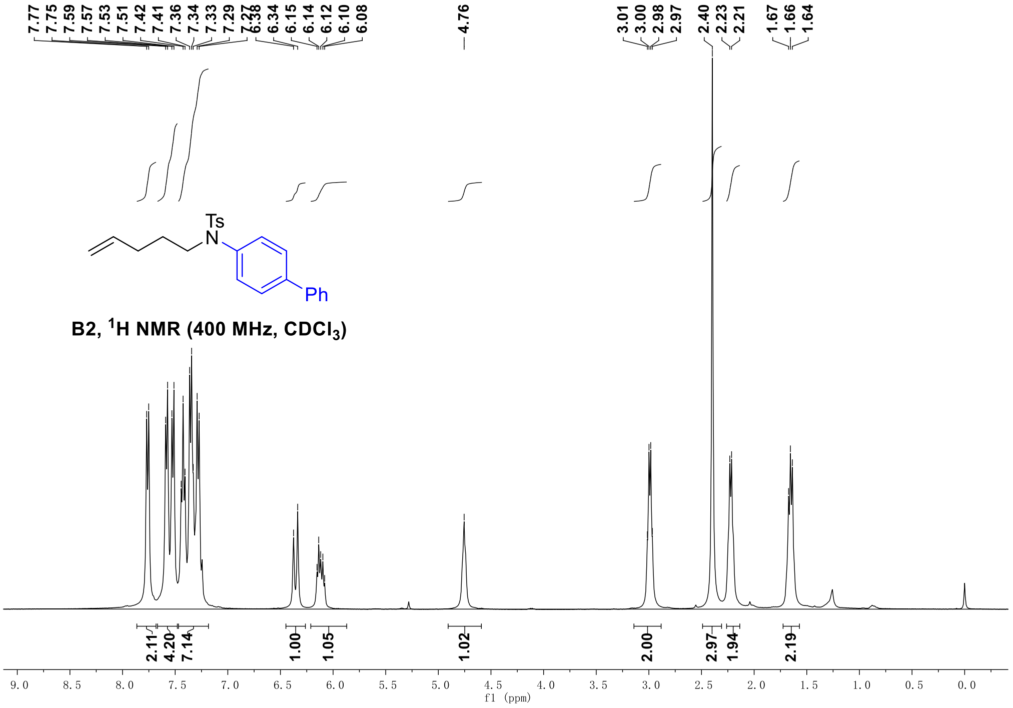

B2, ^{1}H NMR (400 MHz, $CDCl_3$)

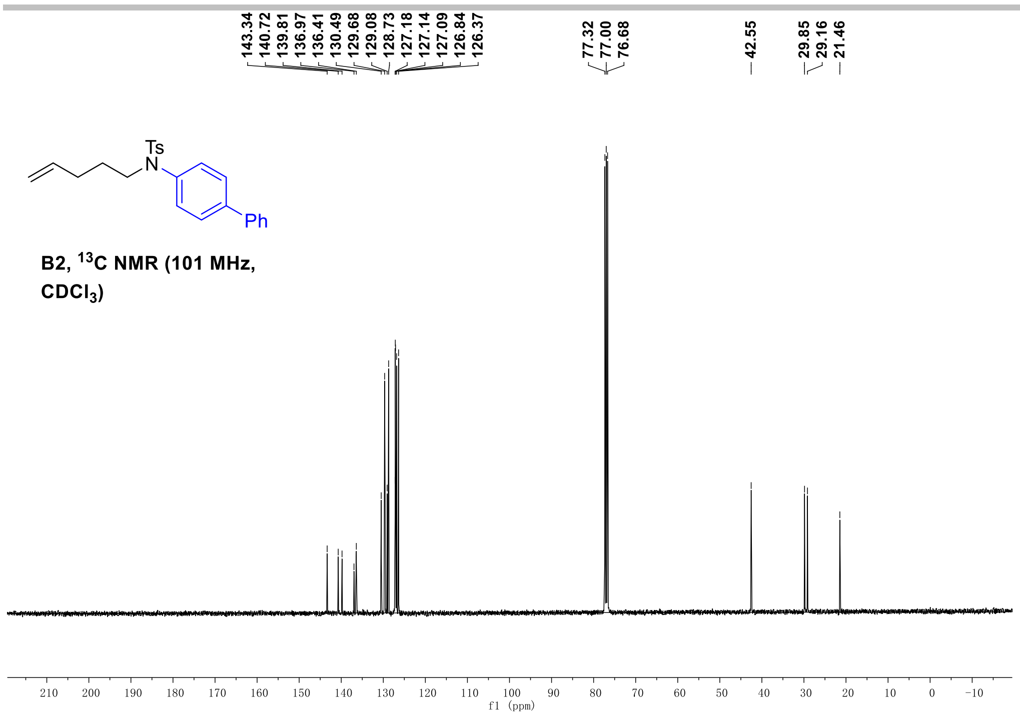

13. Crystal Data Compounds of 3r and (*S*, *Rs*)-Xu4.

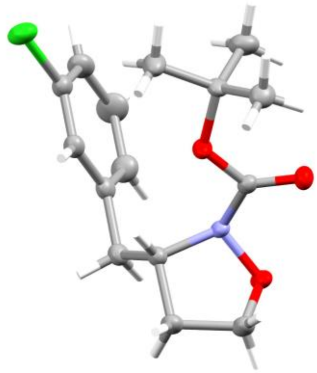

Figure 1. ORTEP representation of compound **3r**（CCDC:2053552）.

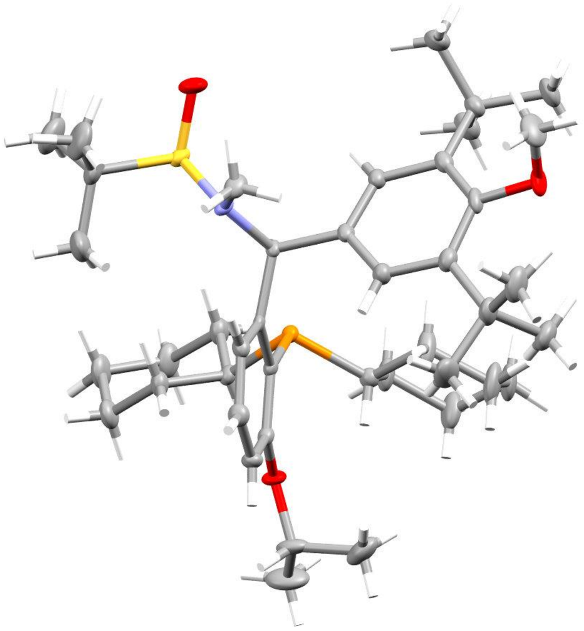

Figure 2. ORTEP representation of compound (*S*, *Rs*)-**Xu4**(CCDC:2053557).

Table 12.1. Crystal Data Compounds of **3r** and (*S*, *Rs*)-**Xu4**.

Compounds	**3r**	**(*S*, *Rs*)-Xu4**
Empirical formula	$C_{15}H_{20}ClNO_3$	$C_{42}H_{68}NO_3PS$
Formula weight	297.783	698.00
T/K	99.99(10)	100.3(7)
Wavelength/Å	1.54184	1.54184
Cryst. syst.	Orthorhombic	Orthorhombic
Space group	$P\ 2_1\ 2_1\ 2_1$	$P\ 2_1\ 2_1\ 2_1$
Flack	0.017(9)	-0.004(6)
a/Å	9.5536(1)	13.71390(10)
b/Å	10.5647(1)	14.69290(10)
c/Å	15.0670(1)	20.80280(10)
α/ °	90	90
β/ °	90	90
γ/ °	90	90
V/ $Å^3$	1520.73(2)	4191.70(5)
Z	4	4
Dcalc (mg m^{-3})	1.301	1.106
μ /mm^{-1}	2.285	1.313
F(000)	635.363	1528
θ range (°)	5.11 to 74.50	3.683 to 67.070
R_{int}	0.0349	0.1439
Data/restraints/parameters	3079/0/184	7476/0/446
GOF on F^2	1.0359	1.039
R_1^a/wR_2^b [$I > 2\sigma(I)$]	0.0228/0.0586	0.0330/0.0822
R_1^a/wR_2^b (all data)	0.0229 /0.0587	0.0338/0.0827

$^aR_1 = \Sigma||F_o|-|F_c||\Sigma/|F_o|$; $^b\ wR_2=\Sigma[w(F_o^2 - F_c^2)^2]/ \Sigma[w(F_o^2)^2]^{1/2}$

14. References

[1] G. S. Lemen, N. C. Giampietro, M. B. Hay, J. P. Wolfe, *J. Org. Chem*. **2009**, 74, 2533-2540.
[2] L. J. Peterson, J. P. Wolfe, *Adv. Synth. Catal*. **2015**, *357*, 2339-2344.
[3] J. Chen, H.-M. Guo,Q.-Q. Zhao, J.-R. Chen W. J. Xiao, *Chem. Commun.*, **2018**, *54*, 6780--6783.
[4] W. Du, Q. Gu, Y. Li, Z. Lin, D. Yang, *Org. Lett.* **2017**, *19*, 316-319.
[5] a) Z.-M. Zhang, B. Xu, Y. Qian, L. Wu, Y. Wu, L. Zhou, Y. Liu, J. Zhang, *Angew. Chem., Int. Ed*. **2018**, *57*, 10373-10377; *Angew. Chem*. **2018**, *130*, 10530-10534; b) Z.-M. Zhang, B. Xu, L. Wu, L. Zhou, D. Ji, Y. Liu, Z. Li, J. Zhang, *J. Am. Chem. Soc*. **2019**, *141*, 8110-8115. c) Z.-M. Zhang, B. Xu, L. Wu, Y. Wu, Y. Qian, L. Zhou, Y. Liu, J. Zhang, *Angew. Chem., Int. Ed*. **2019**, *58*, 14653-14659; *Angew. Chem*. **2019**, *131*, 14795-14801.
[6] S. Cicchi, A. Goti, A. Brandi, A. Guarna, F. D. Sarlo, *Tetrahedron Letters.* **1990**, *31*, 3351-3354.

[7] R. W. Bates, C. J. Lim, *Synlett*, **2010**, 866-868.
